# Expanding the Geographic Characterisation of Epstein–Barr Virus Variation through Gene-Based Approaches

**DOI:** 10.3390/microorganisms8111686

**Published:** 2020-10-29

**Authors:** Marco Telford, David A. Hughes, David Juan, Mark Stoneking, Arcadi Navarro, Gabriel Santpere

**Affiliations:** 1 Institute of Evolutionary Biology (CSIC-Universitat Pompeu Fabra), Department of Experimental and Health Sciences (DCEXS), Barcelona Biomedical Research Park, 08003 Barcelona, Spain; marco.telford@upf.edu (M.T.); david.juan@upf.edu (D.J.); 2 Bristol Population Health Science Institute, University of Bristol, Bristol BS8 2BN, UK; d.a.hughes@bristol.ac.uk; 3 Department of Evolutionary Genetics, Max Planck Institute for Evolutionary Anthropology, 04103 Leipzig, Germany; stonekg@eva.mpg.de; 4 Centre for Genomic Regulation (CRG), Barcelona Institute of Science and Technology (BIST), 08003 Barcelona, Spain; 5 Catalan Institution of Research and Advanced Sciences (ICREA), 08010 Barcelona, Spain; 6 Barcelonaβeta Brain Research Center and Pasqual Maragall Foundation, Carrer Wellington 30, 08005 Barcelona, Spain; 7 Neurogenomics Group, Research Programme on Biomedical Informatics (GRIB), Hospital del Mar Medical Research Institute (IMIM), Department of Experimental and Health Sciences (DCEXS), Universitat Pompeu Fabra, 08003 Barcelona, Spain

**Keywords:** Esptein–Barr virus, human herpes virus 4, genetic diversity, phylogeny, phylogeography, genomics, gene

## Abstract

The Epstein–Barr Virus (EBV) infects the vast majority of human individuals worldwide (~90%) and is associated with several diseases, including different types of cancer and multiple sclerosis, which show wide variation in incidence among global geographical regions. Genetic variants in EBV genomic sequences have been used to determine the geographical structure of EBV isolates, but our understanding of EBV diversity remains highly incomplete. We generated sequences for 13 pivotal EBV genes derived from 103 healthy individuals, expanding current EBV diversity datasets with respect to both geographic coverage and number of isolates per region. These newly generated sequences were integrated with the more than 250 published EBV genomes, generating the most geographically comprehensive data set of EBV strains to date. We report remarkable variation in single-gene phylogenies that, when analysed together, show robust signals of population structure. Our results not only confirm known major global patterns of geographic variation, such as the clear separation of Asian isolates from the rest, and the intermixed relationships among African, European and Australian isolates, but yield novel phylogenetic relationships with previously unreported populations. We provide a better understanding of EBV’s population structure in South America, Africa and, by the inclusion of Turkey and Georgia, we also gain insight into EBV diversity in Western Asia, a crossroads connecting Europe, Africa and Asia. In summary, our results provide a detailed world-wide characterisation of EBV genetic clusters, their enrichment in specific geographic regions, novel inter-population relationships, and a catalogue of geographically informative EBV genetic variants.

## 1. Introduction

Epstein–Barr virus (EBV) is a human herpesvirus chronically infecting 90% of the adult population worldwide [1]. While the EBV primary infection is mostly asymptomatic, it does contribute to the pathogenesis of a variety of diseases. EBV is notoriously linked to cancer [2], with an estimate of over 1.5% of all human cancers being related to EBV [3]. In addition, several autoimmune diseases such as multiple sclerosis have also been epidemiologically linked to EBV [4]. Several of this EBV associated diseases are characterized by strong geographic variation in prevalence, generating hypotheses proposing that such associations are driven by either environmental factors, human genetic variation or both (e.g., multiple sclerosis [5,6,7]). An additional hypothesis suggests that variation in the EBV genome might be related to the differences in prevalence, which has motivated two decades of attempts to identify viral risk variants [8,9,10,11,12]. Despite limited success, these studies have identified a small subset of variants that are statistically-associated with the onset or development of certain diseases [13,14]. However, the identification of disease-linked variants has been generally hindered by limited sample sizes, the presence of geographic stratification in the EBV population, and the consequent risk of spurious associations [1]. 

During the last decade, advances in next generation sequencing and target DNA enrichment have critically expanded our understanding of global patterns of EBV genomic diversity. Phylogeographic analysis performed on increasingly large datasets of EBV sequences derived from different locations, adding up to over 400 sequenced genomes, have provided insights into the geographic distribution of EBV variants [11,14,15,16,17,18,19,20,21,22,23,24,25,26,27]. Broad geographic divisions have been repeatedly observed in these studies, particularly between isolates from East Asian and western countries. However, both additional geographic coverage and a finer resolution of EBV geographic variation would be beneficial to evaluating the links between EBV and disease. Currently, our understanding of EBV genetic diversity is still limited in part due to a combination of two factors: (i) a considerable number of as yet uninterrogated populations, and (ii) a history of pervasive homologous recombination among EBV isolates [28,29,30]. In that regard, a very recent attempt to classify geographic EBV variants while accounting for recombination yielded 12 distinct EBV phylopopulations [30].

Larger population samples, as illustrated by Xu et al., also allow for virome wide association analysis or VWAS, where EBV variants are associated with host phenotypes (nasopharyngeal carcinoma) [14], in Southern China. This result emphasises the importance of persisting in our effort to expand EBV genetic diversity catalogues, increasing geographic representation and the number of isolates per population. 

In this study we generated genetic sequences for 13 selected EBV genes derived from 103 healthy individuals from a wide range of geographic locations. Samples were derived from individuals originating from 9 geographic locations, 5 of which have not been studied to date at the level of whole coding regions (Table 1), expanding the characterisation of the virus from 22 to 27 populations. Gene selection was determined by their genetic variability, relevance to EBV’s biology, and previous disease-association. A particular focus was placed on genes related to latency: *EBNAs, EBERs and LMP-2A*. Latency genes have been consistently shown to be among the most variable genes in the EBV genome, with many exhibiting evidence of persistent positive selection and geographic stratification [22,27,28]. Among them, variation at EBNA-2 and the EBNA-3s define the genetic typing of EBV type-1 and type-2 [31,32], which consistently results in the first split in any EBV phylogenetic tree performed on EBV sequences containing those genes [22,23,27,28]. Further, many viral variants with suggested association to EBV-related diseases are found in latency genes (reviewed in Farrell et al. 2019) [3], underscoring the importance of expanding the resolution of the world-wide map of natural variation of these genes. To help reconstruct complementary phylogenetic relationships, we included a selection of 5 of the 77 non-latency genes which are under higher overall selective constraints [28] and less frequently interrogated: *BDLF4*, *BGLF4*, *BHRF1*, *BRLF1* and, *BZLF1*. These additional genes have crucial roles in the establishment of the lytic phase of EBV’s life cycle, and have also particular clinical interest for their potential associations with a wide range of EBV-related diseases [33,34,35,36,37].

We integrated our dataset with sequences from over 250 publicly available EBV whole genomes and also with the available DNA sequence information from an ancient EBV isolate obtained from ~5700 year old chewed birch pitch from Denmark (Jensen et al. 2019 [38]). We performed gene-based analysis to determine phylogenetic relationships to describe both recurrent and global or gene-specific genetic clusters. The results show both defined global patterns and remarkable differences among individual gene phylogenies, and point to specific genetic variants coincidental with EBV geographic locations, hence providing a framework to define phylogeographic populations. 

## 2. Materials and Methods

### 2.1. Sample Processing and Sequencing

DNA extracted from saliva samples of 103 healthy individuals from nine different geographic locations (Table 1), were obtained from the dataset generated by Nasidze et al., [39]. For each sample we amplified the coding regions of 13 EBV genes, using 12 primer sets. Genes, primer sequences, amplicon length, and other details are listed in Supplementary Tables. PCR reactions were performed using the LongAmp^®^ Taq 2× Master Mix (New England BioLabs) following manufacturer guidelines with an annealing temperature of 61 °C. Blanks were added to each PCR reaction batch to insure the absence of contamination. Amplicons lengths were checked by electrophoresis on a 1.5% agarose gel together with a 200–10,000 bp DNA ladder (NYZDNA ladder III; NZYTech). The amplicons were purified using Solid Phase Reversible Immobilization (SPRI) as described in Meyer & Kircher 2010 [40] with a 1.5× beads/sample volumes ratio. The carboxyl-coated magnetic beads were generated following the protocol described in Rohland and Reich 2012 [41]. 

Amplicons derived from a single individual were equimolarly pooled and transformed into a DNA library following the Blunt-End Single Tube protocol (B.E.S.T. protocol) [42]. Subsequently, libraries from each individual were pooled in equimolar ratios and sequenced on an Illumina MiSeq System at Genomic Regulation Centre’s (CRG, Barcelona, Spain) Genomic Core Facility using the Micro Reagent Kit V2 (Illumina). Base calling was performed using the MiSeq Reporter Illumina pipeline.

### 2.2. Sequence Read Processing

Illumina adapters were trimmed from the sequencing data using Trimmomatic [43]. Trimmed data were filtered for base quality (Phred score ≥ 20), and mapped to the EBV reference (NCBI accession number: NC_007605.1) using BWA [44], filtering for uniquely mapped reads with a minimum mapping quality of 10 using SAMtools [45]. Previously annotated repetitive regions larger than 100 bp were excluded from the mapping (Supplementary Tables). Depth of coverage was computed using the Genome Analysis ToolKit (GATK) [46], using only bases called with a minimum Phred score of 20. For each individual a “callable” genomic region was identified as target spaces with minimum depth coverage of 5 reads passing all quality filters. 

For each gene, an individuals’ callable region was retained if it covered at least 90% of the target coding region. All callable regions retained were then intersected. Comparative analyses were carried out on these intersected callable regions. Variants were called using VarScan 2 [47] using default parameters, and Variant Calling Files (VCFs) were processed using VCFtools [48] and bespoke scripts. Variants called as “heterozygous” were excluded from the analyses, together with multiallelic variants. The short-read alignments generated in this study are available at SRA with accession numbers: SAMN16563520-622; BioProject: PRJNA672338.

### 2.3. Published Sequences

All published and verified EBV complete genomes at the beginning of the project were downloaded from NCBI and separately aligned using MAFFT [49] to the reference genome, to define their relative gene coordinates. Sixty-two sequences were excluded from the analyses because of highly gapped alignments. Both included and excluded sequences are listed in Supplementary Tables. Variants for each sequence alignment were called using custom-made scripts using functions from seqinr package [50]. The R [51] version used in this project was “Eggshell Igloo”, or version 3.5. For each gene, the sequence was intersected with the callable region determined earlier to avoid bias in the comparative analyses. Individuals presenting any gene sequence with more than 10% missing data were excluded from the analysis of the corresponding gene.

### 2.4. Population Structure: Phylogenetic Trees, Supertrees, and Population Pairwise Distances

For each gene, sequences filtered for callability from both newly generated and published sequences were aligned using MAFFT. Alignments were input to PhyML [52] to generate unrooted maximum likelihood trees with 1000 bootstraps using the K80 substitution model [53] (parameters were set as *-d nt -b 1000 - k80 -v e -c 1 -a e -o n*). Trees were joined to generate supertrees using Accurate Species TRee ALgorithm (ASTRAL) [54] using default parameters. 

For each gene, two bootstrapped pairwise distance matrixes were generated by calculating the median values of the median pairwise nucleotide distances, based on the number of pairwise nucleotide substitutions, between all individuals of one population against all individuals of a second population in each one of the 1000 bootstrap trees. The two matrixes were calculated, one using countries of origin to define populations and a second using broader geographic regions (i.e., North Africa, Sub-Saharan Africa, Europe, East Asia, Western Asia, North America, South America, Oceania). Using the ggplot2 R package [55], heatmaps were built to visualise the distance matrixes described above. We performed a second set of heatmaps summarizing the pairwise population distance of all genes by using the median value across the 13 genes.

### 2.5. Definition of Phylogeographic Group for Variant Identification

In order to define phylogenetic groups based on phylogenetic distances, trees were transformed to be ultrametric and midpoint-rooted, and hierarchical clustering was applied using the hclust() function of the R package stats. Each tree was sequentially divided from k = 2 clusters up to k = number of isolates, generating one grouping per each dendrogram, “cut” (grouping 1 = tree divided into 2 clusters, grouping 2 = tree divided into 3 clusters, grouping *n*−1: tree divided in n clusters). In all groupings, clusters containing less than 3 isolates were excluded from the analysis of the correspondent grouping. We then measured the degree of correspondence between the isolates within clusters and the population of origin of the isolates. To obtain that metric of correspondence we calculated 4 indexes: true positive (TP) = number of intra-cluster isolates with the same geographic origin; true negative (TN) = number of isolates in different clusters and with a different origin; false positive (FP) = number of intra-cluster isolates with a different origin; false negative (FN) = number of isolates in different clusters but with the same origin. These values were used to calculate the Matthews correlation coefficient (MCC) using the mcc() function of the mltools R package [56]. MCC ranges from negative 1 to positive 1, where a value of 1 is indicative of perfect structure, a value of -1 is indicative of complete discordance, and a value of 0 is no better than random. In short MCC represents the correlation between clusters and geography and is thus a metric of phylogeographic clustering. In order to determine the optimal number of clusters (k) rendering the highest population correspondence, we defined and calculated a clustering by geography score (CGS) score obtained by multiplying the MCC value by the number of valid clusters (number of clusters after excluding those with less than 3 isolates). The optimal grouping per gene was chosen as the one having the highest value for this CGS score. This approach yielded the best cut option, maximising the overall correspondence to geographic origin of isolates, without losing resolution in terms of cluster number (MCC values and number of valid clusters per grouping are reported in Supplementary Figure S1). 

Additionally, we produced an alternative set of clusters by selecting the grouping solely by maximum MCC value with the same exclusion criteria of the resulting clusters.

For each gene, all clusters derived from the cuts given by maximum CGS or maximum MCC score were tested for significant enrichment of isolates from each country of origin in the dataset by means of a Fisher’s exact test where odds ratios were calculated. Observations were considered significant when the adjusted *p*-value was less than 0.05. Additionally, the same procedure was applied to the supertree, using also the categorization of populations by country or continent of origin. Enrichments were visualized on heatmaps using the ggplot2 R package [55]. The pairs of populations/clusters presenting significant enrichment in this analysis, were tested for variants over-enriched compared to the overall data set. This was tested using a Chi-square test with a significance threshold of 0.05. 

Statistical significances of the comparisons of means between groups were determined with either a Student’s *t*-test or a Mann–Whitney’s U test depending on the normality of the variables (R base and stats packages). Normality of data was tested prior to the application of comparisons tests with the Shapiro–Wilk test (R base package). For both normality and difference tests the significance threshold was set at 0.05.

## 3. Results

### 3.1. Generation of EBV Sequences from Diverse Geographic Origins

Our sequencing effort covered a total of 13 genes (~15% of the total annotated EBV genes), with special focus on the latency group (including 8 out of 9 members, Table 2). We generated high-quality sequences spanning the coding regions of the 13 selected EBV genes by combining long-range PCR amplification and Illumina short read sequencing on DNA extracted from saliva samples from 103 reportedly healthy individuals. After applying strict quality control filters to the sequences in terms of coverage, base calling accuracy and mapping quality (see Methods), we considered only sequences covering at least 90% of the target coding sequence. Gene sequences from 264 out of the 330 published whole EBV genome isolates were also incorporated. This subset of published EBV genomes was composed of sequences passing our quality control; specifically, we retained only those with gaps or unspecified bases (i.e., “Ns”) covering less than 10% of the target coding sequence. This resulted in 13 sets of sequences comprising between 286–364 isolates (Table 2) representing an average 26 countries of origin per gene. These sets of sequences were used to generate multiple alignments and the maximum likelihood phylogenetic trees that would be the basis of our comparative analyses.

We assessed the possibility that samples presented a mixture of isolates from multiple infections by analysing allele balance distributions in QC-filtered variant positions. The average allele balance in our samples was 0.98, with only four individuals presenting values below 0.9 (average allele frequencies: CO_1: 82%, BO_10: 87%, TU_6: 78%, TU_9: 79%). For these putatively multi-infected samples only the dominant strain was considered, since variants of low allele balance were systematically excluded in our filtering procedure.

EBV can be classified in two genetic types by attending at the extreme dissimilarity in the sequence of 4 genes: *EBNA-2* and the *EBNA-3*s. We used genetic variants from *EBNA-2* and *EBNA-3*s for EBV typing and identified 23 type 2 and 305 type 1 isolates. No type 2 strains were found among the new isolates described in this study. Four individuals presented inter-typic recombinant strains of type 1 *EBNA-2* and type 2 *EBNA-3*s, none of which belonged to our dataset of 103 individuals. Two of the four individuals were previously identified as inter-typic recombinants in Correia et al. 2018 [27], but the other two were not. The discrepancy in these two individuals is probably methodological, since Correia et al. 2018 used particular variants for typing, not included in our callable region, and we used alignments of the entire callable coding region. We obtained a 100% agreement in the typing for the rest of the overlapping individuals. Only type 1 strains were used for the analyses on *EBNA-2*, *EBNA-3A*, *EBNA-3B*, and *EBNA-3C*.

Many of the following analyses were performed at two geographic resolutions: country of origin, and broader regions (i.e., North Africa, Sub-Saharan Africa, Europe, East Asia, Western Asia, North America, South America, Oceania). Below, we will sometimes refer to the broader regions as “continents”, even though this is not precise for all mentioned broad regions (e.g., Western Asia).

### 3.2. Genetic Diversity

We observed a wide range of genetic diversity in our entire data set across genes using the Watterson estimator (θ = 0.009–0.08; Table 2). Consistent with previous studies [22,27,28], latency genes show significantly higher diversity than lytic genes (Mann-Whitney U test; *p*-value = 0.009). Latency genes include two short RNAs (*EBER*s) that have been shown to be, in contrast to the rest of this category of genes, very conserved [41,57,58]. When the *EBER*s are excluded from the polymorphism comparison between latency and lytic gene variation, the difference between the two categories becomes more pronounced (Mann–Whitney U test; *p*-value = 0.002). Direct measures of genetic diversity, including the Watterson’s estimator, can be influenced by sample size [59], leading to incorrect comparisons. In our data there is no indication of correlation between number of polymorphisms and sample size, suggesting that the reported difference is actually due to differential selective constraints, as previously suggested [28].

### 3.3. Combining Phylogenetic Information: Method Testing 

In order to find global phylogenetic relationships among isolates, we set out to implement gene-coalescence methods combining the information of the individual genes. For each locus we estimated the phylogeny by maximum likelihood. These were combined using a coalescence-based algorithm to generate a supertree. In order to evaluate an overall population stratification per gene, we began by comparing population diversity (i.e., within population genetic distances) with divergence (i.e., between population genetic distances). The comparison was carried out by calculating, for each gene, the pairwise genetic distances between or within populations in each one of the 1000 bootstraps of the phylogeny. The median of these values for each gene was used as the estimate of genetic divergence or diversity for each pair of populations or within each population respectively, and for each gene. Genetic divergences and diversities were then compared between genes.

Despite considerable variation among genes, there was a substantial difference between divergence and diversity, both estimated as the median the genetic distances observed in 1000 bootstrap trees for each gene. Divergence was always higher than diversity, suggesting stratification (Supplementary Figure S1). When comparing the results for both continental-wise and country-wise classifications, we see similar diversity values (t-student test; *p*-value = 0.07) but significantly higher values of divergence were observed when grouped by country of origin (Student’s *t*-test; *p*-value = 7.7 × 10^−5^). We calculated the ratio of divergence/diversity for each gene and the grouping by country yielded significantly higher values than using broad regions (Mann–Whitney U test; *p*-value = 4.3 × 10^−10^). This suggests that geographic stratification has a relevant role in shaping EBV’s phylogenies, in particular when considering small geographic scales. 

To identify global and recurrent patterns among the phylogenies based on individual genes, we tested three approaches: two based on gene sequence concatenation, and a coalescence-based supertree algorithm (ASTRAL) [54] (Supplementary Figure S2). The two concatenation-based approaches differ in how we dealt with missing data. In one case we selected only individuals represented in all genes; in the other, we filled missing sequences with stretches of Ns (following the reference sequence NC_007605 coordinates). When excluding individuals with missing data in the first concatenation approach, the gene leading to the highest dropout rate was *EBNA-1*, which had a high dropout rate among published datasets, limiting the overall analysis to 164 samples. We thus repeated the analysis excluding *EBNA-1*, which allowed for a total of 227 isolates to be included. We used the same isolates to generate supertrees allowing for the comparison of the two approaches (Supplementary Figure S2a–d). In both cases (with and without *EBNA-1*), the supertrees appear to better separate the isolates per geographic origin than the phylogenies based on gene concatenation, in particular the split between Asian individuals from the rest, which is the most clearly replicated split described to date [22,23,27]. In order to evaluate our observation analytically, we identified clusters in the phylogenies, and measured the correspondence with geographic origin using the Maxwell correlation coefficient (max CGS cut; details in Methods). In phylogenies both with- and without- *EBNA-1*, the correlation was higher in the supertrees than in the trees derived from concatenated sequences (concatenated with/without *EBNA-1*, MCC = 0.32/0.28; supertree with/without *EBNA-1*, MCC = 0.37/0.31). The second concatenation-based approach, based on the substitution of missing data with Ns, allowed for the use of 367 isolates. The resulting phylogenies (repeated both with and without the *EBNA-1* gene), showed a similar scenario to the previous analysis, and groups that were almost monophyletic in the supertree, such as the Asian group, were observed to be divided in the concatenation-based approach (Supplementary Figure S2e–h). When the Maxwell correlation coefficients for these phylogenies were computed, the supertree method again resulted in higher values, although the difference was less pronounced (concatenated with/without *EBNA-1*, MCC = 0.29/0.29; supertree with/without *EBNA-1*, MCC = 0.34/0.31).

These results showed that the supertree approach lead to similar performances in classifying isolates by geographical origin, and that it was more flexible in the handling of missing data with minimum loss of information. Given this, we proceeded with the supertree approach. 

### 3.4. Combining Phylogenetic Information: An Analytical Approach to Define Population Clusters 

We carried out a formal analysis of the isolates’ relationships, clustering and population structure derived from the phylogenetic trees. When generating a supertree, the most representative relationships among isolates are established from a measure of topological recurrence given by the weighted number of times a split appears in the single gene phylogenies. In order to process the information provided by the tree and systematically analyse genetic distances among populations, we calculated the median value of the pairwise distances between all individuals in one population to all individuals in another population. Each of those pairwise distance values were themselves calculated as the median of 1000 bootstrap iterations (see Methods). This median-based approach tolerates the presence of isolates in any particular population that are grouped with another population and that might represent population outliers to the former group. As mentioned before, since classification in type1 and type 2 was the strongest clustering factor for *EBNA-3*s and *EBNA-2* genes, we excluded type 2 isolates from subsequent analysis. Type 2 isolates were included in the analyses of the remaining genes. 

In addition to the median inter-population distances described above, we devised a method to analyse the degree of clustering in each population. We cut gene trees and the supertree at multiple “k”s and calculated enrichments of individuals from each population into the resulting clusters. For some populations, significant clusters appeared at deeper cuts, indicating an older coalescence, while other populations were only enriched at shallow cuts or more recent coalescences. Leveraging this information with the proportion of individuals from each population included in a significantly enriched cluster provides a computational framework for the interpretation of the trees.

**Figure 1:**
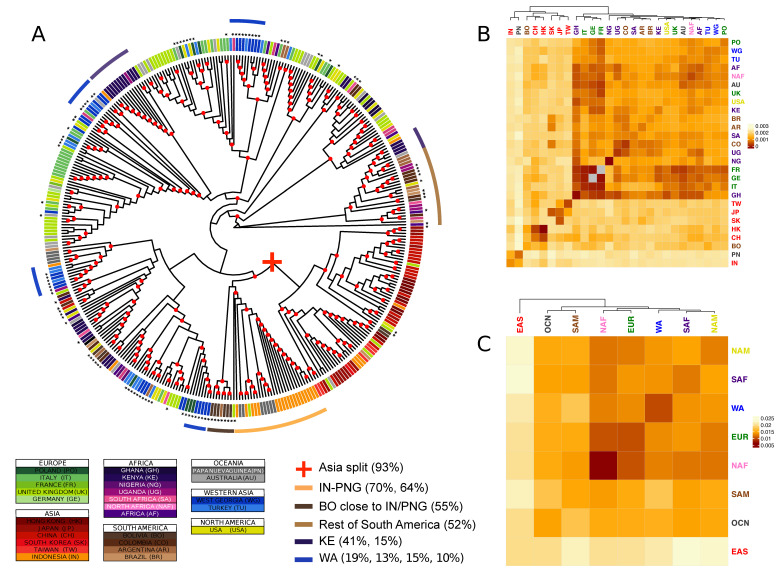
Major phylogenetic relationships. (**A**) Supertree generated by combining the phylogenies of all genes. The strain labels are coloured by country of origin. The countries of the same continent are coloured by different shades of the same colour. The new sequences produced in this study are identified by black asterisks. Nodes with bootstraps values higher than 0.5 are indicated with a red circle. (**B**,**C**) Heatmap representing the bootstrapped pairwise genetic distances between EBV populations weighted per all genes in the data set. Populations IDs correspond to the country of origin (**B**), or the continent of origin (**C**), and are coloured by continent. The colour of the heatmap cells is proportional to the genetic distance value (the closer the populations, the darker red the cell colour). Groups of isolates mentioned in the text are reported together with the proportion of the total number of isolates of the geographical origin.

The analysis of recurrent patterns across genes showed distinct patterns of population stratification (Figure 1). A first and most clear split occurs between Asian and non-Asian isolates, with very few intermixed isolates (the “Asian” branch includes 93% of all Asian isolates in the tree). The proportion of East Asian isolates grouped in clusters that are significantly enriched for this geographic region is above 90% when a minimal number of clusters at early cuts in the tree are generated (i.e., the tree is cut at basal positions), and decreases when cutting the tree at more distal heights (Supplementary Figure S3). Hierarchical clustering analysis applied to the bootstrapped median genetic distances among populations also showed this initial split (Figure 1B,C). Despite East Asian isolates grouping together, the median genetic distance within the East Asian population is significantly higher than for the other populations (one-sample Wilcoxon signed rank test; *p*-value: 5.7 × 10^−10^). This continental heterogeneity is primarily defined by two sub-splits. The first sub-split is between mainland East Asia (China and Hong Kong; 71% and 73% of the total respectively), and the easternmost insular and peninsular populations (Taiwan, South Korea and Japan; 100%, 100% and 58% of the total respectively). This is well distinguishable in the bootstrapped genetic distances and hierarchical clustering analyses, but less conspicuous in the phylogeny. The second sub-split is between South-East Asian isolates (Indonesia; 70% of the total) and the other East Asian populations. More than half of the Papua New Guinea isolates consistently group close to the South-East Asian isolates (Indonesia; 64% of Papua New Guinea isolates). Noticeably, isolates from a single South American country of origin, Bolivia, are found together with East Asian isolates, clustering close to the insular and peninsular subgroup (55% of the total).

As observed in previous EBV whole-genome phylogenetic analysis, non-Asian samples showed more intermingled relationships and lower genetic distances among themselves [22,27]. Often, major splits among non-Asian samples include samples from Africa, Europe, North America and South America, indicating higher admixture levels and a more complex and shared history of recombination events. This is particularly evident in isolates from North America, North Africa and Oceania, for which even the cuts tested closer to the tips of the tree yielded a low proportion of isolates belonging to clusters significantly enriched for those broad regions, if at all (Supplementary Figure S3). A closer examination at a higher resolution (countries of origin), nonetheless, revealed several groups dominated by certain geographic regions. For instance, even if European and African isolates appear highly intermixed, individuals with the same origin tend to form small clusters, as exemplified by the two close Kenya clusters including 41% and 15% of the isolates originated from this country. With the exception of the Bolivian isolates which clustered together with Indonesian samples, around half of South American samples (44%) were clustered together, with intermingling of isolates mostly from Africa (Figure 1A). The separation of Bolivian isolates from the rest of South American might reflect the ancestry of the population, as the sampling for this population was focused on individuals with self-described American ancestry [39]. This relationship suggests an old interaction between EBV and humans showing concordance with the human settlement of the American continent from Asia through the Bering strait. 

Conversely, isolates from North-America (USA) and Australia displayed low genetic distance with European countries. USA and UK clustered together in the hierarchical clustering analysis of the median phylogenetic distance (Figure 1B).

Interestingly, we observed four groups of EBV isolates from the Western Asia, a group poorly described to date both in terms of sequence characterisation and phylogenetic relationships with other EBV strains. The groups comprised 10%, 13%, 15% and 19% of the total number of isolates from the Western Asia in the supertree. Those groups composed by samples from Turkey and West Georgia appeared closely related to other European and Sub-Saharan African groups. Hierarchical clustering on median inter-population genetic distances resulted in the same pattern (Figure 1B), but clustered the two Western Asian countries together showing low values of within-region genetic distances compared to inter-regional values, providing evidence of stratification in West Asia. 

In order to understand the contribution of latency and lytic genes to the supertree, we compared two additional supertrees stacking genes from each class (Supplementary Figure S4). The results show two similar layouts, mainly differing in the grouping of the Asian isolates. While the lytic supertree shows Asian isolates separated in two distinct groups, the latency tree maintained the original supertree structure with the first split separating Asian isolates from non-Asian isolates, showing that latency genes are the main drivers of this configuration. When the bootstrapped median genetic distances are taken into consideration, both supertrees results are more structured at the level of countries, with an intra-regional diversity significantly lower than the inter-regional divergence (Mann–Whitney U test; *p*-value: 0.0035 and 0.004, latent and lytic genes respectively). The two supertrees also reaches significance when the same test is performed at the level of continents, with the lytic one showing higher statistical support (Mann–Whitney U test; *p*-value: 0.004 and 0.02 lytic and latent genes respectively). To notice that when the two non-coding genes (*EBER*s) are excluded from the analysis, the statistical support for the diversity and divergence comparisons in the latent supertree increases of an order of magnitude. It is interesting to notice how South American isolates show limited grouping in either the latency or the lytic supertrees, but come together in the supertree based on the complete gene-set, which illustrates the advantage of gene stacking approaches to reveal global patterns that are otherwise obscured. 

EBV is known to be highly recombinant [28,29,60], which could hinder the detection of population structure. We considered whether masking the recombination regions would significantly alter the phylogenetic relationships among isolates in our analysis. To assess the effect of recombination regions within coding regions in our gene-wise analysis we took advantage of the results of a novel approach developed by Zanella et al. 2019. These authors masked likely recombinant regions from the genomes of around 180 EBV isolates and reconstructed a recombination-free phylogeny. By clustering the masked sequences, they could define 12 phylo-populations. Using the subset of samples matching between both studies (109 samples), we compared the genetic distances in our supertree using the phylo-populations classification proposed by Zanella et al. The isolates of the trees were classified by Zanella et al.’s phylo-population instead of geographic origin, and pairwise population genetic distances were calculated. The comparison revealed a close match between datasets, with median genetic distances of individuals within a phylogroup in our tree being significantly lower than those between phylogroups (Mann-Whitney U test; *p*-value = 6.1 × 10^−8^). This points towards a limited confounding effect of the recombination on the results obtained with our approach, and lead to the recombination region to be kept in the analyses. 

### 3.5. Differences and Commonalities in Individual Genes Phylogenies

**Figure 2:**
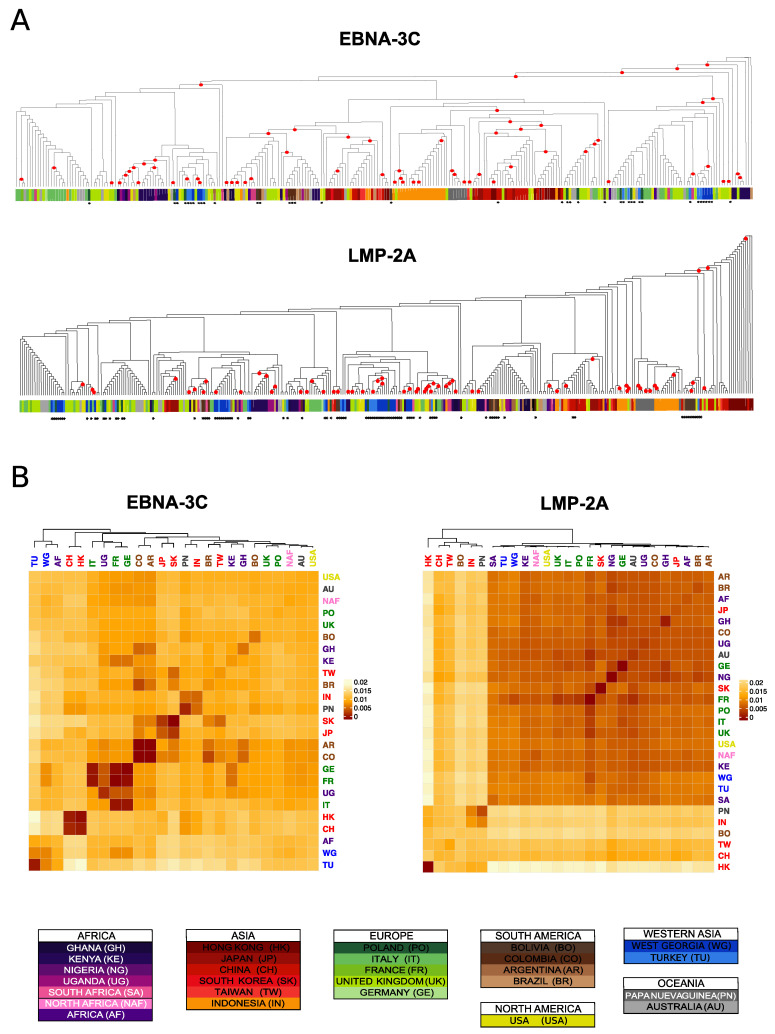
Difference between genes phylogenies. Example of two genes with different genetic structure. (**A**) Cladograms of the maximum likelihood phylogenies are reported for two genes, with the leaves coloured per country of origin. The new sequences produced in this study are identified by black asterisks. Nodes with bootstraps values higher than 0.5 are indicated with a red circle. (**B**) Heatmaps representing the bootstrapped pairwise genetic distances between populations. The heatmap cells colour is proportional to the genetic distance value (the more similar the populations, the darker red the cell colour). Populations IDs correspond to the country of origin and are coloured by continent.

While general stratification patterns become clear when the data from single genes are combined, we observe abundant inter-gene variation among the phylogenetic distances between individuals and populations (examples in Figure 2; all gene phylogenies and bootstrapped pairwise population genetic distances matrices are reported in Supplementary Figure S5). These differences might be in part explained by the variable information content of different genes in terms of genetic diversity, leading to differences in the ability to detect geographic clustering. In particular, conserved genes such as *BDLF4*, or very short ones such as the *EBER*s, allowed only for the recognition of some major splits, while finer scale stratification became more difficult to resolve. 

In the previous analyses of major phylogenetic relationships, we robustly detected an early split distinguishing East Asian from non-Asian isolates. However, this split was detected for only 3 genes (*BGLF4*, *EBNA-1*, *LMP-2A*); for the remaining genes the East Asian individuals were grouped in different and various layouts. Despite multiple differences among trees, individual genes also showed some consistent relationships; for example, the majority of the East Asian isolates are routinely found in groups with low within population genetic distances, and the mainland/peninsular separation we observed in the supertree was evident in the individual trees for several genes (*BRLF1*, *BZLF1*, *EBNA-3A*, *-3C*). In particular, the mainland East Asian isolates (China and Hong Kong), were found grouped together in 7/13 genes, as were Indonesia and Papua New Guinea isolates (Supplementary Figure S5). 

Between genes, tree variability was particularly important among non-Asian isolates, which appeared more intermingled among continental regions. When the median distances within and among these broad regions are compared (Mann–Whitney U test), statistical support for stratification is found in 7/13 genes, even if narrowly significant at the level of nominal *p*-values: *BDLF4*, *BGLF1*, *BZLF1, EBER-1, EBNA-1, EBNA-2*, *EBNA-3C* (*p*-values: 0.031, 0.02, 0.02, 0.04, 0.01, 0.02, 0.01, respectively). Despite the apparent lack of stratification within non-Asian isolates, when we increased the geographic resolution and considered countries instead of continents, the statistical support for lower values of diversity compared to divergence was found in all genes, and with an increase in nominal *p*-value in average of one order of magnitude (Mann–Whitney U test; *p*-values range: 0.04–3.5 × 10^−6^), again a signal of more defined stratification at the level of countries, as we observed in the supertree. Interestingly, we observed a high level of variability in which countries dominated in detected clusters among different genes. An example of this involves the African isolates, which for certain genes showed little country-level specific clustering but for other genes showed Kenya-dominated clusters, which occurred much more frequently than clusters dominated by Ghana or Uganda (Supplementary Figure S5). 

The isolates derived from Western Asian hosts also showed inter-gene variability, from the observed distribution in 4 paraphyletic groups in the supertree. For most genes, the hierarchical clustering of the genetic distances revealed close relationships among these populations, but some genes clustered them closer to African isolates while others clustered them with Europe (Supplementary Figure S5). Taken together, this gene-level analysis indicates that individual genes provide evidence of population structure and unveil both common and specific association patterns among isolates and their countries of origin. 

We tested if geographical patterns displayed by individual genes were correlated with the physical distance among those genes in the EBV genome which would suggests an effect of recombination. A Mantel test [61] between the gene-to-gene phylogenetic distance correlation with a second gene-to-gene matrix based on nucleotide coordinate distance showed no significant correlation, neither grouping per country nor by continent. 

### 3.6. Identification of Geographically Informative Clusters and Genetic Variants

**Figure 3:**
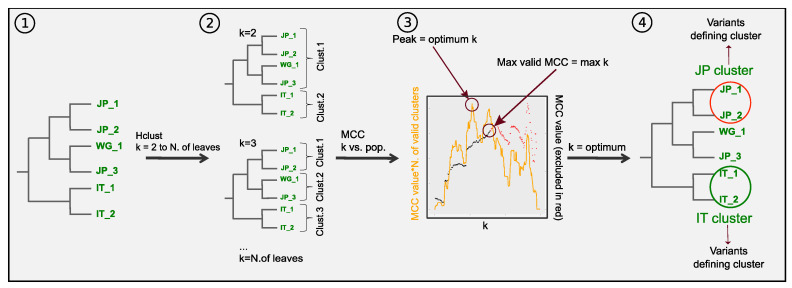
Clustering workflow. For each gene, (**①**) a maximum likelihood phylogenetic tree is generated. (**②**) Hierarchical clustering is used to generate groupings that separate the isolates in increasing number of clusters. (**③**) For each grouping a similarity value with the geographical origin of the isolates is calculated (see methods), as well as the number of valid clusters (i.e., clusters containing at least 3 isolates). The optimum clusterisation is determined as the one giving the highest similarity value for the maximum number of valid clusters. (**④**) The grouping with the optimum number of clusters is taken and for each cluster the odds ratios for the presence of isolates of each population is calculated. The polymorphic positions found in each clusters/origin pair with high odds ratios undergo analysis to calculate statistical significance of the presence of each allele.

We found signals of stratification in our data set characterized by remarkable phylogeographic variability among genes, despite showing general recurrent patterns. We set out to pinpoint the variation originating the stratification signals in each gene identifying the polymorphisms characterizing the geographical population. In order to do so, we choose the cut in the tree maximising the combination of the geographical segregation and the resolution of the obtained clusters. For this, we applied separately to each gene the workflow outlined in Figure 3 (see Methods for a detailed description). This can be summarized as follows: a hierarchical clustering algorithm was applied to each gene’s phylogenic distance matrix and the tree was sequentially cut to yield an increasing number of clusters (from k = 2 clusters up to the number of leaves of the tree). For each tree cut, geographical segregation was assessed according to the matching between clustering classification and known geographic origins using the Matthews correlation coefficient (MCC). The optimum clusterisation (or tree cut) was established as the one maximising a clustering by geography score (CGS) defined as the product between the MCC and the number of valid clusters (see Supplementary Figure S6). Then, the statistical significance of the enrichment of each population in each cluster was calculated (Fisher’s exact test with *p*-values corrected by multiple testing).

Lastly, the polymorphisms in significant population/cluster associations were analysed to determine which variants are significantly over-represented in each significant sub-population cluster. Of note, one cluster can present enrichment in multiple populations. Although this approach can be applied systematically across genes, a tree split based on global optimal CGS might not be equally suitable for all populations, and this could apply to any possible tree-cut optimization method. We decided to complement our analysis with an alternative tree-cut optimization method, and we derived an additional set of clusters using the same set of filters but basing the selection of the tree split only on the maximum MCC score, instead of CGS. This resulted in an alternative grouping, partially overlapping, generally presenting smaller clusters. Heatmaps showing odds ratios for significant cluster/population associations are shown in Supplementary Figure S7).

Subsequent steps to identify genetic variants enriched in population sub-clusters were applied in both sets. The resulting variants per group per gene are reported in Supplementary Tables for max CGS and max MCC respectively. Gene trees clusterings are reported in Supplementary Figure S8.

The total number of single nucleotide variants in our data set was 1726. Around one third of this variation were identified as significantly over-represented (thus putatively involved in the cluster formation) in at least one cluster (range: country: 461–486 SNVs, continent: 526–527), most of which were identified in both the max MCC and max CGS clusters. The overall nonsynonymous/synonymous ratio in the complete data set was 1.55. This ratio varied among genes (Supplementary Tables), with lytic genes resulting generally more conserved than latency genes, but the difference does not reach statistical significance. The *BHRF1* lytic gene is an exception, with a remarkably high nonsynonymous/synonymous ratio of 2.47. When this gene is excluded from the comparison of gene categories, statistical significance was reached (Student’s *t*-test; *p*-value = 0.014). When only considering variants significantly enriched for the population of origin/cluster in each gene, we found a similar scenario in the ratios of nonsynonymous/synonymous changes, with the latency genes showing significant higher value. The difference between nonsynonymous/synonymous ratios measured in all polymorphisms or measured only in variants enriched for origin/cluster pairs was analysed (Fisher’s exact test; Supplementary Tables). *BHRF1* showed significantly lower values in the origin/cluster pair variants, while *BZLF1* and *EBNA-1* showed higher values. This indicates that, for most genes, population informative markers are not biased toward non-synonymous variants compared to the entire set of segregating sites.

**Figure 4:**
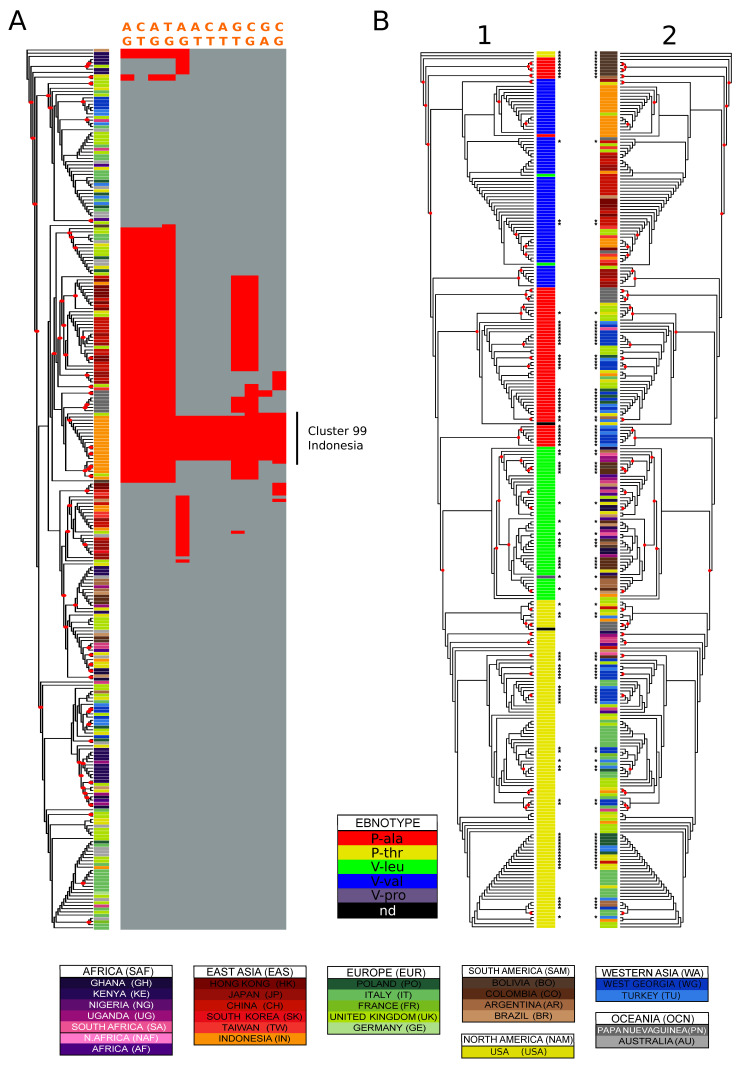
Variation distribution examples. (**A**) characterization of genetic variation in a cluster: Indonesia in EBNA-3C type 1. EBNA-3C type 1 phylogenetic tree coloured by country of origin. The asterisks identify sequences added in this study. The variation of 14 polymorphisms highly enriched in the purely Indonesian cluster number 20 (optimal clustering against country of origin) is shown on the right of the tree. The variant positions relative to the reference sequence are: 86143, 86160, 86212, 86233, 86894, 86926, 86927, 86938, 87557, 88122, 88233, 88901. (**B**) Variation in EBNA-1. EBNA-1 maximum likelihood phylogenetic tree with labels coloured by (**1**) ebnotype (**2**) country of origin. Four isolates with poor alignment around the codons translating for the typing residues were excluded from the analysis. The asterisks identify sequences added in this study, and the red circles nodes with bootstrap values higher than 0.5.

In order to identify the most population informative variants and haplotypes (i.e., variant highly specific for a population or subpopulation), we devised a set of filters based on the proportion of individuals from a given population represented in a given cluster (>30%), the proportion of that population in the cluster (i.e., how diverse is the cluster; >70%) and the difference in allele frequency between the population/cluster and all populations (difference in frequencies > 0.6). This approach yielded multiple instances of population-structured variants and haplotypes. An illustrative example can be found in the *EBNA-3C* gene (Figure 4A), where the hierarchical clustering using both optimal and maximum MCC values divided the Indonesian isolates into two subgroups. The larger of the two clusters, consisting only of isolates from Indonesia, showed an over-representation of 14 variants, most of which were exclusive to these isolates.

As expected, among the set of variants retrieved we found many that were previously and robustly associated with geography and are routinely interrogated in genetic studies of EBV isolates. A well-known example is *EBNA-1*, where a single amino acid change is used to classify subtypes that are known to have differential geographic distribution [62]. The nucleotide determining the change from Valine to Alanine (reference strain coordinate: 97,121), represented in Asian and European/Western Asian isolates (Figure 4B; see the following section for details), significantly distinguishes these regions in our analysis, as well as subgroups of isolates form Sub-Saharan Africa and South America. A similar scenario can be found for the *BHRF1* gene, where the change at position 42,349 (reference strain coordinates), which results in either Leucine or Valine at residue 88, is commonly investigated in sequence analyses of this gene. In our analysis this position characterised different cluster/population pairs from Asian (Taiwan, Japan, China), concordantly with its distribution (Supplementary Figure S9; for details see the following section). In a similar manner, the change at position 42,322, defining the change in either Valine, Leucine or Serine, characterizes groups of isolates from Papua New Guinea, Hong Kong and Indonesia. 

### 3.7. Classifying EBV Geographic Distribution

Early studies on EBV diversity focused on a handful of genes whose variants were used for classification of EBV subtypes. Some of these variants were identified in our geographically informed classification of isolates, discussed in the previous section. We investigated our integrated dataset for these previously reported variants and then considered how they can inform geography in our extended dataset. By combining variants, we calculated haplotype frequencies among the studied populations (Table 3).

*EBNA-1 types*. The EBNA-1 gene has been classified into two major types based on an amino acid residue in the protein C-terminus: Prototype and Variant (P- and V-) [62]. Both types subdivide further in two groups depending on the amino acid residue, which can take the form of A or T for the Prototype (P-Ala, P-Thr), and V or L for the Variant (V-Val, V-Leu). In our data set we found an additional residue at position 487 in a single individual (CO_2). Proline. This polymorphism alone seems to explain the *EBNA-1* tree topology nicely (Figure 4B). *EBNA-1* variants clustered in monophyletic groups, with 2-5 outliers per group (Figure 4B) The P-Ala variant segregated in two regions of the tree: a larger one including isolates form Europe, Western Asia and Oceania, and a smaller one including South American isolates, mostly from Bolivia. As shown in Table 3, Asian isolates mostly presented V-Val; African isolates presented V-Leu, and those from Europe showed P-Thr, and in smaller proportion P-Ala (the latter found mostly in Poland and the UK). P-Ala was also represented in Papua New Guinea isolates. Also, in this classification Western Asian populations appeared closely related to Europe. Both West Georgian and Turkish isolates presented both P-Ala and P-Thr with similar proportions. The new South American populations described in this study presented mostly V-Leu, with smaller proportions of P-Ala and P-Thr. 

*EBNA-2 insertion*. *EBNA-2*, the product of which is essential for B-cell immortalisation, has been another pivotal gene for EBV classification, since it shows both high genetic diversity and several population informative sites. A commonly interrogated variant in this gene is the “CTC” insertion described by Aitken et al. [63]. The insertion was present in most European isolates, and in lower proportion in South America and Western Asia. Countries characterised by European immigration also presented relatively high presence of the insertion: South Africa, Australia and the United States. Additionally, the insertion was found in around half of the isolates originated from Kenya, Hong Kong and Indonesia (Table 3, Supplementary Figure S9). In the *EBNA-2* phylogeny, Western Asian isolates divided into two well-separated groups. The more “European-like” group presented the insertion, while the more intermixed did not, supporting a polyphyletic origin of these populations.

*EBERs variation*. EBV encodes two small RNAs that are abundantly expressed in latency, and that play a range of significant roles in EBV-infected cancer cells [64,65,66]. Wang et al. 2010 [67] characterized EBERs subtypes based on a limited number of polymorphisms. We investigated the polymorphisms falling in the genic region that define groups of isolates in Wang et al., and a pattern appeared in the *EBER-2* versions among the isolates in our dataset: almost all region of origins showed the exact fixed haplotypes. The exception were the isolates from Asia and Oceania, that showed varying base proportions, including notably all the ones originating from Hong Kong, show an almost identical pattern of SNPs, different from all the other isolates (Table 3). The only non-Asian and non-Oceania country that presented variation at these positions was Brazil. 

*BHRF1 variation*. *BHRF1* is a gene involved in the prevention of host cell apoptosis during virus production [68]. *BHRF1* has been shown to be differentially expressed in different EBV-associated cancers [35]. A classification was proposed by Jing et al. [35] based on the variation of 2 *BHRF1* product residues (aa positions 79 and 88). These authors tested 132 isolates derived from Chinese hosts, and classified them in 3 subtypes, showing the predominance of 79V88V. Our expanded data set showed how 79V88V was indeed one of the predominant subtypes, but only in Asian isolates, followed by 79L88L, that was found also in most Western Asians and Papua new Guineans (Table 3). Other isolates almost uniformly presented variant 79V88L (Supplementary Figure S9). 

*5700-year-old Danish sample*. Jensen et al. 2019 [38] sequenced DNA that belonged to a woman who lived 5700 years ago, extracted from a piece of chewed birch found in Syltholm, South Denmark. A tiny fraction of the sequencing data mapped to the EBV genome and presumably belongs to the strain infecting the Danish woman. The limited data allowed us to type the ancient isolate (referred to as “Syltholm_1”, see Table 3) as EBNA-1 to the P-Ala type, elsewhere found in the UK, South Africa, USA, and the Western Asia. In addition, a part of the EBER-2 gene was available for Syltholm_1, showing the pattern of variation fixed in most geographical origin except Hong Kong. 

## 4. Discussion

Understanding the global phylogeographic variation in EBV and its relationship to EBV-associated diseases, including infectious mononucleosis, multiple sclerosis and several types of cancer, has been pursued since the 1980s. This interest has been largely motivated by the identification of epidemiological markers linked to disease [69,70]. Technological advances in targeted genomic capturing and next-generation sequencing [14,22,23,27,71] have greatly accelerated this endeavour.

A proper representation of the geographic pattern of diversity of EBV among healthy individuals, even if still incomplete, is fundamental to ground future research on EBV associations with disease. Our study was designed to survey and characterize diversity and phylogeographic structure in classically studied EBV genes among healthy individuals derived from populations previously uninterrogated.

We produced new EBV genes sequences derived from the saliva of 103 healthy donors and performed an integrated analysis which included additional sequences from 264 published EBV genomes. Novel sequences were derived from a range of geographic origins that covers 5 continents and 9 countries; to the best of our knowledge 5 of these had not been studied previously in the context of EBV genetic diversity.

We generated high quality variant calls from what in the majority of cases represented unique EBV infections per individual. Despite EBV infection leading to an adaptive immune response, this is insufficient to prevent additional transitory infections even in immunocompetent individuals, which is a possible explanation for the pervasive homologous recombination history of this virus. The establishment of multiple life-long infections also seems a recurring phenomenon [72], but its magnitude is still debated [22,72,73]. In our dataset of 103 EBV sequences, four samples exhibited a noticeable proportion of variants with allele balances smaller the 90%. The possibility that this heterogeneity is the product of multiple infections would need to be further studied, for example, by cloning, Sanger sequencing, or long-read technologies.

We found no individuals with evidence of recombination between type 1 *EBNA-2* and type 2 *EBNA-3*s among our 103 individuals, but we identified four recombinant individuals from a published dataset. The reduced number of cases of recombinant *EBNA-2* and *EBNA-3*s is additional evidence of the strong type-specific linkage between these two genes, confirming in our expanded data set the results reported in Palser et al. 2015 [22] and Correia et al. 2018 [27], and in sharp contrast with the high prevalence of inter-typic recombinant forms suggested in Midgley et al. 2000 [74]. Notably, no type 2 *EBNA-2*/type 1 *EBNA-3*s recombinant strain was found, suggesting a possible selective pressure against this genetic configuration.

We also considered the effect of recombination within our genes by comparing our phylogenetic trees with the phylogroups defined in Zanella et al. [30] with masked regions of recombination, obtaining similar relationships. Additionally, Zanella et al. showed that recombination regions are shared by geographic groups and can in consequence contribute to the geographic genetic differentiation among EBV populations. 

In recent years, investigations of EBV diversity have focused on whole genome analysis [14,23,27,30,71]. We instead took an alternative approach focusing on multiple individual genes and complementarily performing phylogeny stacking, or supertrees, and getting useful insight into genetic diversity and divergence. To support our approach, we carried out a method comparison on our data which resulted in similar pattern of population stratification in our phylogeny stacking methodology relative to that observed in gene concatenation, showing robustness between the two approaches. 

When the genes are analysed individually, we found abundant variation in the genetic distances among populations, and in their phylogenetic relationships. This inter-gene variability is likely due to different recombination histories among strains with different origin during the EBV worldwide radiation, as well as to the effect of particular modes of evolution, including selective constraints and local adaptation. Indeed, EBV genes with high levels of genetic diversity and population informative markers host high levels of accelerated evolution and/or positive selection [28].

Combining a gene-wise approach with a global measure across trees has revealed instances of population structure otherwise obscured in either one or the other approach alone. Stacking genes and analysing median distances across genes and bootstraps is more robust to the presence of isolates that fall far away from the bulk of isolates from the same geographic origin. We devised a formal measure of the structure of the overall tree in order to minimise conclusion drawn by visual inspection (MCC method), and to support and systematically produce inferences from the relative genetic distances between and within populations. 

Collectively, our analyses confirmed an elevated gene-to-gene variation, with the presence of recurrent, as well as gene-specific phylogeographic patterns. In type 1 isolates, the clearest split divides Asian isolates from the rest, confirming the scenario found in most studies of the geographic variability of EBV [22,23,27,28,29], as well as in the phylo-populations defined by Zanella et al. 2019 [30]. The remaining isolates tend to group in clusters dominated by a single geographic origin, but more often also contributed by intermingled isolates from different origins. This high-complexity scenario is consistent with the findings of most population structure studies on EBV, except for two studies implementing a different approach. One is Chiara et al. 2016 [23], where the admixed isolates were excluded from the analysis, forcing homogeneity within clusters; another is the previously mentioned Zanella et al. 2019 study [30], where EBV genomes were masked for their recombinant regions, revealing that at least some isolates were placed in the tree distant to other members of their population because of recombination.

The data set we generated allowed us to identify variants and haplotypes localized to specific populations or geographic regions. We showed how a systematic and statistically supported characterisation of EBV variation can provide useful information on the complex population structure of this virus and pinpoint variants with strong geographic structure (caused by genetic drift and/or selection) that are thus potentially relevant for EBV’s biology, local adaptation and pathology.

Paleo-genomics studies have proven critical to understand current patterns of genetic variation in humans, while informing us about multiple aspects, including past migrations, instances of selection and even sociocultural characteristics of ancient populations. The reconstruction of archaic sequences of EBV will be critical to understand the historical dynamics of EBV variants across the world as well as past episodes of selection and adaptation in the context of an also adapting host immune system through geography and time. In this analysis we included EBV sequence data derived from an isolate found to be infecting a 5700 year old Danish woman (Sylthom_1 sample in Table 3) [38]. For *EBER-2*, this ancient EBV strain matched the fixed haplotype world-wide, with the exception of a little part of few populations, mostly Asian. The only exception for this gene is Hong Kong, for which all isolates showed a completely alternative haplotype. *EBNA-1* haplotype composition is highly variable world-wide but also within Europe, and the Syltholm_1 presented the P-Ala variant in residue 487. This variant is present in continental Europe, as well as in countries with a demographic history of European immigration and/or colonization [73,74,75]. Interestingly, it is also found at high frequency in Bolivia and Papua New Guinea. Additional information on the ethnicity of these samples would be needed to draw more accurate conclusions. Despite low coverage and lack of a proper genome-wide characterization of this ancient strain, available variants indicate that certain polymorphisms in extant EBV strains were already present in Europe 5700 years ago. 

Finally, this study reflects an effort to systematically define statistically supported geographic clusters in EBV genes, thereby providing insights into population structure and a catalogue of geographically-informative variants. These new resource and workflow should facilitate the comparison of EBV populations in future sequencing efforts.

## Figures and Tables

**Table 1:** Population of origin representation. The countries for which isolates were added for this study’s analyses are coloured in green, while newly described countries are in bold and coloured in blue. In red we highlight the gene/populations pair for which no isolate passed the quality filters. The number of added isolates is reported in parenthesis together with the country name. Values in the table represent, for each gene the number of isolates per country. * only type 1 isolates. ** = Annotations do not specify country of origin.

				N. Of Represented Strains												
Region	Country	Country_ID	Colour	BDLF4	BGLF4	BHRF1	BRLF1	BZLF1	EBER-1	EBER-2	EBNA-1	EBNA-2 *	EBNA-3A *	EBNA-3B *	EBNA-3C *	LMP-2
**EUROPE**	Poland (10)	PO		10 (10)	10 (10)	10 (10)	10 (10)	10 (10)	9 (9)	9 (9)	10 (10)	9 (9)	10 (10)	10 (10)	8 (8)	9 (9)
	Italy	IT		27	27	27	27	27	27	27	27	27	27	27	27	27
	France	FR		1	1	1	1	1	1	1	1	1	1	1	1	1
	UK	UK		48	46	49	46	47	46	47	34	38	47	48	46	47
	Germany	GE		1	1	1	1	1	1	1	1	1	1	1	1	1
**AFRICA**	Ghana	GH		6	6	6	6	6	6	6	6	5	5	5	5	6
	Kenya	KE		27	27	27	27	27	27	27	3	19	20	20	20	26
	Nigeria	NG		2	2	2	2	2	2	2	1	0	0	0	0	2
	Uganda	UG		6	6	6	6	6	6	6	6	3	5	5	5	6
	**South Africa (4)**	**SA**		4 (4)	4 (4)	4 (4)	4 (4)	4 (4)	4 (4)	4 (4)	4 (4)	2 (2)	3 (3)	3 (3)	0 (0)	4 (4)
	North Africa **	NAF		7	7	7	7	7	6	6	6	5	6	6	6	6
	Africa **	AF		7	7	7	7	7	7	7	6	5	5	5	4	7
**OCEANIA**	Papa New Guinea	PN		11	11	11	11	11	11	9	10	5	7	7	7	11
	Australia	AU		20	20	20	20	20	20	20	0	19	19	19	19	20
**EAST ASIA**	Hong Kong	HK		11	11	11	11	11	11	11	5	11	11	11	11	11
	Japan	JP		10	11	12	10	10	10	12	10	8	11	11	11	12
	China (4)	CH		24 (4)	24 (4)	24 (4)	24 (4)	24 (4)	24 (4)	23 (4)	20 (4)	22 (2)	23 (3)	23 (3)	22 (2)	24 (4)
	South Korea	SK		1	1	1	1	1	1	1	0	1	1	1	1	1
	Taiwan	TW		5	5	5	5	5	5	5	5	3	4	4	4	5
	Indonesia	IN		27	27	27	27	27	27	27	27	22	25	25	25	26
**SOUTH AMERICA**	**Bolivia (11)**	**BO**		11 (11)	11 (11)	10 (10)	11 (11)	11 (11)	9 (9)	9 (9)	11 (11)	7 (7)	6 (6)	5 (5)	3 (3)	10 (10)
	**Colombia (12)**	**CO**		9 (9)	12 (12)	12 (12)	10 (10)	9 (9)	9 (9)	9 (9)	11 (11)	4 (4)	4 (4)	5 (5)	4 (4)	9 (9)
	Argentina (6)	AR		9 (6)	9 (6)	9 (6)	8 (5)	8 (5)	8 (5)	8 (5)	9 (6)	6 (3)	6 (3)	6 (3)	4 (1)	9 (6)
	Brazil	BR		6	6	6	6	6	6	6	6	6	6	6	6	6
**WESTERN ASIA**	**West Georgia (30)**	**WG**		29 (29)	29 (29)	28 (28)	30 (30)	30 (30)	29 (29)	29 (29)	30 (30)	16 (16)	17 (17)	18 (18)	8 (8)	29 (29)
	**Turkey (18)**	**TU**		18 (18)	18 (18)	18 (18)	18 (18)	18 (18)	16 (16)	17 (17)	18 (18)	13 (13)	14 (14)	14 (14)	9 (9)	16 (16)
**NORTH AMERICA**	USA (8)	US		23 (8)	22 (7)	22 (7)	22 (7)	23 (8)	22 (7)	22 (7)	20 (8)	21 (7)	19 (4)	19 (4)	19 (4)	23 (8)

**Table 2:** Variability in the data set. Summary of variability indexes, sample numbers (type1 in brackets), and callable region lengths per gene. Genetic diversity (θw) was estimated using the Watterson’s estimator.

Genes													
	Lytic					Latent							
	BDLF4	BGLF4	BHRF1	BRLF1	BZLF1	EBER-1	EBER-2	EBNA-1	EBNA-2	EBNA-3A	EBNA-3B	EBNA-3C	LMP-2
**Coding region without repeats (bp)**	678	1290	576	1818	738	167	173	1211	1340	2835	2641	2681	1494
**Callable region (bp)**	662	1189	576	1633	675	163	171	1187	1188	2257	1899	2392	1209
**Callable regions (%)**	98	92	100	90	91	98	99	98	89	80	72	89	81
**Polymorphic pos (S)**	49	61	67	117	66	8	27	143	205	307	229	263	184
**Genetic diversity (θW)**	0.0114	0.0079	0.0180	0.0111	0.0151	0.0076	0.0245	0.0193	0.0275	0.0214	0.0189	0.0175	0.0236
**New strains (N. of strains)**	99	101	99	99	99	92	93	102	63	64	65	39	95
**Published strains (N. of strains)**	262	261	265	260	261	259	259	184	234	262	263	260	260
**Final data set (Total N. Strains)**	361	362	364	359	360	351	352	286	279 (297)	303 (326)	305 (328)	276 (299)	355

**Table 3:** Geographical variation distribution. The main typing variation described for EBV is summarised in this table as intra-population percentages. The countries for which specific variants were not described present a “nd” (i.e., no data). The ancient isolate “Syltholm_1” is reported at the end of the table.

		EBNA-1							EBNA-2			BHRF1					EBER-2									
		Res. 16		Res. 487					Insertion pos. 633/4			Res. 79			Res. 88		Pos.44		Pos. 46		Pos. 57		Pos. 61		Pos. 93	
Region	Country_ID	E	Q	A	L	P	T	V	CTC	CTT	No ins.	L	V	S	L	V	T	G	A	T	A	G	A	T	A	C
**EUROPE**	**PO**	40	60	40	0	0	60	0	56	0	44	10	90	0	100	0	100	0	100	0	100	0	100	0	100	0
	**IT**	4	96	4	0	0	96	0	67	0	33	0	100	0	100	0	100	0	100	0	100	0	100	0	100	0
	**FR**	0	100	0	0	0	100	0	100	0	0	0	100	0	100	0	100	0	100	0	100	0	100	0	100	0
	**UK**	38	62	26	6	0	50	18	58	0	42	2	98	0	90	10	100	0	100	0	100	0	100	0	100	0
	**GE**	nd	nd	nd	nd	nd	nd	nd	100	0	0	0	100	0	100	0	100	0	100	0	100	0	100	0	100	0
**AFRICA**	**GH**	83	17	0	100	0	0	0	0	0	100	0	100	0	100	0	100	0	100	0	100	0	100	0	100	0
	**KE**	67	33	0	67	0	33	0	63	0	38	4	96	0	100	0	100	0	100	0	100	0	100	0	100	0
	**NG**	100	0	0	100	0	0	0	0	0	100	0	100	0	100	0	100	0	100	0	100	0	100	0	100	0
	**UG**	83	17	0	83	0	17	0	0	0	100	0	100	0	100	0	100	0	100	0	100	0	100	0	100	0
	**SA**	75	25	25	50	0	25	0	50	0	50	0	100	0	100	0	100	0	100	0	100	0	100	0	100	0
	**NAF**	33	67	0	33	0	67	0	0	0	100	0	100	0	100	0	100	0	100	0	100	0	100	0	100	0
	**AF**	67	33	0	67	0	33	0	0	0	100	0	100	0	100	0	100	0	100	0	100	0	100	0	100	0
**OCEANIA**	**PN**	50	50	50	0	0	20	20	0	0	100	82	18	0	100	0	78	22	78	22	78	22	78	22	78	22
	**AU**	nd	nd	nd	nd	nd	nd	nd	60	0	40	0	100	0	95	5	100	0	100	0	100	0	100	0	100	0
**EAST ASIA**	**HK**	0	100	0	0	0	0	100	55	18	27	73	27	0	73	27	0	100	0	100	0	100	0	100	0	100
	**JP**	50	50	0	0	0	0	100	0	0	100	0	100	0	8	92	100	0	100	0	100	0	100	0	100	0
	**CH**	10	90	0	10	0	15	75	9	0	91	17	83	0	25	75	78	22	78	22	78	22	78	22	78	22
	**SK**	nd	nd	nd	nd	nd	nd	nd	0	0	100	0	100	0	0	100	100	0	100	0	100	0	100	0	100	0
	**TW**	0	100	0	0	0	0	100	0	0	100	0	100	0	20	80	80	20	80	20	80	20	80	20	80	20
	**IN**	11	89	7	4	0	15	74	46	0	54	56	44	0	78	22	85	15	85	15	85	15	85	15	85	15
**SOUTH AMERICA**	**BO**	27	73	55	27	0	18	0	71	0	29	20	60	20	100	0	100	0	100	0	100	0	100	0	100	0
	**CO**	91	9	0	82	9	9	0	25	0	75	0	100	0	100	0	100	0	100	0	100	0	100	0	100	0
	**AR**	67	33	22	56	0	22	0	17	0	83	11	89	0	100	0	100	0	100	0	100	0	100	0	100	0
	**BR**	67	33	0	67	0	17	17	33	0	67	33	67	0	100	0	83	17	83	17	83	17	83	17	83	17
**WESTERN ASIA**	**WG**	40	60	53	0	0	47	0	38	0	63	29	71	0	100	0	100	0	100	0	100	0	100	0	100	0
	**TU**	33	67	44	0	0	56	0	46	0	54	39	61	0	100	0	100	0	100	0	100	0	100	0	100	0
**NORTH AMERICA**	**US**	55	45	25	15	0	50	5	38	0	62	0	100	0	86	14	100	0	100	0	100	0	100	0	100	0
**TOTAL**		**37.4**	**62.6**	**20.6**	**17.8**	**0.35**	**37.8**	**22.7**	**41.6**	**0.67**	**57.8**	**16.3**	**83.2**	**0.55**	**85.7**	**14.3**	**93.2**	**6.8**	**93.2**	**6.8**	**93.2**	**6.8**	**93.2**	**6.8**	**93.2**	**6.8**
**Sylthom_1**		**nd**	**nd**	**+**	**-**	**-**	**-**	**-**	**nd**	**nd**	**nd**	**nd**	**nd**	**nd**	**nd**	**nd**	**+**	**-**	**+**	**-**	**+**	**-**	**nd**	**nd**	**nd**	**nd**

## Supplementary Materials

The supplementary materials are available online at https://www.mdpi.com/2076-2607/8/11/1686/s1.
