# Correction: Telford et al. Expanding the Geographic Characterisation of Epstein–Barr Virus Variation through Gene-Based Approaches. *Microorganisms* 2020, *8*, 1686

**DOI:** 10.3390/microorganisms9112328

**Published:** 2021-11-11

**Authors:** Marco Telford, David A. Hughes, David Juan, Mark Stoneking, Arcadi Navarro, Gabriel Santpere

**Affiliations:** 1 Institute of Evolutionary Biology (CSIC-Universitat Pompeu Fabra), Department of Experimental and Health Sciences (DCEXS), Barcelona Biomedical Research Park, 08003 Barcelona, Spain; marco.telford@upf.edu (M.T.); david.juan@upf.edu (D.J.); 2 Bristol Population Health Science Institute, University of Bristol, Bristol BS8 2BN, UK; d.a.hughes@bristol.ac.uk; 3 Department of Evolutionary Genetics, Max Planck Institute for Evolutionary Anthropology, 04103 Leipzig, Germany; stonekg@eva.mpg.de; 4 Centre for Genomic Regulation (CRG), Barcelona Institute of Science and Technology (BIST), 08003 Barcelona, Spain; 5 Catalan Institution of Research and Advanced Sciences (ICREA), 08010 Barcelona, Spain; 6 Barcelonaβeta Brain Research Center and Pasqual Maragall Foundation, Carrer Wellington 30, 08005 Barcelona, Spain; 7 Neurogenomics Group, Research Programme on Biomedical Informatics (GRIB), Hospital del Mar Medical Research Institute (IMIM), Department of Experimental and Health Sciences (DCEXS), Universitat Pompeu Fabra, 08003 Barcelona, Spain

The authors wish to make the following correction to this paper [1].

The published versions of Figure 1 and Figure 4 have a very low resolution, introduced during the proofreading process, resulting in labels that are difficult to read. Moreover, the phylogenetic tree in Figure 1 lacks color coding, which was explained in the legend but was not shown in the figure.

The authors have provided the complete and high-resolution versions of Figure 1 and Figure 4 to replace the current ones.

**Figure 1:**
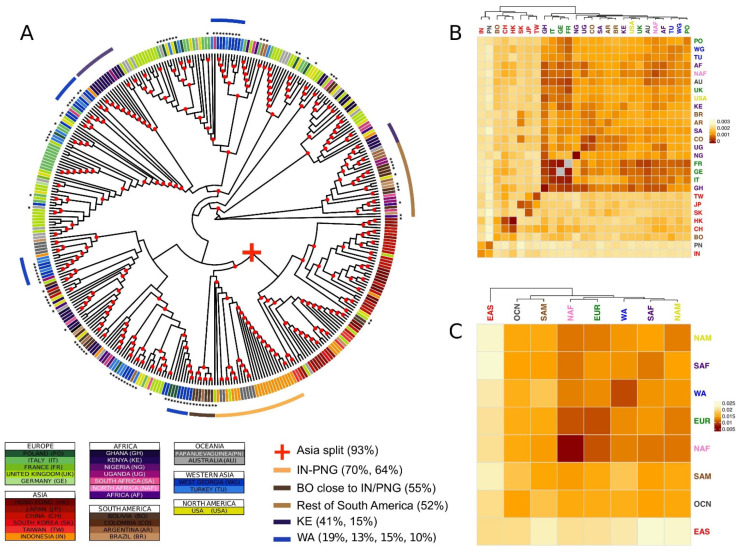
Major phylogenetic relationships. (**A**) Supertree generated by combining the phylogenies of all genes. The strain labels are coloured by country of origin. The countries of the same continent are coloured by different shades of the same colour. The new sequences produced in this study are identified by black asterisks. Nodes with bootstraps values higher than 0.5 are indicated with a red circle. (**B**,**C**) Heatmap representing the bootstrapped pairwise genetic distances between EBV populations weighted per all genes in the data set. Populations IDs correspond to the country of origin (**B**), or the continent of origin (**C**), and are coloured by continent. The colour of the heatmap cells is proportional to the genetic distance value (the closer the populations, the darker red the cell colour). Groups of isolates mentioned in the text are reported together with the proportion of the total number of isolates of the geographical origin.

**Figure 4:**
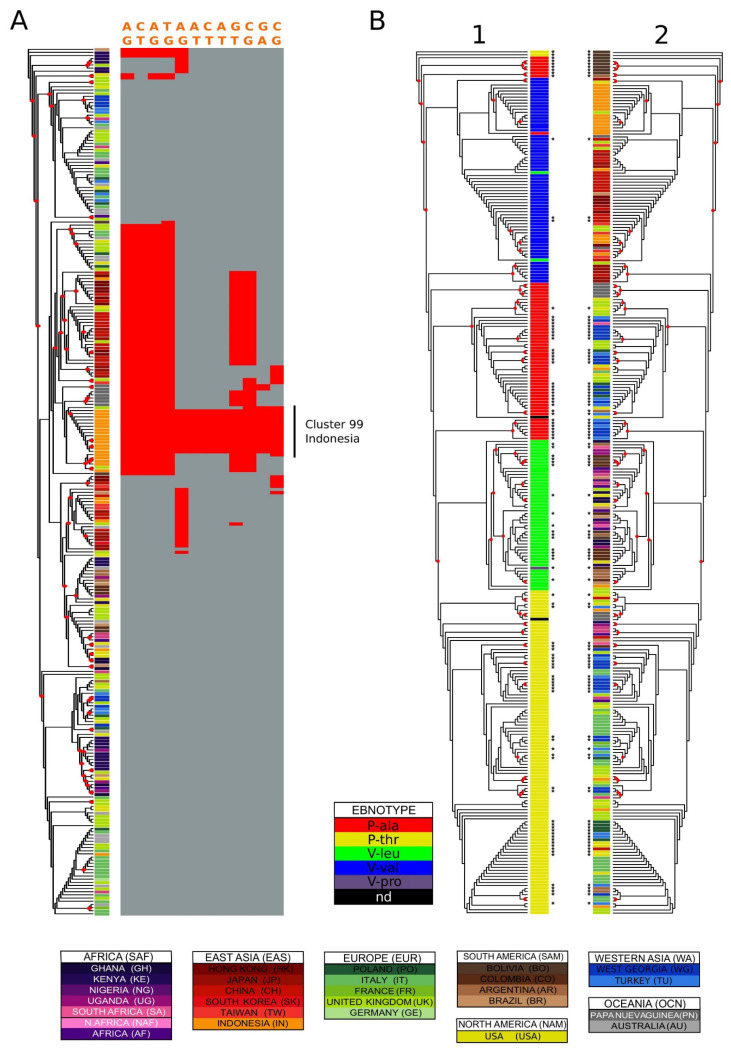
Variation distribution examples. (**A**) characterization of genetic variation in a cluster: Indonesia in EBNA-3C type 1. EBNA-3C type 1 phylogenetic tree coloured by country of origin. The asterisks identify sequences added in this study. The variation of 14 polymorphisms highly enriched in the purely Indonesian cluster number 20 (optimal clustering against country of origin) is shown on the right of the tree. The variant positions relative to the reference sequence are: 86143, 86160, 86212, 86233, 86894, 86926, 86927, 86938, 87557, 88122, 88233, 88901. (**B**) Variation in EBNA-1. EBNA-1 maximum likelihood phylogenetic tree with labels coloured by (**1**) ebnotype (**2**) country of origin. Four isolates with poor alignment around the codons translating for the typing residues were excluded from the analysis. The asterisks identify sequences added in this study, and the red circles nodes with bootstrap values higher than 0.5.

The authors would like to apologize for any inconvenience caused to the readers by these changes.
