# Supplementary material for: Expanding the Geographic Characterisation of Epstein–Barr Virus Variation through Gene-Based Approaches
Source: Microorganisms. 2020 Oct 29;8(11):1686. doi: 10.3390/microorganisms8111686 (PMC7692309; doi:10.3390/microorganisms8111686)
Supplement: Supplementary file 1 [file microorganisms-08-01686-s001.zip › Supplementary/Supplementary_Figure_1.pdf]

Supplementary Figure 1

Genetic population diversity and divergence. Boxplots of the median bootstrapped genetic distances within (diversity) and between (divergence) population. For each gene the average and standard deviation are represented. The populations considered are: **a)** continents, and **b)** countries.

A

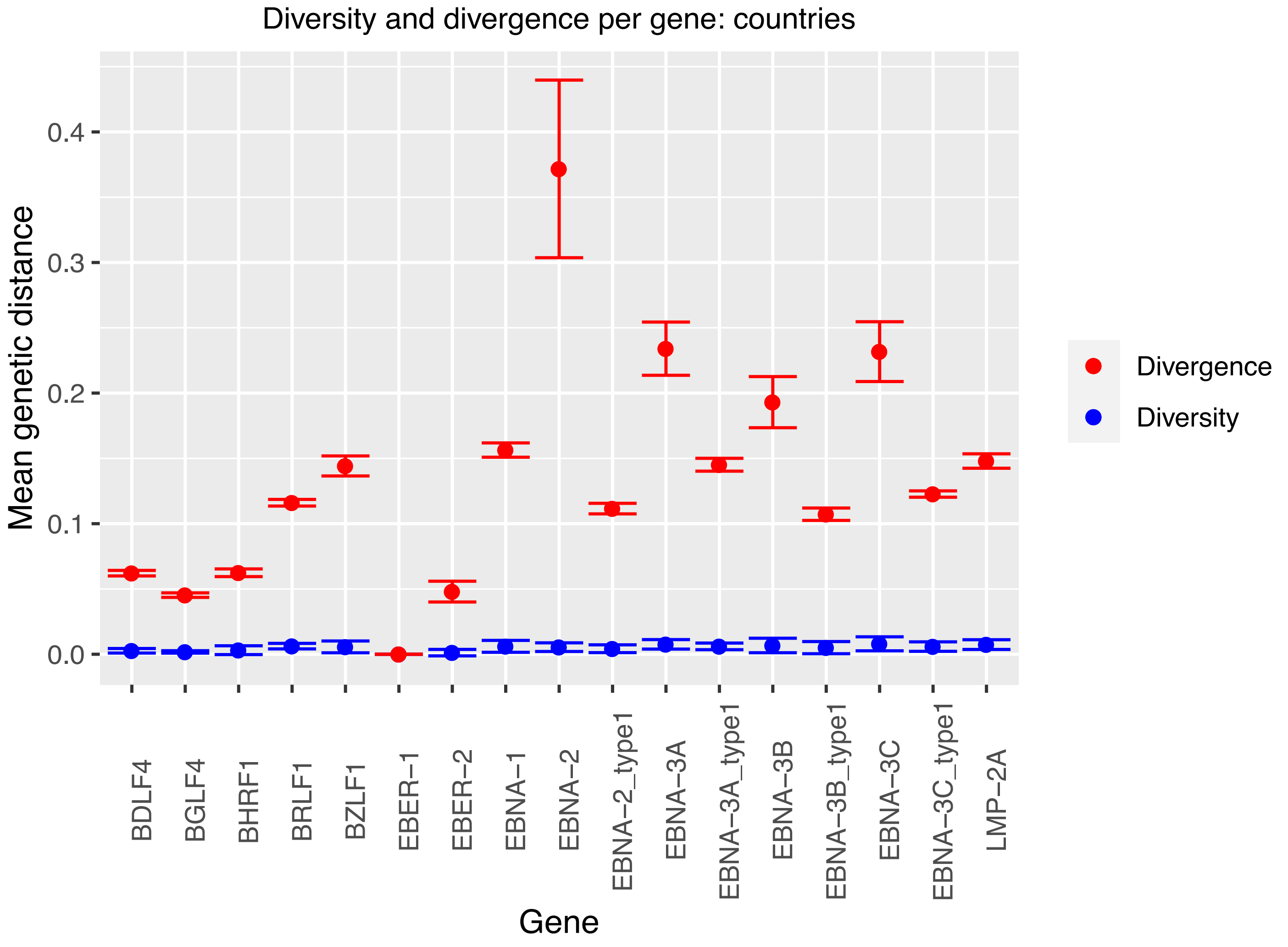

Diversity and divergence per gene: countries
Mean genetic distance
0.0
0.1
0.2
0.3
0.4
BDLF4
BGLF4
BHRF1
BRLF1
BZLF1
EBER−1
EBER−2
EBNA−1
EBNA−2
EBNA−2_type1
EBNA−3A
EBNA−3A_type1
EBNA−3B
EBNA−3B_type1
EBNA−3C
EBNA−3C_type1
LMP−2A
Gene
Divergence
Diversity

B

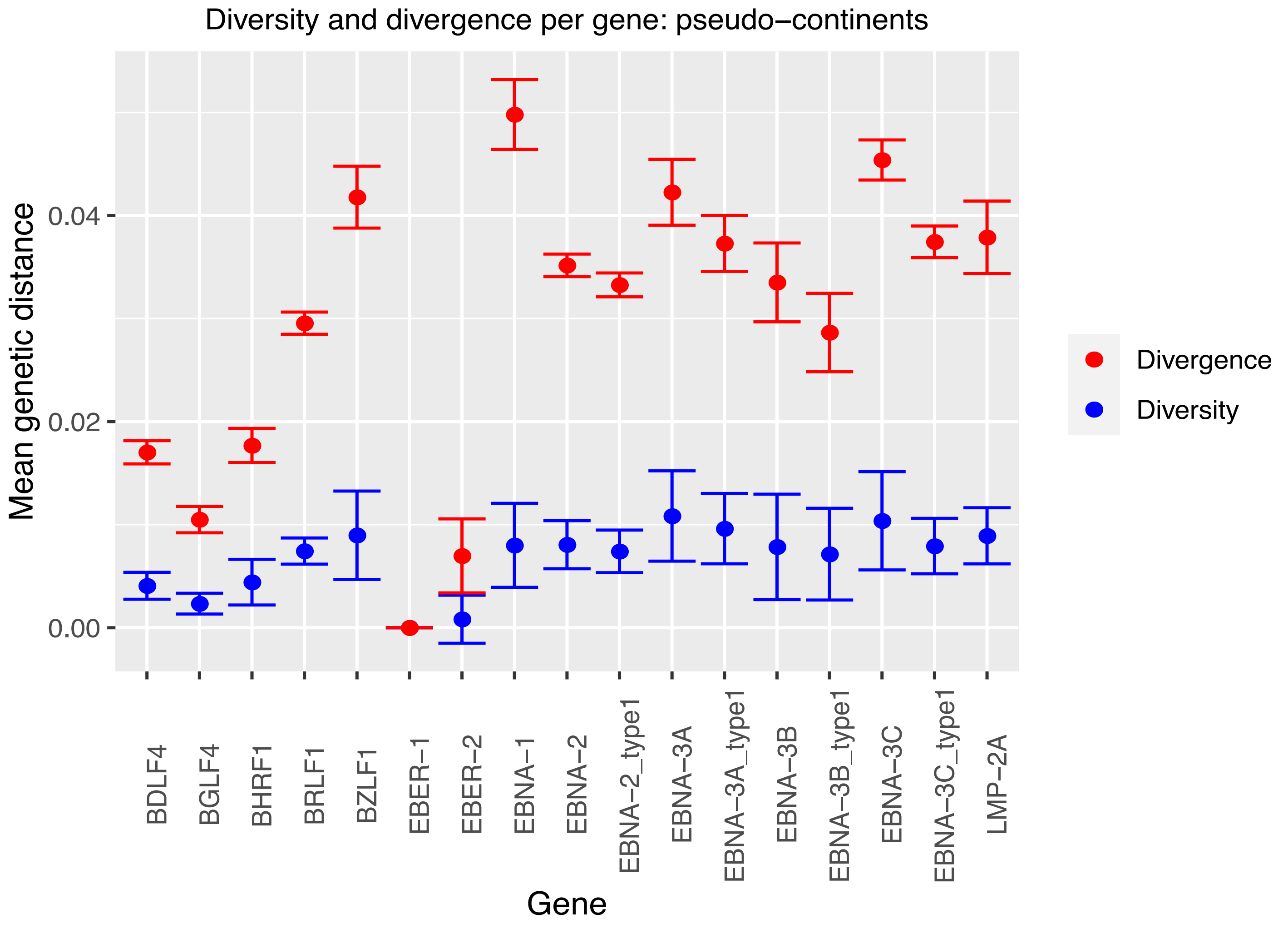

Diversity and divergence per gene: pseudo−continents
Mean genetic distance
0.00
0.02
0.04
BDLF4
BGLF4
BHRF1
BRLF1
BZLF1
EBER−1
EBER−2
EBNA−1
EBNA−2
EBNA−2_type1
EBNA−3A
EBNA−3A_type1
EBNA−3B
EBNA−3B_type1
EBNA−3C
EBNA−3C_type1
LMP−2A
Gene
Divergence
Diversity
