# Supplementary material for: Expanding the Geographic Characterisation of Epstein–Barr Virus Variation through Gene-Based Approaches
Source: Microorganisms. 2020 Oct 29;8(11):1686. doi: 10.3390/microorganisms8111686 (PMC7692309; doi:10.3390/microorganisms8111686)
Supplement: Supplementary file 1 [file microorganisms-08-01686-s001.zip › Supplementary/Supplementary_Figure_2.pdf]

Supplementary Figure 2

Methods comparison: concatenation VS supertree. Comparison of phylogenies produced by concatenation of the genes' sequences with supertrees produced with the same isolates. **a-b)** Concatenation after excluding isolates with missing genes, and correspondent supertree. **c-d)** Same approach but EBNA-1 excluded from the analysis, leading to a higher number of isolates. **e-f)** Concatenation substituting missing genes with Ns, and correspondent supertree. **g-h)** Same approach but EBNA-1 excluded from the analysis. The leaves are coloured per country of origin. The new sequences produced in this study are identified by black asterisks. Nodes with bootstraps values higher than 0.5 are indicated with a red circle. Mind that bootstraps in concatenated phylogenies and in supertrees represent slightly different aspect of the recurrence of the nodes (see method section for details).

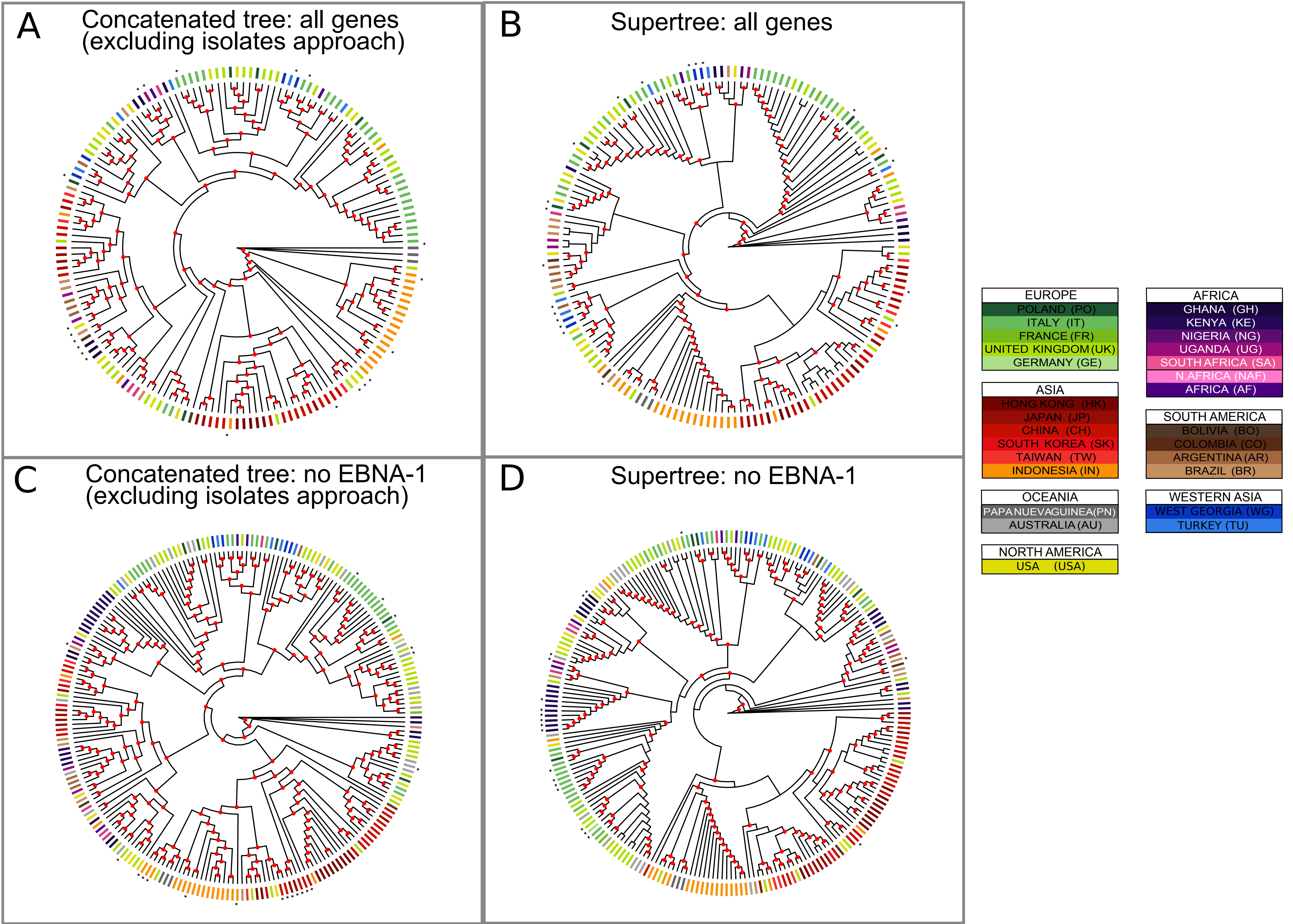
A
Concatenated tree: all genes
(excluding isolates approach)
B
Supertree: all genes
C
Concatenated tree: no EBNA-1
(excluding isolates approach)
D
Supertree: no EBNA-1
EUROPE
POLAND (PO)
ITALY (IT)
FRANCE (FR)
UNITED KINGDOM (UK)
GERMANY (GE)
ASIA
HONG KONG (HK)
JAPAN (JP)
CHINA (CH)
SOUTH KOREA (SK)
TAIWAN (TW)
INDONESIA (IN)
OCEANIA
PAPA NUEVAGUINEA(PN)
AUSTRALIA (AU)
NORTH AMERICA
USA (USA)
AFRICA
GHANA (GH)
KENYA (KE)
NIGERIA (NG)
UGANDA (UG)
SOUTH AFRICA (SA)
N.AFRICA (NAF)
AFRICA (AF)
SOUTH AMERICA
BOLIVIA (BO)
COLOMBIA (CO)
ARGENTINA (AR)
BRAZIL (BR)
WESTERN ASIA
WEST GEORGIA (WG)
TURKEY (TU)

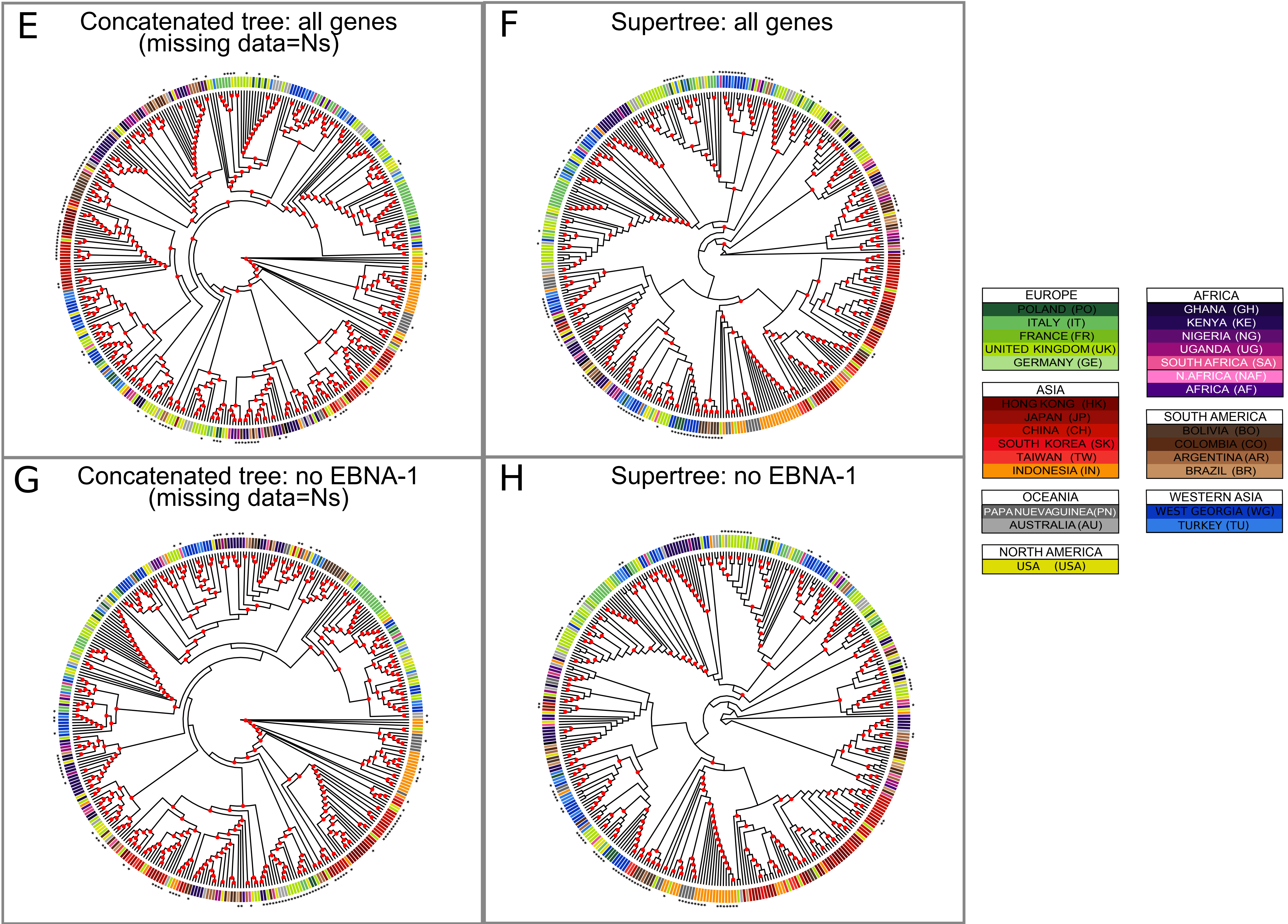
E
Concatenated tree: all genes
(missing data=Ns)
F
Supertree: all genes
G
Concatenated tree: no EBNA-1
(missing data=Ns)
H
Supertree: no EBNA-1
EUROPE
POLAND (PO)
ITALY (IT)
FRANCE (FR)
UNITED KINGDOM (UK)
GERMANY (GE)
ASIA
HONG KONG (HK)
JAPAN (JP)
CHINA (CH)
SOUTH KOREA (SK)
TAIWAN (TW)
INDONESIA (IN)
OCEANIA
PAPA NUEVA GUINEA (PN)
AUSTRALIA (AU)
NORTH AMERICA
USA (USA)
AFRICA
GHANA (GH)
KENYA (KE)
NIGERIA (NG)
UGANDA (UG)
SOUTH AFRICA (SA)
N.AFRICA (NAF)
AFRICA (AF)
SOUTH AMERICA
BOLIVIA (BO)
COLOMBIA (CO)
ARGENTINA (AR)
BRAZIL (BR)
WESTERN ASIA
WEST GEORGIA (WG)
TURKEY (TU)
