# Supplementary material for: Expanding the Geographic Characterisation of Epstein–Barr Virus Variation through Gene-Based Approaches
Source: Microorganisms. 2020 Oct 29;8(11):1686. doi: 10.3390/microorganisms8111686 (PMC7692309; doi:10.3390/microorganisms8111686)
Supplement: Supplementary file 1 [file microorganisms-08-01686-s001.zip › Supplementary/Supplementary_Figure_3.pdf]

## Supplementary Figure 3

**Proportion of the geographical populations in the supertree's clusters.** Representation of the proportion of individual of each population falling in clusters where the population is significantly represented (tested through Fisher's exact test) for groupings generating from 1 to 200 clusters. The red vertical line identifies the optimal chosen clustering. Notice that the total number of clusters is not the one for which the Fisher's tests are applied, as the ones with less than 3 isolates are excluded from the analyses. The comparisons are against **a)** continent, and **b)** country of origin.

A

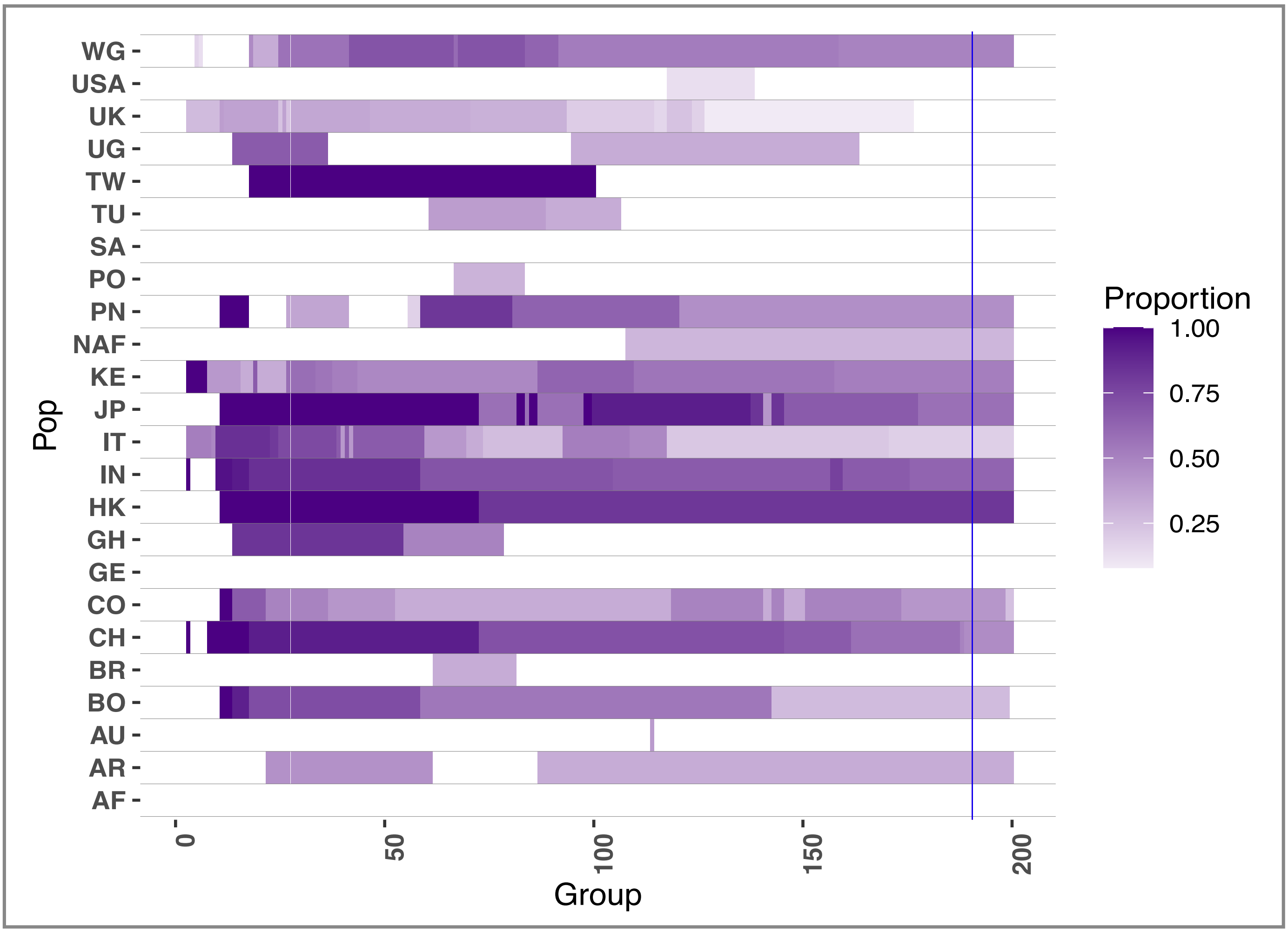
WG
USA
UK
UG
TW
TU
SA
PO
PN
NAF
KE
JP
IT
IN
HK
GH
GE
CO
CH
BR
BO
AU
AR
AF
Pop
0
50
100
150
200
Group
Proportion
1.00
0.75
0.50
0.25

B

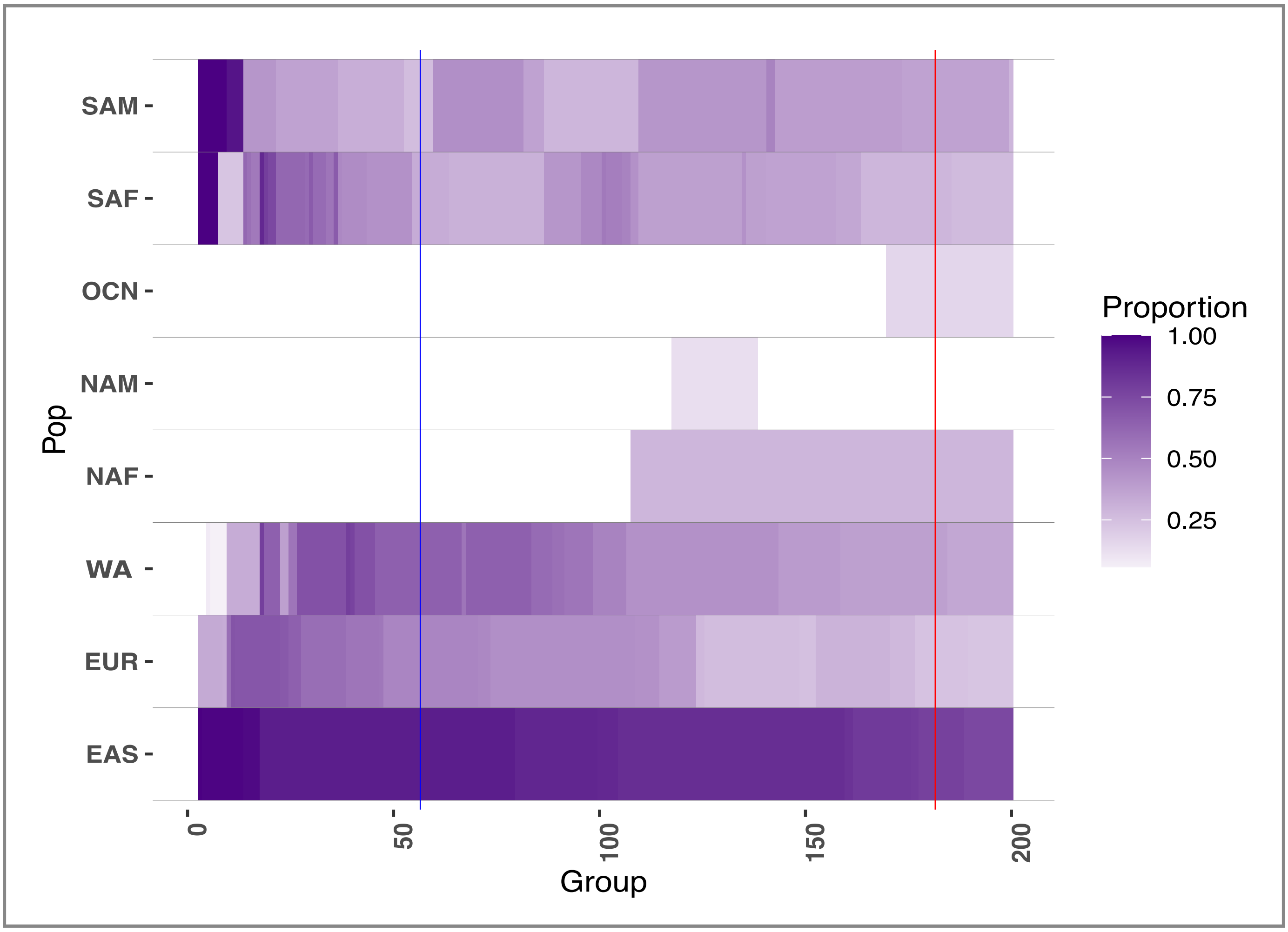
SAM
SAF
OCN
NAM
NAF
WA
EUR
EAS
Pop
0
50
100
150
200
Group
Proportion
1.00
0.75
0.50
0.25
