# Supplementary material for: Expanding the Geographic Characterisation of Epstein–Barr Virus Variation through Gene-Based Approaches
Source: Microorganisms. 2020 Oct 29;8(11):1686. doi: 10.3390/microorganisms8111686 (PMC7692309; doi:10.3390/microorganisms8111686)
Supplement: Supplementary file 1 [file microorganisms-08-01686-s001.zip › Supplementary/Supplementary_Figure_4.pdf]

Supplementary Figure 4

Supertrees per gene class. Supertree generated by combining the phylogenies **a)** Latent genes, and **b)** lytic genes. The strain labels are coloured by country of origin. The countries of the same continent are coloured by different shades of the same colour. The new sequences produced in this study are identified by black asterisks. Nodes with bootstraps values higher than 0.5 are indicated with a red circle.

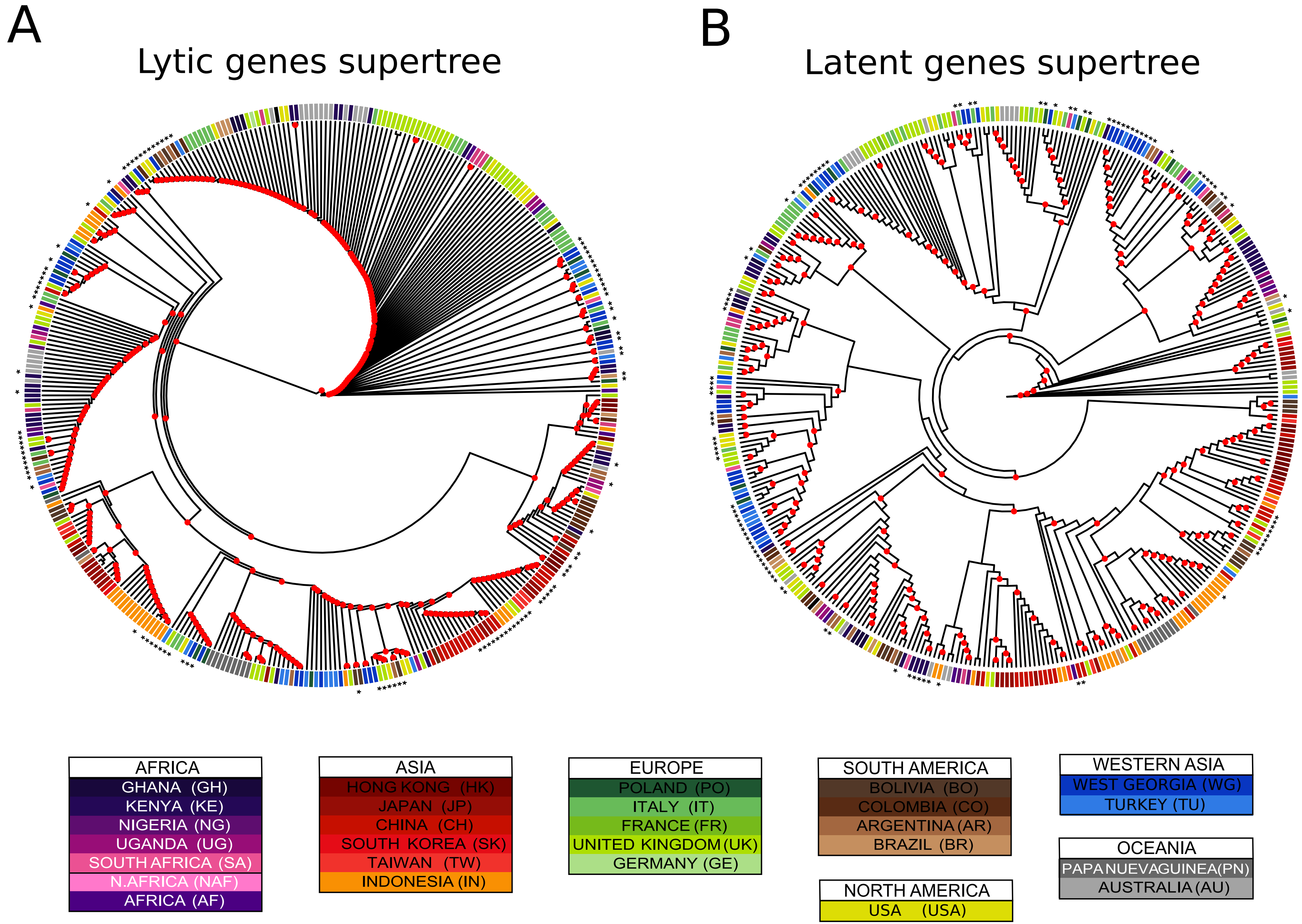
A
Lytic genes supertree
B
Latent genes supertree
AFRICA
GHANA (GH)
KENYA (KE)
NIGERIA (NG)
UGANDA (UG)
SOUTH AFRICA (SA)
N.AFRICA (NAF)
AFRICA (AF)
ASIA
HONG KONG (HK)
JAPAN (JP)
CHINA (CH)
SOUTH KOREA (SK)
TAIWAN (TW)
INDONESIA (IN)
EUROPE
POLAND (PO)
ITALY (IT)
FRANCE (FR)
UNITED KINGDOM (UK)
GERMANY (GE)
SOUTH AMERICA
BOLIVIA (BO)
COLOMBIA (CO)
ARGENTINA (AR)
BRAZIL (BR)
NORTH AMERICA
USA (USA)
WESTERN ASIA
WEST GEORGIA (WG)
TURKEY (TU)
OCEANIA
PAPA NUEVAGUINEA(PN)
AUSTRALIA (AU)
