# Supplementary material for: Expanding the Geographic Characterisation of Epstein–Barr Virus Variation through Gene-Based Approaches
Source: Microorganisms. 2020 Oct 29;8(11):1686. doi: 10.3390/microorganisms8111686 (PMC7692309; doi:10.3390/microorganisms8111686)
Supplement: Supplementary file 1 [file microorganisms-08-01686-s001.zip › Supplementary/Supplementary_Figure_5.pdf]

Supplementary Figure 5

Single genes population cladogram of the phylogenetic trees and bootstrapped pairwise genetic distances between populations. The leaves of the phylogenetic trees are coloured based on the country of origin of the isolates. The new sequences produced in this study are identified by black asterisks. Nodes with bootstraps values higher than 0.5 are indicated with a red circle. Each tree is paired with heatmaps representing the bootstrapped genetic distances among populations for both country and continents. The populations acnoryms are coloured by continent. Coloured bars are used to help to distinguish groups commented on in the text, and the proportion of the total isolates of a geographic origin in the group is reported.

BDLF4

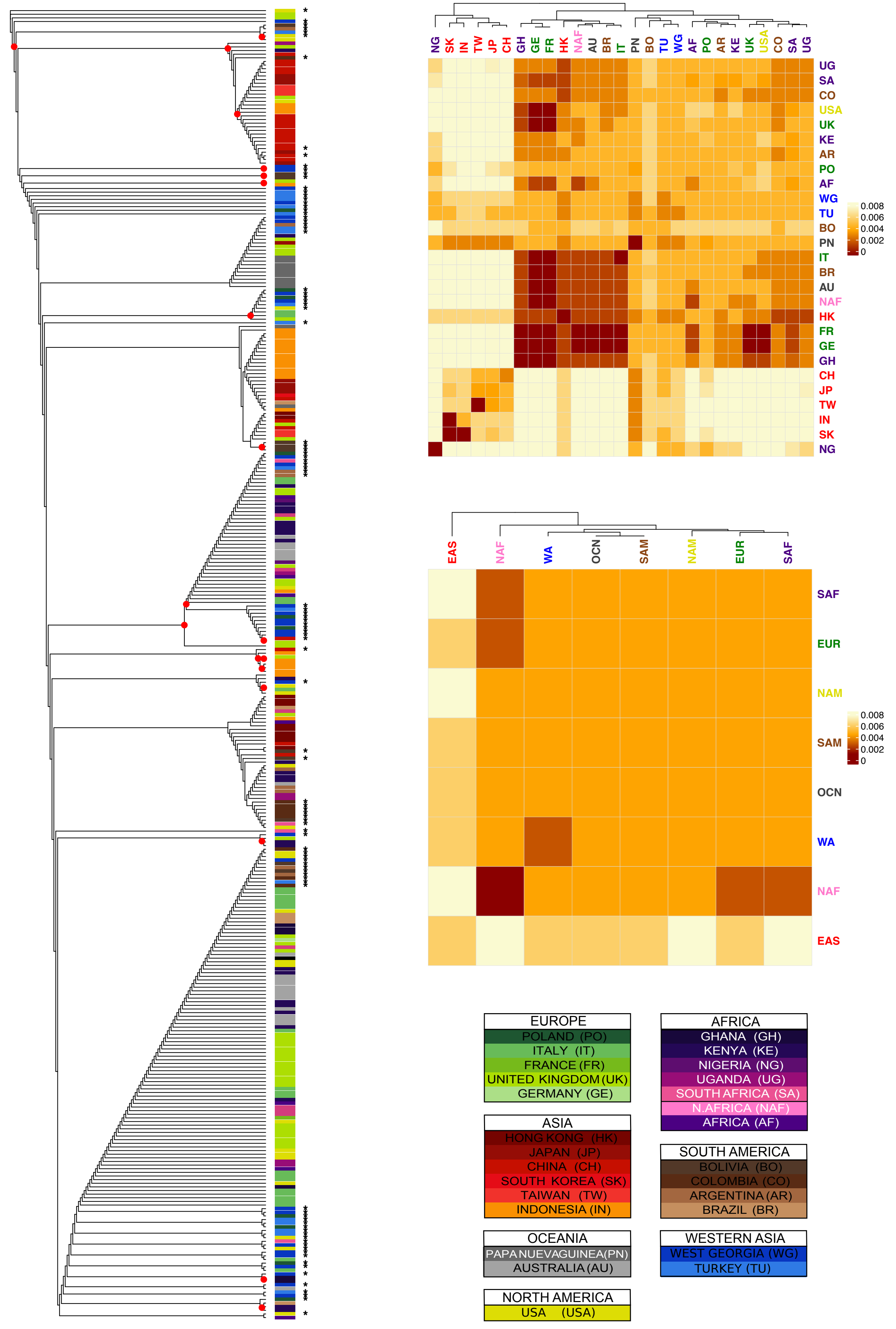

NG SK IN TW JP CH GH GE FR HK NAF AU BR IT PN BO TU WG AF PO AR KE UK USA CO SA UG
UG SA CO USA UK KE AR PO AF WG TU BO PN IT BR AU NAF HK FR GE GH CH JP TW IN SK NG
0.008
0.006
0.004
0.002
0
EAS NAF WA OCN SAM NAM EUR SAF
SAF EUR NAM SAM OCN WA NAF EAS
0.008
0.006
0.004
0.002
0
EUROPE
POLAND (PO)
ITALY (IT)
FRANCE (FR)
UNITED KINGDOM (UK)
GERMANY (GE)
AFRICA
GHANA (GH)
KENYA (KE)
NIGERIA (NG)
UGANDA (UG)
SOUTH AFRICA (SA)
N.AFRICA (NAF)
AFRICA (AF)
ASIA
HONG KONG (HK)
JAPAN (JP)
CHINA (CH)
SOUTH KOREA (SK)
TAIWAN (TW)
INDONESIA (IN)
SOUTH AMERICA
BOLIVIA (BO)
COLOMBIA (CO)
ARGENTINA (AR)
BRAZIL (BR)
OCEANIA
PAPA NUEVAGUINEA(PN)
AUSTRALIA (AU)
WESTERN ASIA
WEST GEORGIA (WG)
TURKEY (TU)
NORTH AMERICA
USA (USA)

BGLF4

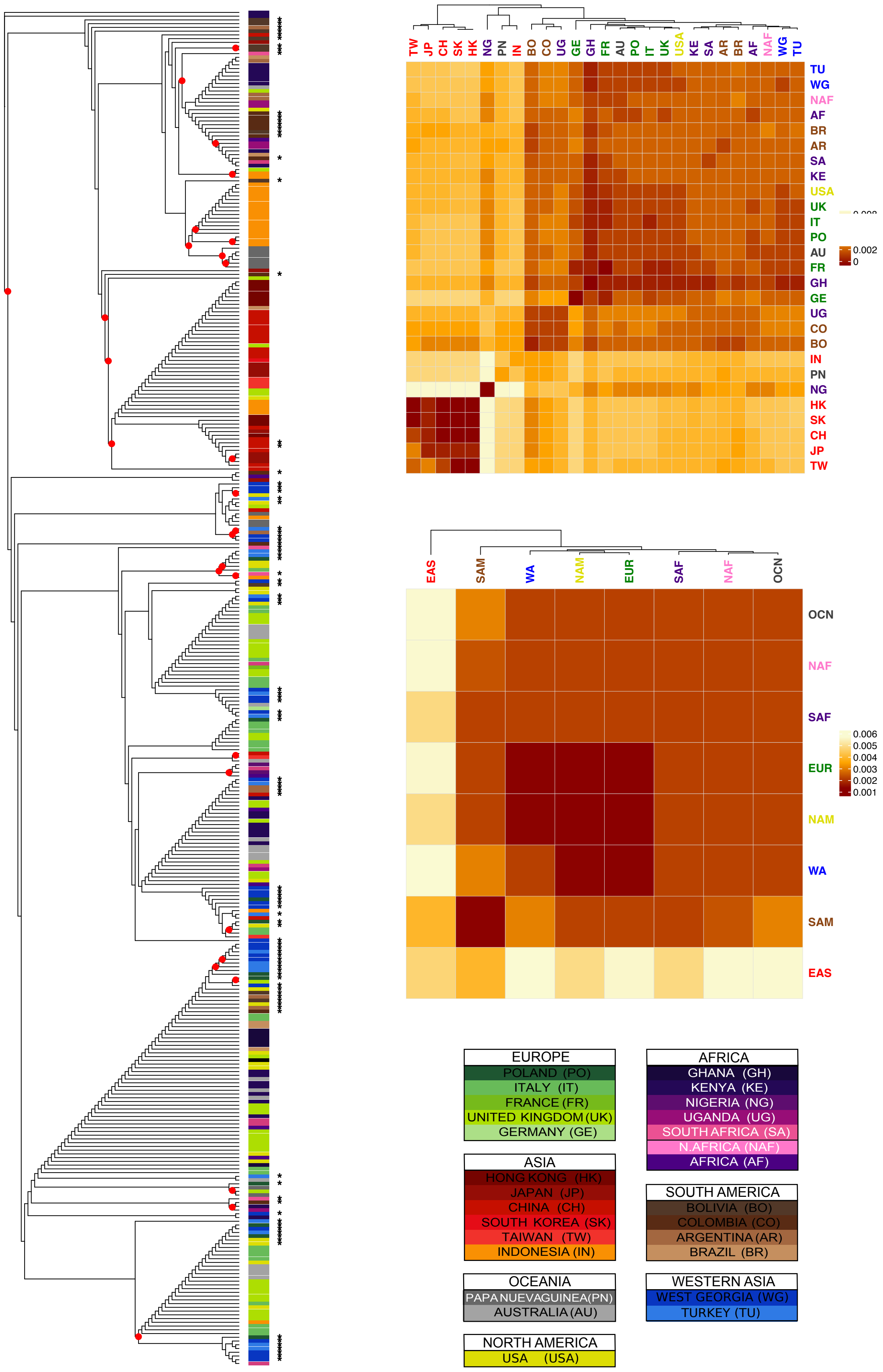

TW JP CH SK HK NG PN IN BO CO UG GE GH FR AU PO IT UK USA KE SA AR BR AF NAF WG TU
TU WG NAF AF BR AR SA KE USA UK IT PO AU FR GH GE UG CO BO IN PN NG HK SK CH JP TW
0.002
0
EAS SAM WA NAM EUR SAF NAF OCN
OCN NAF SAF EUR NAM WA SAM EAS
0.006
0.005
0.004
0.003
0.002
0.001
EUROPE
POLAND (PO)
ITALY (IT)
FRANCE (FR)
UNITED KINGDOM (UK)
GERMANY (GE)
AFRICA
GHANA (GH)
KENYA (KE)
NIGERIA (NG)
UGANDA (UG)
SOUTH AFRICA (SA)
N.AFRICA (NAF)
AFRICA (AF)
ASIA
HONG KONG (HK)
JAPAN (JP)
CHINA (CH)
SOUTH KOREA (SK)
TAIWAN (TW)
INDONESIA (IN)
SOUTH AMERICA
BOLIVIA (BO)
COLOMBIA (CO)
ARGENTINA (AR)
BRAZIL (BR)
OCEANIA
PAPA NUEVAGUINEA(PN)
AUSTRALIA (AU)
WESTERN ASIA
WEST GEORGIA (WG)
TURKEY (TU)
NORTH AMERICA
USA (USA)

BHRF1

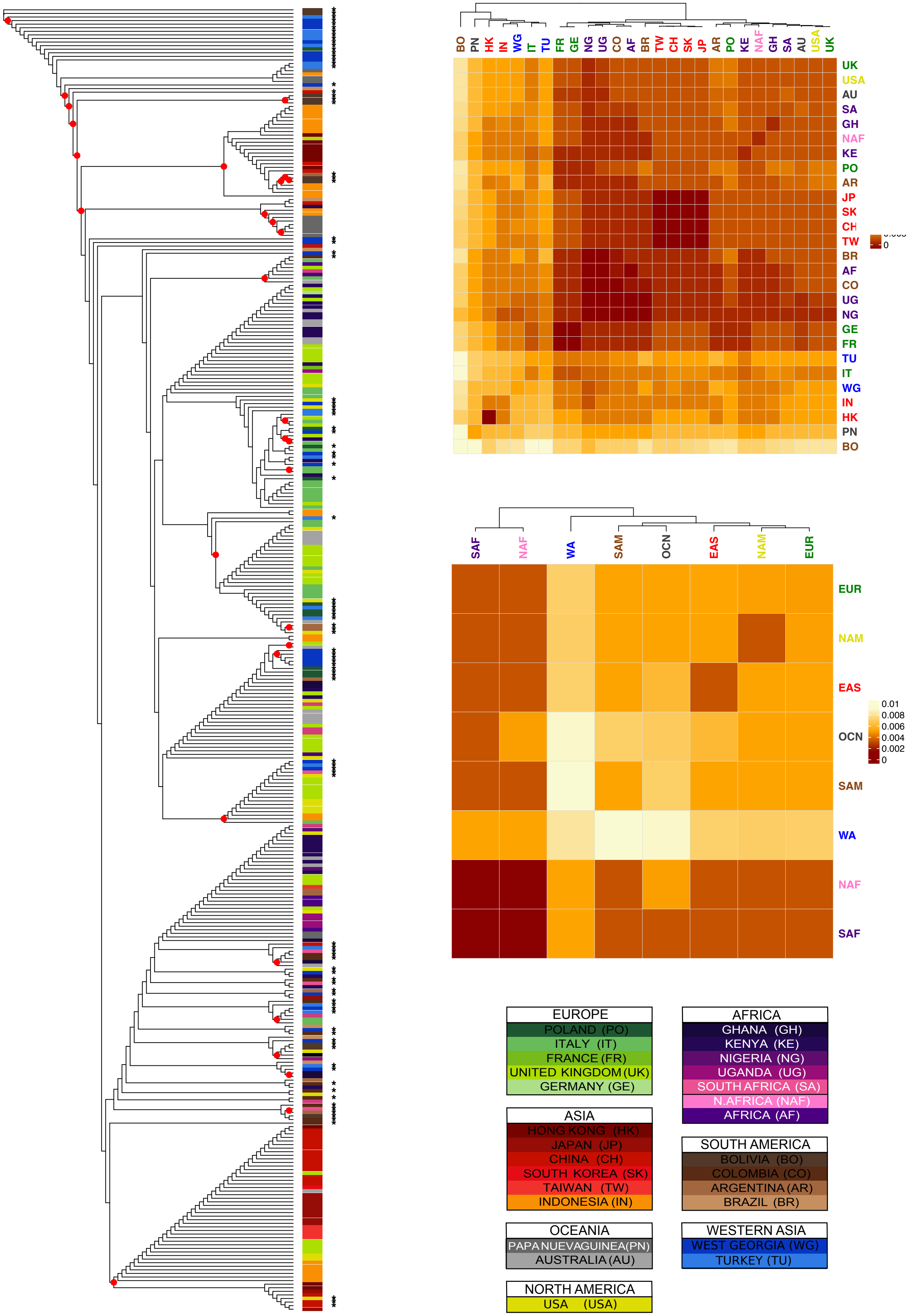

BO PN HK IN WG IT TU FR GE NG UG CO AF BR TW CH SK JP AR PO KE NAF GH SA AU USA UK
UK USA AU SA GH NAF KE PO AR JP SK CH TW BR AF CO UG NG GE FR TU IT WG IN HK PN BO
0
SAF NAF WA SAM OCN EAS NAM EUR
EUR NAM EAS OCN SAM WA NAF SAF
0.01
0.008
0.006
0.004
0.002
0
EUROPE
POLAND (PO)
ITALY (IT)
FRANCE (FR)
UNITED KINGDOM (UK)
GERMANY (GE)
AFRICA
GHANA (GH)
KENYA (KE)
NIGERIA (NG)
UGANDA (UG)
SOUTH AFRICA (SA)
N.AFRICA (NAF)
AFRICA (AF)
ASIA
HONG KONG (HK)
JAPAN (JP)
CHINA (CH)
SOUTH KOREA (SK)
TAIWAN (TW)
INDONESIA (IN)
SOUTH AMERICA
BOLIVIA (BO)
COLOMBIA (CO)
ARGENTINA (AR)
BRAZIL (BR)
OCEANIA
PAPA NUEVAGUINEA(PN)
AUSTRALIA (AU)
WESTERN ASIA
WEST GEORGIA (WG)
TURKEY (TU)
NORTH AMERICA
USA (USA)

BRLF1

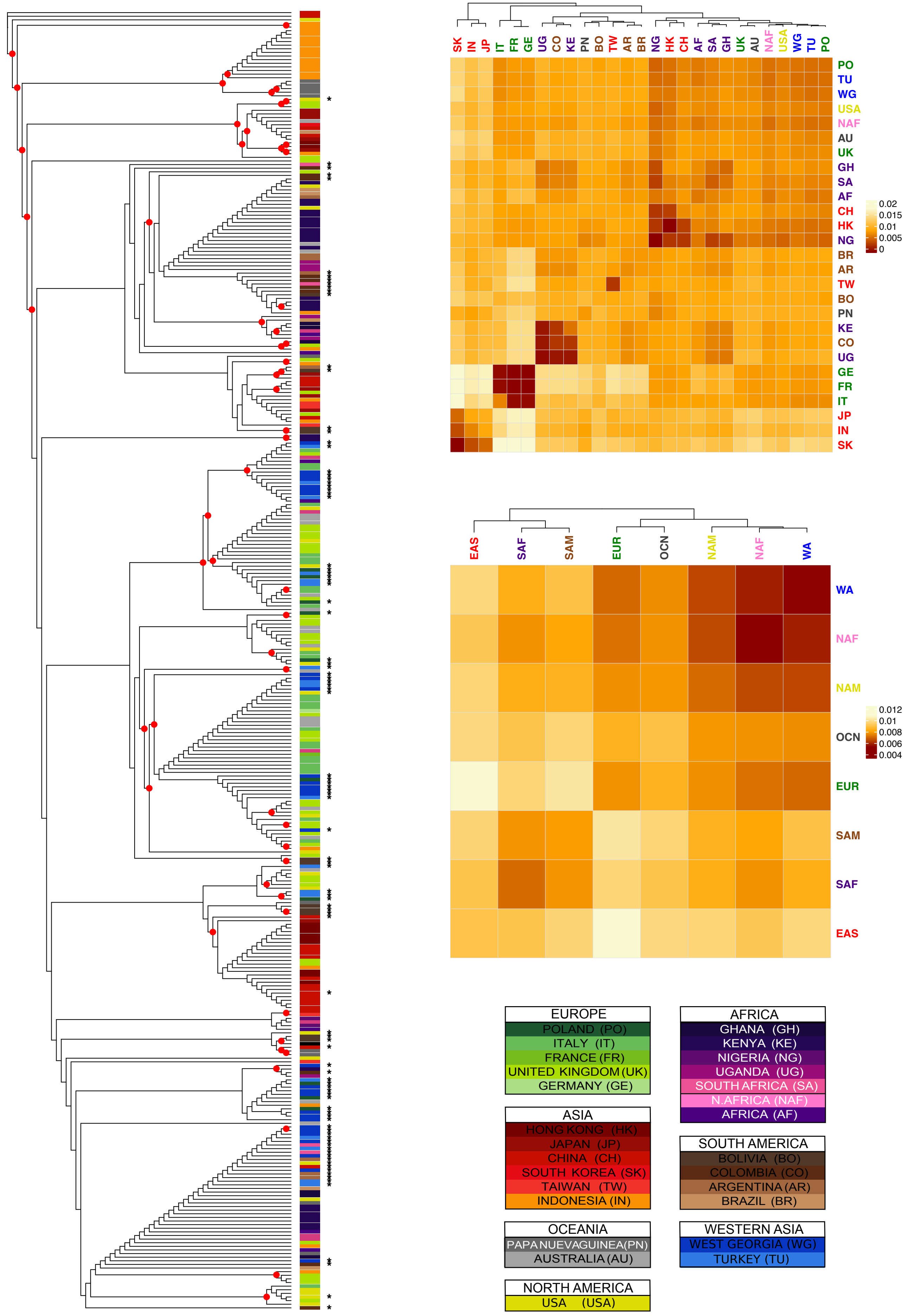

SK IN JP IT FR GE UG CO KE PN BO TW AR BR NG HK CH AF SA GH UK AU NAF USA WG TU PO
PO TU WG USA NAF AU UK GH SA AF CH HK NG BR AR TW BO PN KE CO UG GE FR IT JP IN SK
0.02
0.015
0.01
0.005
0
EAS SAF SAM EUR OCN NAM NAF WA
WA NAF NAM OCN EUR SAM SAF EAS
0.012
0.01
0.008
0.006
0.004
EUROPE
POLAND (PO)
ITALY (IT)
FRANCE (FR)
UNITED KINGDOM (UK)
GERMANY (GE)
AFRICA
GHANA (GH)
KENYA (KE)
NIGERIA (NG)
UGANDA (UG)
SOUTH AFRICA (SA)
N.AFRICA (NAF)
AFRICA (AF)
ASIA
HONG KONG (HK)
JAPAN (JP)
CHINA (CH)
SOUTH KOREA (SK)
TAIWAN (TW)
INDONESIA (IN)
SOUTH AMERICA
BOLIVIA (BO)
COLOMBIA (CO)
ARGENTINA (AR)
BRAZIL (BR)
OCEANIA
PAPA NUEVAGUINEA (PN)
AUSTRALIA (AU)
WESTERN ASIA
WEST GEORGIA (WG)
TURKEY (TU)
NORTH AMERICA
USA (USA)

BZLF1

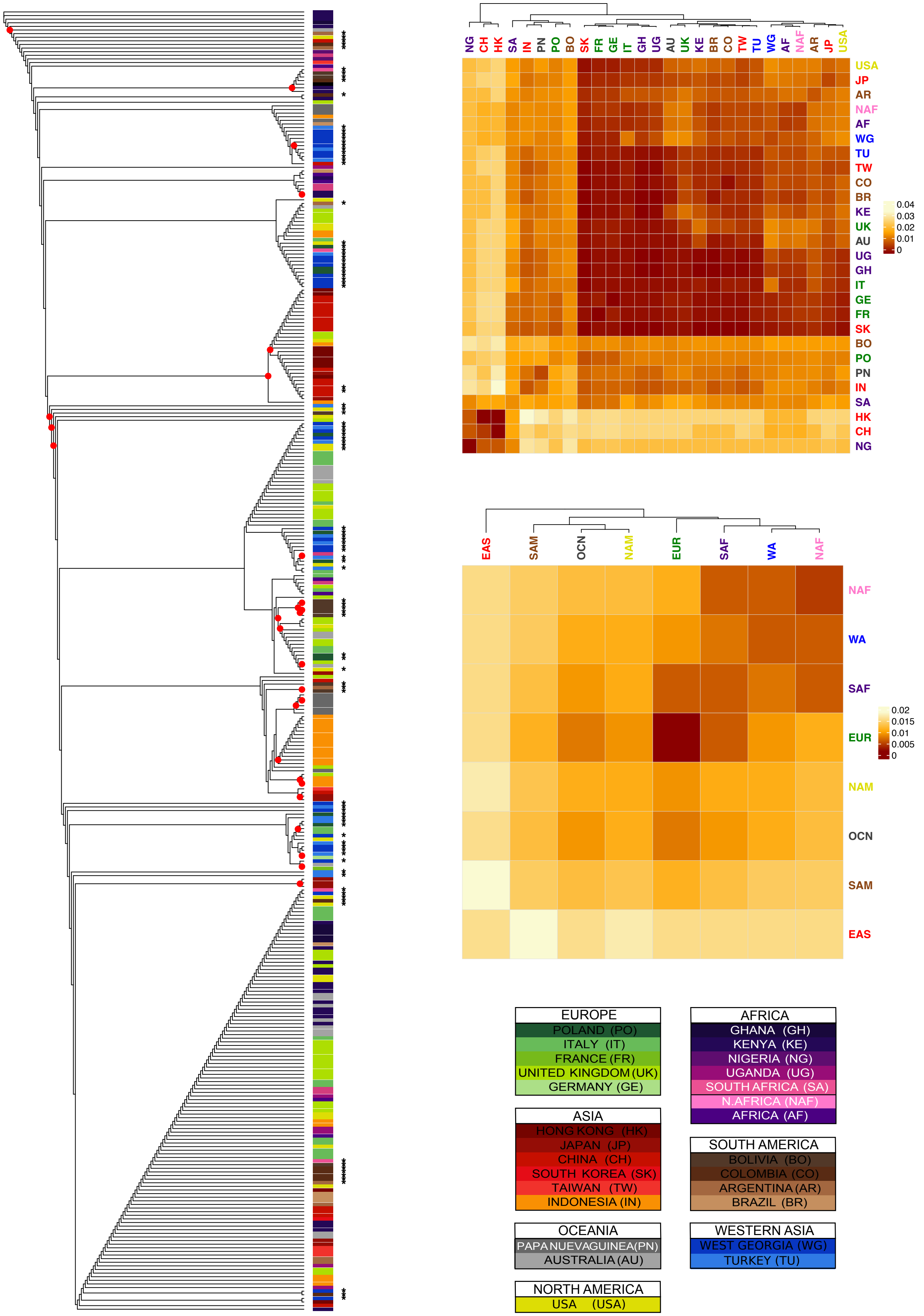

NG CH HK SA IN PN PO BO SK FR GE IT GH UG AU UK KE BR CO TW TU WG AF NAF AR JP USA
USA JP AR NAF AF WG TU TW CO BR KE UK AU UG GH IT GE FR SK BO PO PN IN SA HK CH NG
0.04
0.03
0.02
0.01
0
EAS SAM OCN NAM EUR SAF WA NAF
NAF WA SAF EUR NAM OCN SAM EAS
0.02
0.015
0.01
0.005
0
EUROPE
POLAND (PO)
ITALY (IT)
FRANCE (FR)
UNITED KINGDOM (UK)
GERMANY (GE)
AFRICA
GHANA (GH)
KENYA (KE)
NIGERIA (NG)
UGANDA (UG)
SOUTH AFRICA (SA)
N.AFRICA (NAF)
AFRICA (AF)
ASIA
HONG KONG (HK)
JAPAN (JP)
CHINA (CH)
SOUTH KOREA (SK)
TAIWAN (TW)
INDONESIA (IN)
SOUTH AMERICA
BOLIVIA (BO)
COLOMBIA (CO)
ARGENTINA (AR)
BRAZIL (BR)
OCEANIA
PAPA NUEVAGUINEA(PN)
AUSTRALIA (AU)
WESTERN ASIA
WEST GEORGIA (WG)
TURKEY (TU)
NORTH AMERICA
USA (USA)

EBER-1

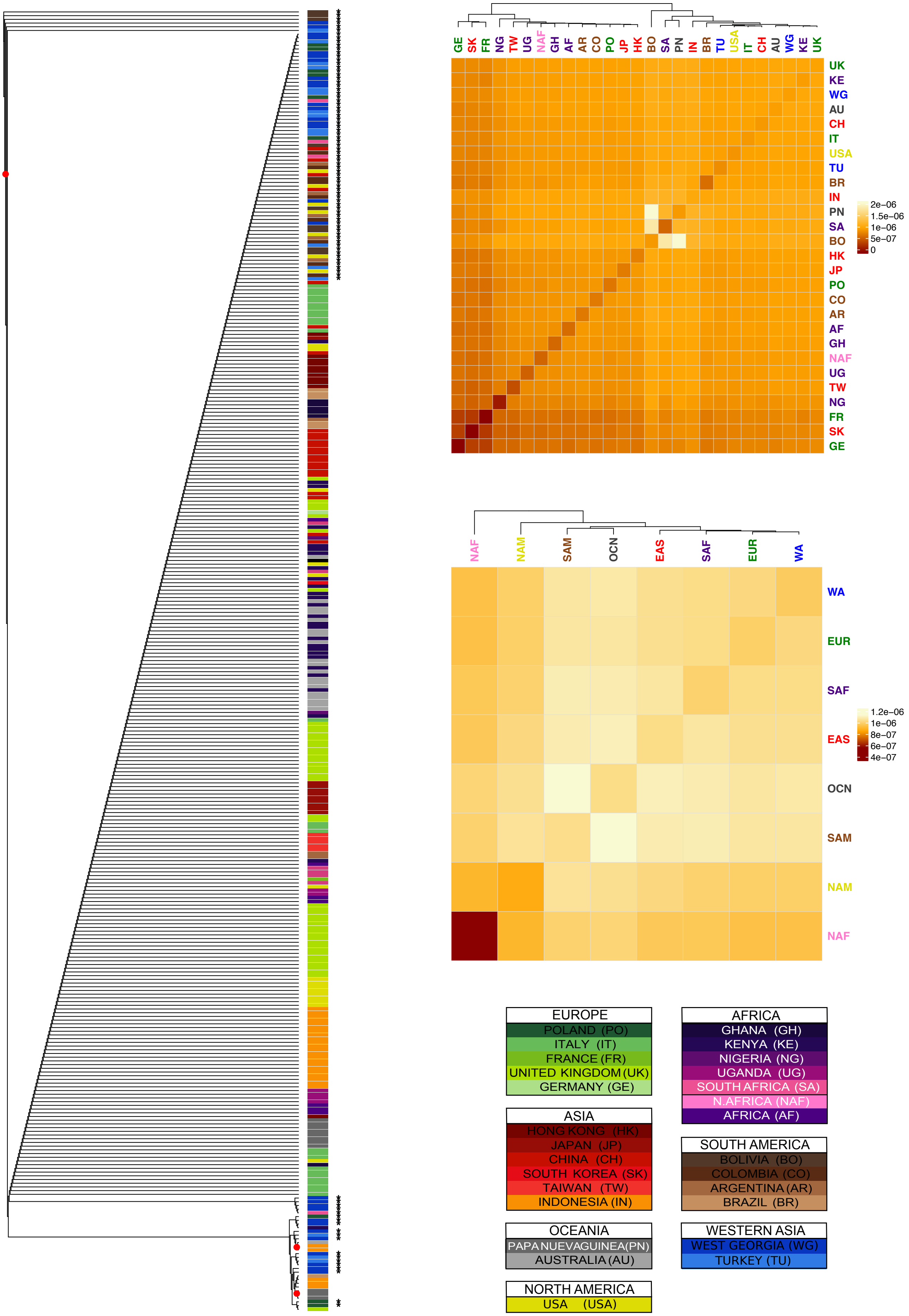
GE SK FR NG TW UG NAF GH AF AR CO PO JP HK BO SA PN IN BR TU USA IT CH AU WG KE UK
UK KE WG AU CH IT USA TU BR IN PN SA BO HK JP PO CO AR AF GH NAF UG TW NG FR SK GE
2e−06
1.5e−06
1e−06
5e−07
0
NAF NAM SAM OCN EAS SAF EUR WA
WA EUR SAF EAS OCN SAM NAM NAF
1.2e−06
1e−06
8e−07
6e−07
4e−07
EUROPE
POLAND (PO)
ITALY (IT)
FRANCE (FR)
UNITED KINGDOM (UK)
GERMANY (GE)
AFRICA
GHANA (GH)
KENYA (KE)
NIGERIA (NG)
UGANDA (UG)
SOUTH AFRICA (SA)
N.AFRICA (NAF)
AFRICA (AF)
ASIA
HONG KONG (HK)
JAPAN (JP)
CHINA (CH)
SOUTH KOREA (SK)
TAIWAN (TW)
INDONESIA (IN)
SOUTH AMERICA
BOLIVIA (BO)
COLOMBIA (CO)
ARGENTINA (AR)
BRAZIL (BR)
OCEANIA
PAPA NUEVAGUINEA(PN)
AUSTRALIA (AU)
WESTERN ASIA
WEST GEORGIA (WG)
TURKEY (TU)
NORTH AMERICA
USA (USA)

EBER-2

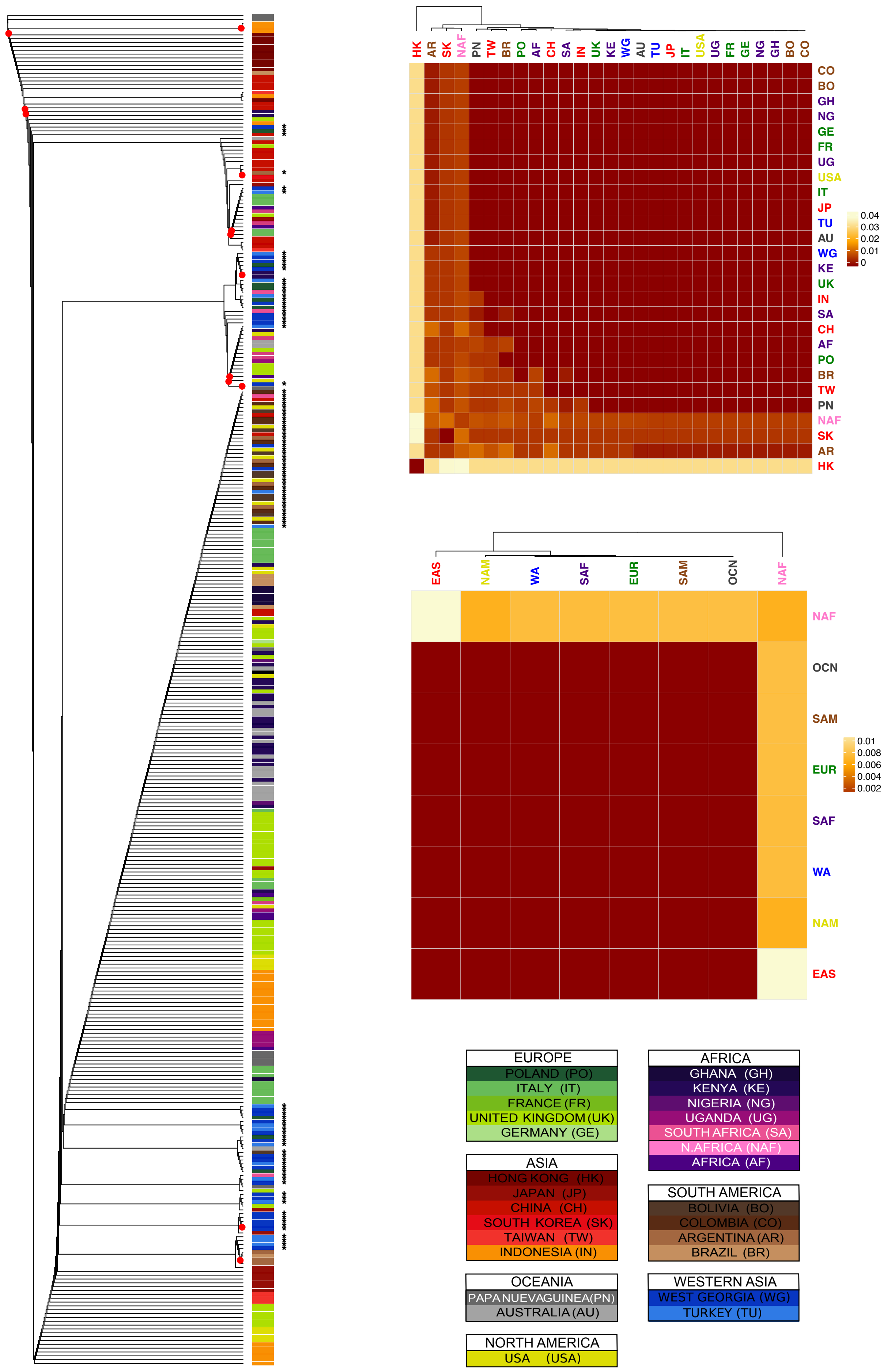

HK AR SK NAF PN TW BR PO AF CH SA IN UK KE WG AU TU JP IT USA UG FR GE NG GH BO CO
CO BO GH NG GE FR UG USA IT JP TU AU WG KE UK IN SA CH AF PO BR TW PN NAF SK AR HK
0.04 0.03 0.02 0.01 0
EAS NAM WA SAF EUR SAM OCN NAF
NAF OCN SAM EUR SAF WA NAM EAS
0.01 0.008 0.006 0.004 0.002
EUROPE
POLAND (PO)
ITALY (IT)
FRANCE (FR)
UNITED KINGDOM (UK)
GERMANY (GE)
AFRICA
GHANA (GH)
KENYA (KE)
NIGERIA (NG)
UGANDA (UG)
SOUTH AFRICA (SA)
N.AFRICA (NAF)
AFRICA (AF)
ASIA
HONG KONG (HK)
JAPAN (JP)
CHINA (CH)
SOUTH KOREA (SK)
TAIWAN (TW)
INDONESIA (IN)
SOUTH AMERICA
BOLIVIA (BO)
COLOMBIA (CO)
ARGENTINA (AR)
BRAZIL (BR)
OCEANIA
PAPA NUEVAGUINEA(PN)
AUSTRALIA (AU)
WESTERN ASIA
WEST GEORGIA (WG)
TURKEY (TU)
NORTH AMERICA
USA (USA)

EBNA-1

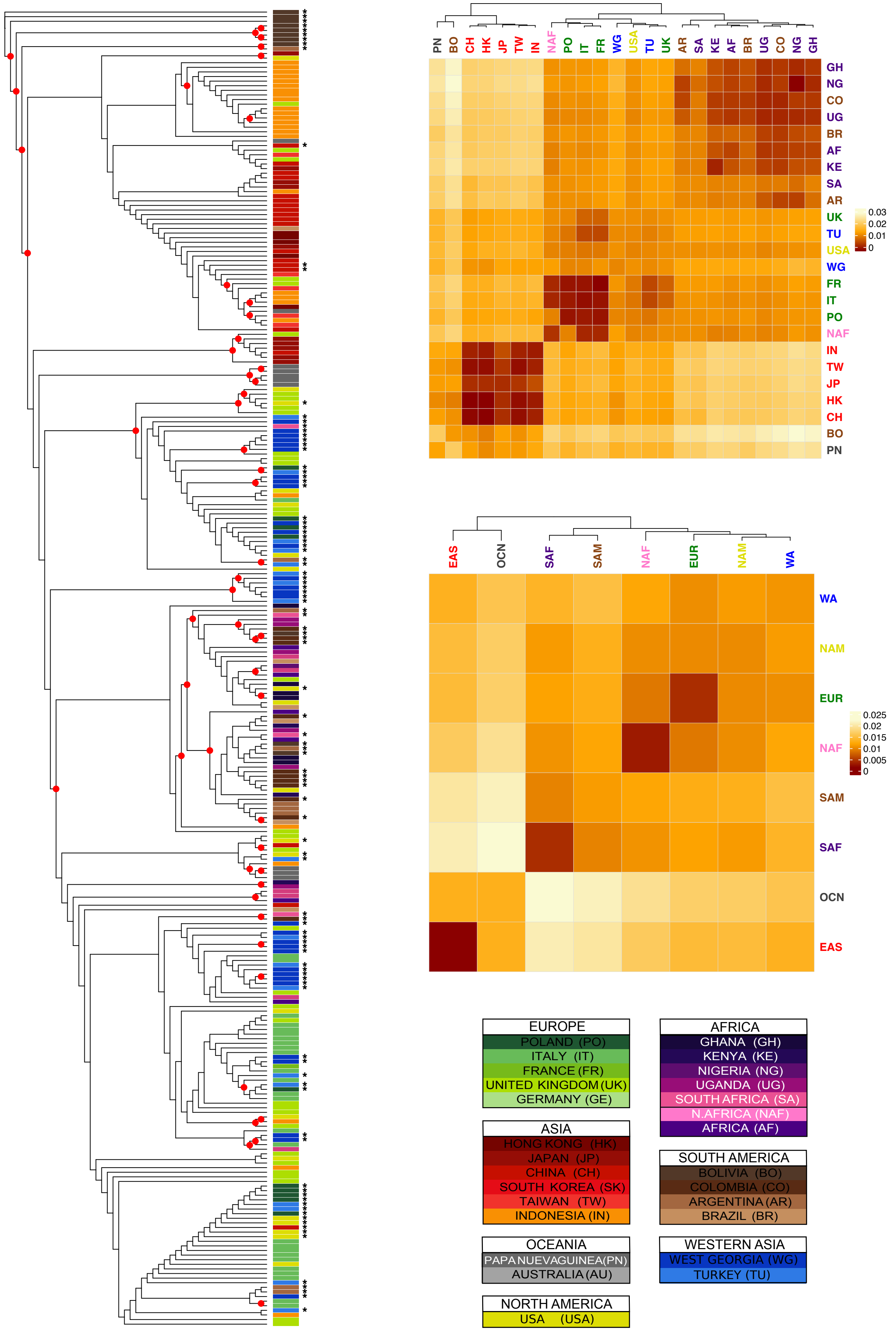

PN BO CH HK JP TW IN NAF PO IT FR WG USA TU UK AR SA KE AF BR UG CO NG GH
GH NG CO UG BR AF KE SA AR UK TU USA WG FR IT PO NAF IN TW JP HK CH BO PN
0.03
0.02
0.01
0
EAS OCN SAF SAM NAF EUR NAM WA
WA NAM EUR NAF SAM SAF OCN EAS
0.025
0.02
0.015
0.01
0.005
0
EUROPE
POLAND (PO)
ITALY (IT)
FRANCE (FR)
UNITED KINGDOM (UK)
GERMANY (GE)
AFRICA
GHANA (GH)
KENYA (KE)
NIGERIA (NG)
UGANDA (UG)
SOUTH AFRICA (SA)
N.AFRICA (NAF)
AFRICA (AF)
ASIA
HONG KONG (HK)
JAPAN (JP)
CHINA (CH)
SOUTH KOREA (SK)
TAIWAN (TW)
INDONESIA (IN)
SOUTH AMERICA
BOLIVIA (BO)
COLOMBIA (CO)
ARGENTINA (AR)
BRAZIL (BR)
OCEANIA
PAPA NUEVAGUINEA(PN)
AUSTRALIA (AU)
WESTERN ASIA
WEST GEORGIA (WG)
TURKEY (TU)
NORTH AMERICA
USA (USA)

EBNA-2

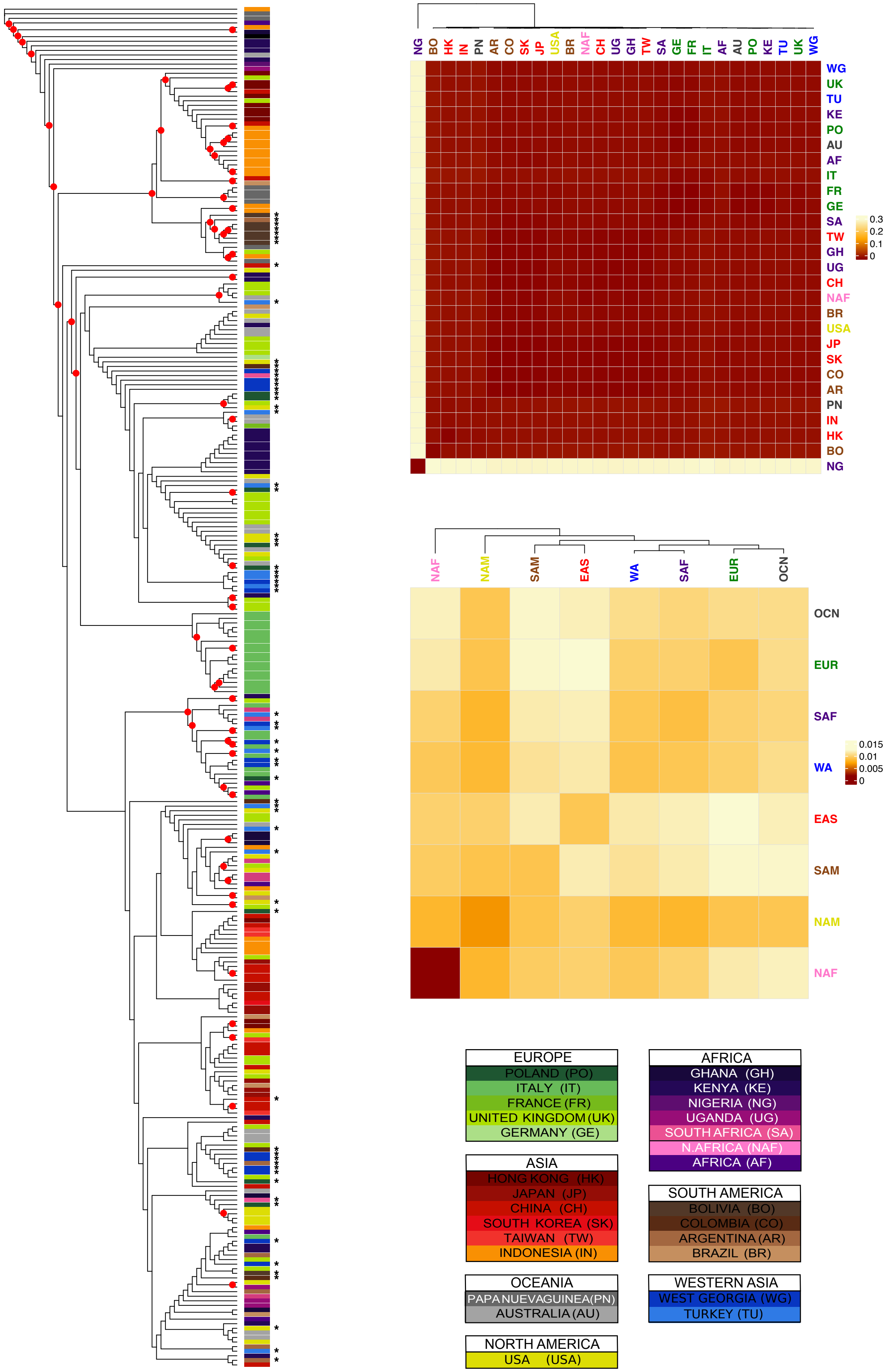

NG BO HK IN PN AR CO SK JP USA BR NAF CH UG GH TW SA GE FR IT AF AU PO KE TU UK WG
WG UK TU KE PO AU AF IT FR GE SA TW GH UG CH NAF BR USA JP SK CO AR PN IN HK BO NG
0.3 0.2 0.1 0
NAF NAM SAM EAS WA SAF EUR OCN
OCN EUR SAF WA EAS SAM NAM NAF
0.015 0.01 0.005 0
EUROPE
POLAND (PO)
ITALY (IT)
FRANCE (FR)
UNITED KINGDOM (UK)
GERMANY (GE)
AFRICA
GHANA (GH)
KENYA (KE)
NIGERIA (NG)
UGANDA (UG)
SOUTH AFRICA (SA)
N.AFRICA (NAF)
AFRICA (AF)
ASIA
HONG KONG (HK)
JAPAN (JP)
CHINA (CH)
SOUTH KOREA (SK)
TAIWAN (TW)
INDONESIA (IN)
SOUTH AMERICA
BOLIVIA (BO)
COLOMBIA (CO)
ARGENTINA (AR)
BRAZIL (BR)
OCEANIA
PAPA NUEVAGUINEA(PN)
AUSTRALIA (AU)
WESTERN ASIA
WEST GEORGIA (WG)
TURKEY (TU)
NORTH AMERICA
USA (USA)

EBNA-2 type1

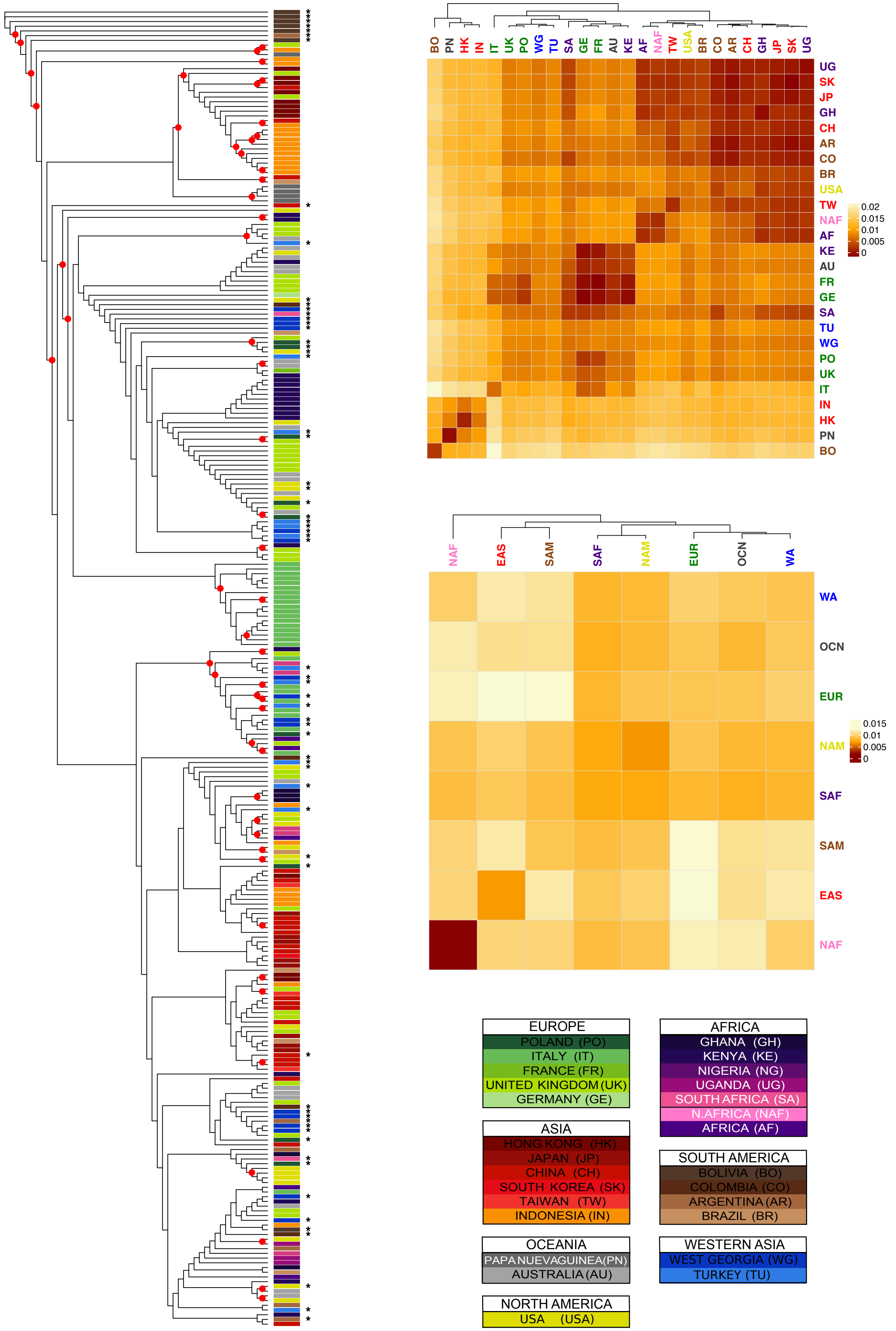
BO PN HK IN IT UK PO WG TU SA GE FR AU KE AF NAF TW USA BR CO AR CH GH JP SK UG
UG SK JP GH CH AR CO BR USA TW NAF AF KE AU FR GE SA TU WG PO UK IT IN HK PN BO
0.02
0.015
0.01
0.005
0
NAF EAS SAM SAF NAM EUR OCN WA
WA OCN EUR NAM SAF SAM EAS NAF
0.015
0.01
0.005
0
EUROPE
POLAND (PO)
ITALY (IT)
FRANCE (FR)
UNITED KINGDOM (UK)
GERMANY (GE)
AFRICA
GHANA (GH)
KENYA (KE)
NIGERIA (NG)
UGANDA (UG)
SOUTH AFRICA (SA)
N.AFRICA (NAF)
AFRICA (AF)
ASIA
HONG KONG (HK)
JAPAN (JP)
CHINA (CH)
SOUTH KOREA (SK)
TAIWAN (TW)
INDONESIA (IN)
SOUTH AMERICA
BOLIVIA (BO)
COLOMBIA (CO)
ARGENTINA (AR)
BRAZIL (BR)
OCEANIA
PAPA NUEVAGUINEA(PN)
AUSTRALIA (AU)
WESTERN ASIA
WEST GEORGIA (WG)
TURKEY (TU)
NORTH AMERICA
USA (USA)

EBNA-3A

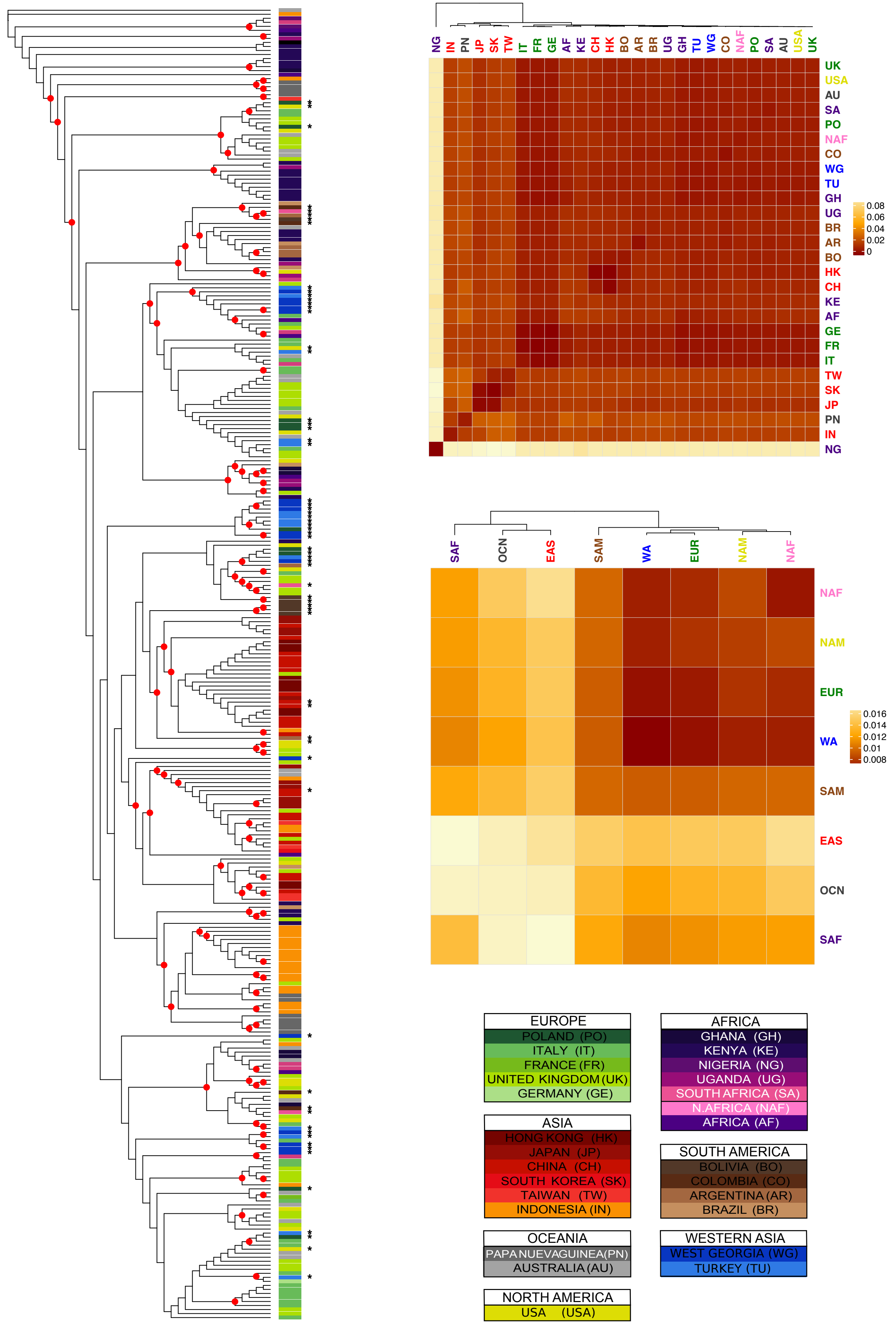

NG IN PN JP SK TW IT FR GE AF KE CH HK BO AR BR UG GH TU WG CO NAF PO SA AU USA UK
UK USA AU SA PO NAF CO WG TU GH UG BR AR BO HK CH KE AF GE FR IT TW SK JP PN IN NG
0.08 0.06 0.04 0.02 0
SAF OCN EAS SAM WA EUR NAM NAF
NAF NAM EUR WA SAM EAS OCN SAF
0.016 0.014 0.012 0.01 0.008
EUROPE
POLAND (PO)
ITALY (IT)
FRANCE (FR)
UNITED KINGDOM (UK)
GERMANY (GE)
AFRICA
GHANA (GH)
KENYA (KE)
NIGERIA (NG)
UGANDA (UG)
SOUTH AFRICA (SA)
N.AFRICA (NAF)
AFRICA (AF)
ASIA
HONG KONG (HK)
JAPAN (JP)
CHINA (CH)
SOUTH KOREA (SK)
TAIWAN (TW)
INDONESIA (IN)
SOUTH AMERICA
BOLIVIA (BO)
COLOMBIA (CO)
ARGENTINA (AR)
BRAZIL (BR)
OCEANIA
PAPA NUEVAGUINEA(PN)
AUSTRALIA (AU)
WESTERN ASIA
WEST GEORGIA (WG)
TURKEY (TU)
NORTH AMERICA
USA (USA)

EBNA-3A type1

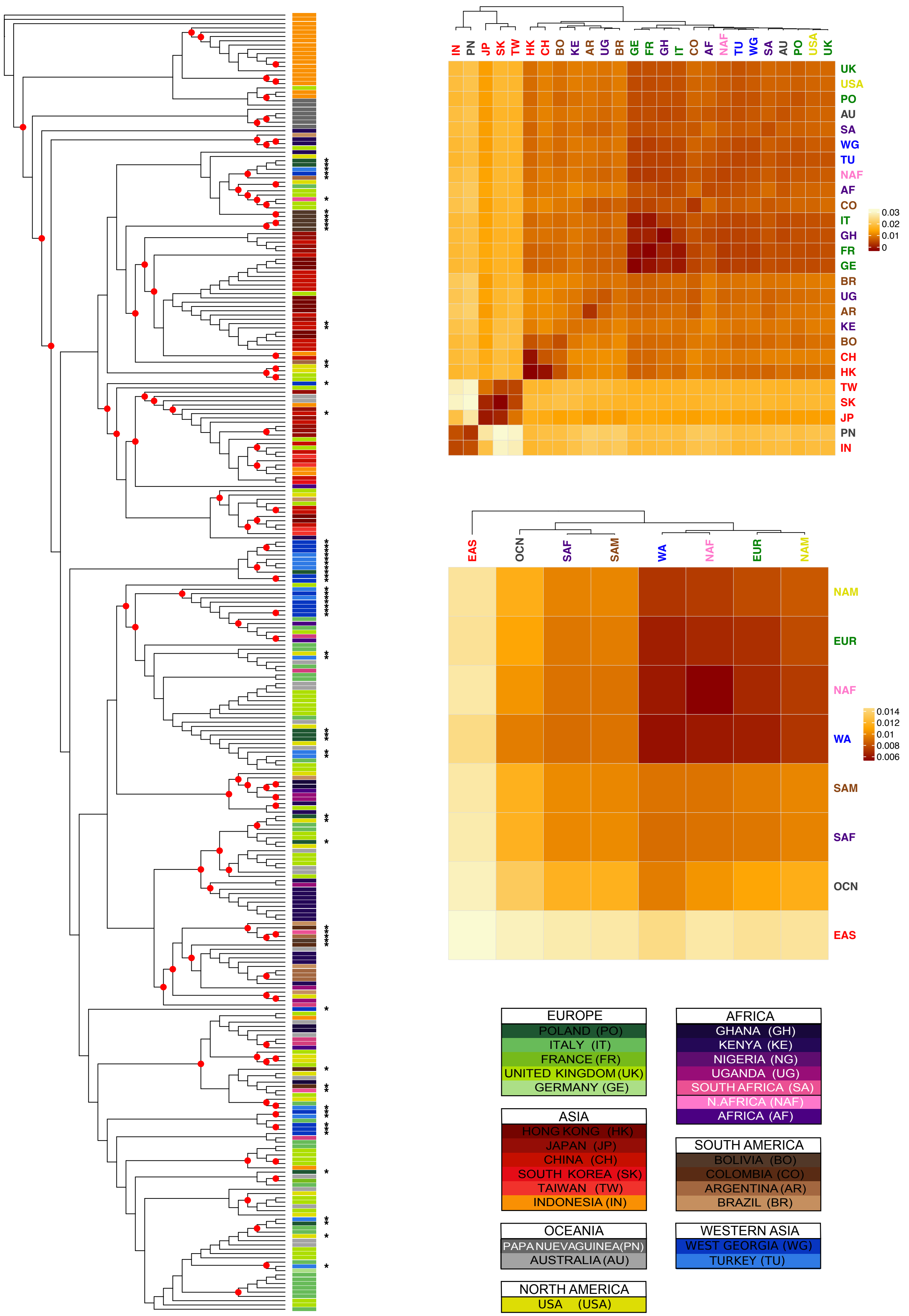

EBNA-3B

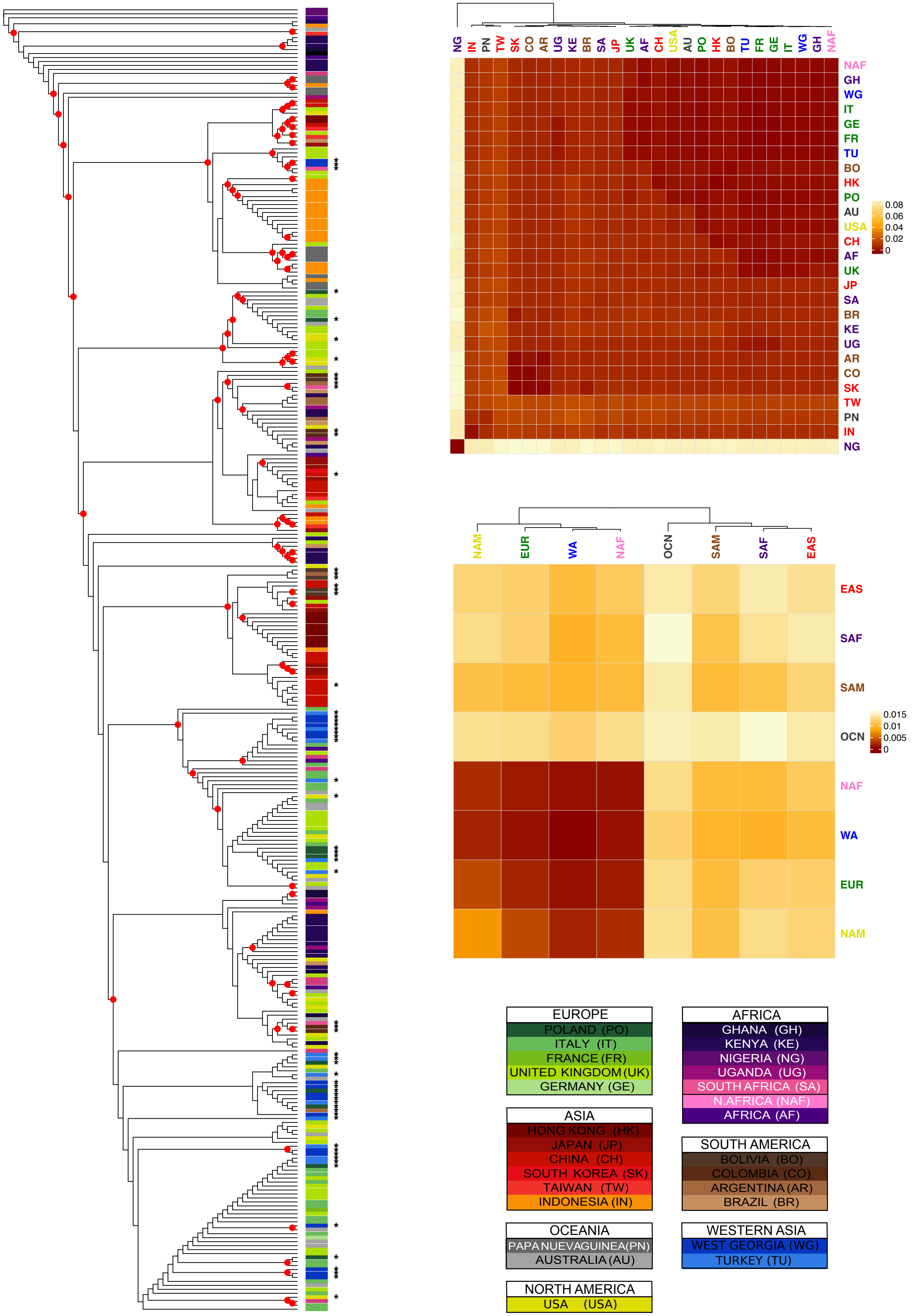

NG IN PN TW SK CO AR UG KE BR SA JP UK AF CH USA AU PO HK BO TU FR GE IT WG GH NAF
NAF GH WG IT GE FR TU BO HK PO AU USA CH AF UK JP SA BR KE UG AR CO SK TW PN IN NG
0.08
0.06
0.04
0.02
0
NAM EUR WA NAF OCN SAM SAF EAS
EAS SAF SAM OCN NAF WA EUR NAM
0.015
0.01
0.005
0
EUROPE
POLAND (PO)
ITALY (IT)
FRANCE (FR)
UNITED KINGDOM (UK)
GERMANY (GE)
ASIA
HONG KONG (HK)
JAPAN (JP)
CHINA (CH)
SOUTH KOREA (SK)
TAIWAN (TW)
INDONESIA (IN)
OCEANIA
PAPA NUEVAGUINEA(PN)
AUSTRALIA (AU)
NORTH AMERICA
USA (USA)
AFRICA
GHANA (GH)
KENYA (KE)
NIGERIA (NG)
UGANDA (UG)
SOUTH AFRICA (SA)
N.AFRICA (NAF)
AFRICA (AF)
SOUTH AMERICA
BOLIVIA (BO)
COLOMBIA (CO)
ARGENTINA (AR)
BRAZIL (BR)
WESTERN ASIA
WEST GEORGIA (WG)
TURKEY (TU)

EBNA-3B type1

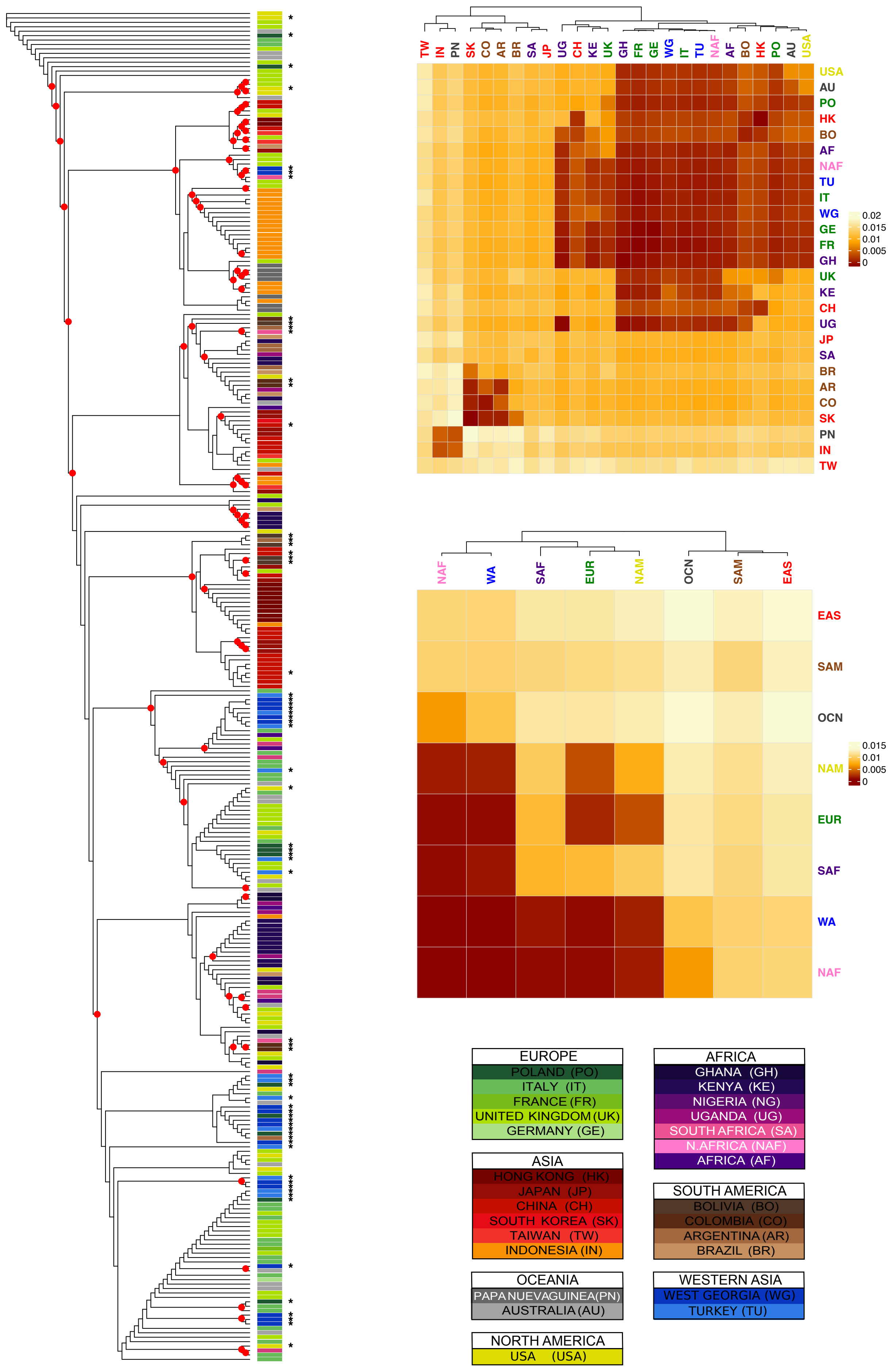

EBNA-3C

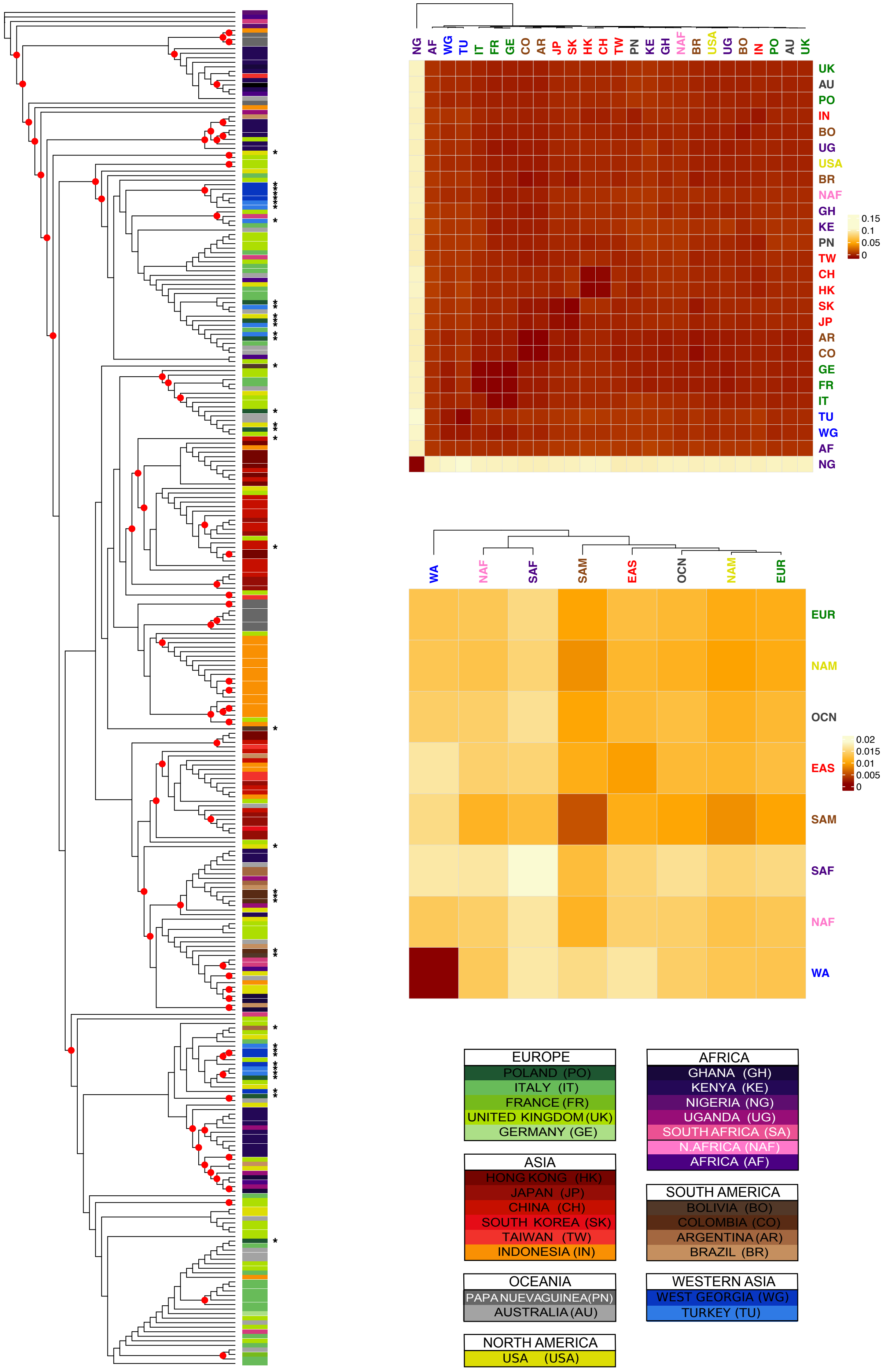

NG AF WG TU IT FR GE CO AR JP SK HK CH TW PN KE GH NAF BR USA UG BO IN PO AU UK
UK AU PO IN BO UG USA BR NAF GH KE PN TW CH HK SK JP AR CO GE FR IT TU WG AF NG
0.15 0.1 0.05 0
WA NAF SAF SAM EAS OCN NAM EUR
EUR NAM OCN EAS SAM SAF NAF WA
0.02 0.015 0.01 0.005 0
EUROPE
POLAND (PO)
ITALY (IT)
FRANCE (FR)
UNITED KINGDOM (UK)
GERMANY (GE)
AFRICA
GHANA (GH)
KENYA (KE)
NIGERIA (NG)
UGANDA (UG)
SOUTH AFRICA (SA)
N.AFRICA (NAF)
AFRICA (AF)
ASIA
HONG KONG (HK)
JAPAN (JP)
CHINA (CH)
SOUTH KOREA (SK)
TAIWAN (TW)
INDONESIA (IN)
SOUTH AMERICA
BOLIVIA (BO)
COLOMBIA (CO)
ARGENTINA (AR)
BRAZIL (BR)
OCEANIA
PAPA NUEVAGUINEA(PN)
AUSTRALIA (AU)
WESTERN ASIA
WEST GEORGIA (WG)
TURKEY (TU)
NORTH AMERICA
USA (USA)

EBNA-3C type1

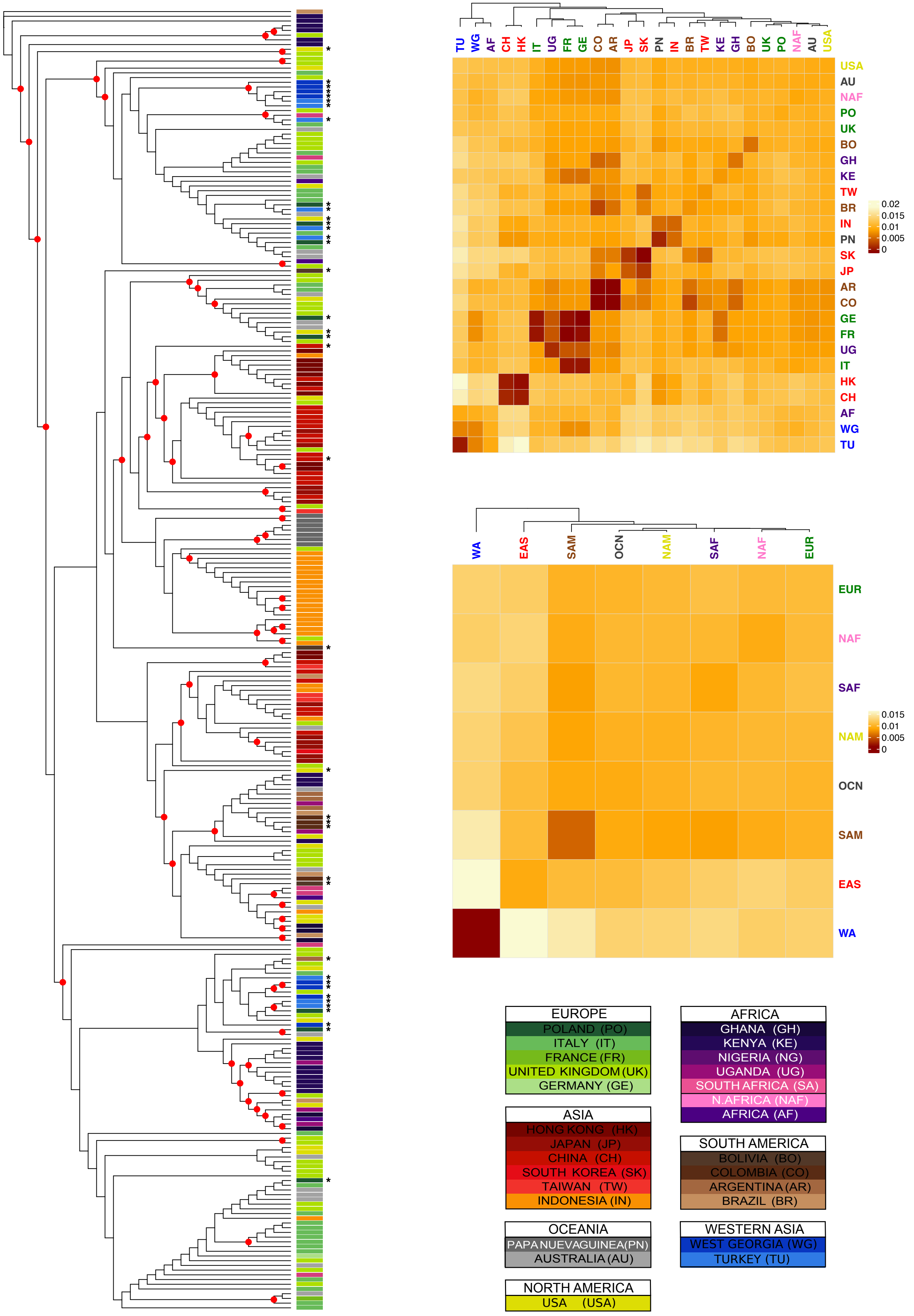

LMP-2A

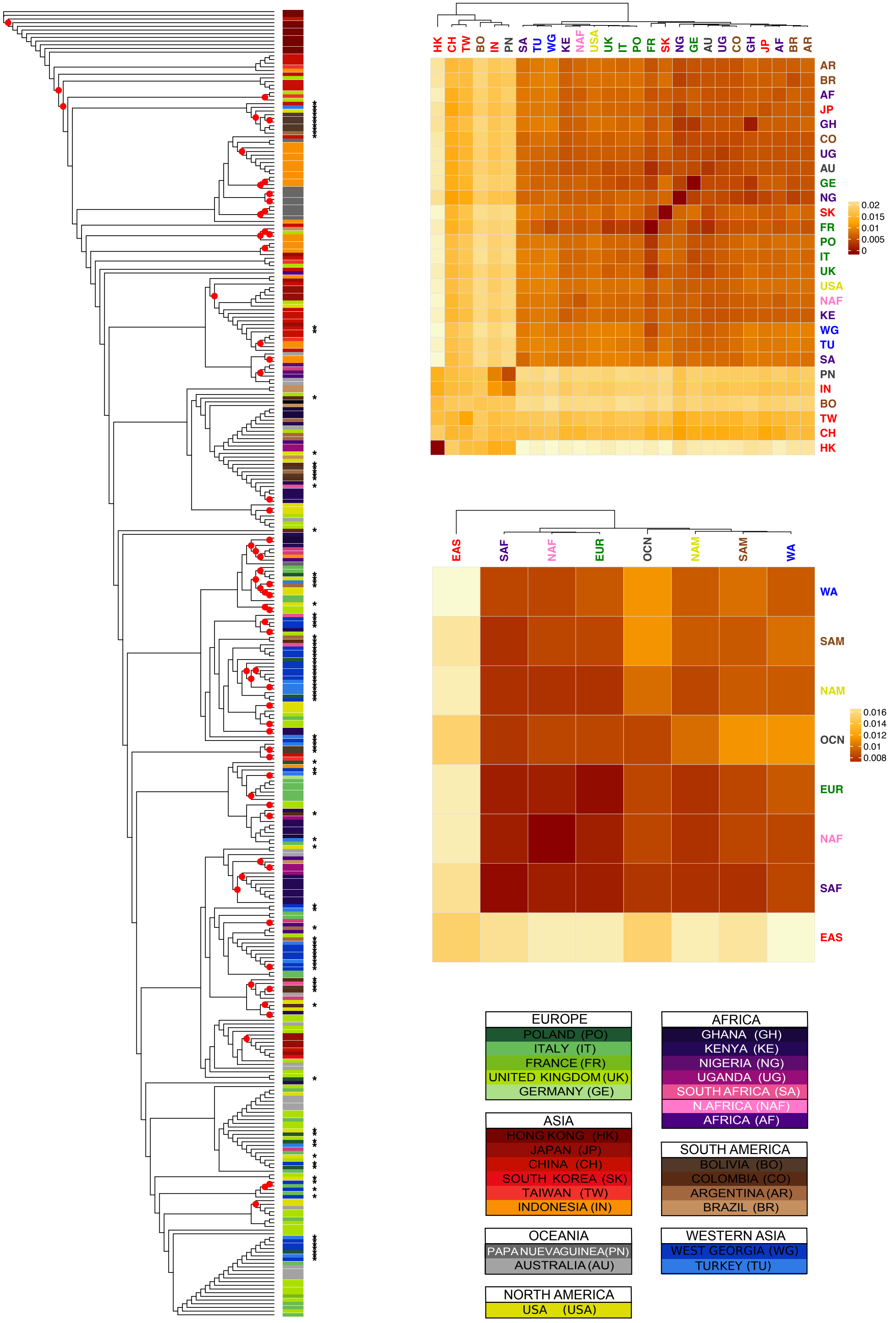

HK CH TW BO IN PN SA TU WG KE NAF USA UK IT PO FR SK NG GE AU UG CO GH JP AF BR AR
AR BR AF JP GH CO UG AU GE NG SK FR PO IT UK USA NAF KE WG TU SA PN IN BO TW CH HK
0.02
0.015
0.01
0.005
0
EAS SAF NAF EUR OCN NAM SAM WA
WA SAM NAM OCN EUR NAF SAF EAS
0.016
0.014
0.012
0.01
0.008
EUROPE
POLAND (PO)
ITALY (IT)
FRANCE (FR)
UNITED KINGDOM (UK)
GERMANY (GE)
AFRICA
GHANA (GH)
KENYA (KE)
NIGERIA (NG)
UGANDA (UG)
SOUTH AFRICA (SA)
N.AFRICA (NAF)
AFRICA (AF)
ASIA
HONG KONG (HK)
JAPAN (JP)
CHINA (CH)
SOUTH KOREA (SK)
TAIWAN (TW)
INDONESIA (IN)
SOUTH AMERICA
BOLIVIA (BO)
COLOMBIA (CO)
ARGENTINA (AR)
BRAZIL (BR)
OCEANIA
PAPA NUEVAGUINEA(PN)
AUSTRALIA (AU)
WESTERN ASIA
WEST GEORGIA (WG)
TURKEY (TU)
NORTH AMERICA
USA (USA)
