# Supplementary material for: Expanding the Geographic Characterisation of Epstein–Barr Virus Variation through Gene-Based Approaches
Source: Microorganisms. 2020 Oct 29;8(11):1686. doi: 10.3390/microorganisms8111686 (PMC7692309; doi:10.3390/microorganisms8111686)
Supplement: Supplementary file 1 [file microorganisms-08-01686-s001.zip › Supplementary/Supplementary_Figure_6.pdf]

Supplementary Figure 6

Optimal grouping selection. Grouping statistics per gene. The dots represent the MCC value for each grouping; the black dots represent groupings for which the isolates inside valid clusters are above 50% of the total number of isolates for the gene, the red dots represent the ones that do not meet this criterion. The green line represents the number of valid clusters in each grouping (*i.e.* clusters formed by at least 3 isolates). The orange line represents the Clustering per Geography Score (CGS) given by the MCC value multiplied for the number of valid clusters. The grouping with the maximum point reached by the orange line where the isolates in valid clusters is more than 50% of the total (black dots), indicates the optimum grouping for the gene. A second grouping was chosen as the one with the maximum MCC score within those including at least 50% of the isolates in valid clusters (*i.e.* higher black dot).

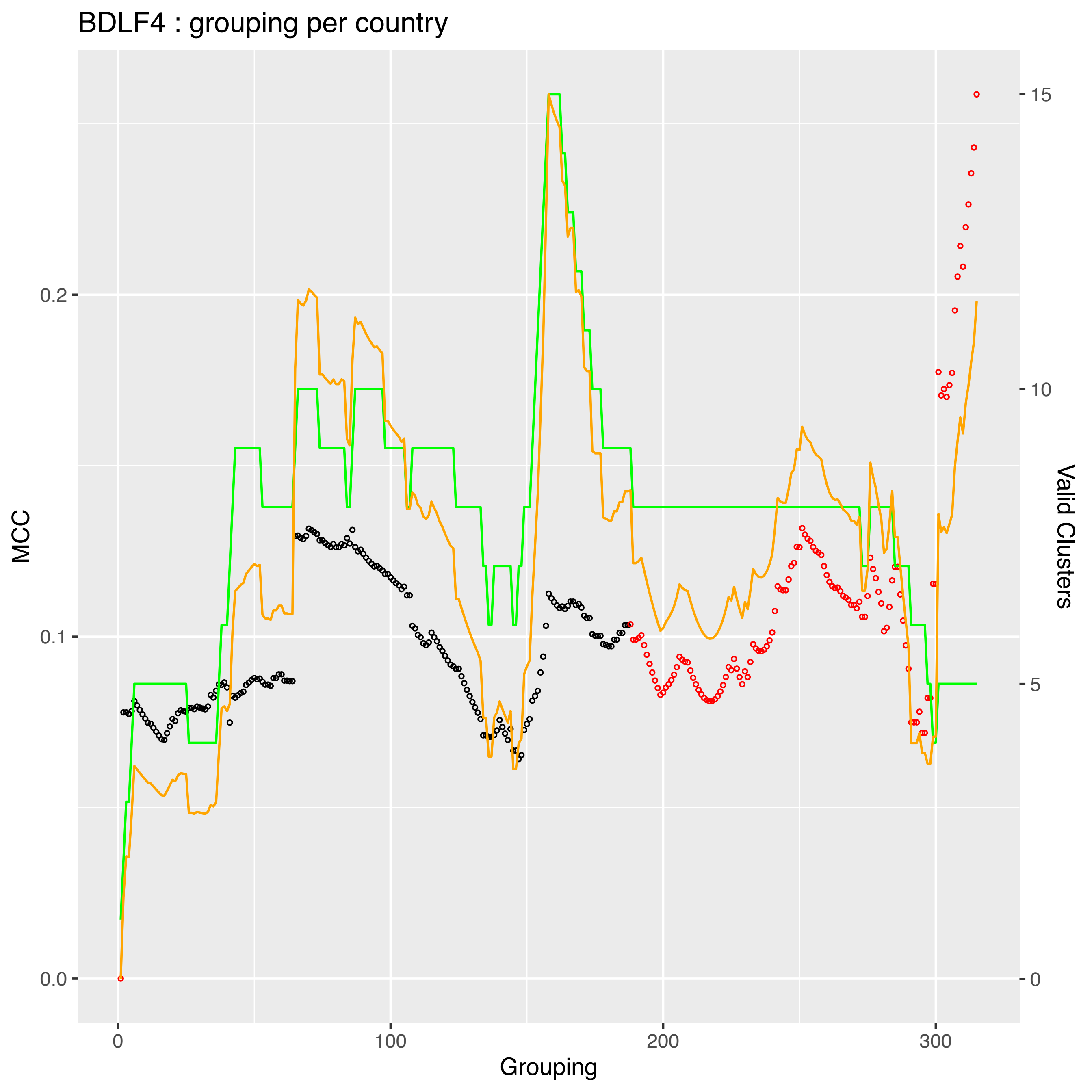

BDLF4 : grouping per country
MCC
0.0
0.1
0.2
Valid Clusters
0
5
10
15
Grouping
0
100
200
300

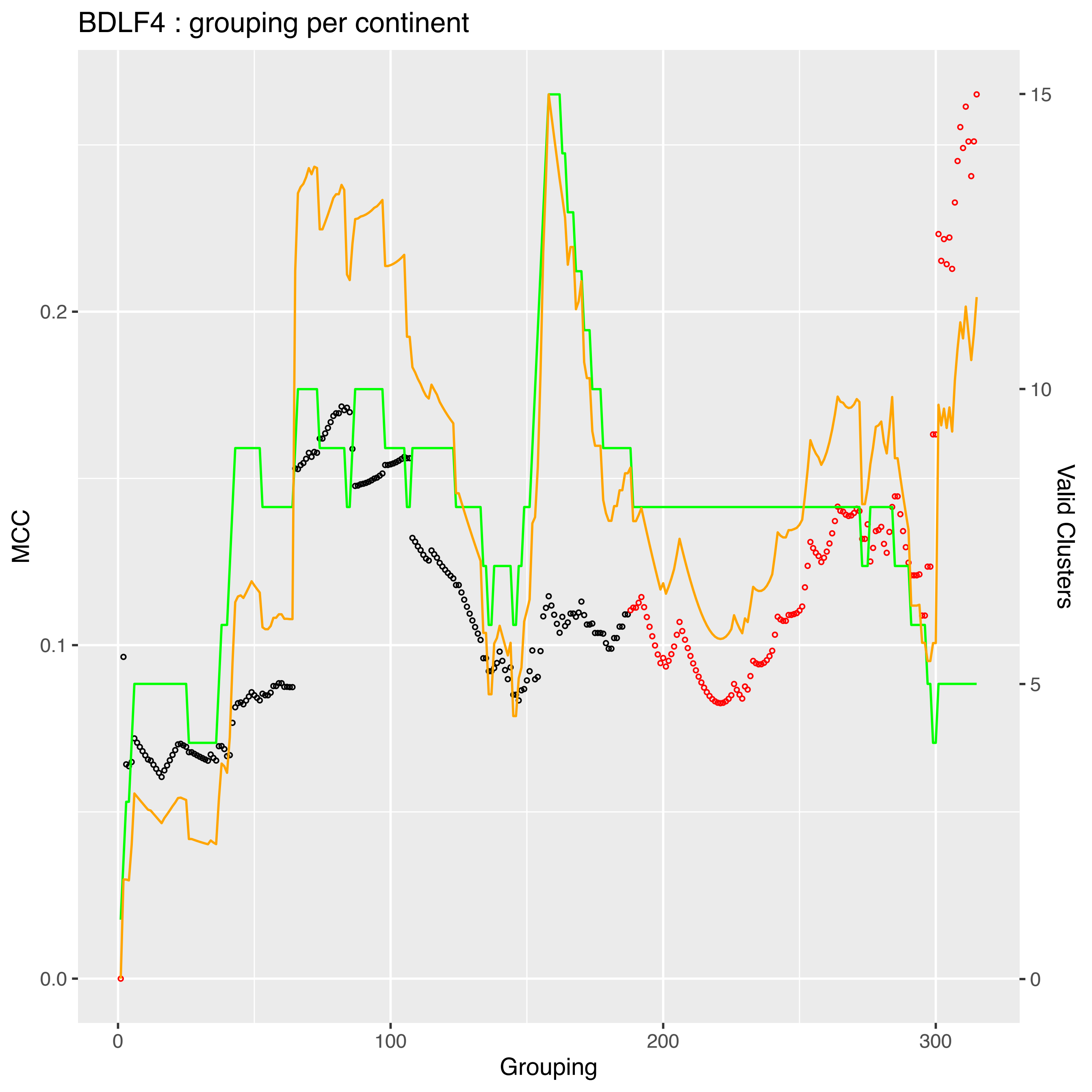
BDLF4 : grouping per continent
MCC
0.0
0.1
0.2
Valid Clusters
0
5
10
15
Grouping
0
100
200
300

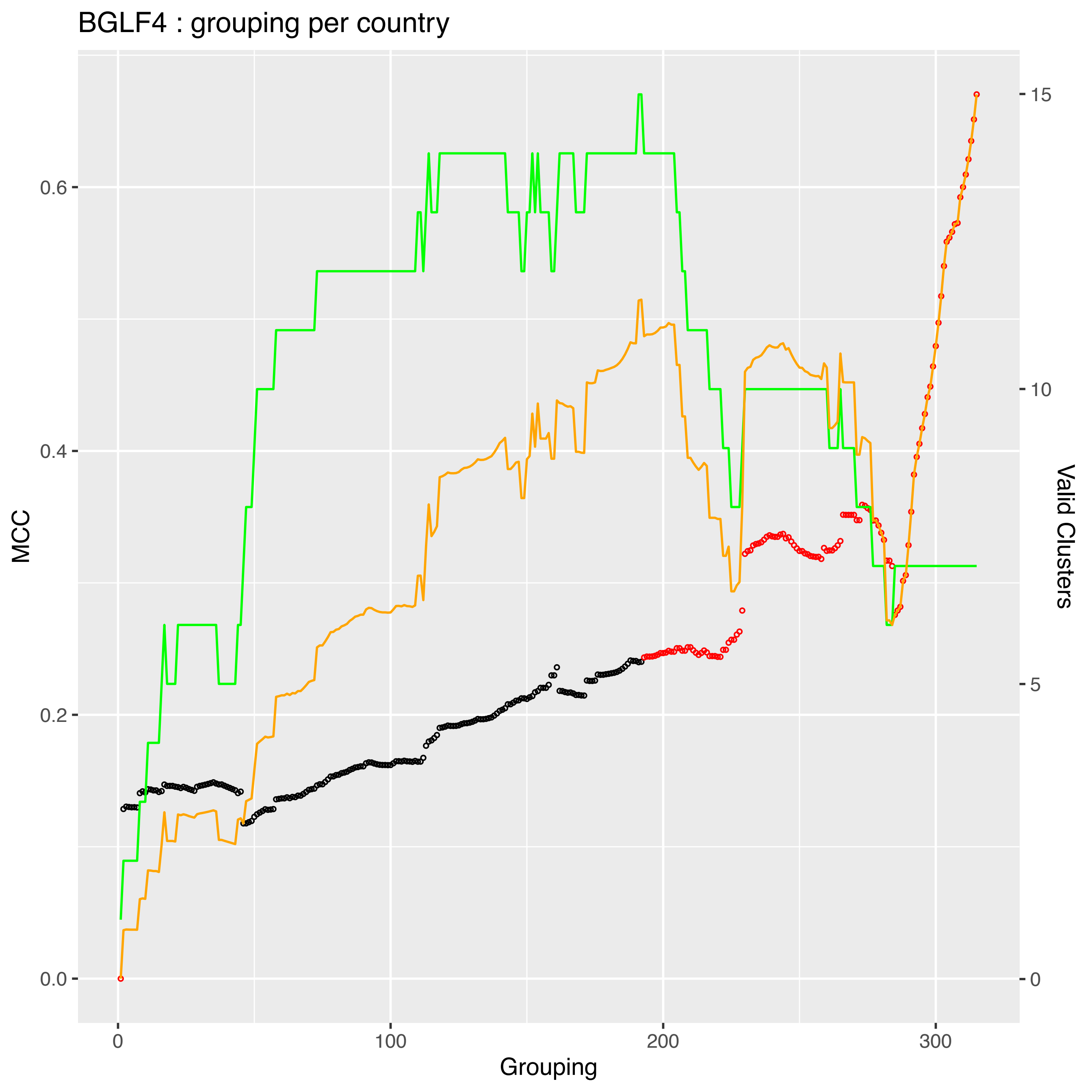

BGLF4 : grouping per country
MCC
0.0
0.2
0.4
0.6
Valid Clusters
0
5
10
15
Grouping
0
100
200
300

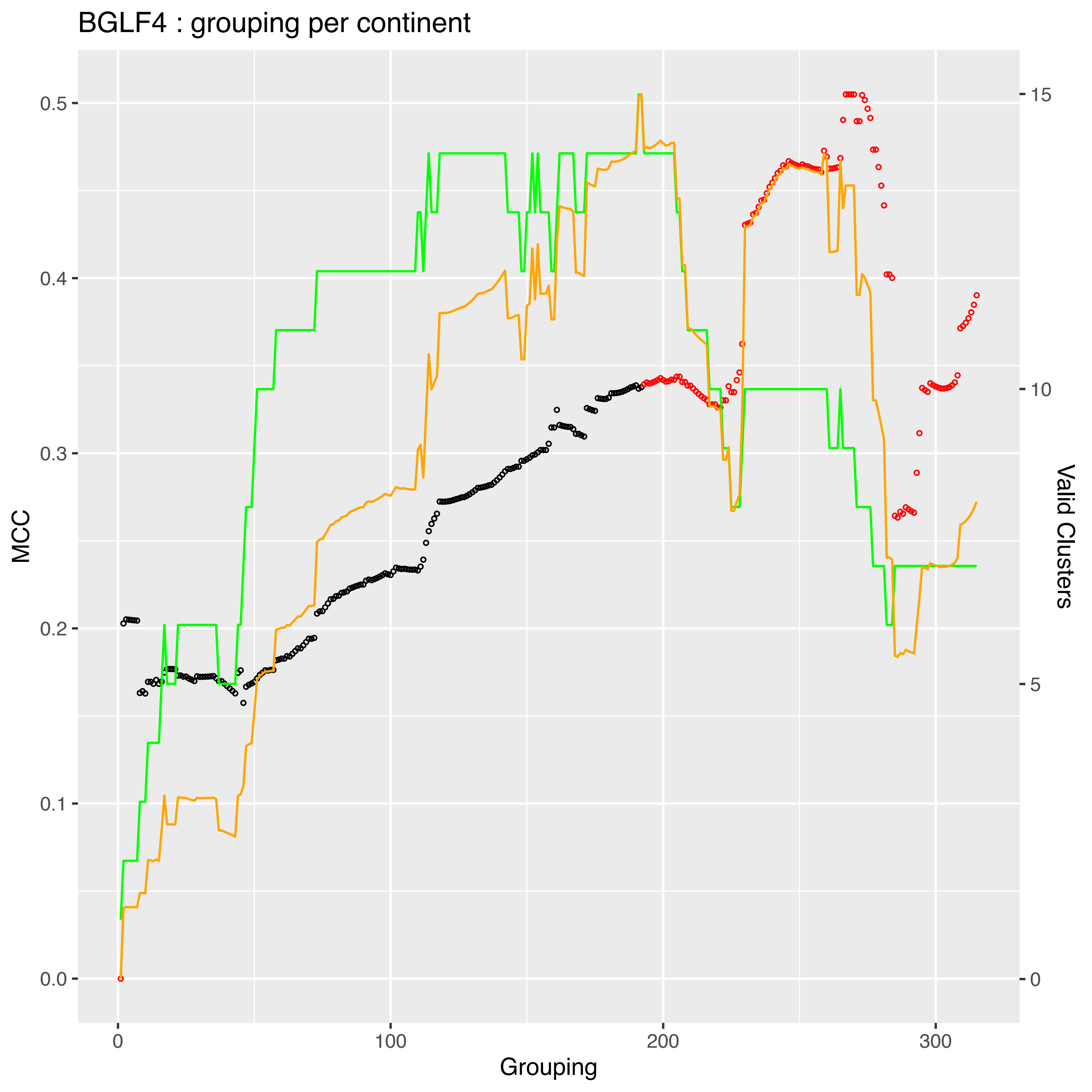
BGLF4 : grouping per continent
MCC
Valid Clusters
Grouping
0.0
0.1
0.2
0.3
0.4
0.5
0
5
10
15
0
100
200
300

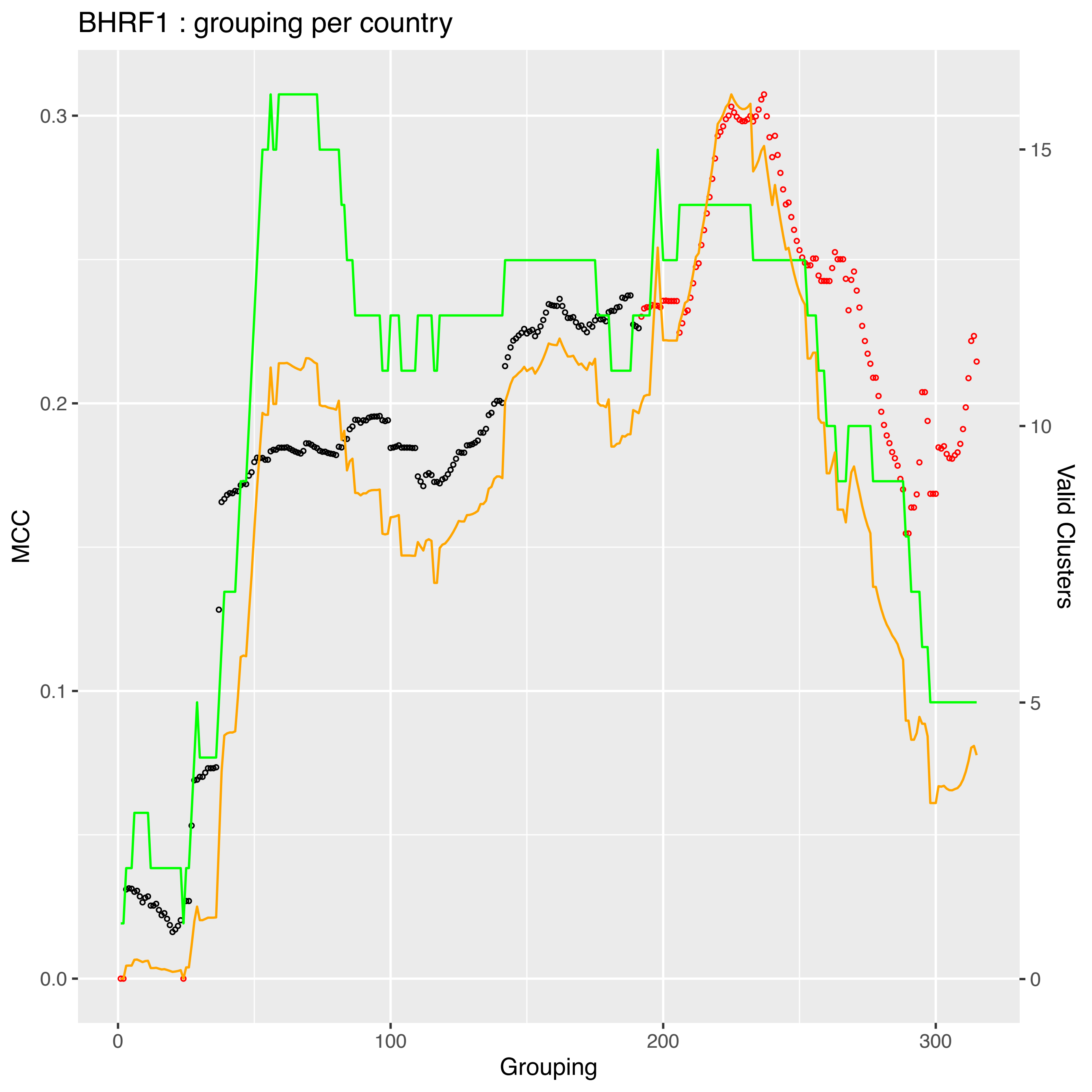

BHRF1 : grouping per country
MCC
Valid Clusters
Grouping
0.0
0.1
0.2
0.3
0
5
10
15
0
100
200
300

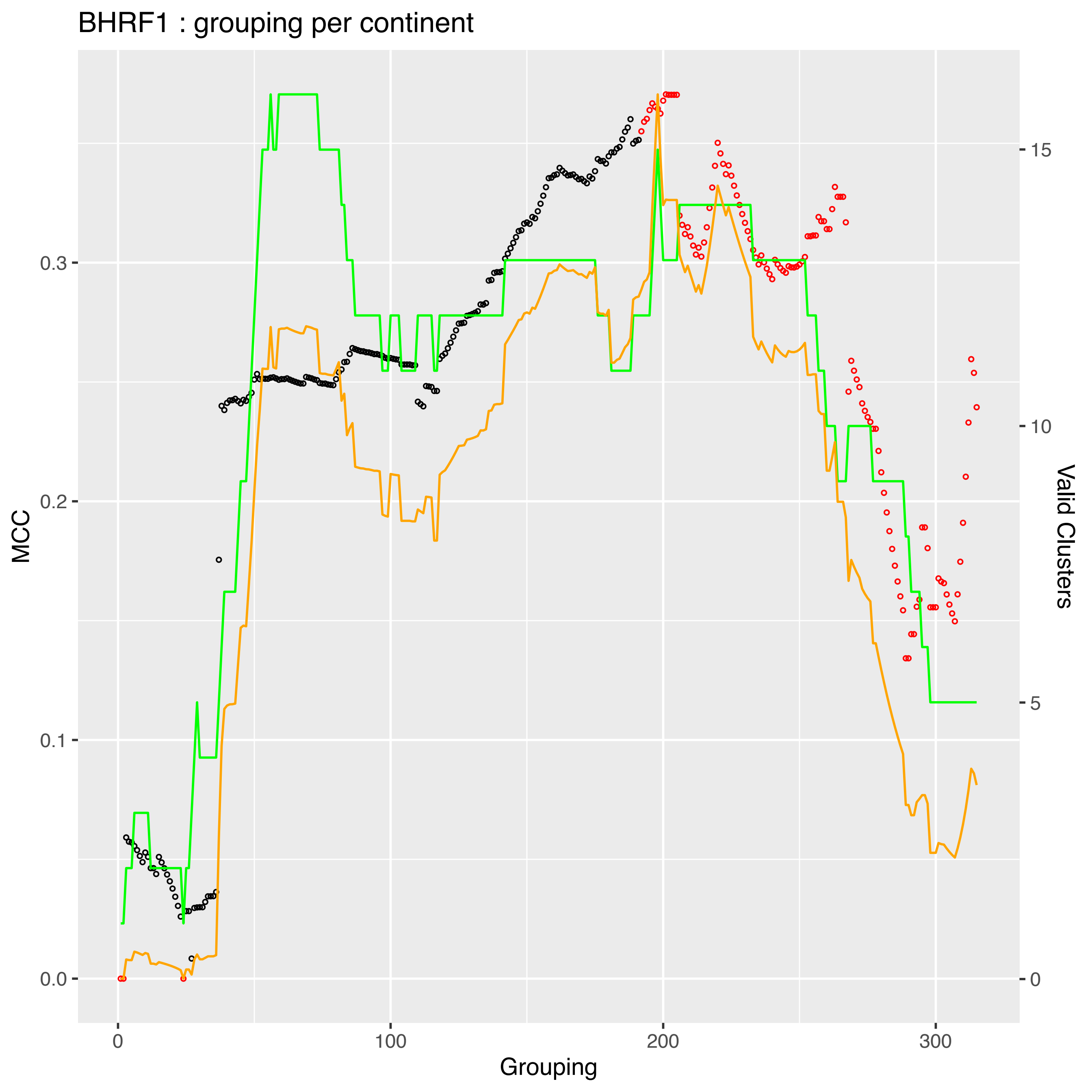

BHRF1 : grouping per continent
MCC
Valid Clusters
Grouping
0.0
0.1
0.2
0.3
0
5
10
15
0
100
200
300

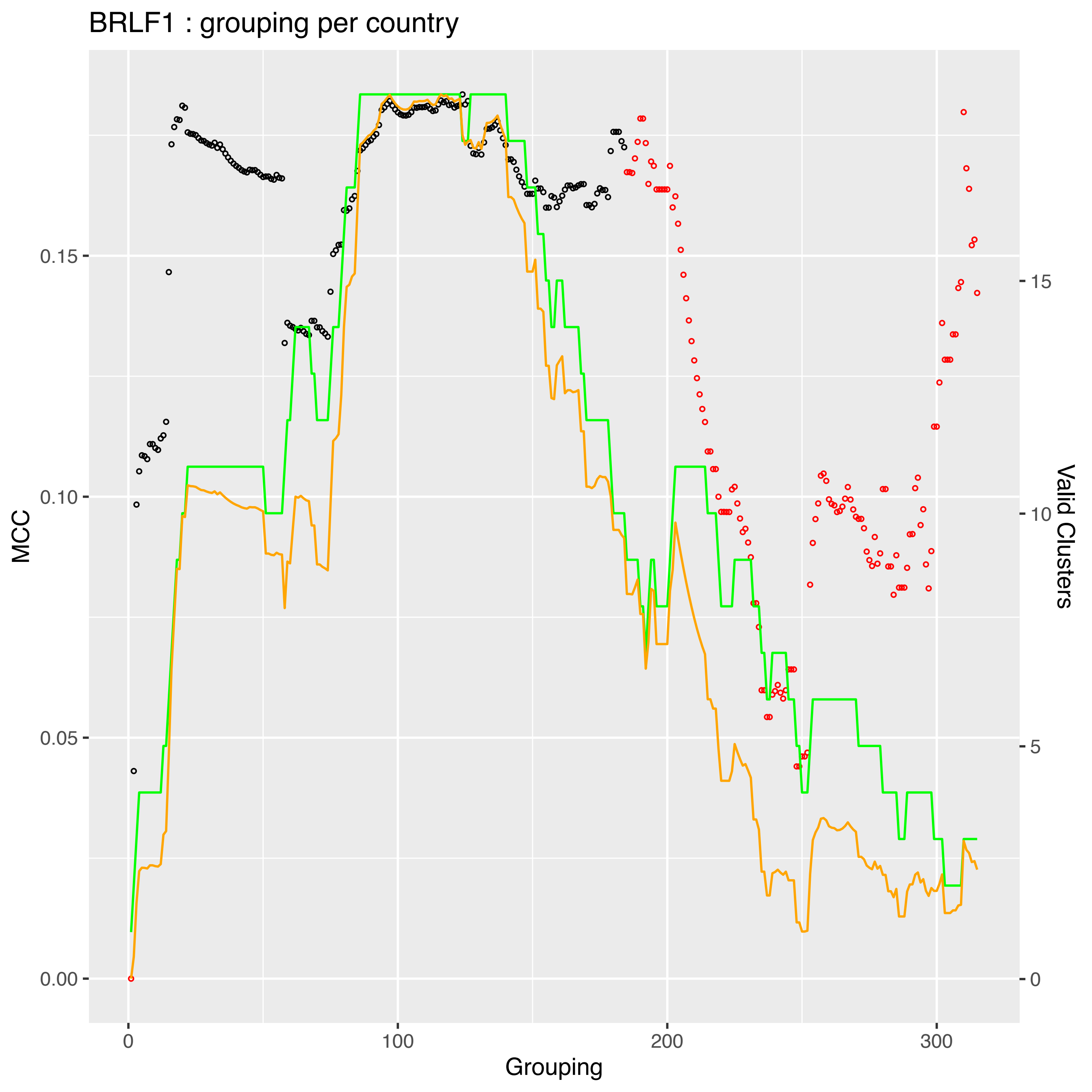
BRLF1 : grouping per country
MCC
0.00
0.05
0.10
0.15
Valid Clusters
0
5
10
15
Grouping
0
100
200
300

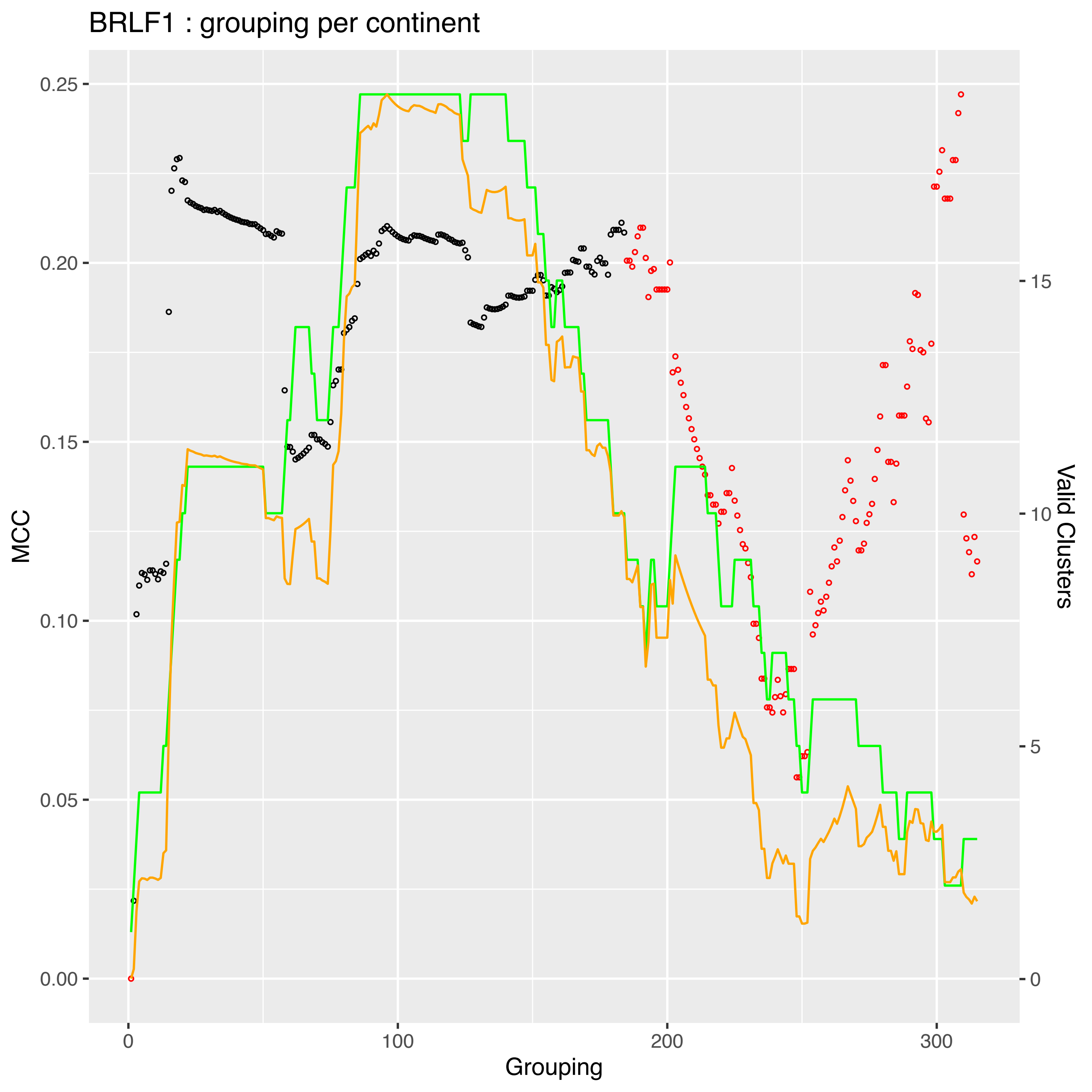
BRLF1 : grouping per continent
MCC
0.25
0.20
0.15
0.10
0.05
0.00
Valid Clusters
15
10
5
0
Grouping
0
100
200
300

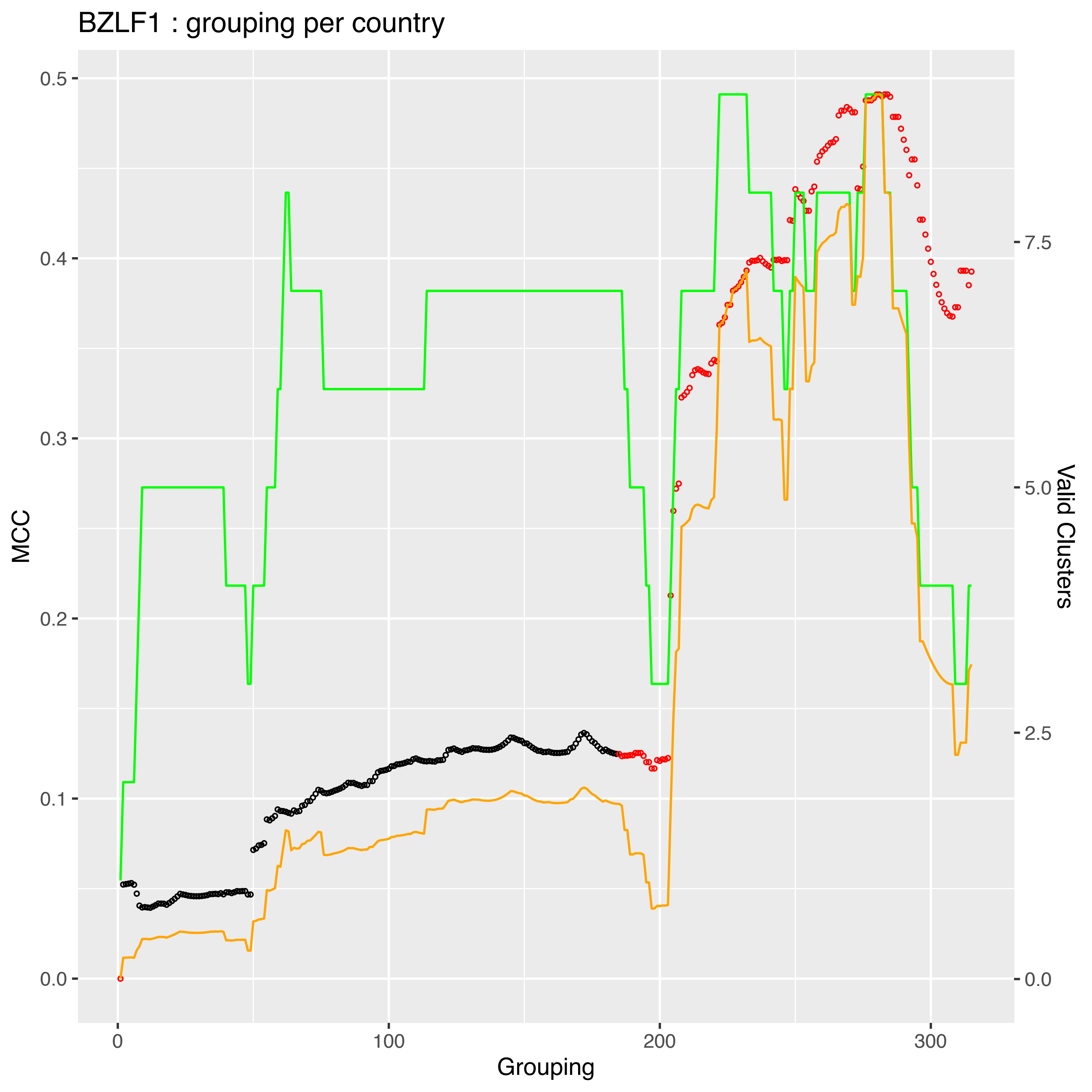

BZLF1 : grouping per country
MCC
Valid Clusters
Grouping
0.0
0.1
0.2
0.3
0.4
0.5
0.0
2.5
5.0
7.5
0
100
200
300

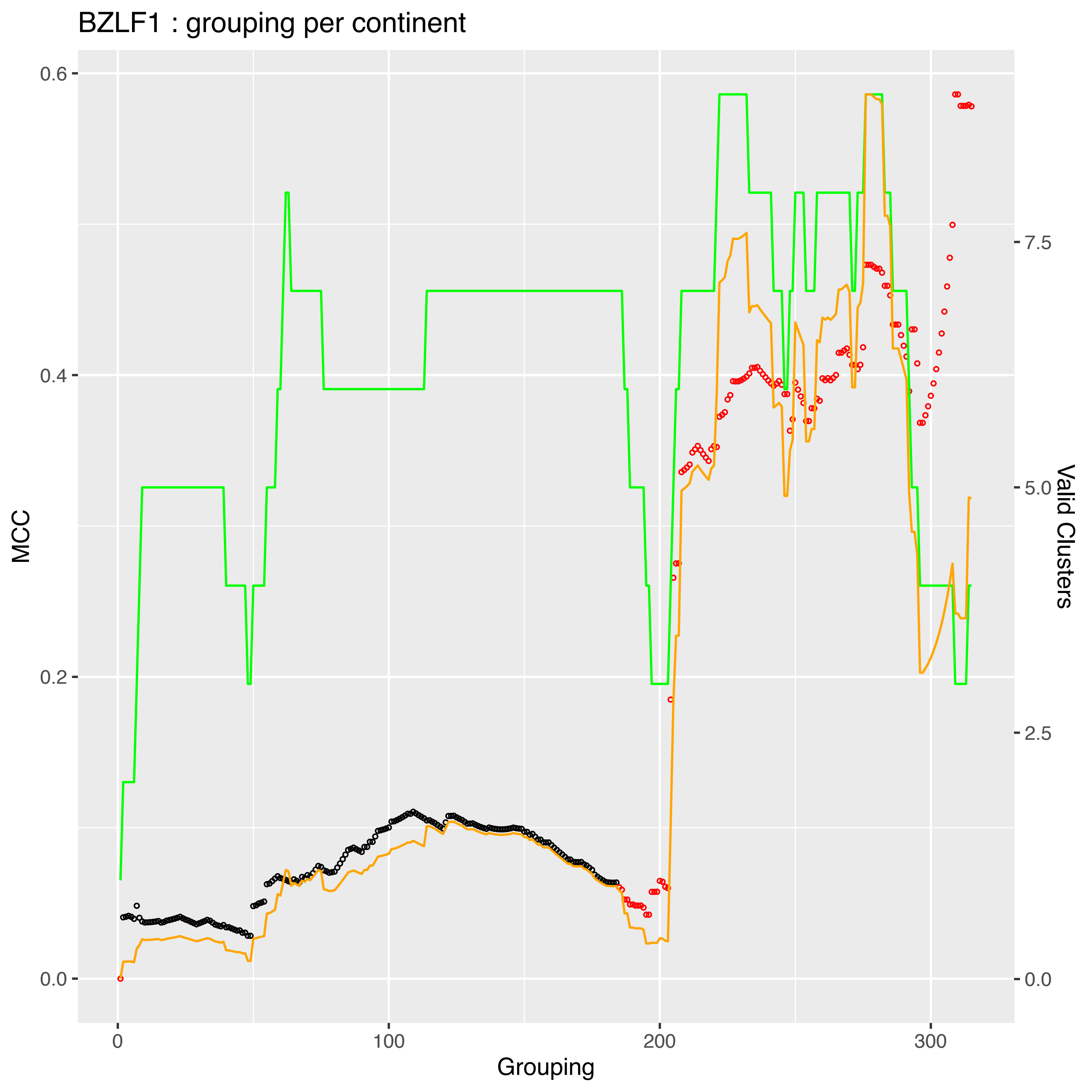
BZLF1 : grouping per continent
MCC
Valid Clusters
Grouping
0.0
0.2
0.4
0.6
0.0
2.5
5.0
7.5
0
100
200
300

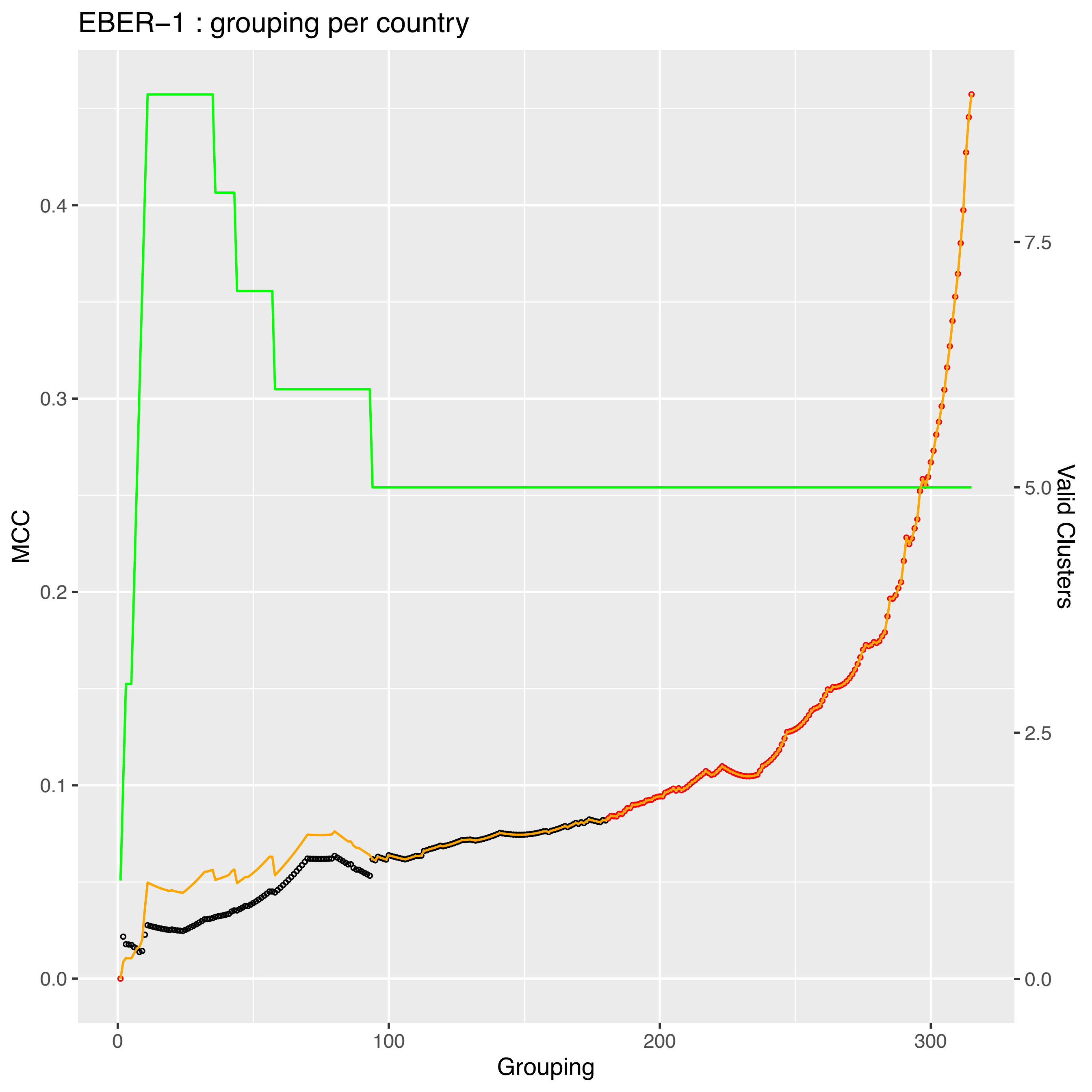

EBER−1 : grouping per country
MCC
0.0
0.1
0.2
0.3
0.4
Valid Clusters
0.0
2.5
5.0
7.5
0
100
200
300
Grouping

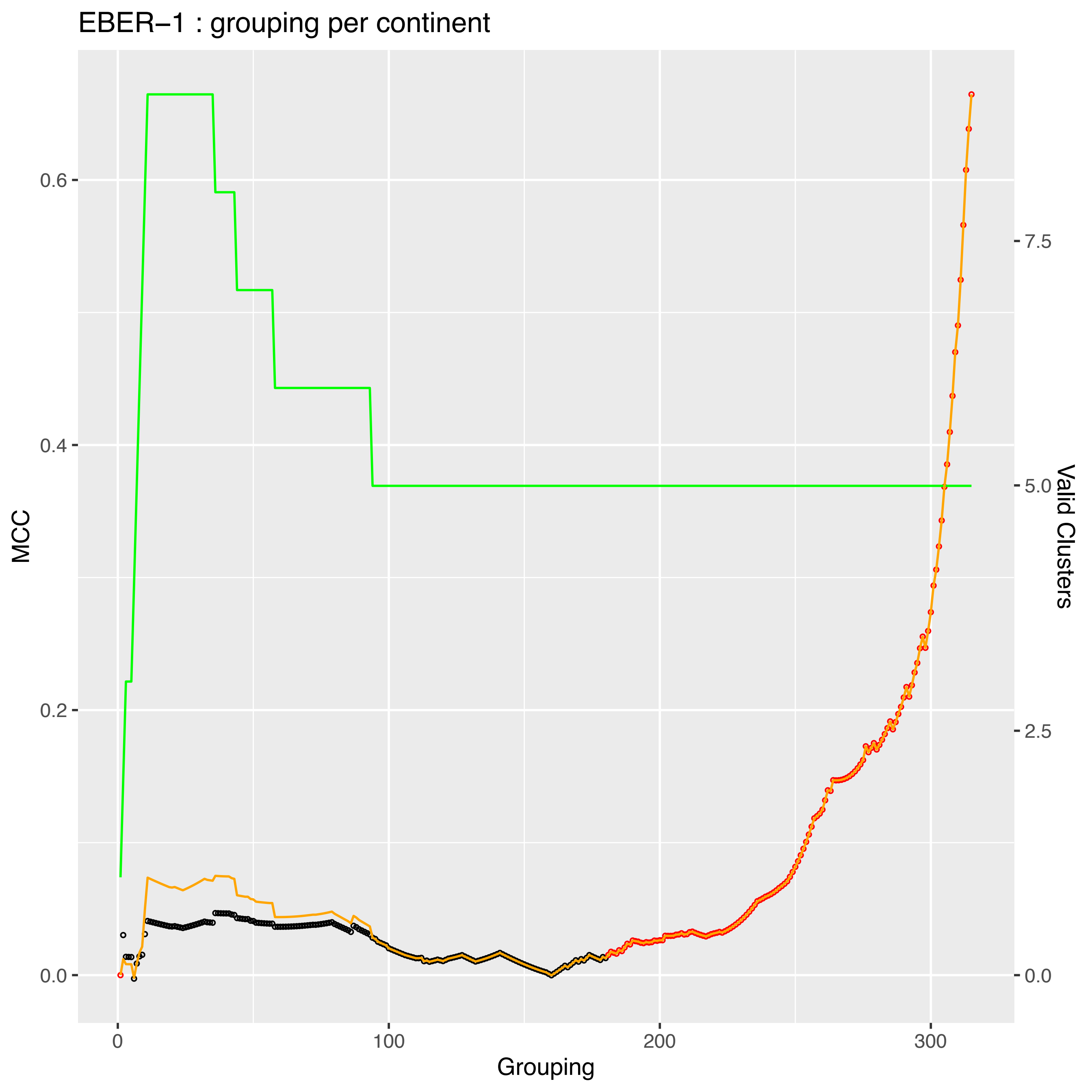
EBER−1 : grouping per continent
MCC
0.0
0.2
0.4
0.6
Valid Clusters
0.0
2.5
5.0
7.5
Grouping
0
100
200
300

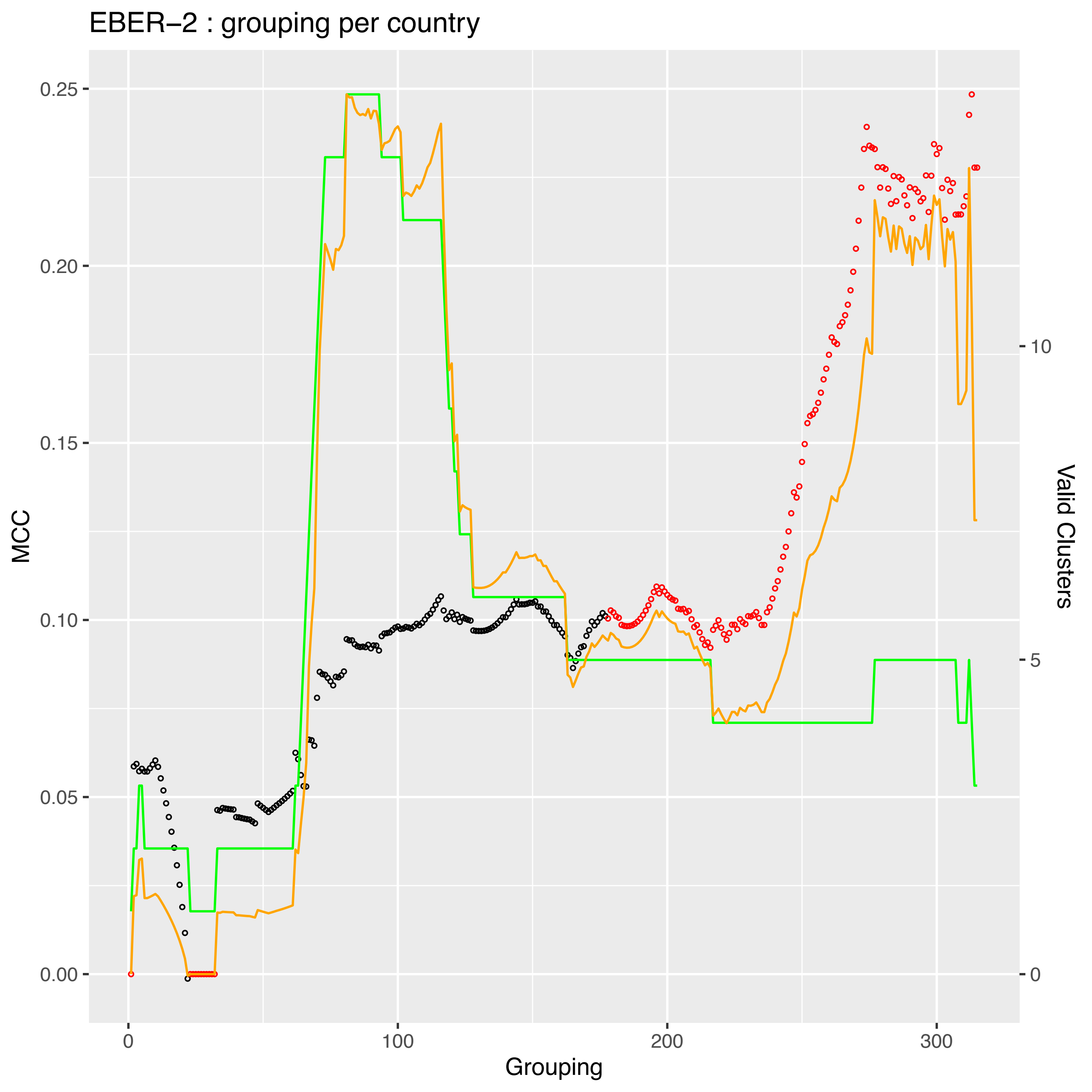

EBER−2 : grouping per country
MCC
0.25
0.20
0.15
0.10
0.05
0.00
Valid Clusters
10
5
0
0
100
200
300
Grouping

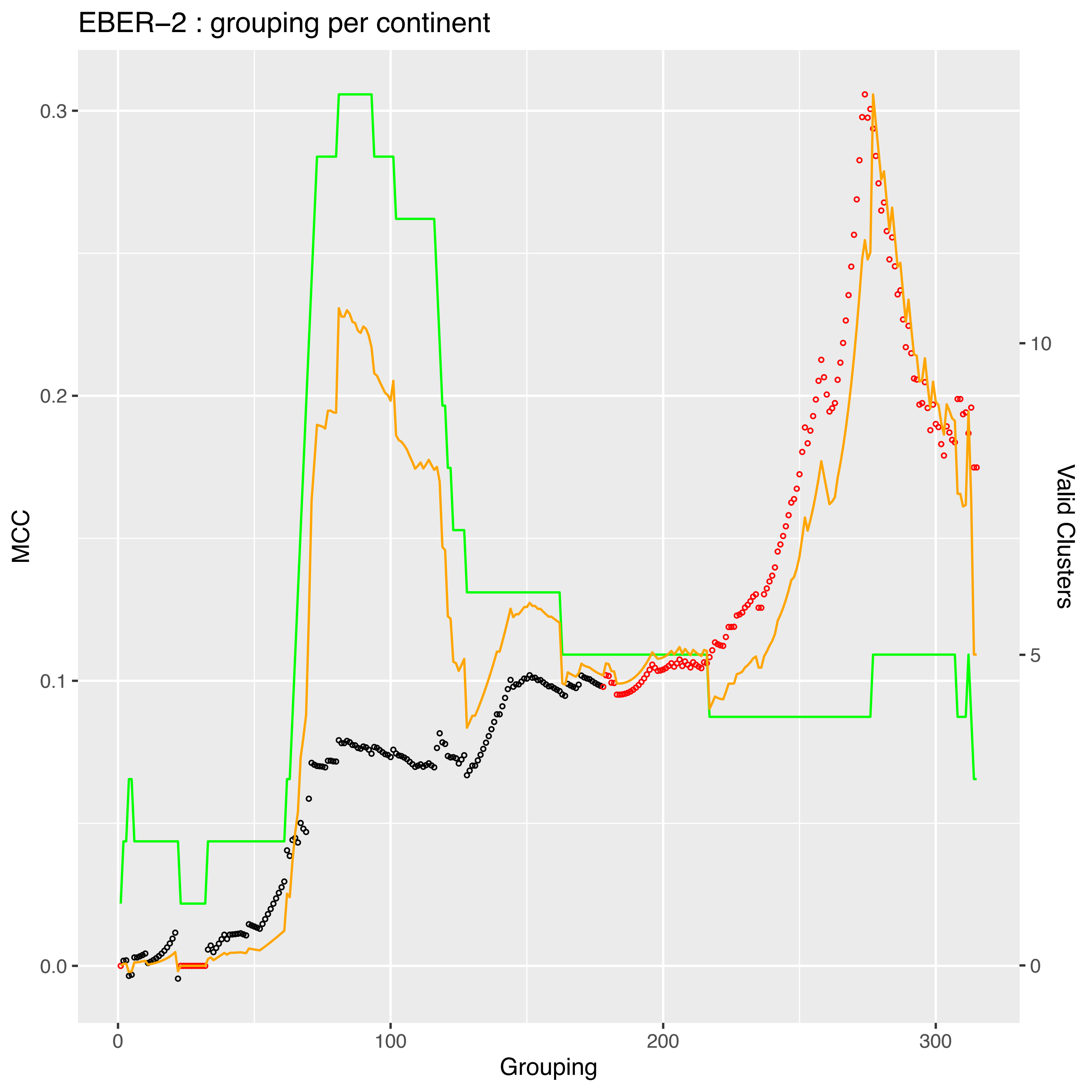

EBER−2 : grouping per continent
MCC
0.0
0.1
0.2
0.3
Valid Clusters
0
5
10
Grouping
0
100
200
300

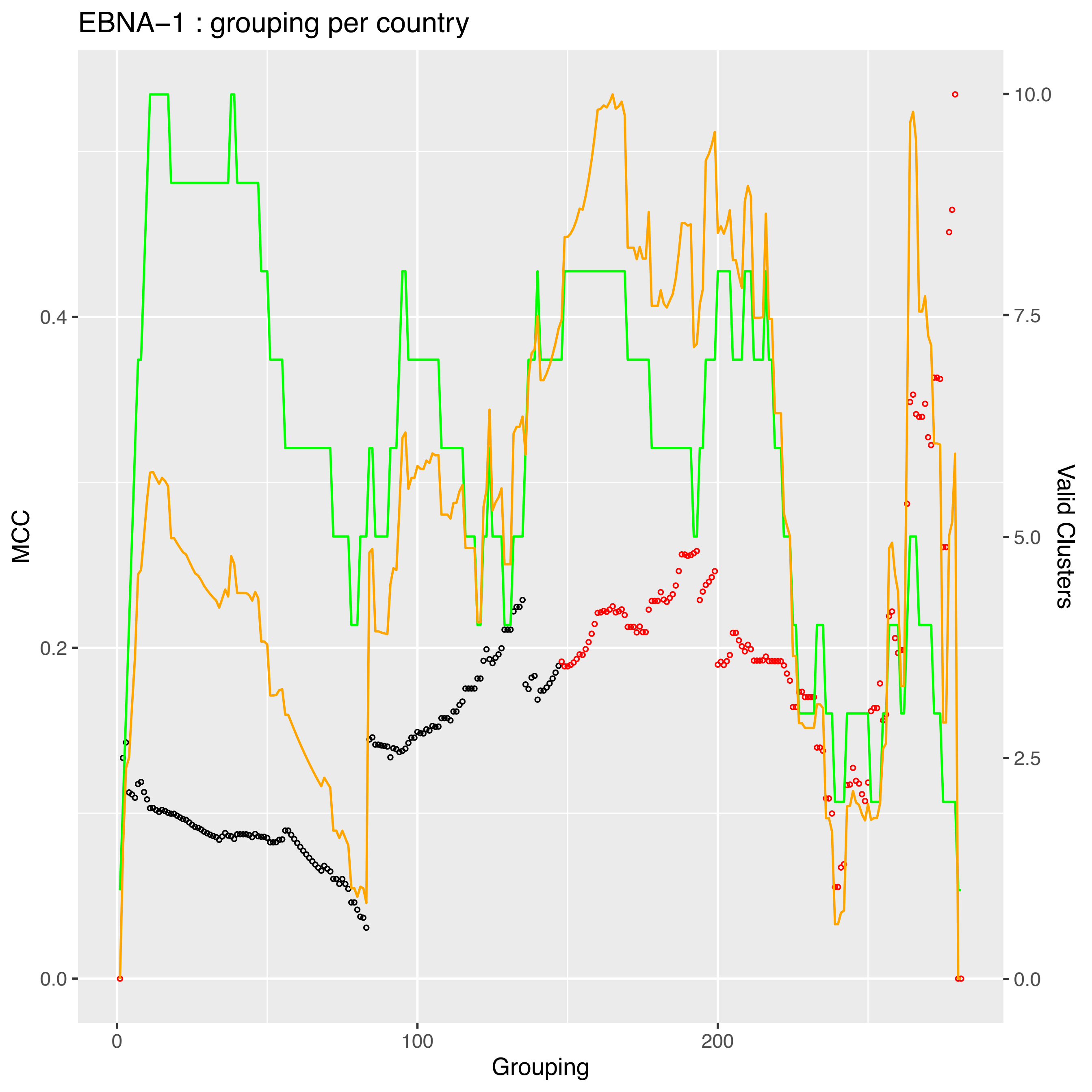

EBNA−1 : grouping per country
MCC
0.0
0.2
0.4
Valid Clusters
0.0
2.5
5.0
7.5
10.0
Grouping
0
100
200

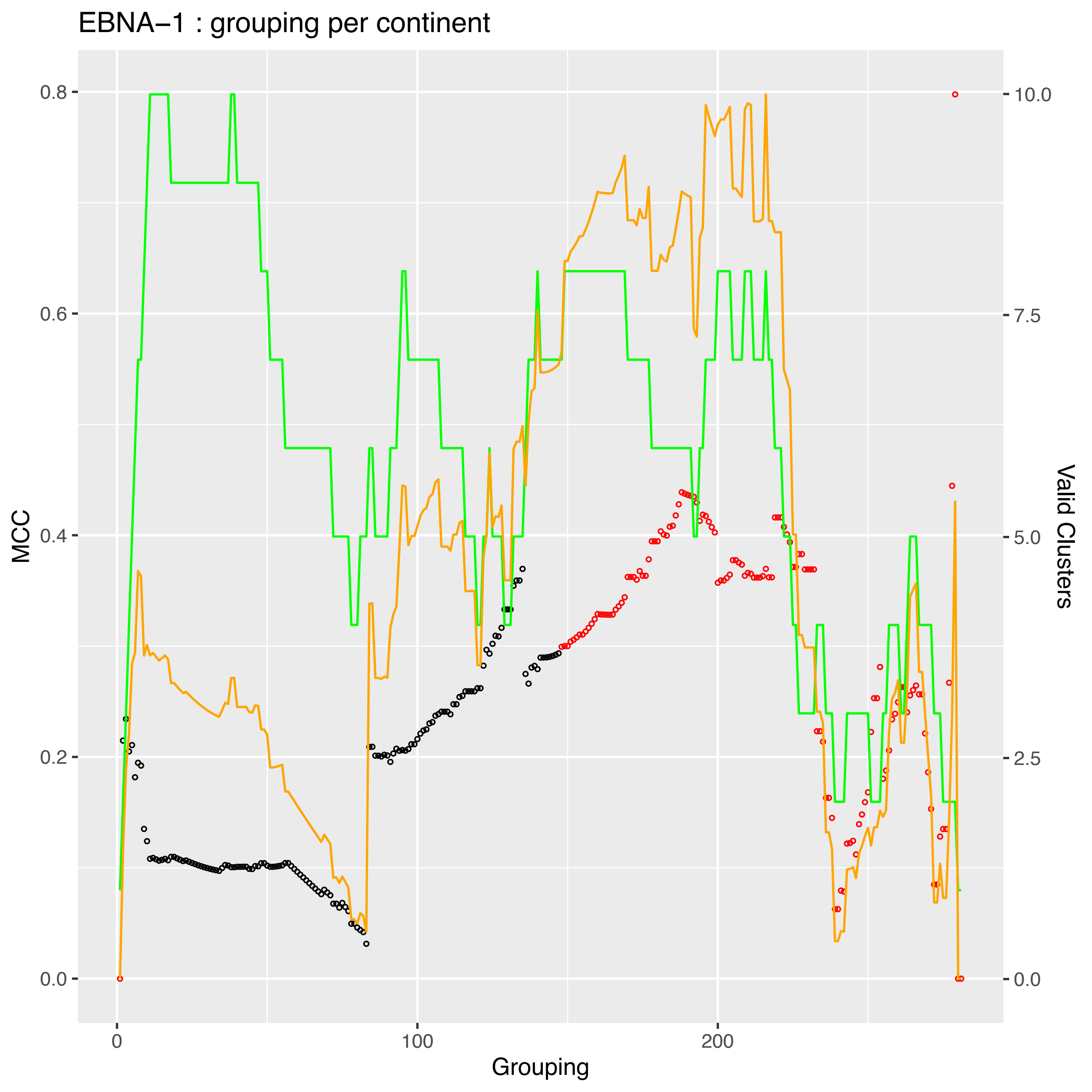
EBNA−1 : grouping per continent
MCC
Valid Clusters
Grouping
0.0
0.2
0.4
0.6
0.8
0.0
2.5
5.0
7.5
10.0
0
100
200

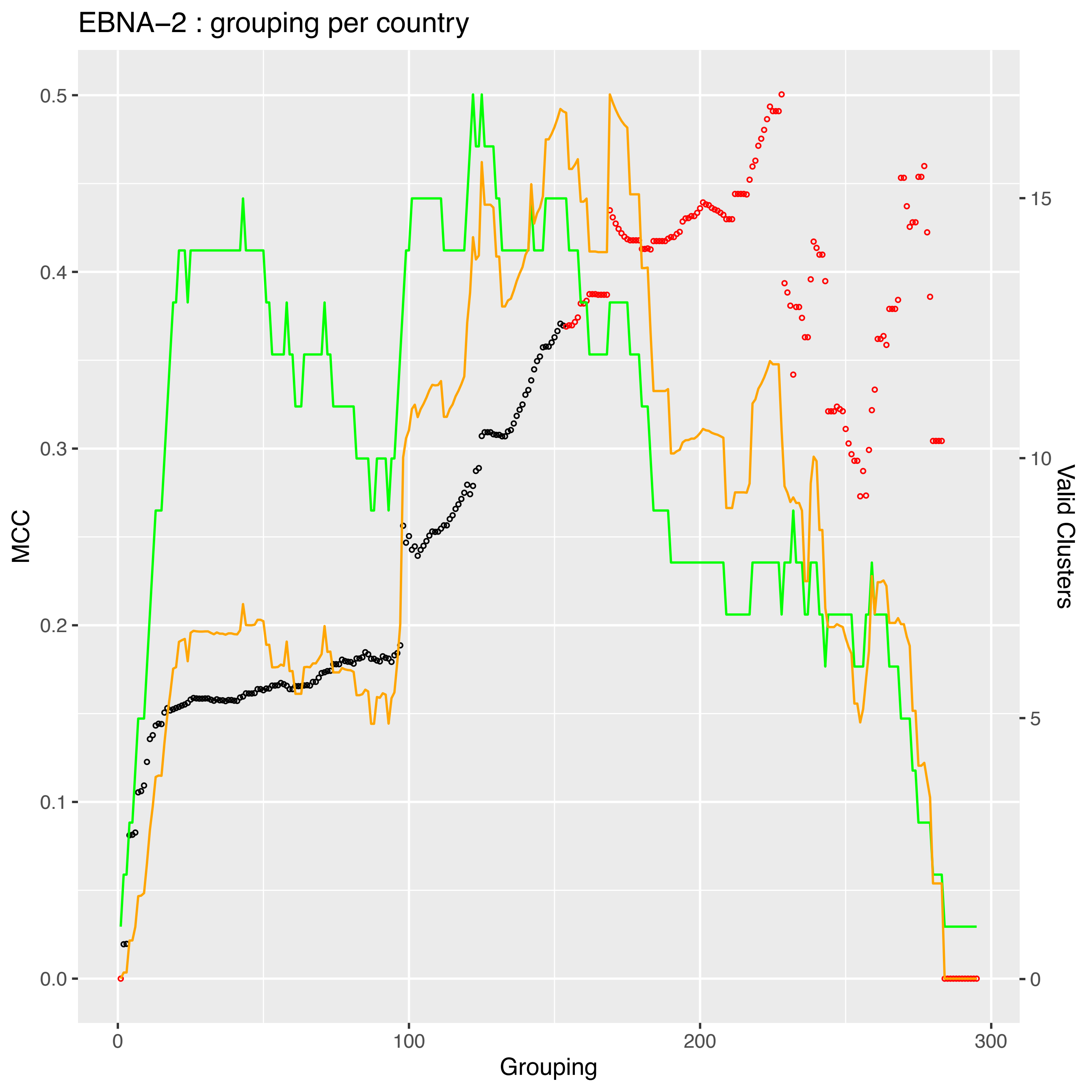
EBNA−2 : grouping per country
MCC
Valid Clusters
Grouping
0.0
0.1
0.2
0.3
0.4
0.5
0
100
200
300
0
5
10
15

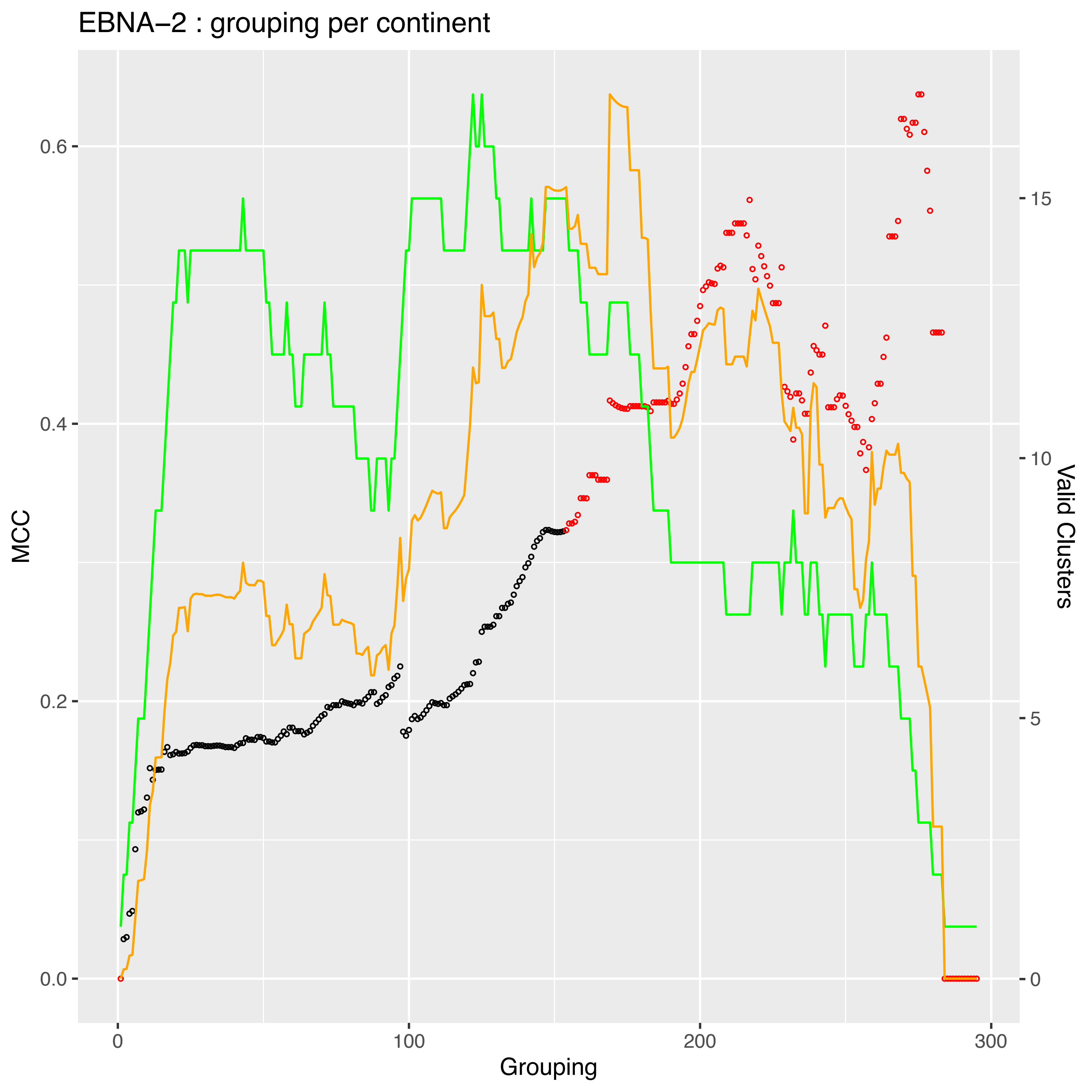

EBNA−2 : grouping per continent
MCC
Valid Clusters
Grouping
0.0
0.2
0.4
0.6
0
5
10
15
0
100
200
300

EBNA−2_type1 : grouping per country

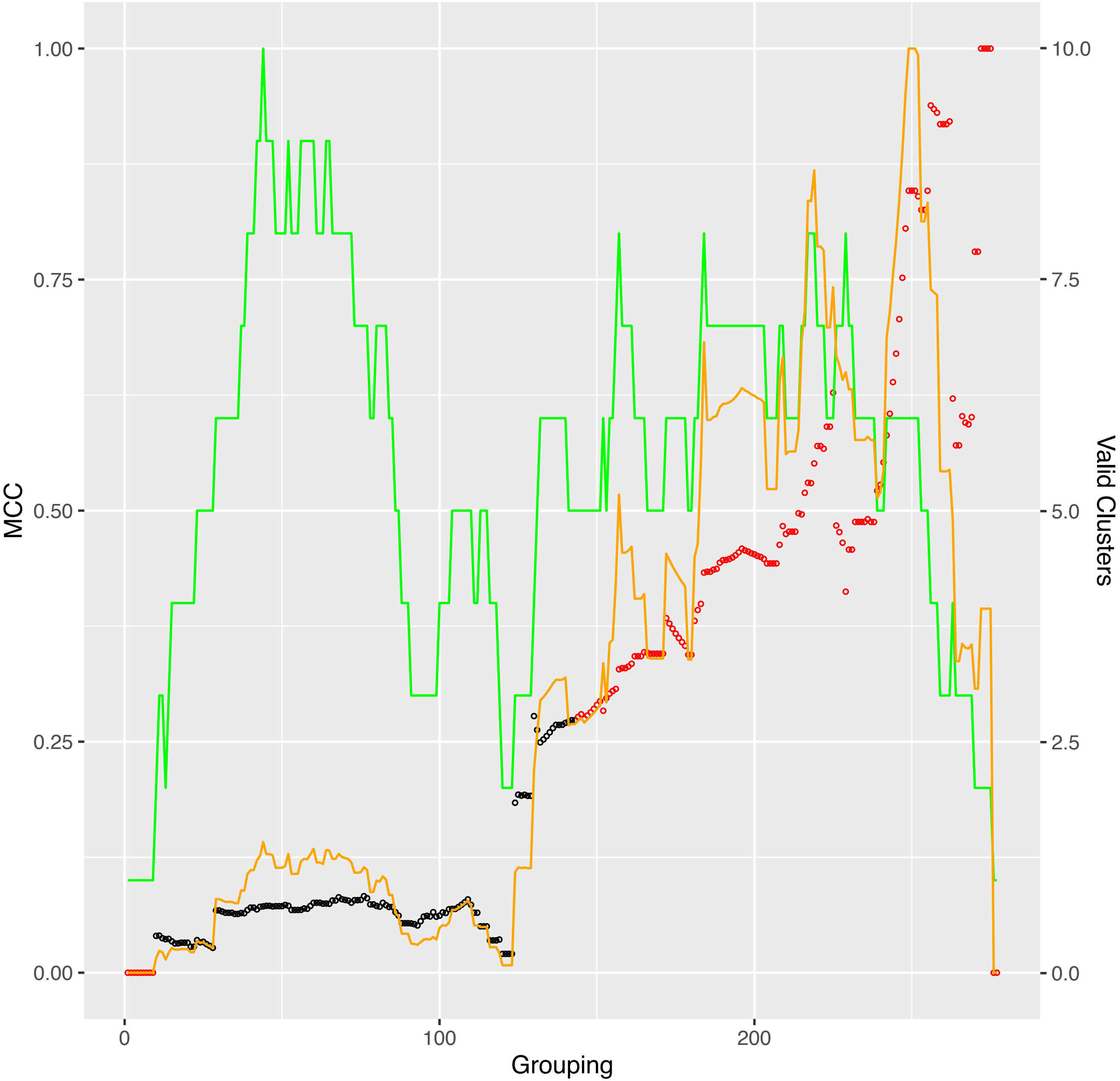

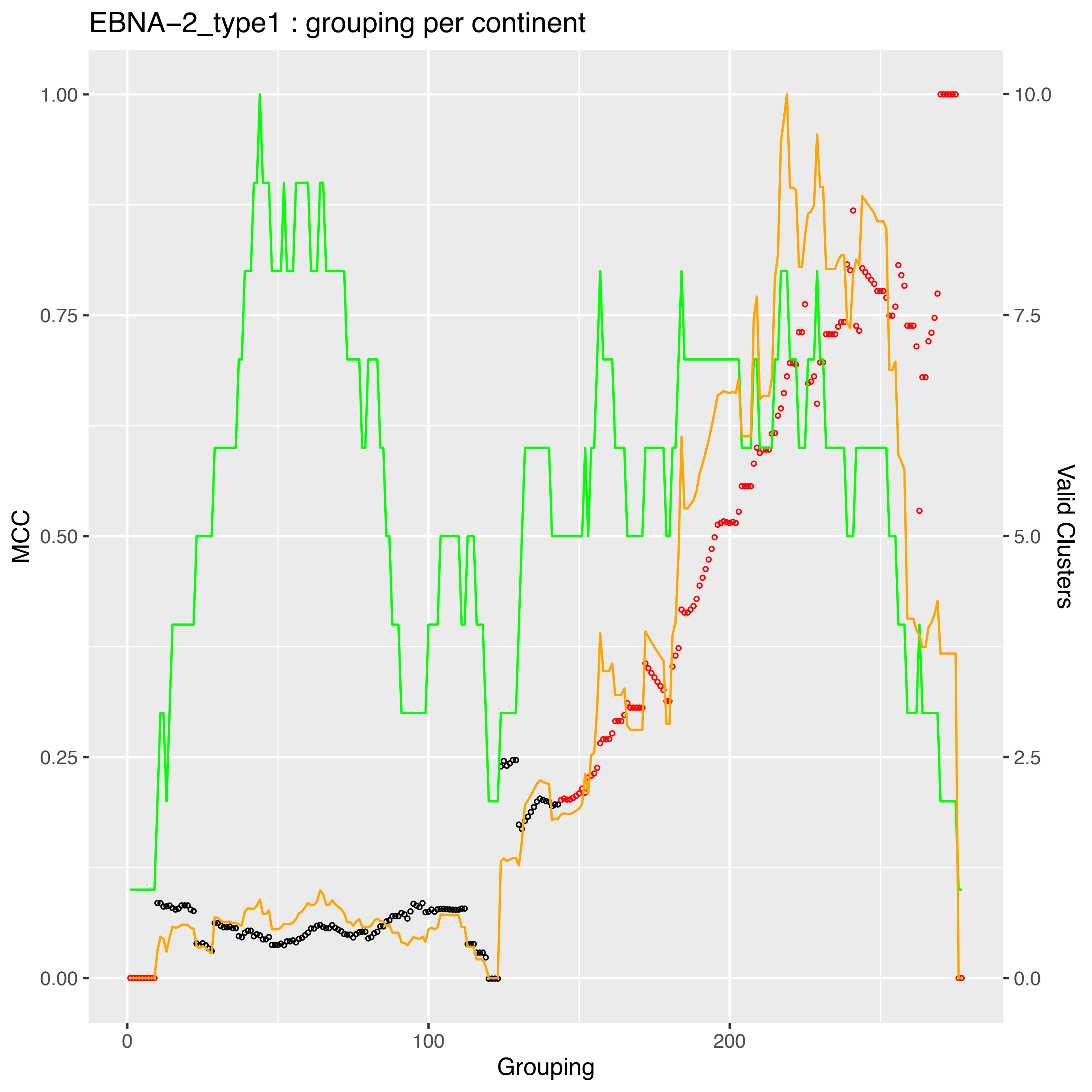
EBNA−2_type1 : grouping per continent
MCC
1.00
0.75
0.50
0.25
0.00
Valid Clusters
10.0
7.5
5.0
2.5
0.0
0
100
200
Grouping

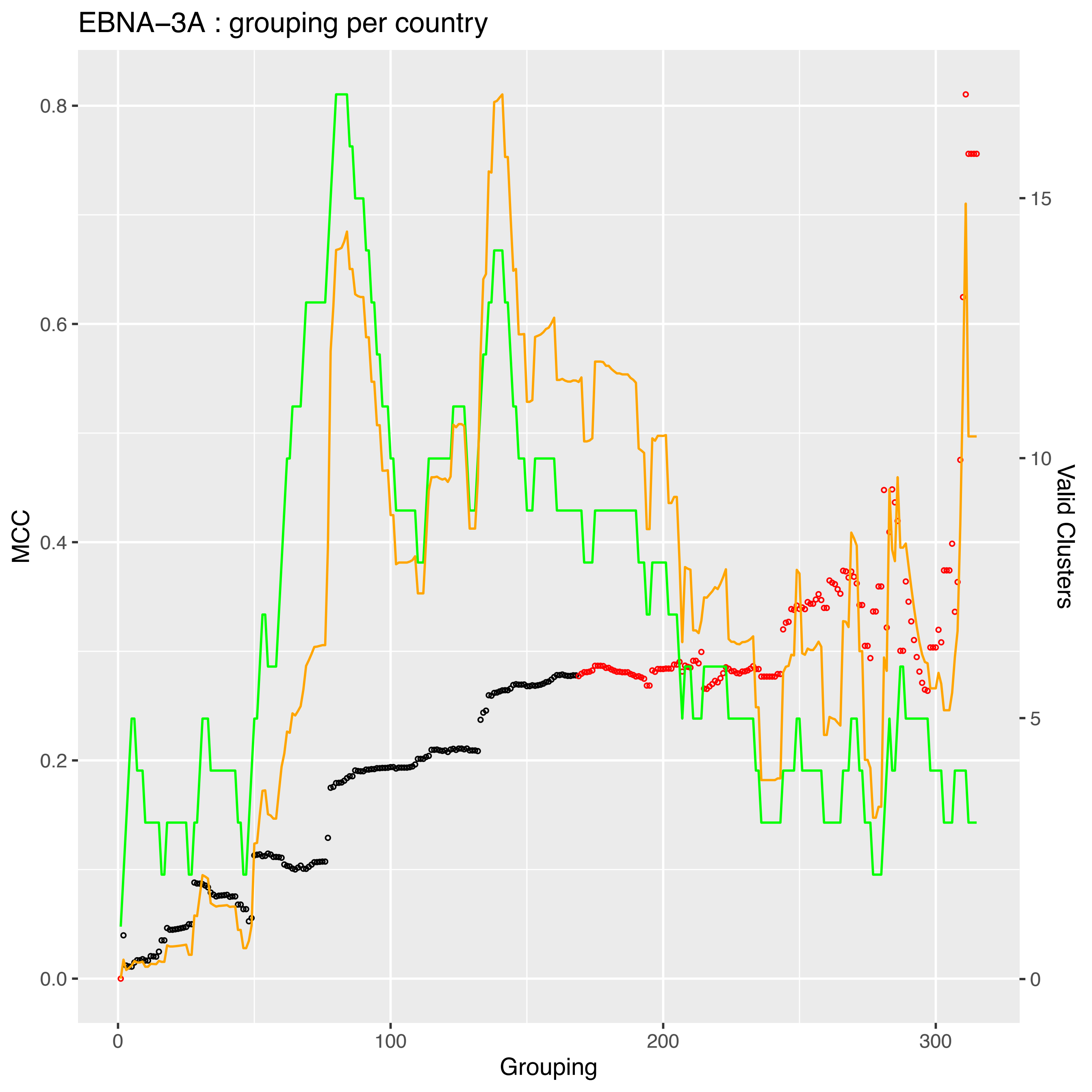
EBNA−3A : grouping per country
MCC
0.0
0.2
0.4
0.6
0.8
Valid Clusters
0
5
10
15
Grouping
0
100
200
300

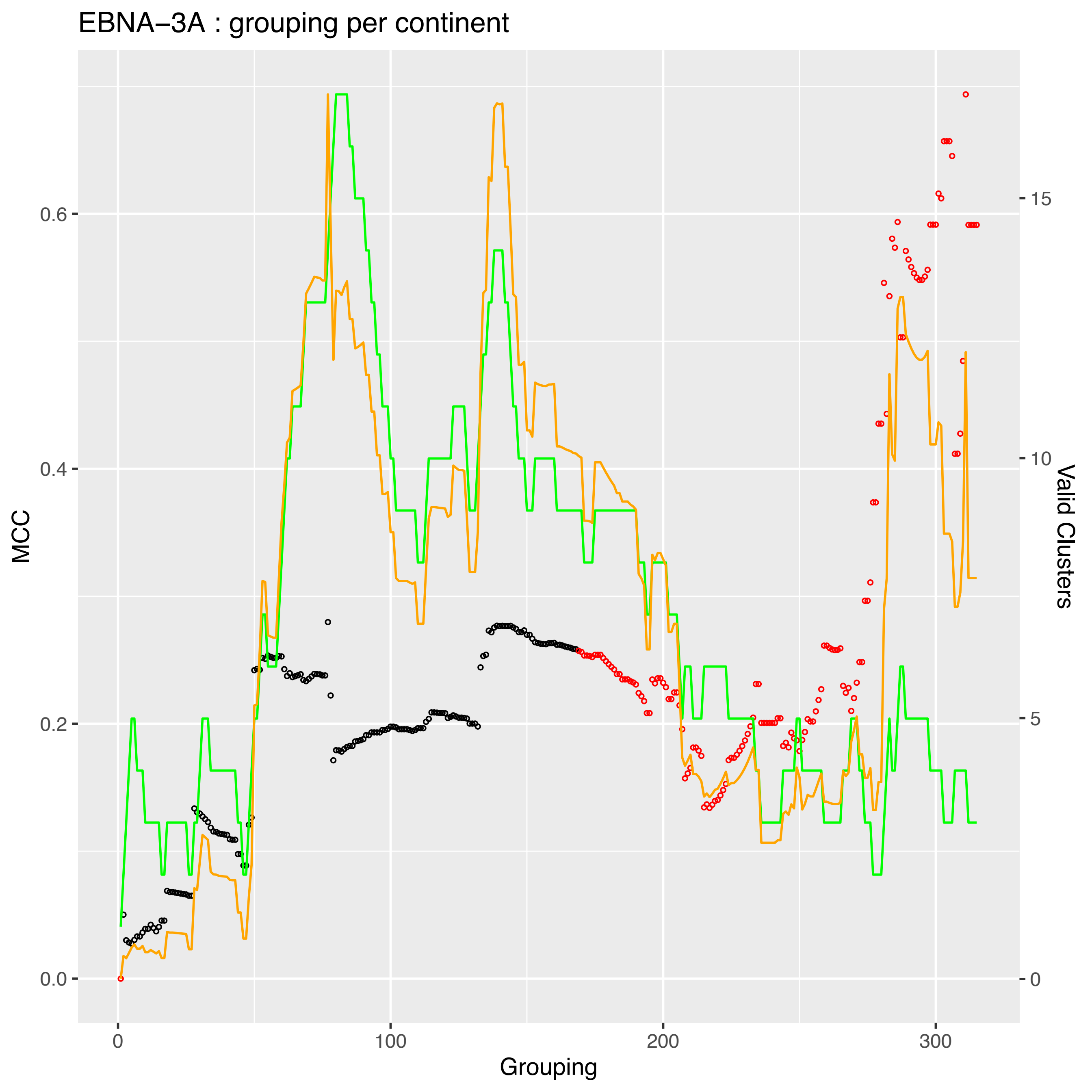

EBNA−3A : grouping per continent
MCC
0.0
0.2
0.4
0.6
Valid Clusters
0
5
10
15
Grouping
0
100
200
300

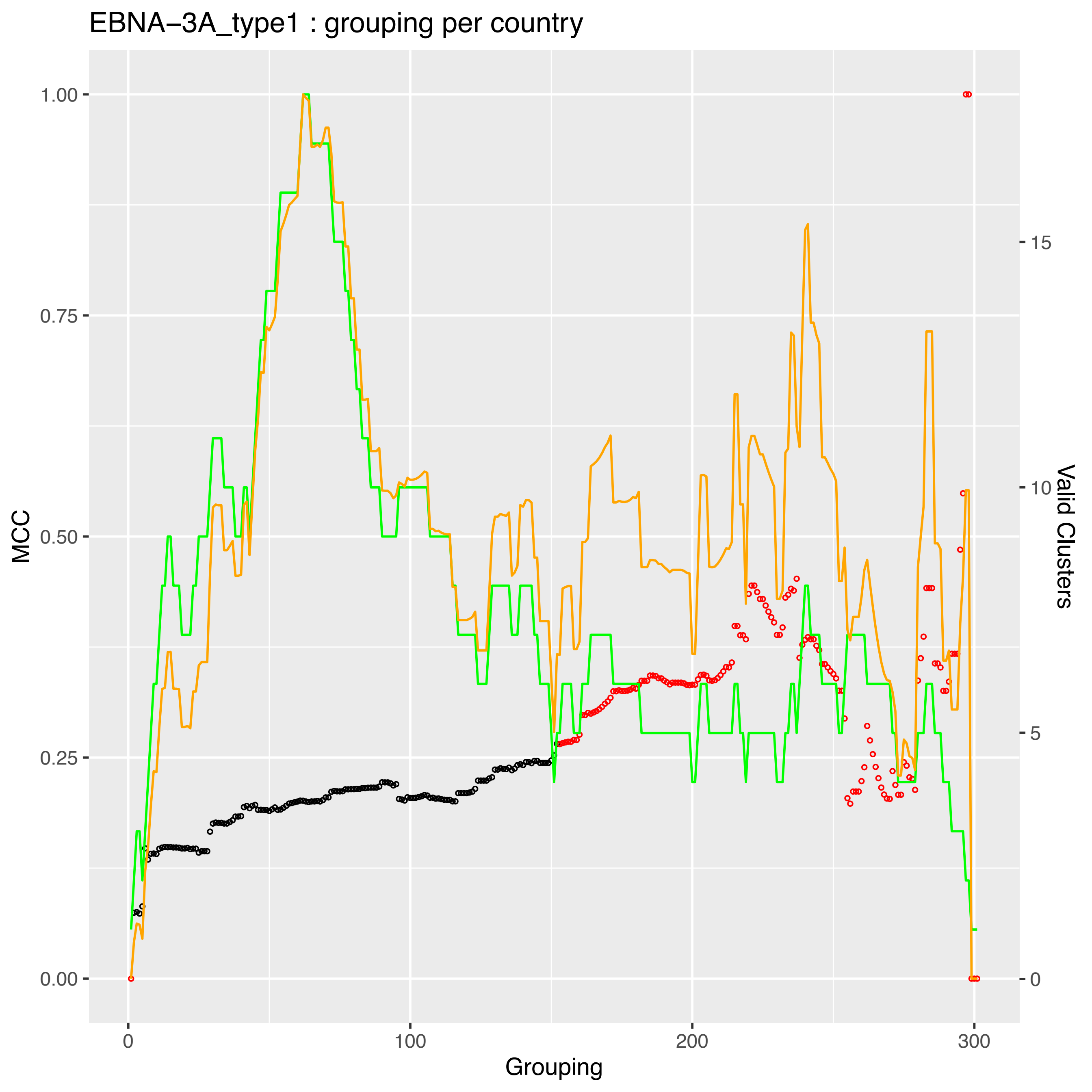
EBNA−3A_type1 : grouping per country
MCC
Valid Clusters
Grouping
0.00
0.25
0.50
0.75
1.00
0
5
10
15
0
100
200
300

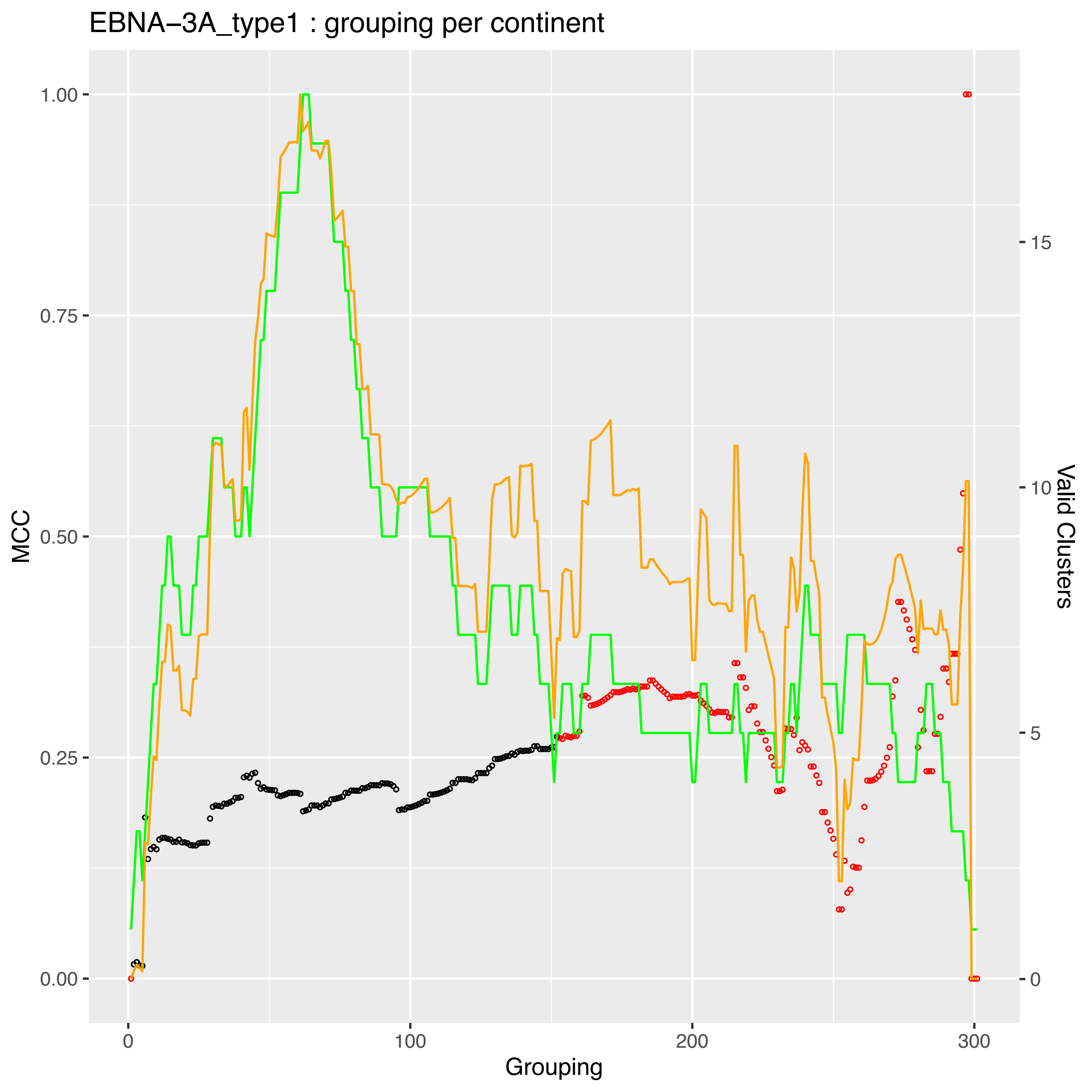
EBNA−3A_type1 : grouping per continent
MCC
1.00
0.75
0.50
0.25
0.00
Valid Clusters
15
10
5
0
0
100
200
300
Grouping

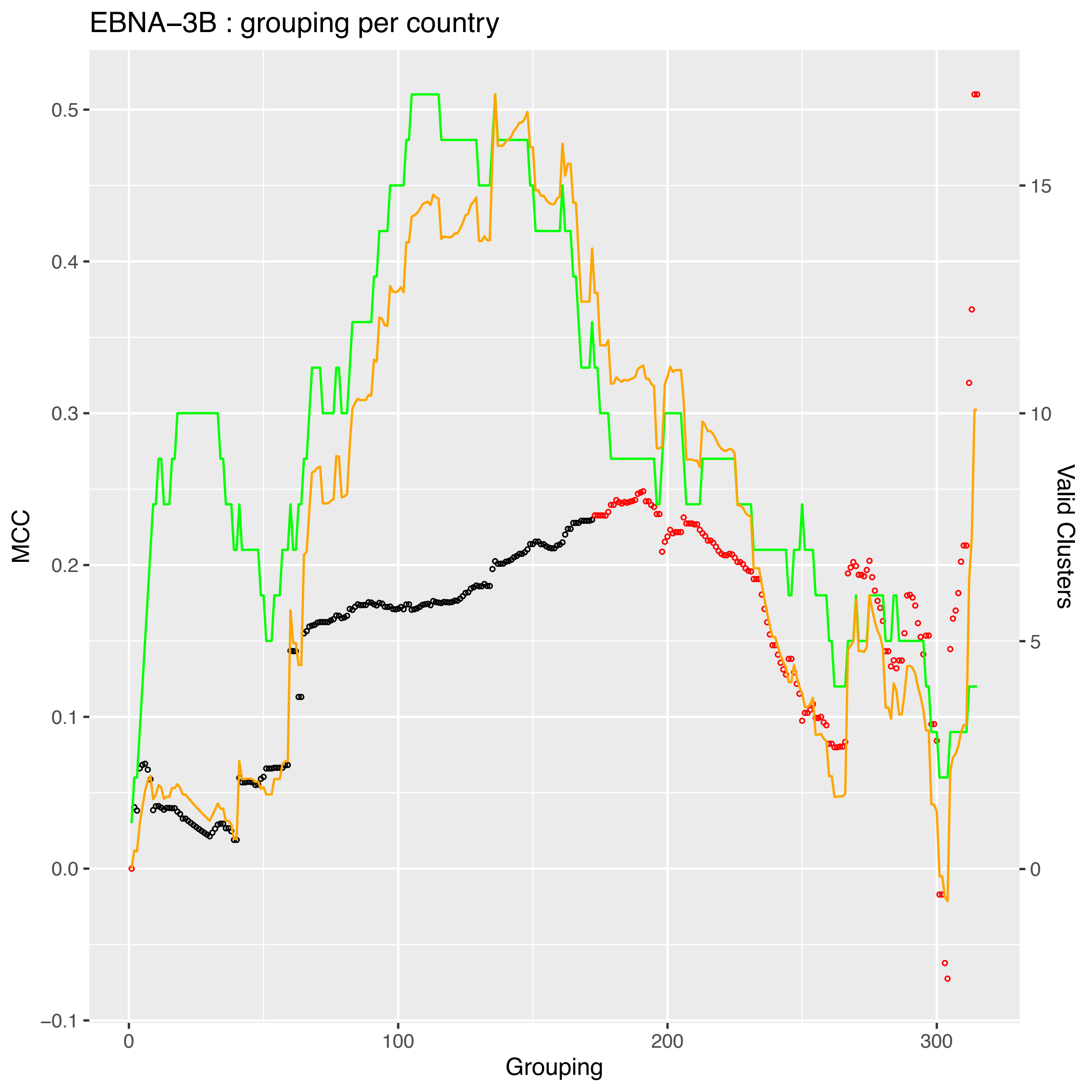
EBNA−3B : grouping per country
MCC
Valid Clusters
Grouping
0.5
0.4
0.3
0.2
0.1
0.0
−0.1
15
10
5
0
0
100
200
300

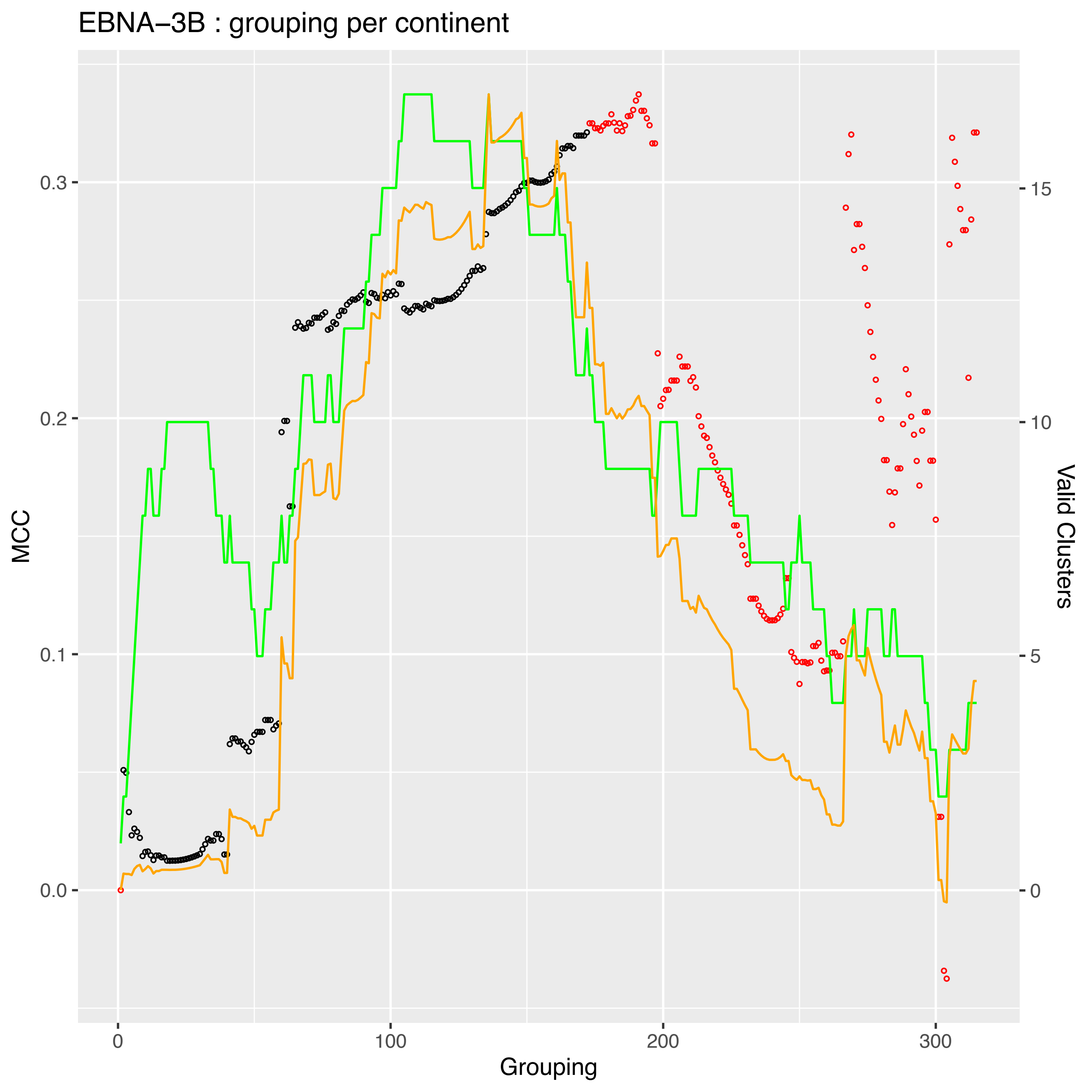
EBNA−3B : grouping per continent
MCC
Valid Clusters
Grouping
0.0
0.1
0.2
0.3
0
5
10
15
0
100
200
300

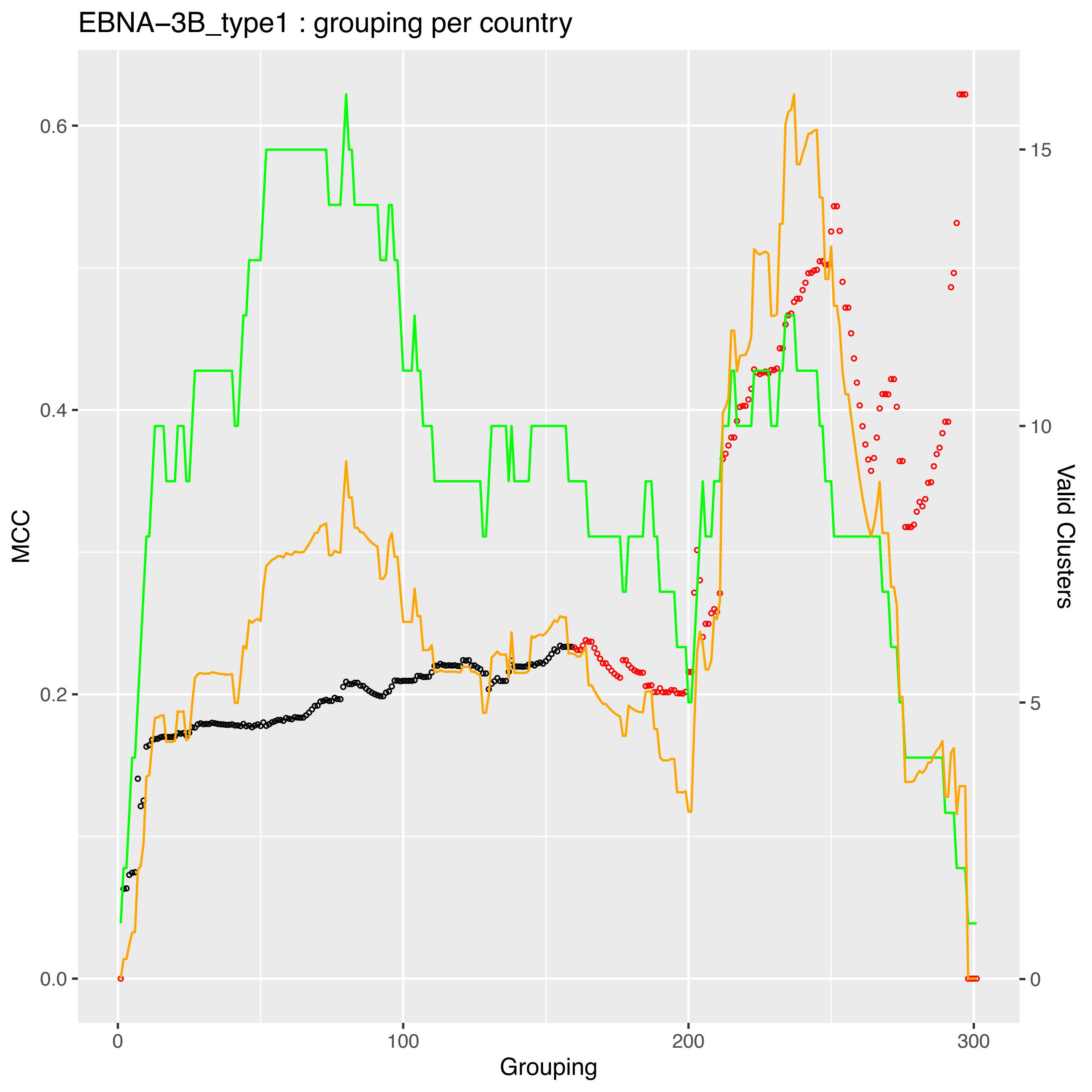
EBNA−3B_type1 : grouping per country
MCC
Valid Clusters
Grouping
0.0
0.2
0.4
0.6
0
5
10
15
0
100
200
300

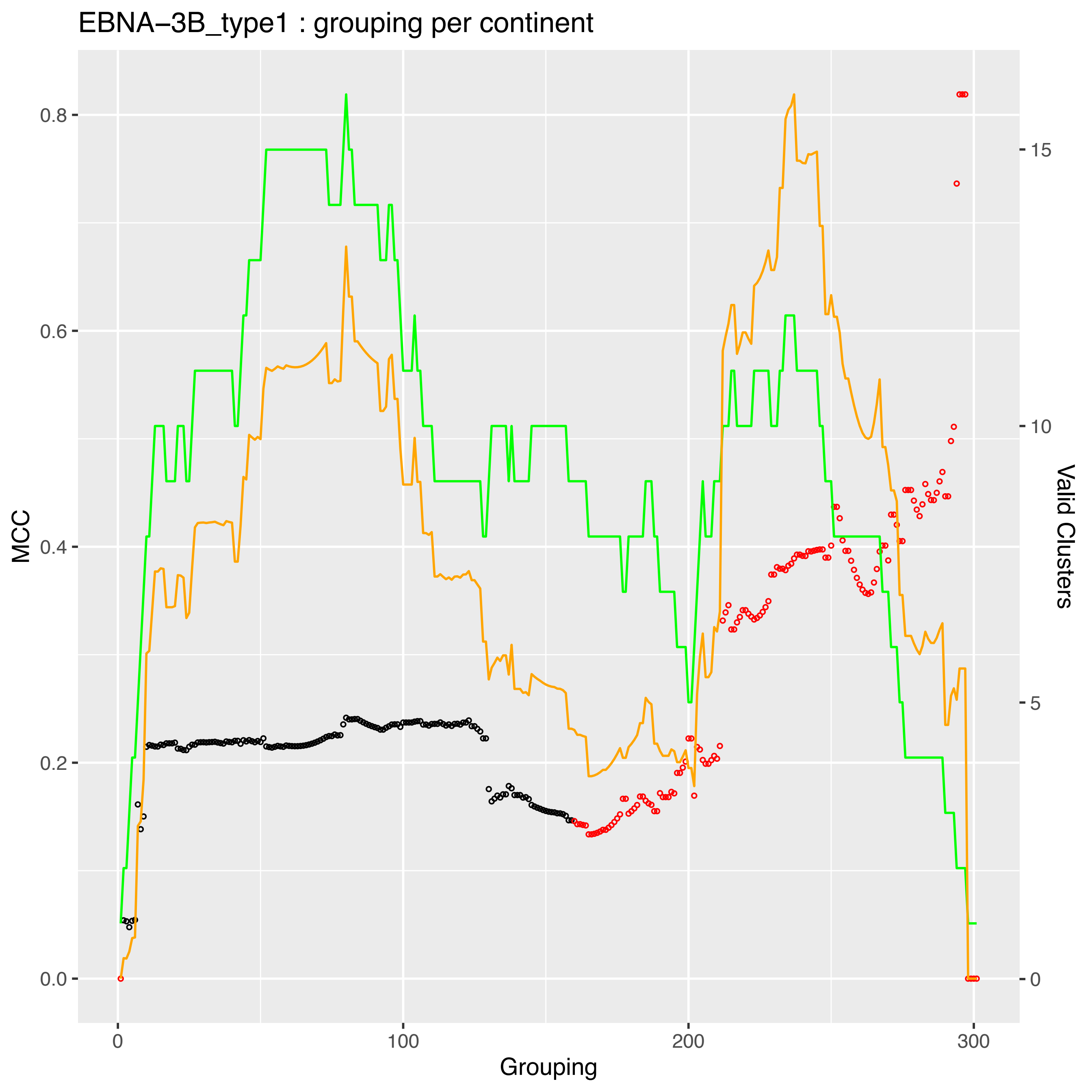

EBNA−3B_type1 : grouping per continent
MCC
Valid Clusters
Grouping
0.0
0.2
0.4
0.6
0.8
0
5
10
15
0
100
200
300

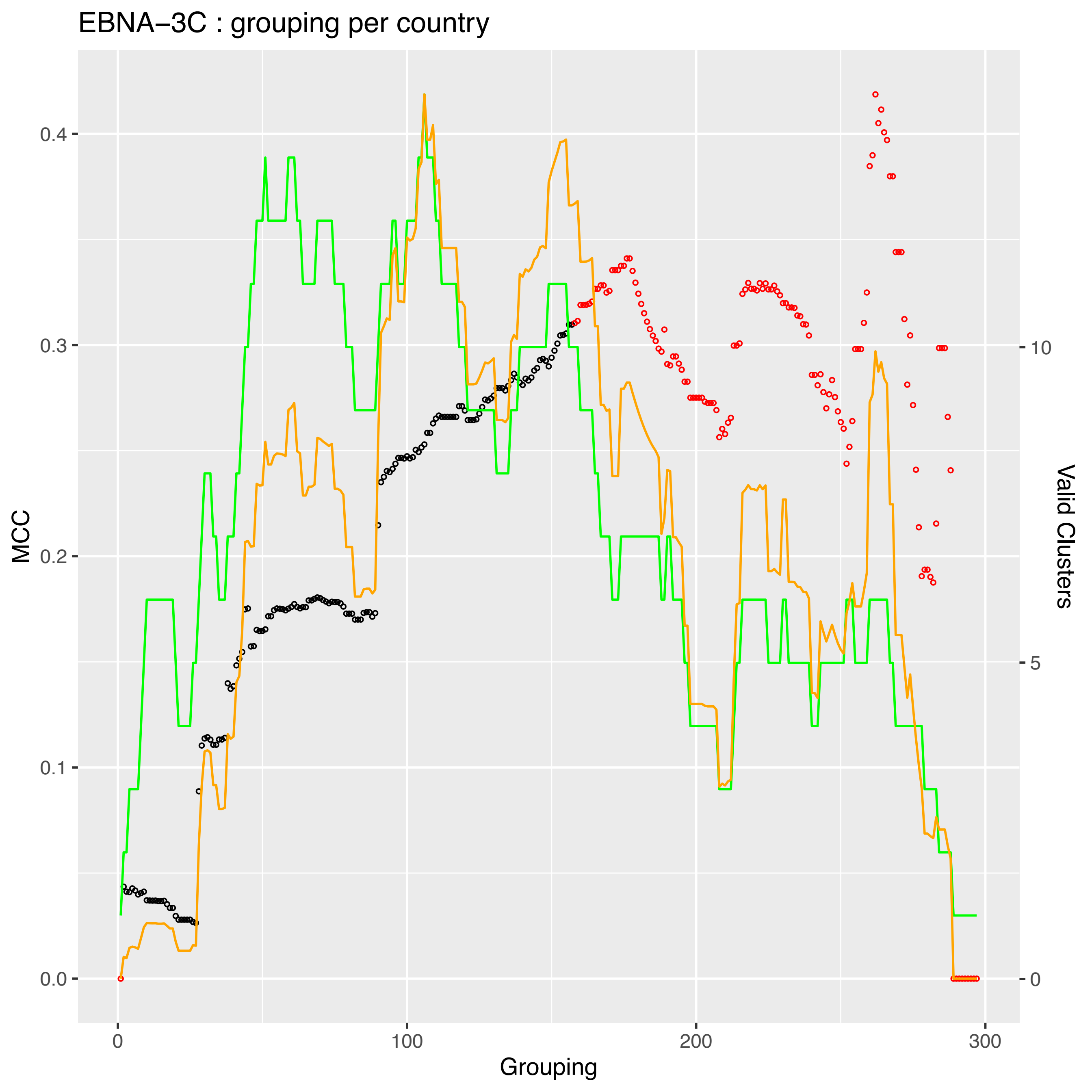
EBNA−3C : grouping per country
MCC
Valid Clusters
Grouping
0.0
0.1
0.2
0.3
0.4
0
100
200
300
0
5
10

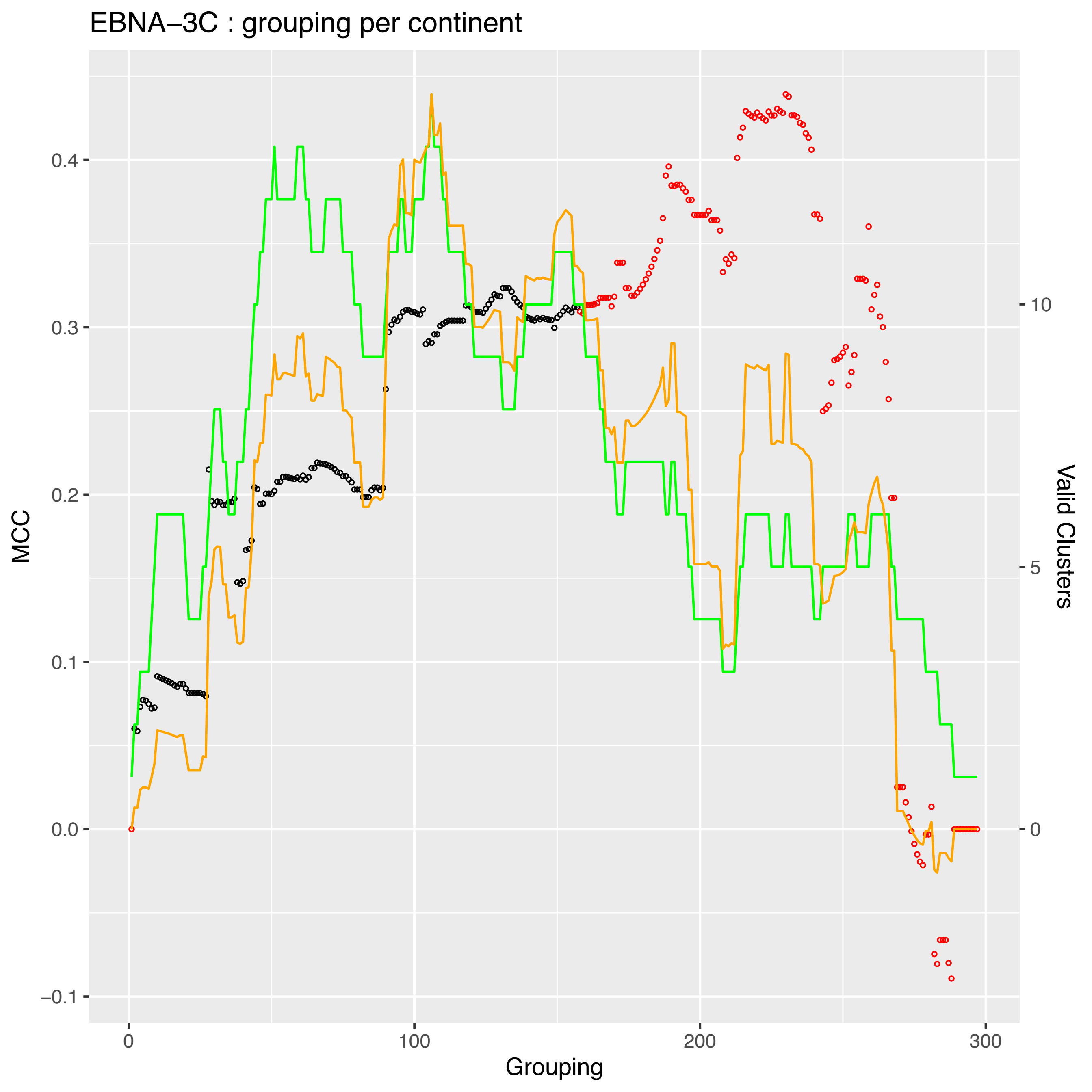

EBNA−3C : grouping per continent
MCC
Valid Clusters
Grouping
0.4
0.3
0.2
0.1
0.0
−0.1
0
100
200
300
10
5
0

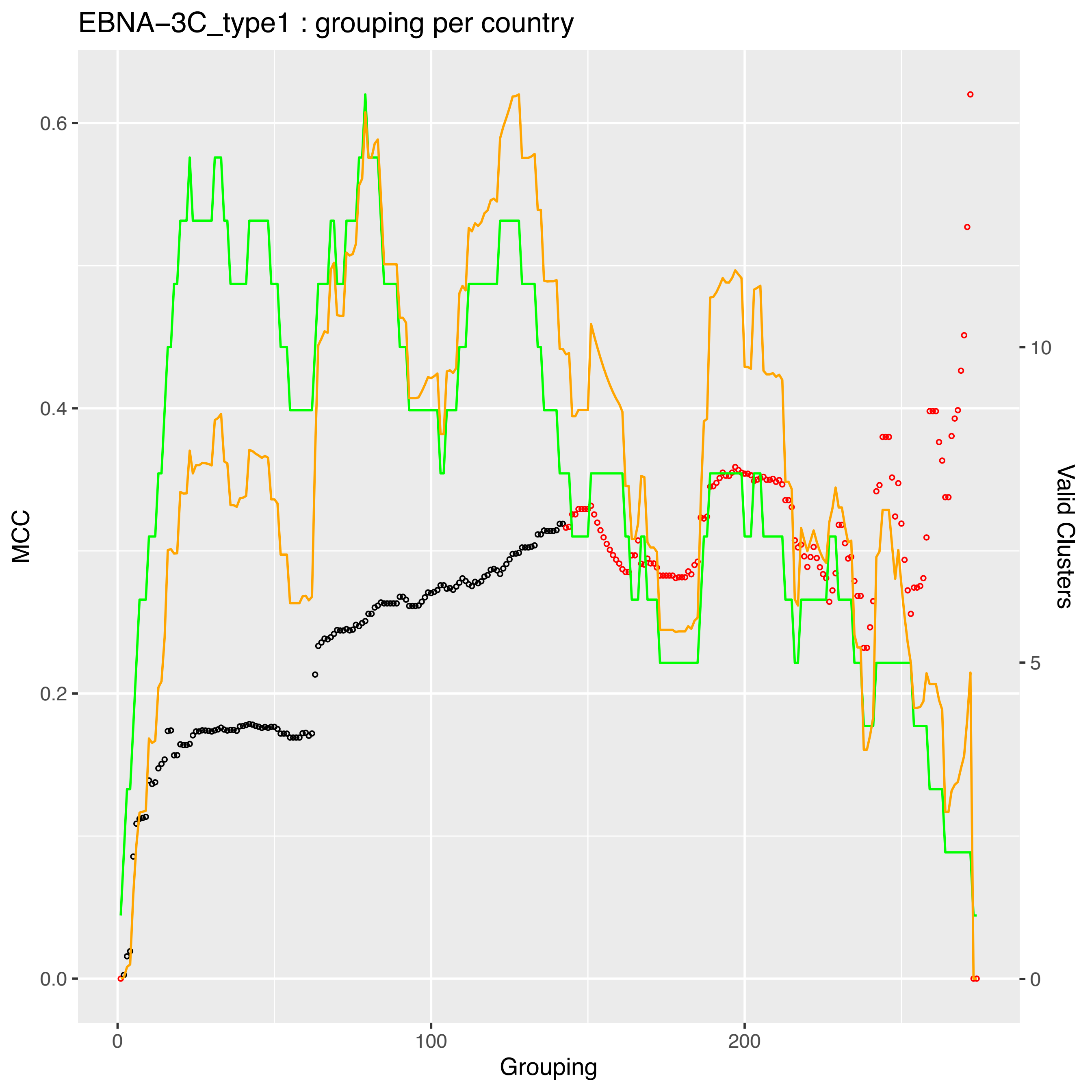

EBNA−3C_type1 : grouping per country
MCC
0.0
0.2
0.4
0.6
Valid Clusters
0
5
10
Grouping
0
100
200

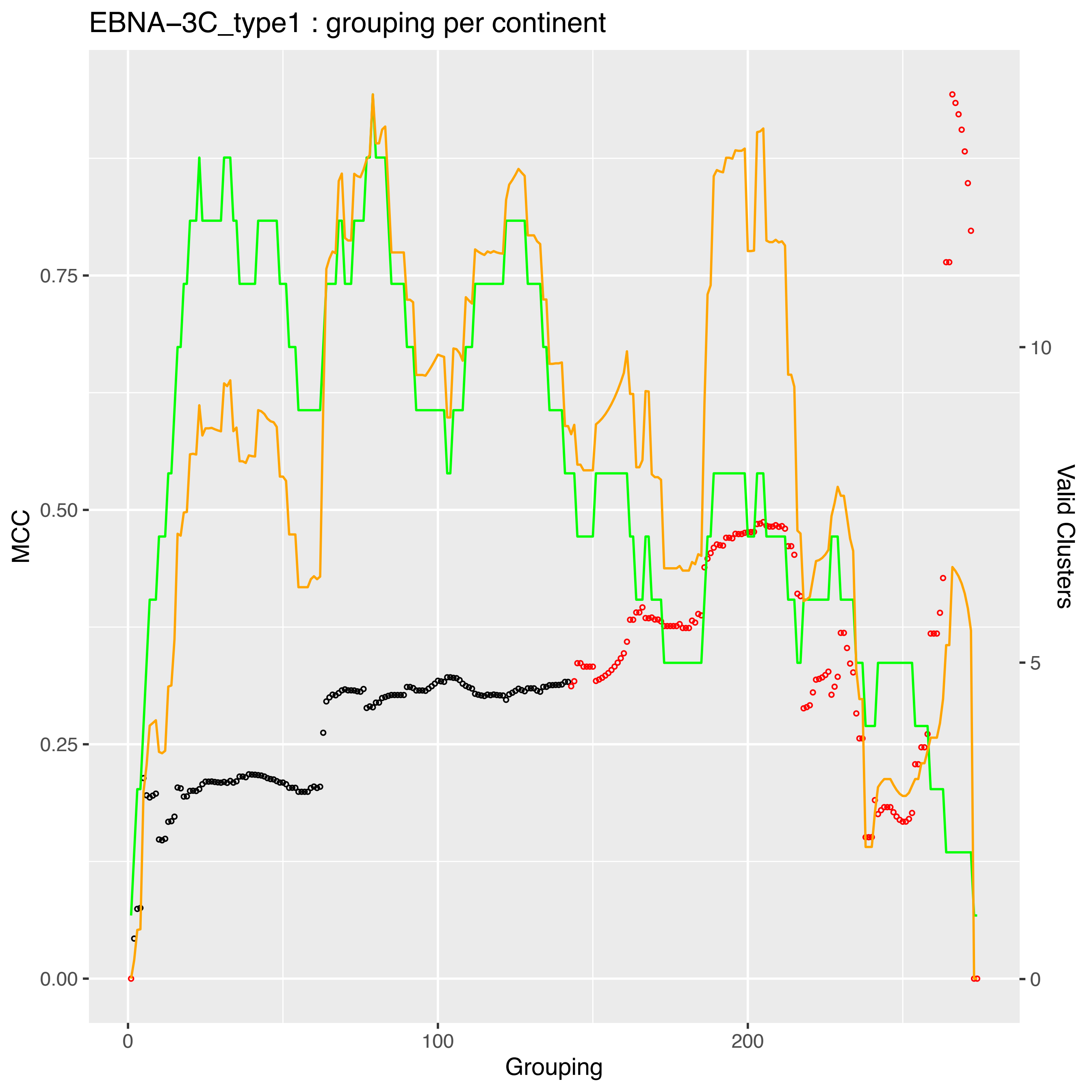

EBNA−3C_type1 : grouping per continent
MCC
0.00
0.25
0.50
0.75
Valid Clusters
0
5
10
Grouping
0
100
200

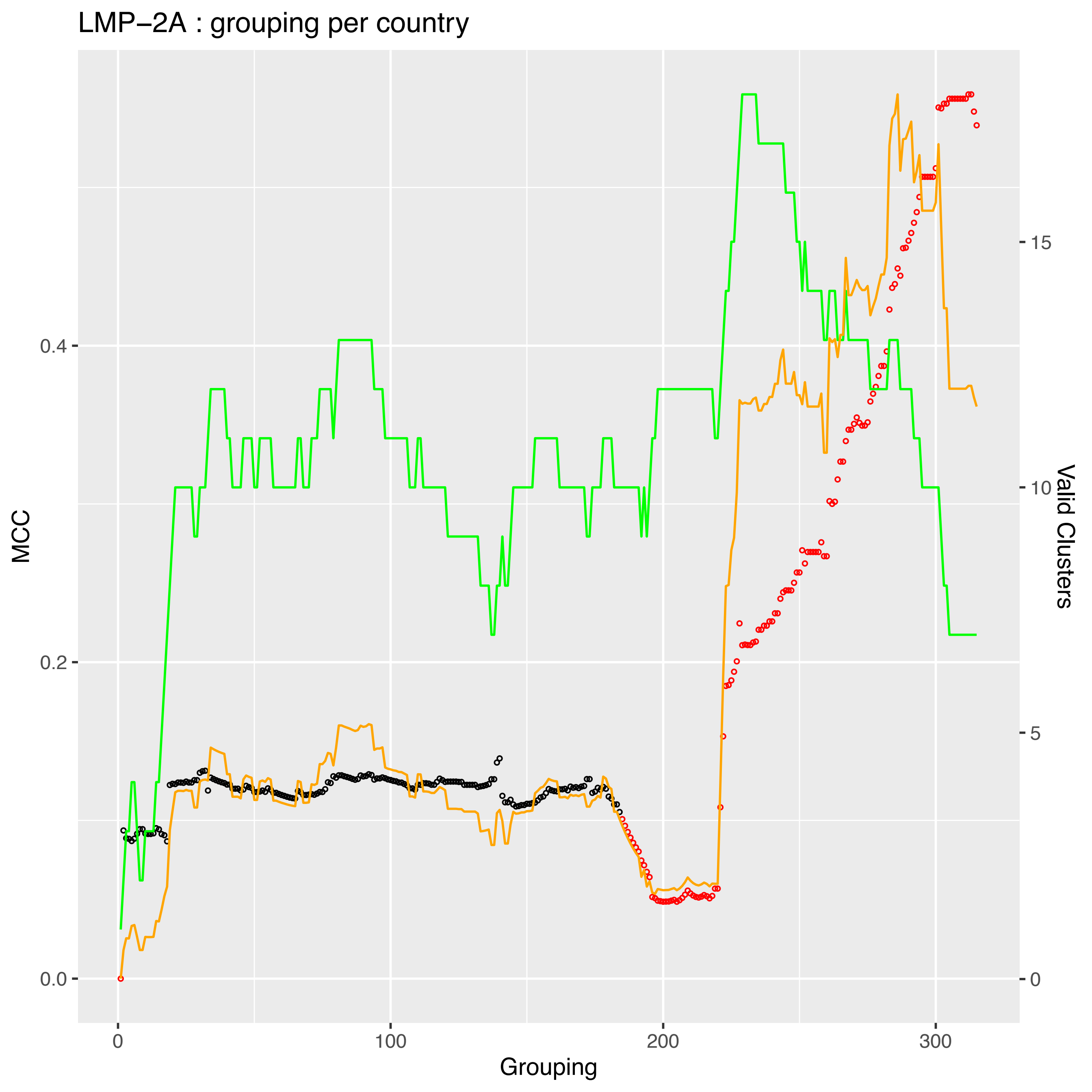

LMP−2A : grouping per country
MCC
0.0
0.2
0.4
Valid Clusters
0
5
10
15
Grouping
0
100
200
300

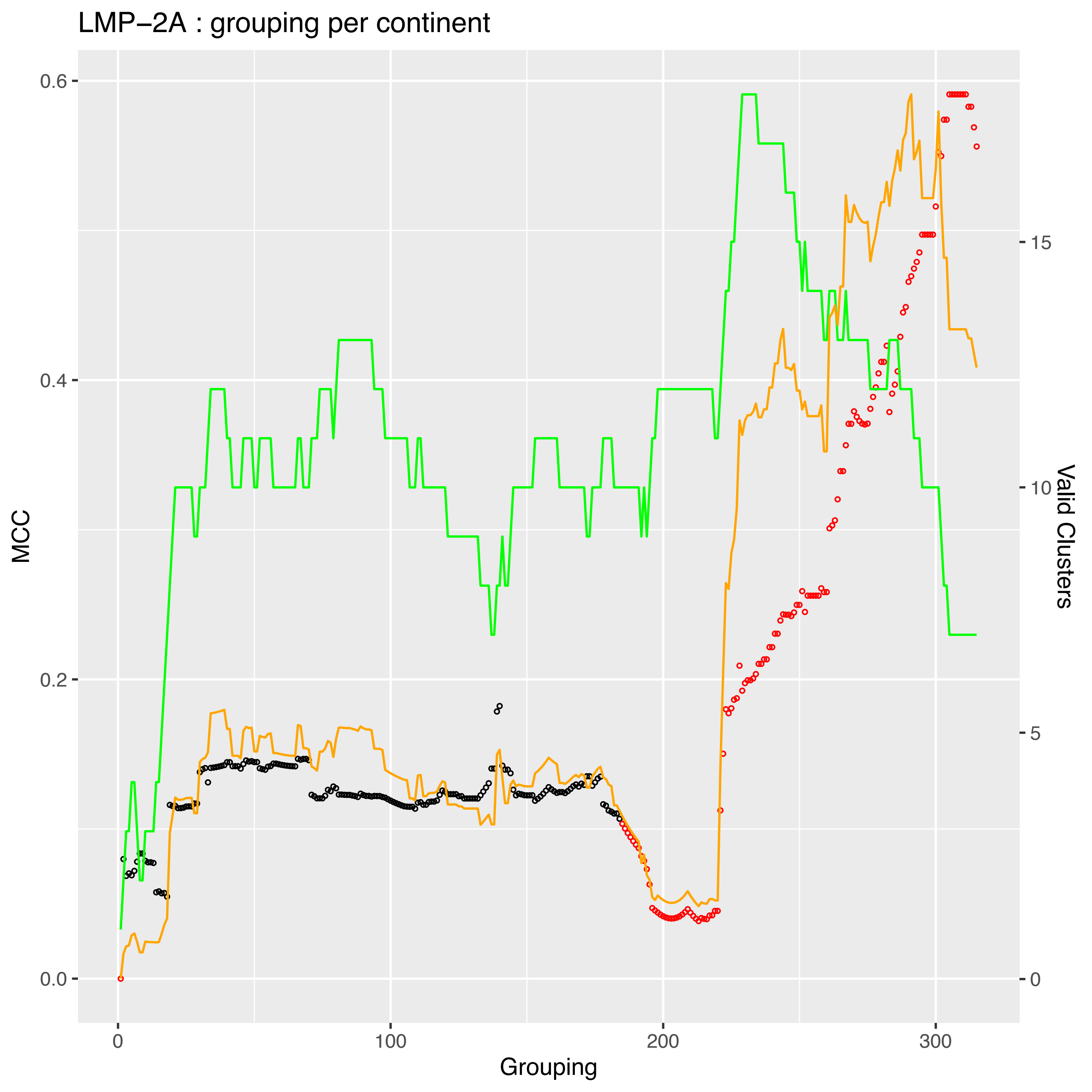
LMP−2A : grouping per continent
MCC
0.0
0.2
0.4
0.6
Valid Clusters
0
5
10
15
Grouping
0
100
200
300

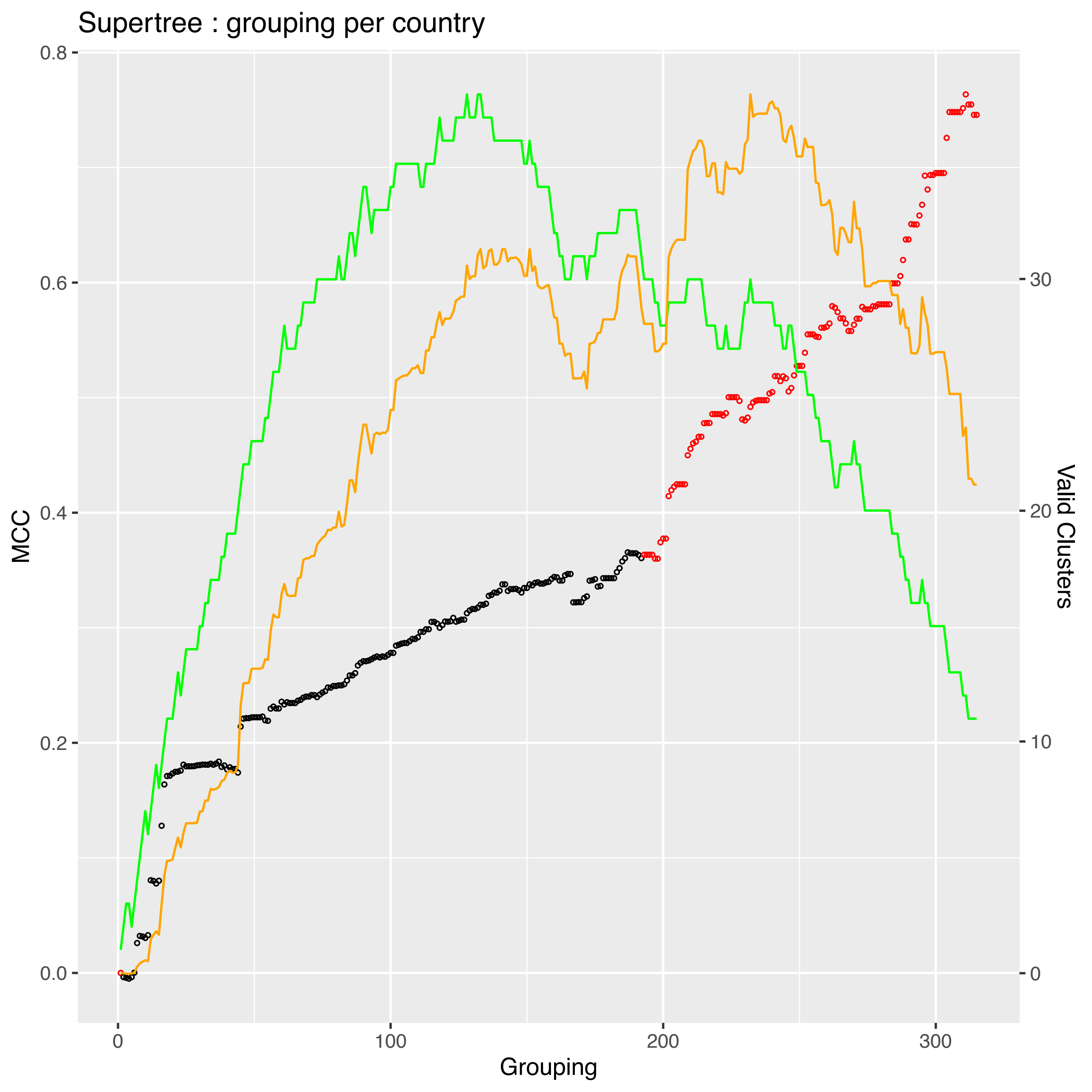

Supertree : grouping per country
MCC
Valid Clusters
Grouping
0.0
0.2
0.4
0.6
0.8
0
10
20
30
0
100
200
300

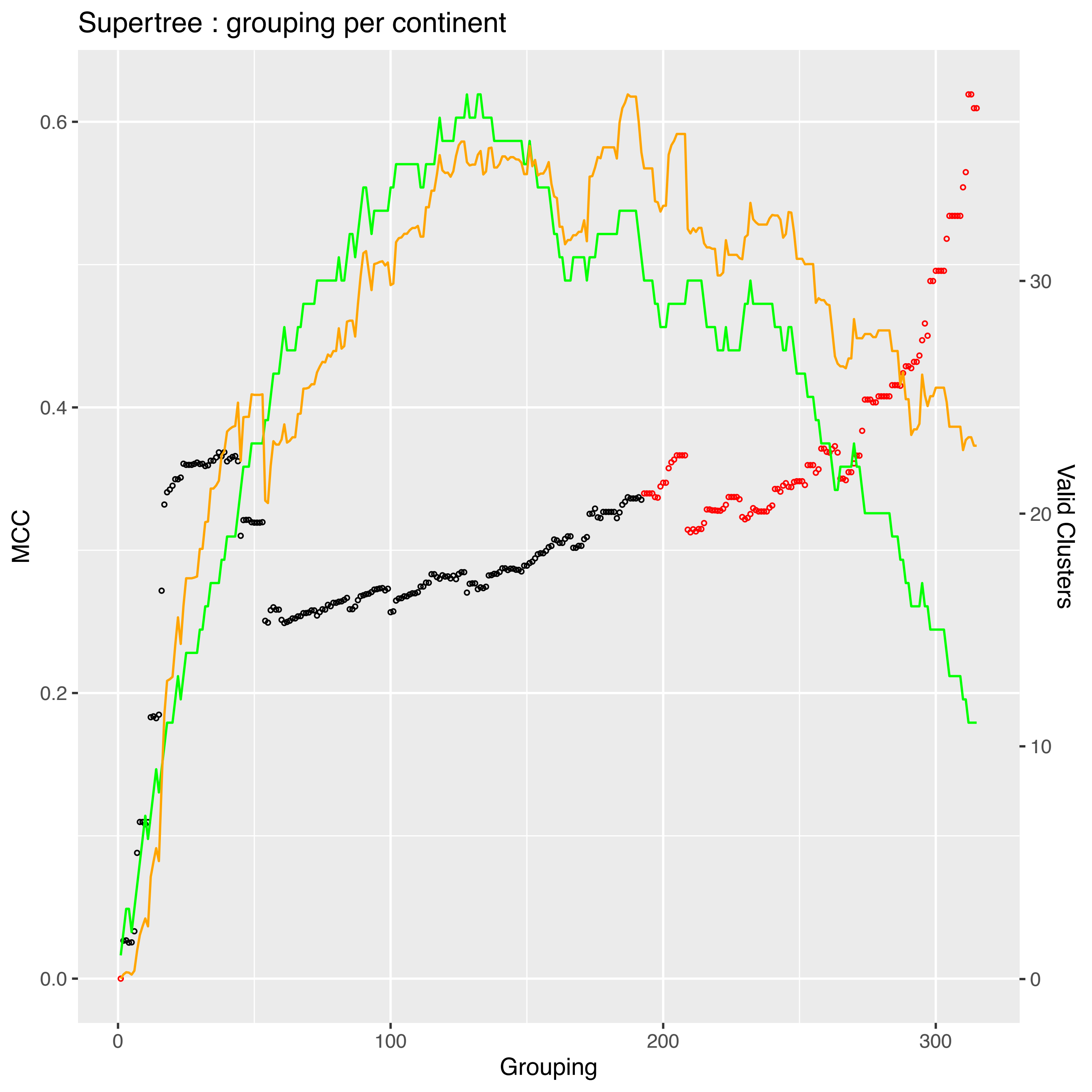
Supertree : grouping per continent
MCC
0.0
0.2
0.4
0.6
Valid Clusters
0
10
20
30
Grouping
0
100
200
300

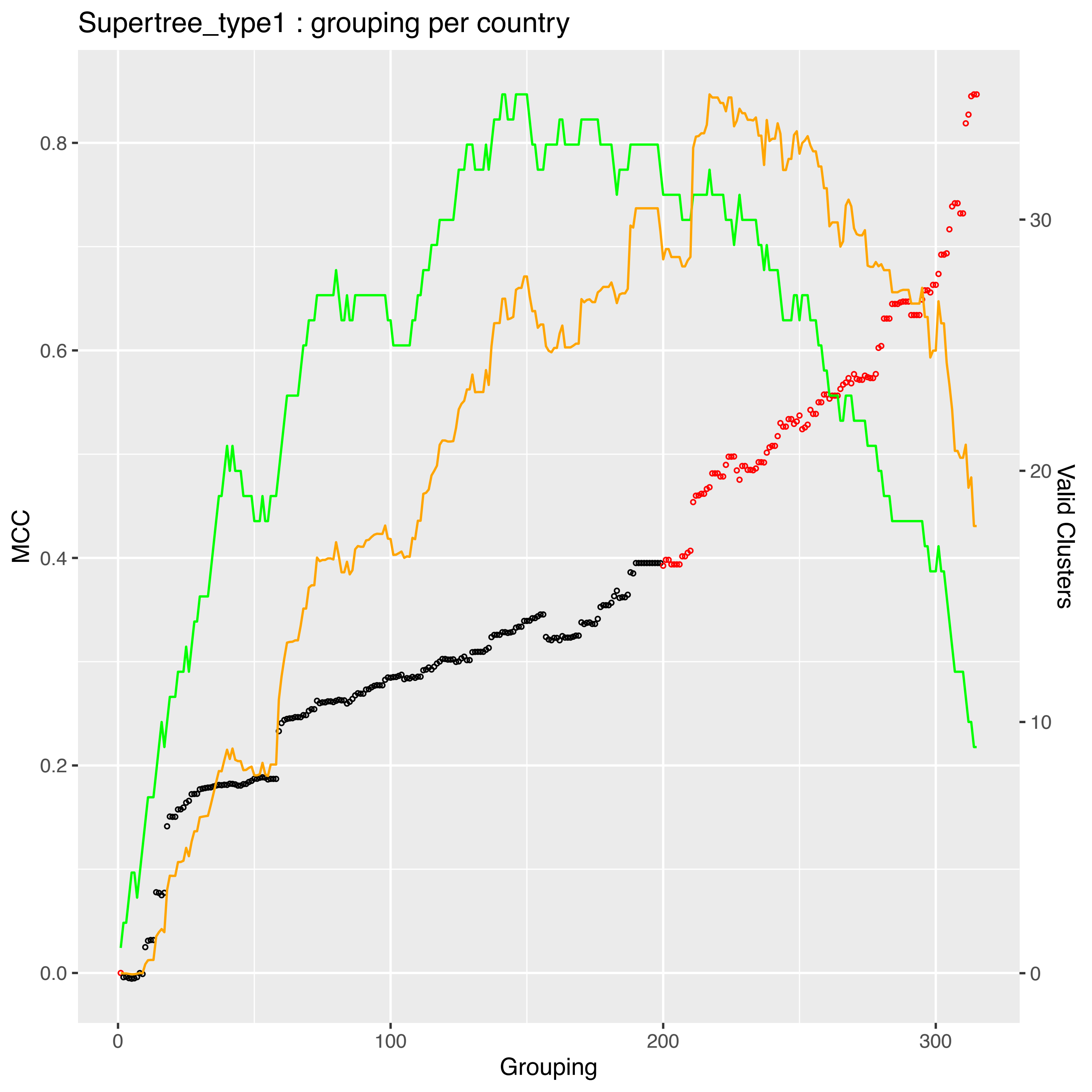

Supertree_type1 : grouping per country
MCC
0.0
0.2
0.4
0.6
0.8
Valid Clusters
0
10
20
30
Grouping
0
100
200
300

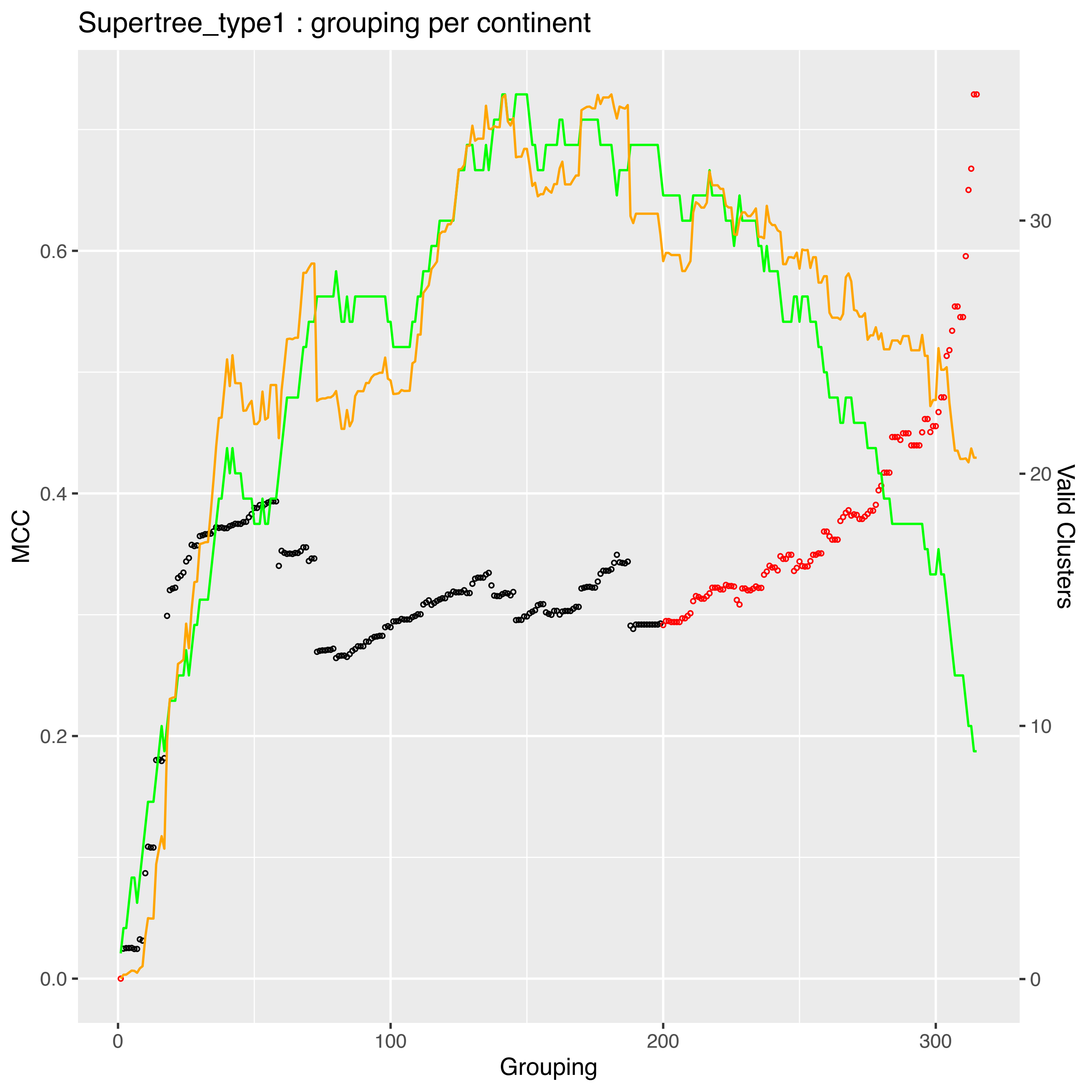
Supertree_type1 : grouping per continent
MCC
0.0
0.2
0.4
0.6
Valid Clusters
0
10
20
30
Grouping
0
100
200
300

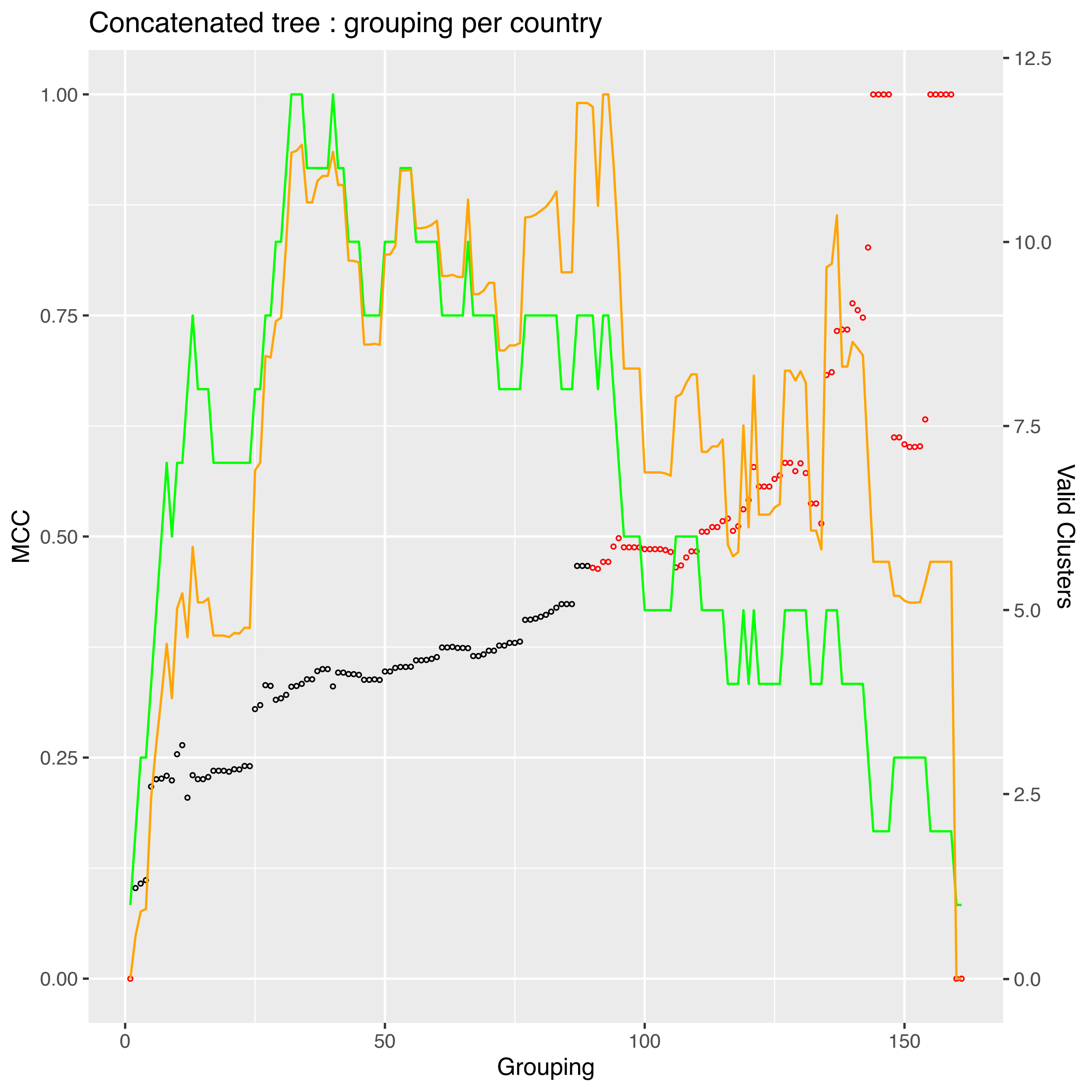

Concatenated tree : grouping per country
MCC
Valid Clusters
Grouping
1.00
0.75
0.50
0.25
0.00
12.5
10.0
7.5
5.0
2.5
0.0
0
50
100
150

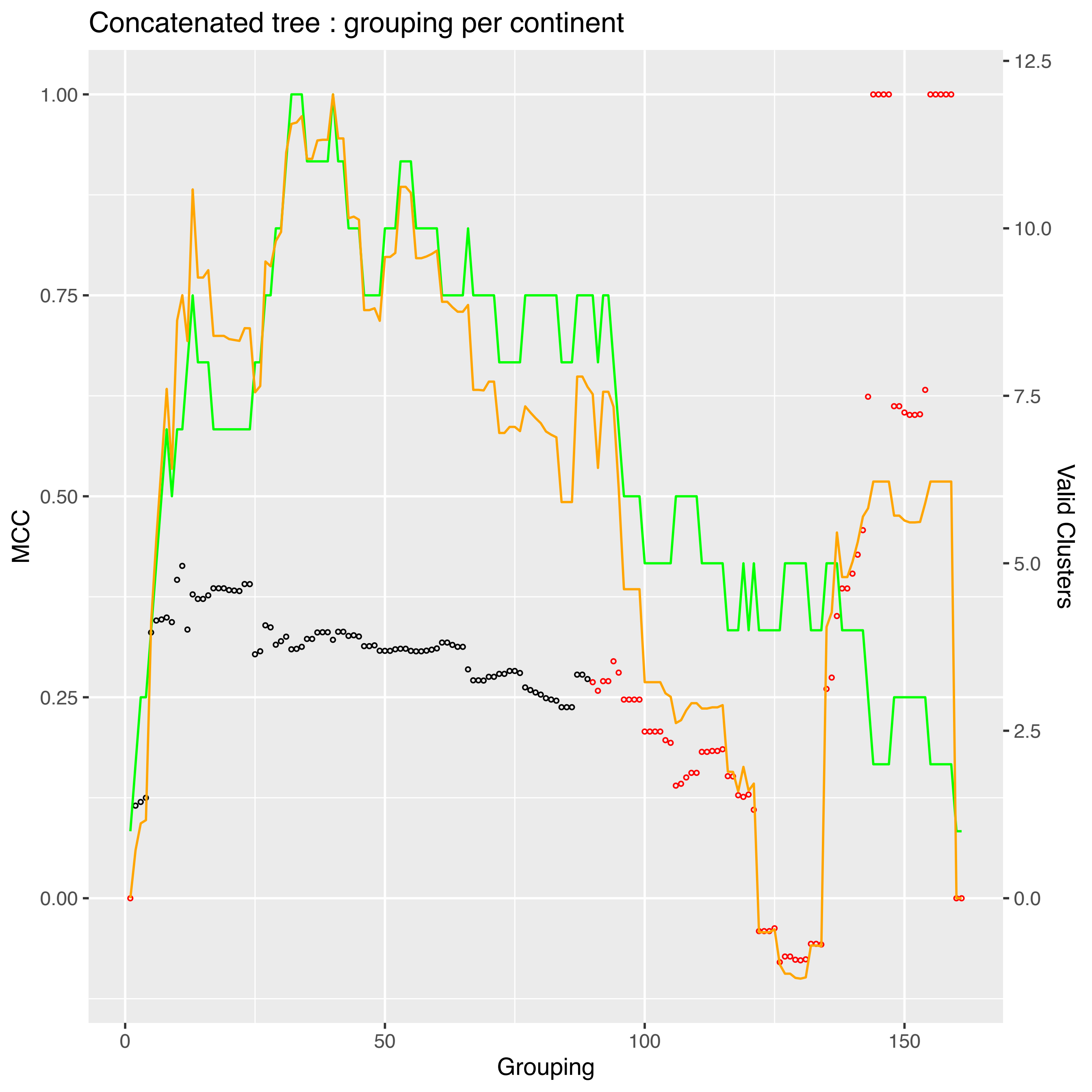

Concatenated tree : grouping per continent
MCC
Valid Clusters
Grouping
1.00
0.75
0.50
0.25
0.00
12.5
10.0
7.5
5.0
2.5
0.0
0
50
100
150

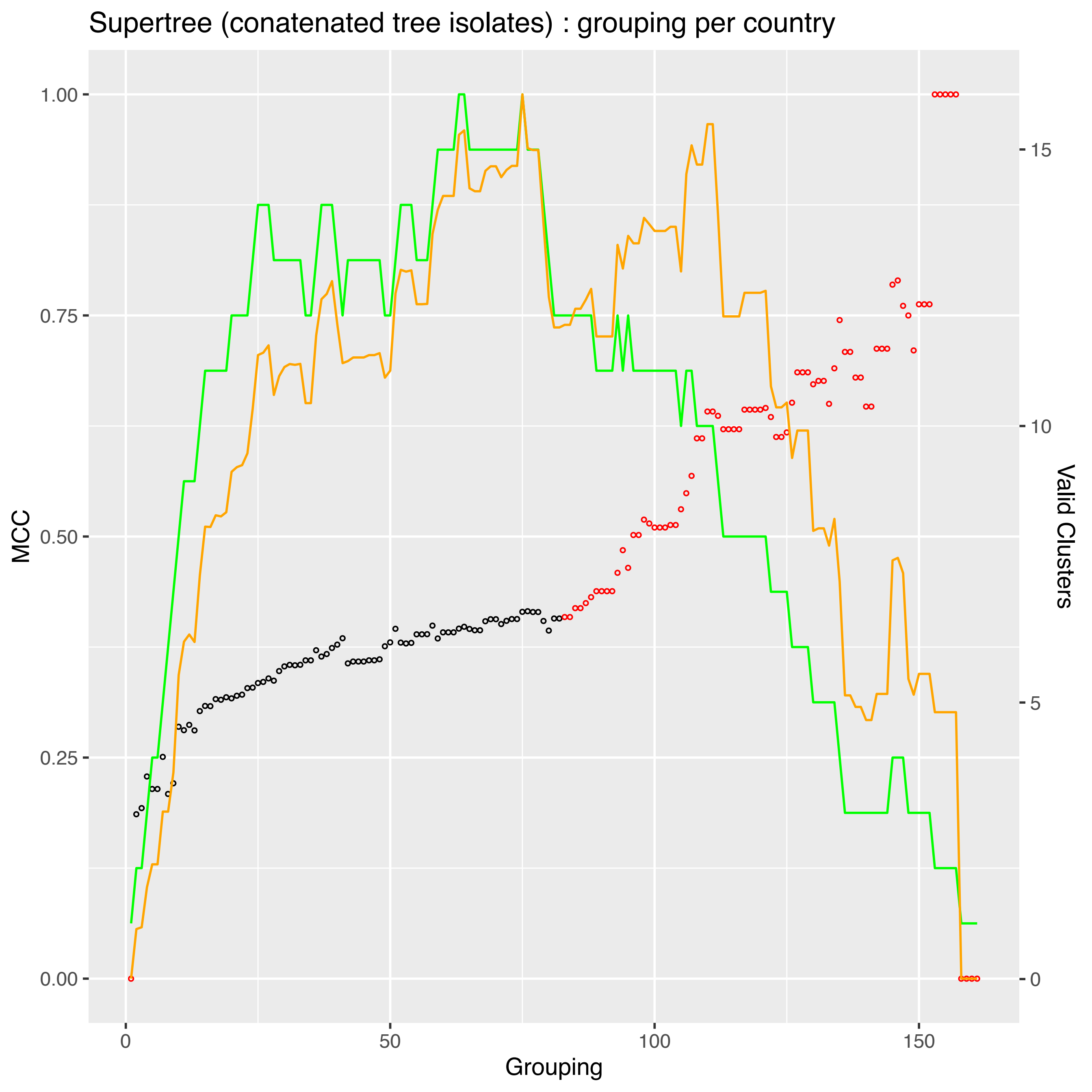
Supertree (conatenated tree isolates) : grouping per country
MCC
1.00
0.75
0.50
0.25
0.00
Valid Clusters
15
10
5
0
0
50
100
150
Grouping

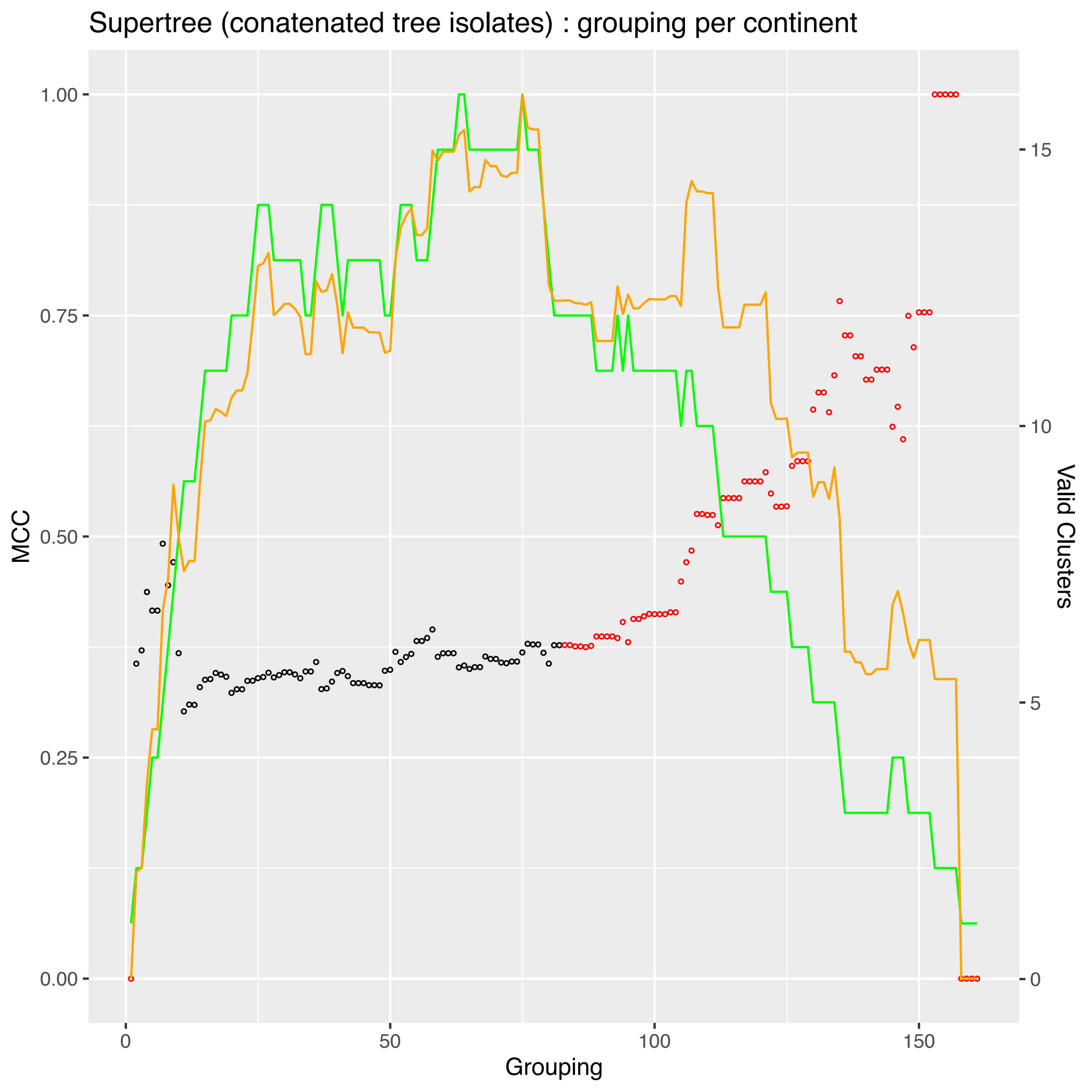

Supertree (conatenated tree isolates) : grouping per continent
MCC
1.00
0.75
0.50
0.25
0.00
Valid Clusters
15
10
5
0
0
50
100
150
Grouping

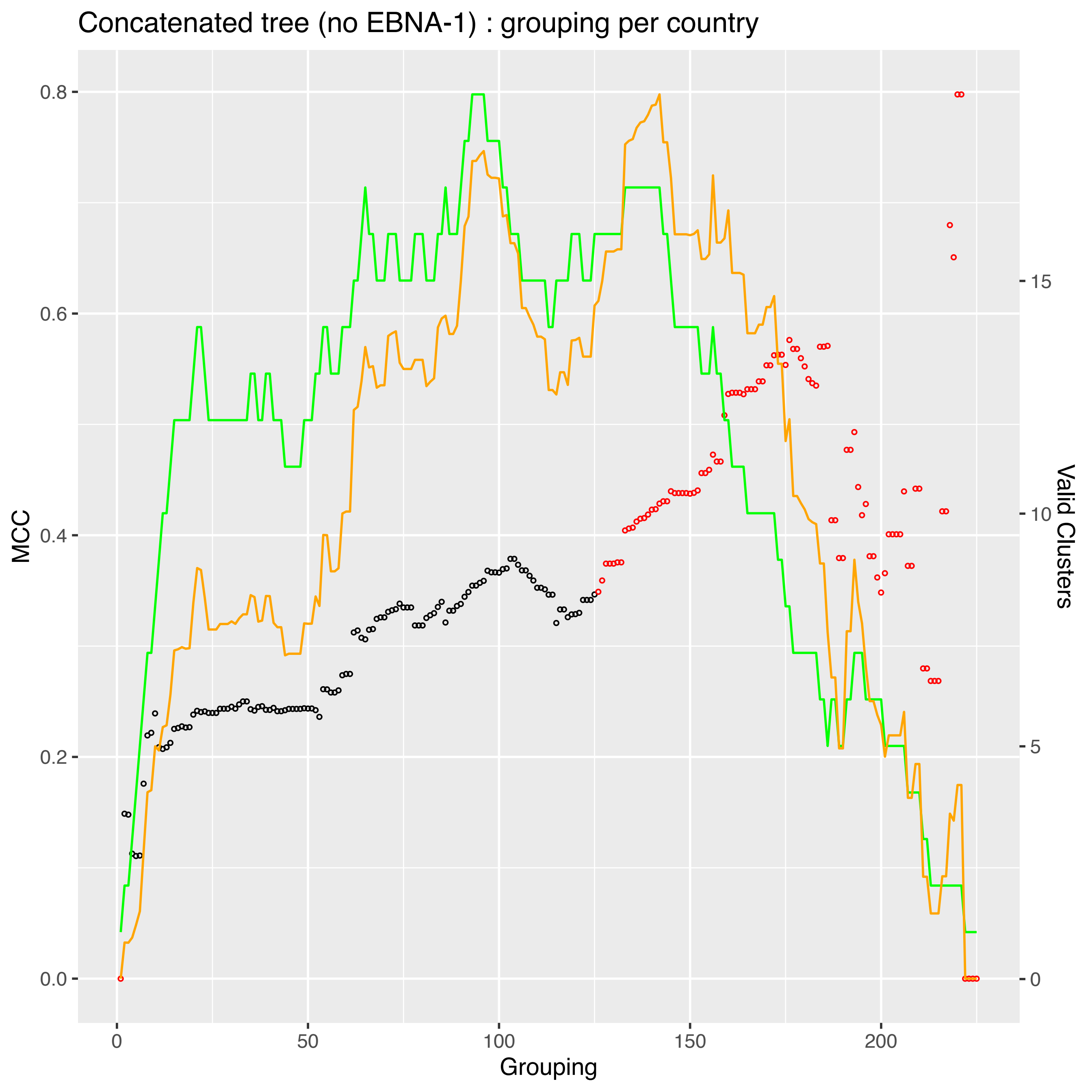

Concatenated tree (no EBNA-1) : grouping per country
MCC
0.0
0.2
0.4
0.6
0.8
Valid Clusters
0
5
10
15
Grouping
0
50
100
150
200

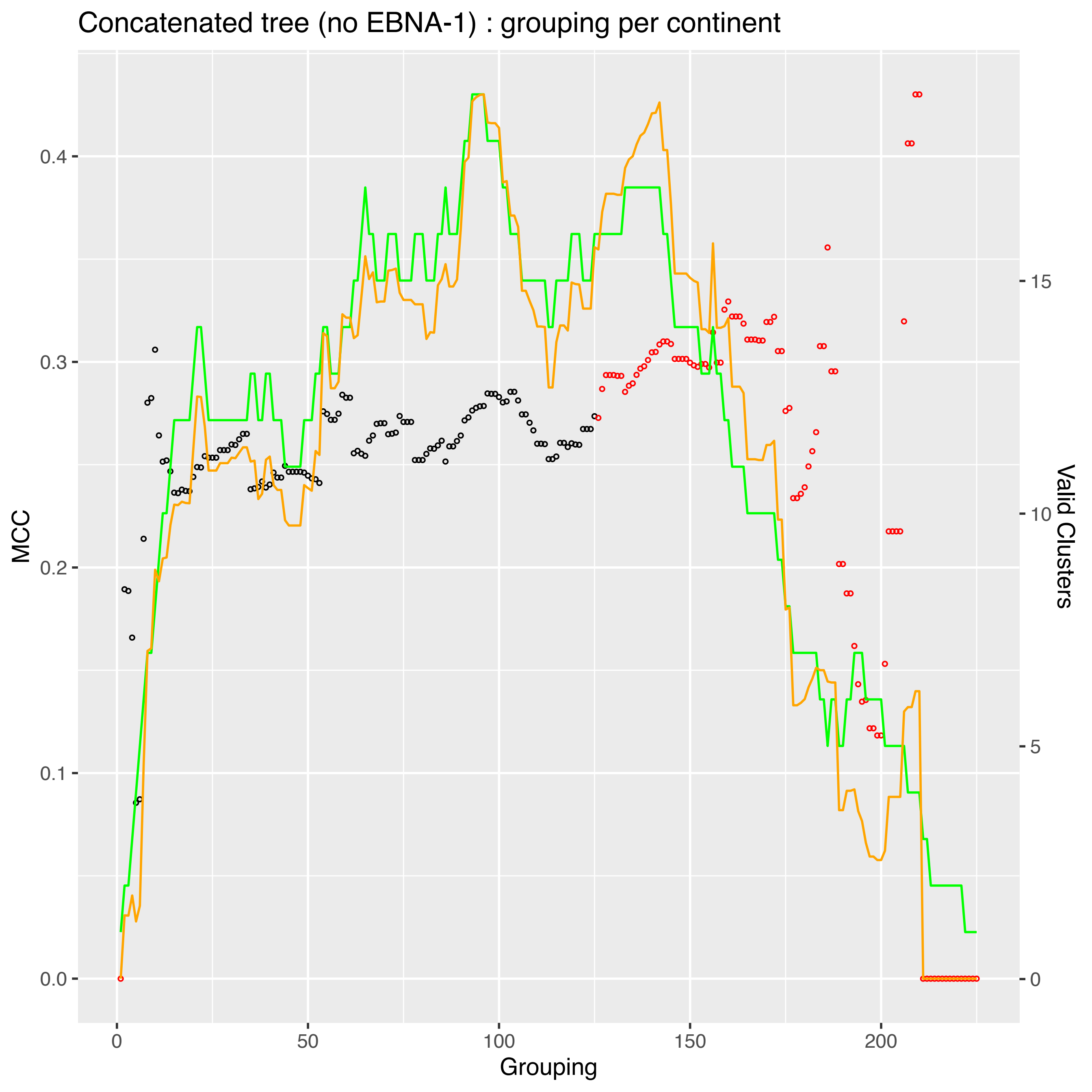

Concatenated tree (no EBNA-1) : grouping per continent
MCC
0.0
0.1
0.2
0.3
0.4
Valid Clusters
0
5
10
15
Grouping
0
50
100
150
200

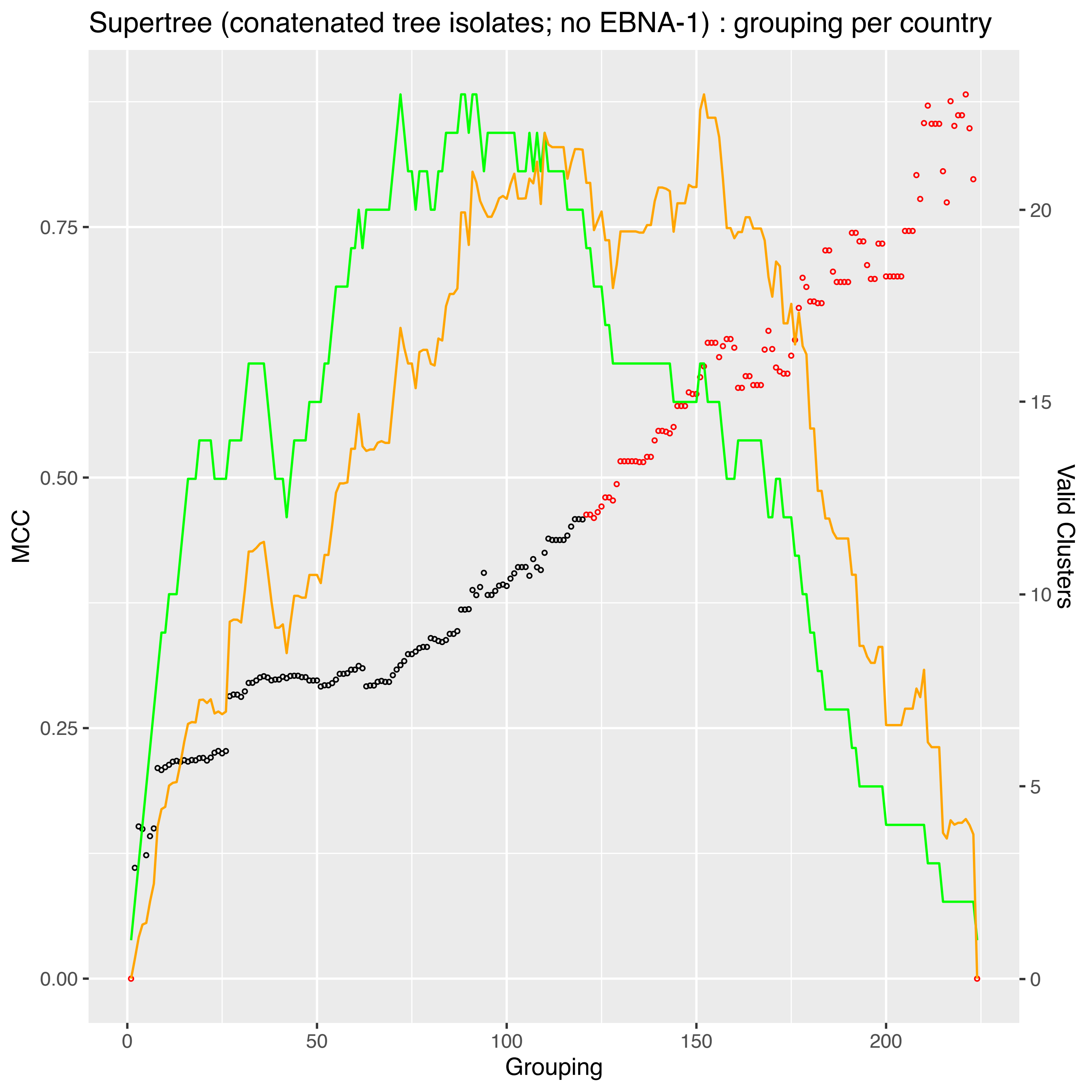

Supertree (conatenated tree isolates; no EBNA-1) : grouping per country
MCC
0.00
0.25
0.50
0.75
Valid Clusters
0
5
10
15
20
Grouping
0
50
100
150
200

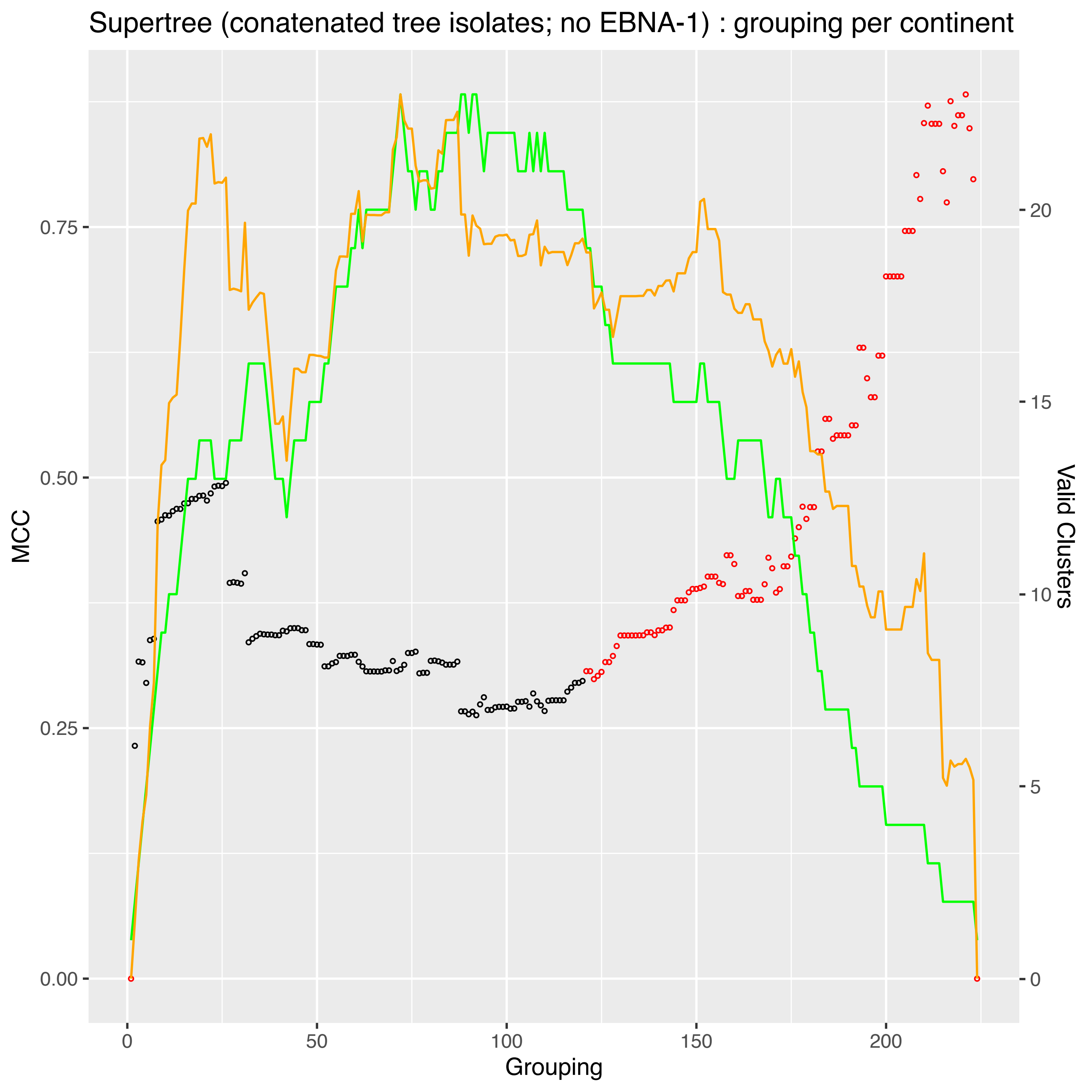

Supertree (conatenated tree isolates; no EBNA-1) : grouping per continent
MCC
Valid Clusters
Grouping
0.00
0.25
0.50
0.75
0
5
10
15
20
0
50
100
150
200

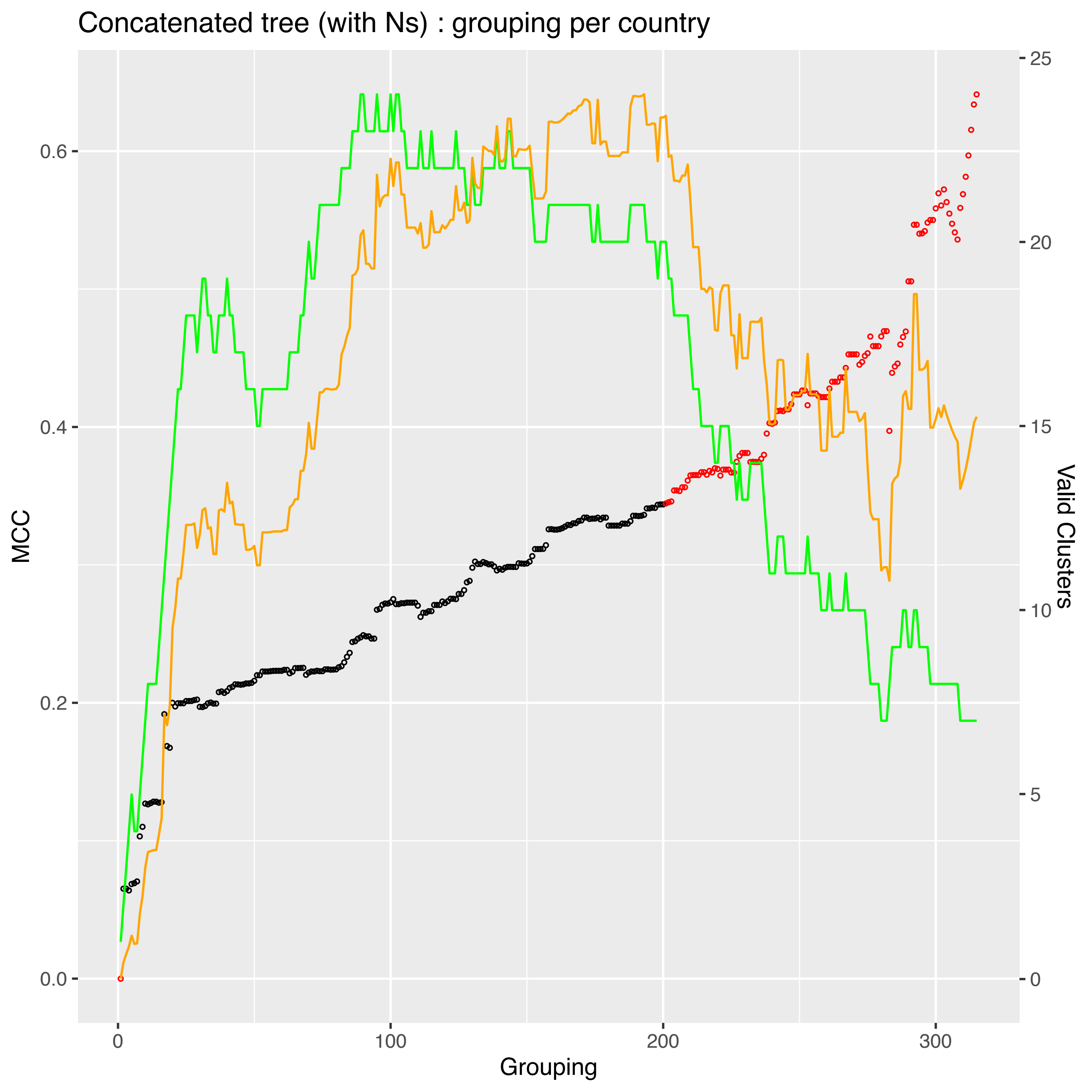
Concatenated tree (with Ns) : grouping per country
MCC
0.0
0.2
0.4
0.6
Valid Clusters
0
5
10
15
20
25
Grouping
0
100
200
300

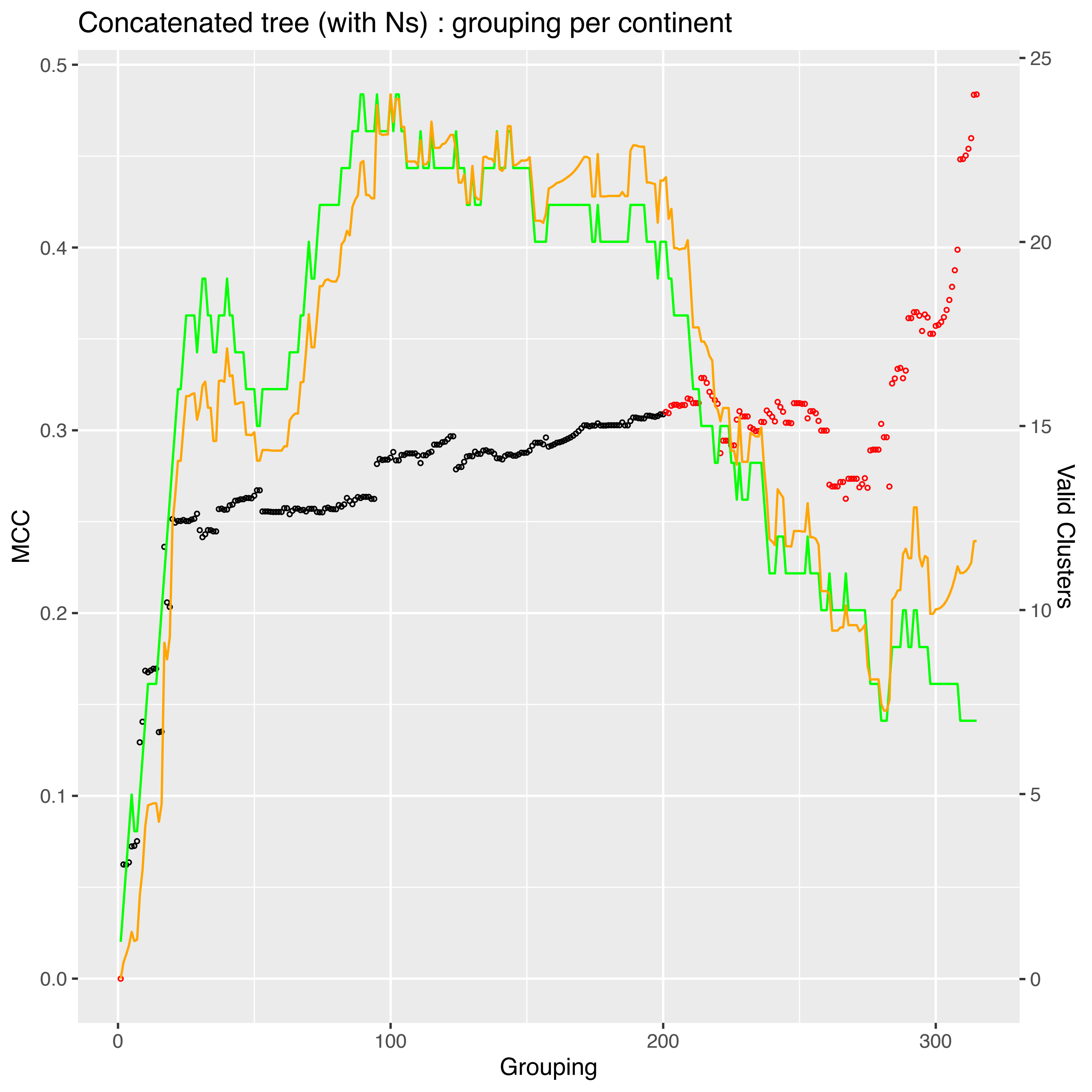

Concatenated tree (with Ns) : grouping per continent
MCC
0.5
0.4
0.3
0.2
0.1
0.0
Valid Clusters
25
20
15
10
5
0
Grouping
0
100
200
300

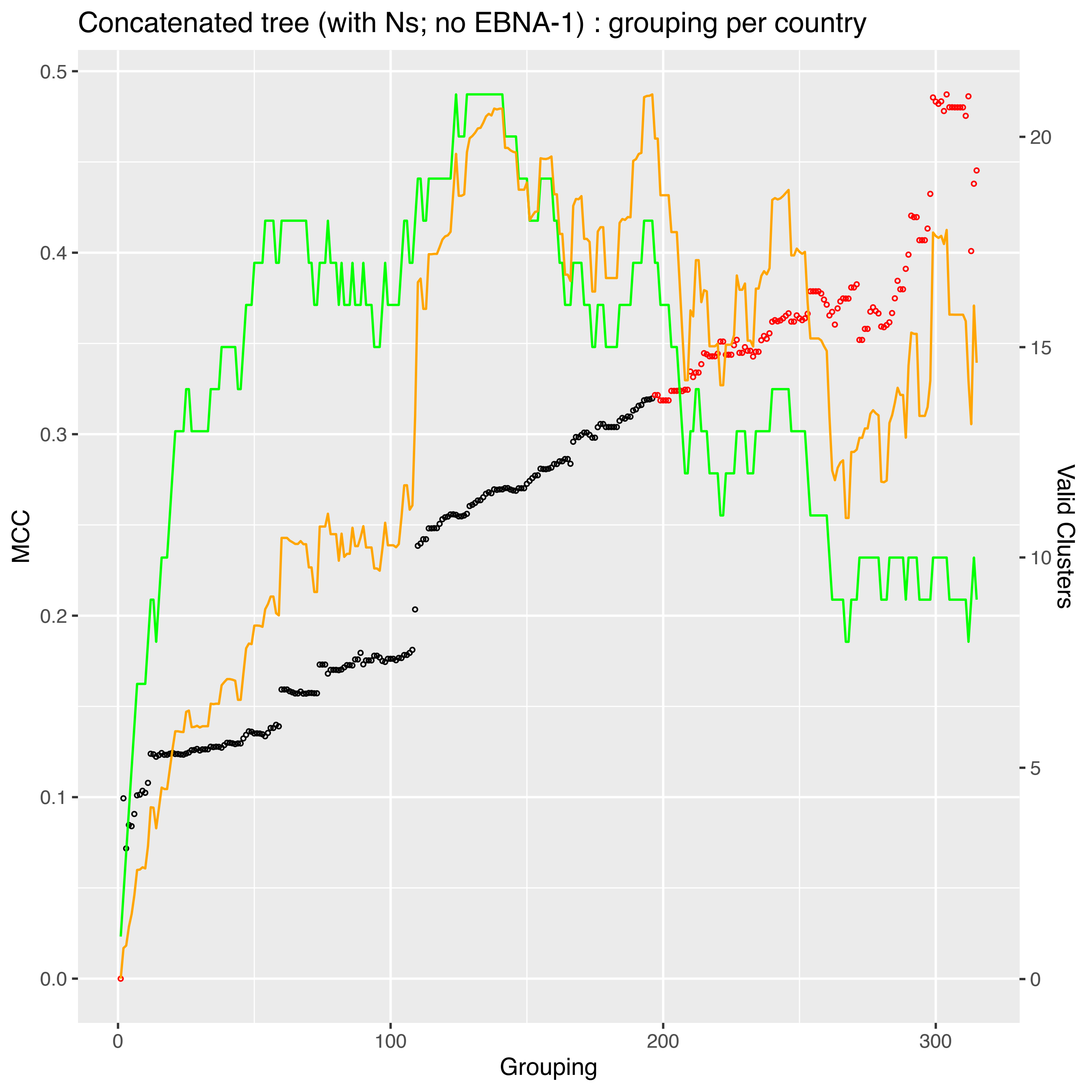

Concatenated tree (with Ns; no EBNA-1) : grouping per country
MCC
Valid Clusters
Grouping
0.0
0.1
0.2
0.3
0.4
0.5
0
5
10
15
20
0
100
200
300

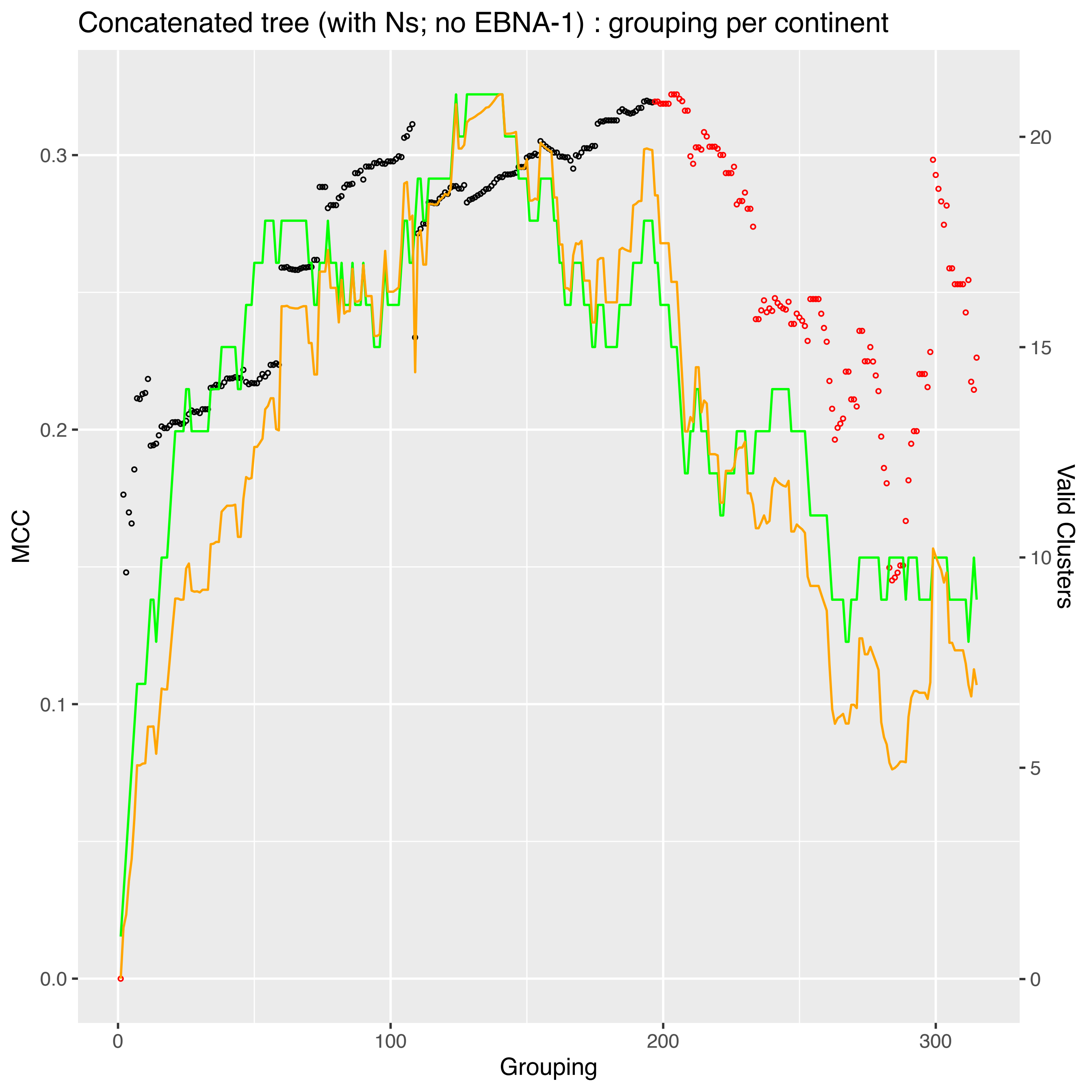
Concatenated tree (with Ns; no EBNA-1) : grouping per continent
MCC
Valid Clusters
Grouping
0.0
0.1
0.2
0.3
0
5
10
15
20
0
100
200
300
