# Supplementary material for: Expanding the Geographic Characterisation of Epstein–Barr Virus Variation through Gene-Based Approaches
Source: Microorganisms. 2020 Oct 29;8(11):1686. doi: 10.3390/microorganisms8111686 (PMC7692309; doi:10.3390/microorganisms8111686)
Supplement: Supplementary file 1 [file microorganisms-08-01686-s001.zip › Supplementary/Supplementary_Figure_7.pdf]

Supplementary Figure 7

Cluster/population associations: Heatmaps representing the odds ratios of clusters/population associations for each gene, and for both country and continent are shown. The cells are coloured based on the logarithmic value of the correspondent odds ratios, and the absolute values are reported. Significant cluster/population associations are marked by blue borders. Clusters with less than 3 isolates were excluded from the analysis.

The heatmaps were generated for both max CGS and max MCC cuts.

BDLF4 : grouping per country – max CGS

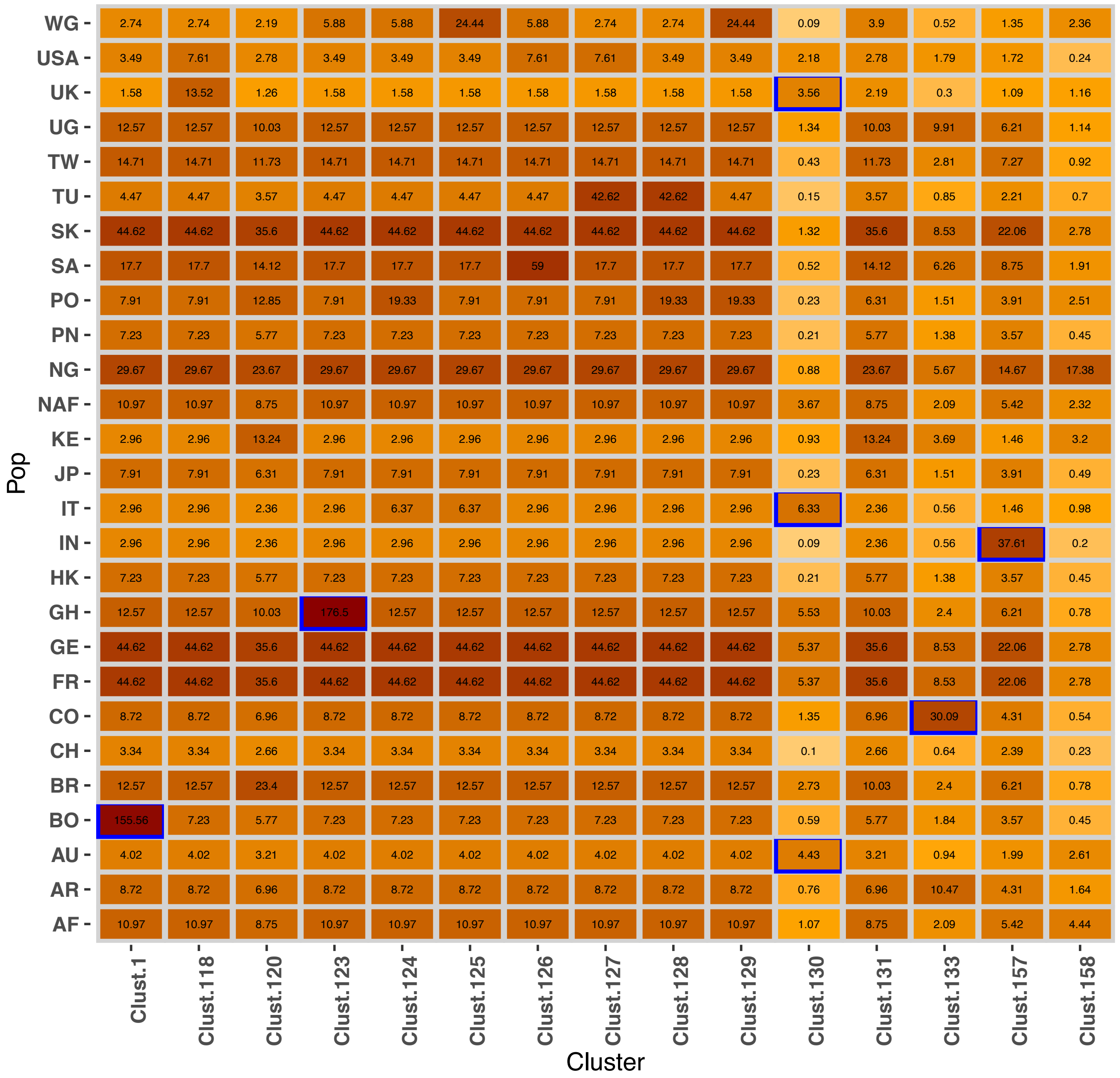

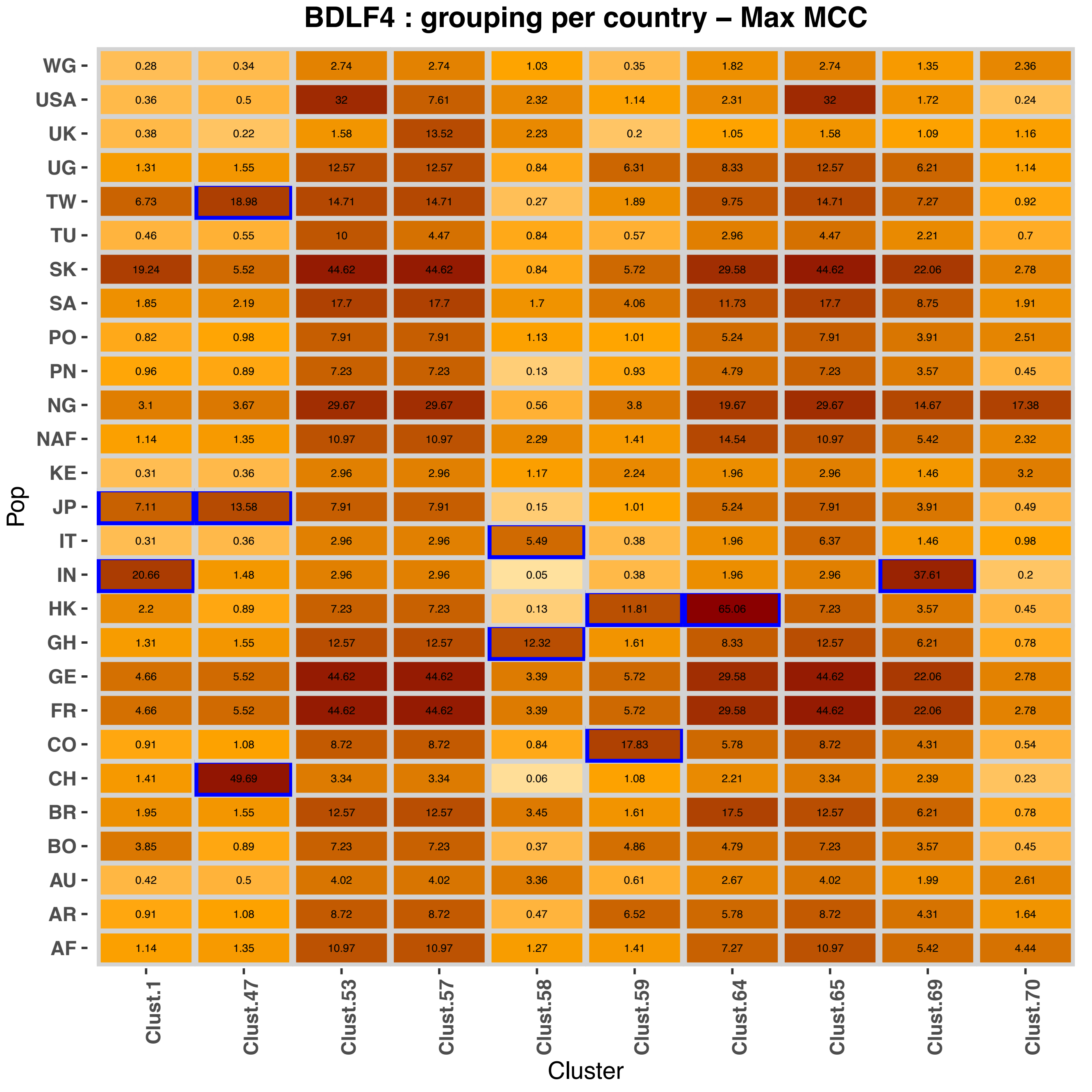
BDLF4 : grouping per country – Max MCC
Pop
Cluster
Clust.1 Clust.47 Clust.53 Clust.57 Clust.58 Clust.59 Clust.64 Clust.65 Clust.69 Clust.70
WG 0.28 0.34 2.74 2.74 1.03 0.35 1.82 2.74 1.35 2.36
USA 0.36 0.5 32 7.61 2.32 1.14 2.31 32 1.72 0.24
UK 0.38 0.22 1.58 13.52 2.23 0.2 1.05 1.58 1.09 1.16
UG 1.31 1.55 12.57 12.57 0.84 6.31 8.33 12.57 6.21 1.14
TW 6.73 18.98 14.71 14.71 0.27 1.89 9.75 14.71 7.27 0.92
TU 0.46 0.55 10 4.47 0.84 0.57 2.96 4.47 2.21 0.7
SK 19.24 5.52 44.62 44.62 0.84 5.72 29.58 44.62 22.06 2.78
SA 1.85 2.19 17.7 17.7 1.7 4.06 11.73 17.7 8.75 1.91
PO 0.82 0.98 7.91 7.91 1.13 1.01 5.24 7.91 3.91 2.51
PN 0.96 0.89 7.23 7.23 0.13 0.93 4.79 7.23 3.57 0.45
NG 3.1 3.67 29.67 29.67 0.56 3.8 19.67 29.67 14.67 17.38
NAF 1.14 1.35 10.97 10.97 2.29 1.41 14.54 10.97 5.42 2.32
KE 0.31 0.36 2.96 2.96 1.17 2.24 1.96 2.96 1.46 3.2
JP 7.11 13.58 7.91 7.91 0.15 1.01 5.24 7.91 3.91 0.49
IT 0.31 0.36 2.96 2.96 5.49 0.38 1.96 6.37 1.46 0.98
IN 20.66 1.48 2.96 2.96 0.05 0.38 1.96 2.96 37.61 0.2
HK 2.2 0.89 7.23 7.23 0.13 11.81 65.06 7.23 3.57 0.45
GH 1.31 1.55 12.57 12.57 12.32 1.61 8.33 12.57 6.21 0.78
GE 4.66 5.52 44.62 44.62 3.39 5.72 29.58 44.62 22.06 2.78
FR 4.66 5.52 44.62 44.62 3.39 5.72 29.58 44.62 22.06 2.78
CO 0.91 1.08 8.72 8.72 0.84 17.83 5.78 8.72 4.31 0.54
CH 1.41 49.69 3.34 3.34 0.06 1.08 2.21 3.34 2.39 0.23
BR 1.95 1.55 12.57 12.57 3.45 1.61 17.5 12.57 6.21 0.78
BO 3.85 0.89 7.23 7.23 0.37 4.86 4.79 7.23 3.57 0.45
AU 0.42 0.5 4.02 4.02 3.36 0.61 2.67 4.02 1.99 2.61
AR 0.91 1.08 8.72 8.72 0.47 6.52 5.78 8.72 4.31 1.64
AF 1.14 1.35 10.97 10.97 1.27 1.41 7.27 10.97 5.42 4.44

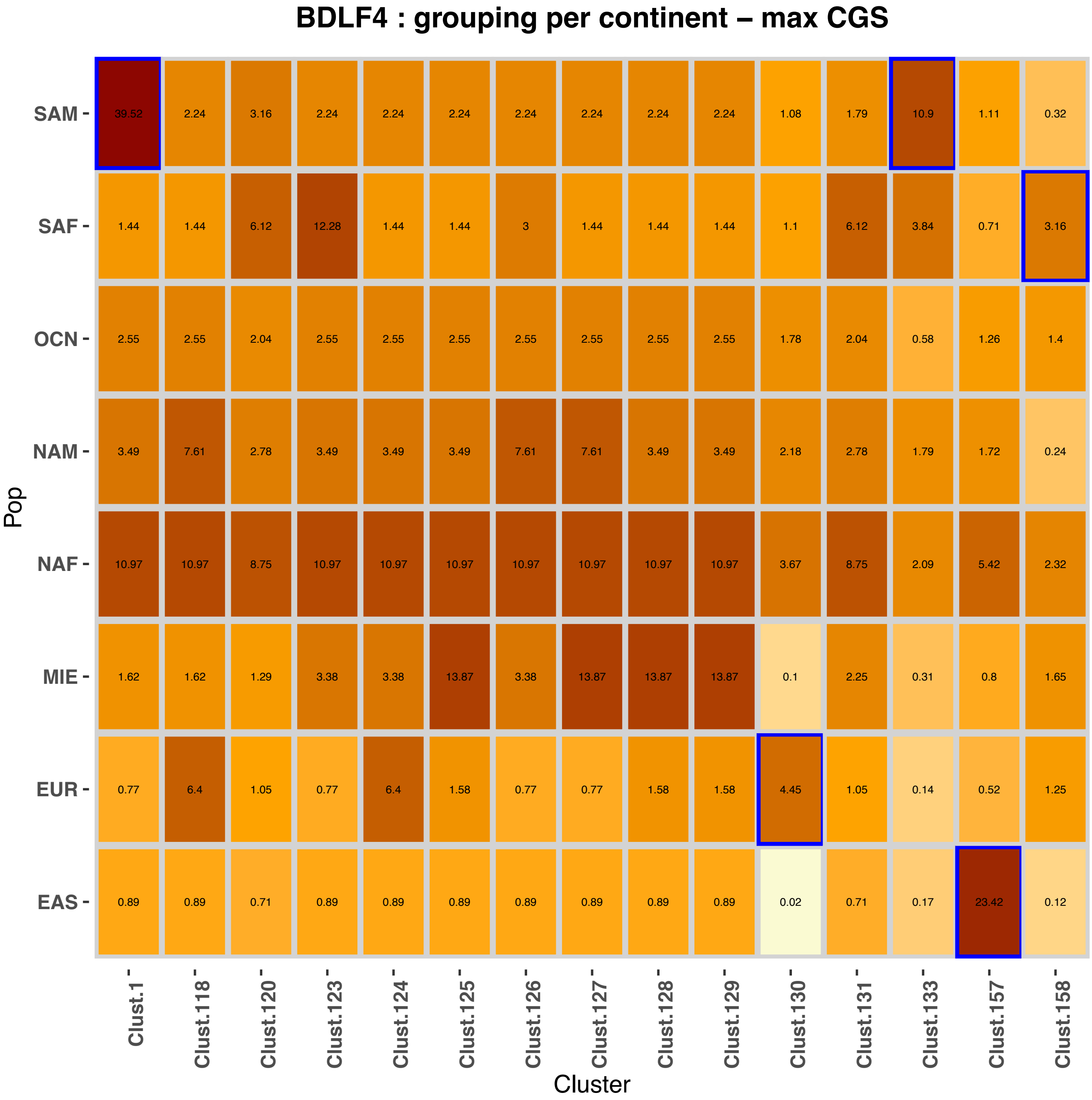
BDLF4 : grouping per continent – max CGS
Pop
Cluster
SAM
SAF
OCN
NAM
NAF
MIE
EUR
EAS
Clust.1
Clust.118
Clust.120
Clust.123
Clust.124
Clust.125
Clust.126
Clust.127
Clust.128
Clust.129
Clust.130
Clust.131
Clust.133
Clust.157
Clust.158
39.52 2.24 3.16 2.24 2.24 2.24 2.24 2.24 2.24 2.24 1.08 1.79 10.9 1.11 0.32
1.44 1.44 6.12 12.28 1.44 1.44 3 1.44 1.44 1.44 1.1 6.12 3.84 0.71 3.16
2.55 2.55 2.04 2.55 2.55 2.55 2.55 2.55 2.55 2.55 1.78 2.04 0.58 1.26 1.4
3.49 7.61 2.78 3.49 3.49 3.49 7.61 7.61 3.49 3.49 2.18 2.78 1.79 1.72 0.24
10.97 10.97 8.75 10.97 10.97 10.97 10.97 10.97 10.97 10.97 3.67 8.75 2.09 5.42 2.32
1.62 1.62 1.29 3.38 3.38 13.87 3.38 13.87 13.87 13.87 0.1 2.25 0.31 0.8 1.65
0.77 6.4 1.05 0.77 6.4 1.58 0.77 0.77 1.58 1.58 4.45 1.05 0.14 0.52 1.25
0.89 0.89 0.71 0.89 0.89 0.89 0.89 0.89 0.89 0.89 0.02 0.71 0.17 23.42 0.12

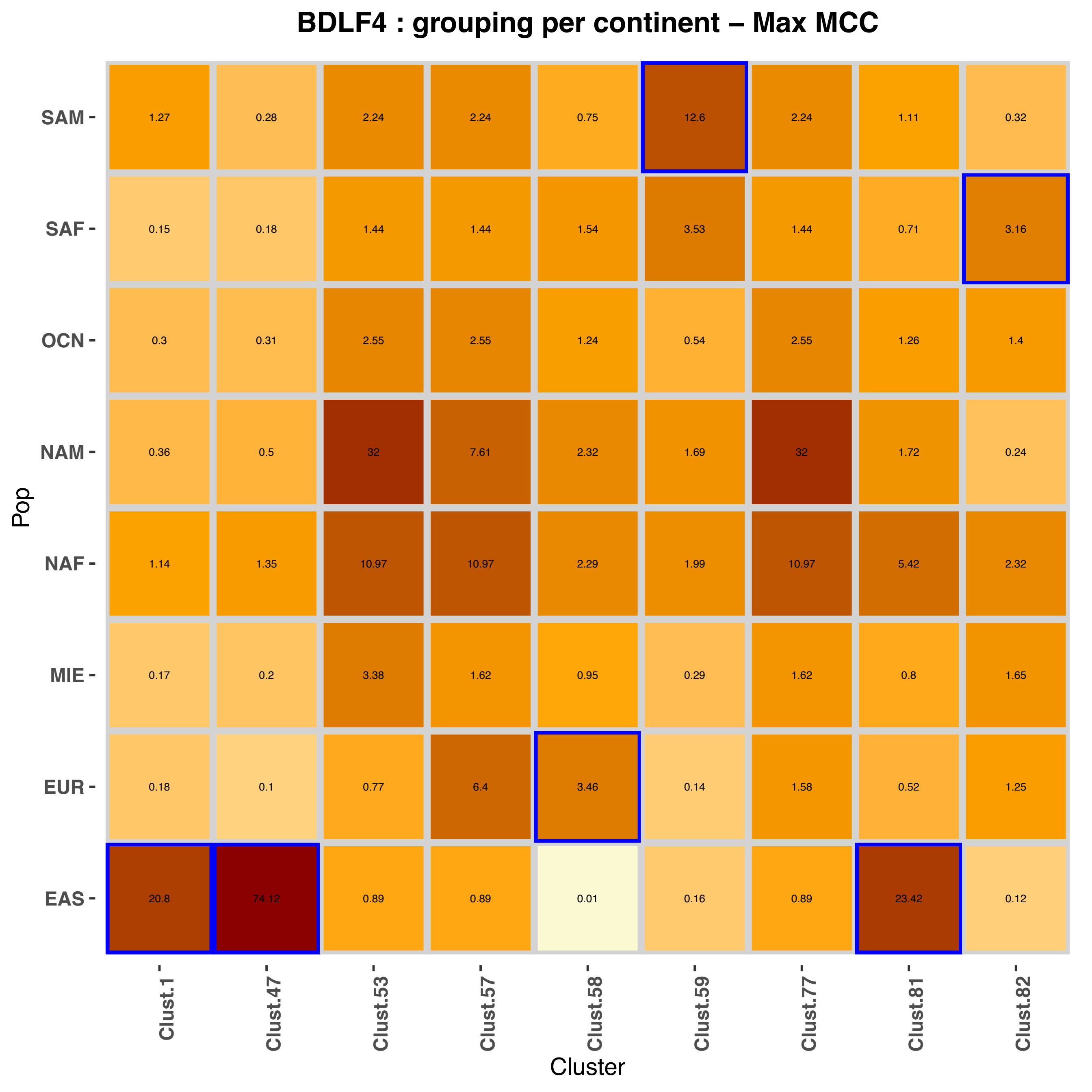
BDLF4 : grouping per continent – Max MCC
Pop
Cluster
SAM
SAF
OCN
NAM
NAF
MIE
EUR
EAS
Clust.1
Clust.47
Clust.53
Clust.57
Clust.58
Clust.59
Clust.77
Clust.81
Clust.82
1.27 0.28 2.24 2.24 0.75 12.6 2.24 1.11 0.32
0.15 0.18 1.44 1.44 1.54 3.53 1.44 0.71 3.16
0.3 0.31 2.55 2.55 1.24 0.54 2.55 1.26 1.4
0.36 0.5 32 7.61 2.32 1.69 32 1.72 0.24
1.14 1.35 10.97 10.97 2.29 1.99 10.97 5.42 2.32
0.17 0.2 3.38 1.62 0.95 0.29 1.62 0.8 1.65
0.18 0.1 0.77 6.4 3.46 0.14 1.58 0.52 1.25
20.8 74.12 0.89 0.89 0.01 0.16 0.89 23.42 0.12

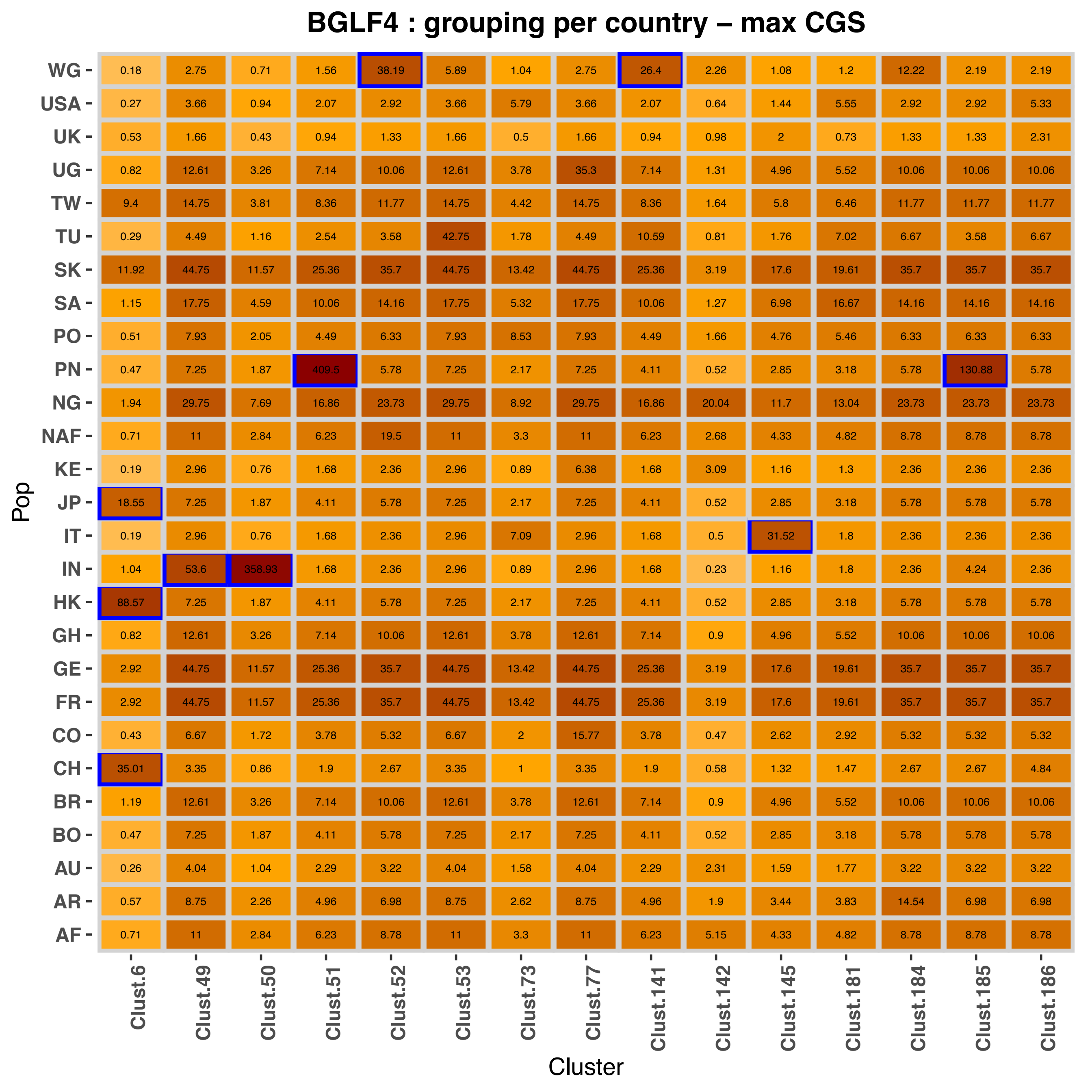
BGLF4 : grouping per country – max CGS
Pop
Cluster
Clust.6 Clust.49 Clust.50 Clust.51 Clust.52 Clust.53 Clust.73 Clust.77 Clust.141 Clust.142 Clust.145 Clust.181 Clust.184 Clust.185 Clust.186
WG 0.18 2.75 0.71 1.56 38.19 5.89 1.04 2.75 26.4 2.26 1.08 1.2 12.22 2.19 2.19
USA 0.27 3.66 0.94 2.07 2.92 3.66 5.79 3.66 2.07 0.64 1.44 5.55 2.92 2.92 5.33
UK 0.53 1.66 0.43 0.94 1.33 1.66 0.5 1.66 0.94 0.98 2 0.73 1.33 1.33 2.31
UG 0.82 12.61 3.26 7.14 10.06 12.61 3.78 35.3 7.14 1.31 4.96 5.52 10.06 10.06 10.06
TW 9.4 14.75 3.81 8.36 11.77 14.75 4.42 14.75 8.36 1.64 5.8 6.46 11.77 11.77 11.77
TU 0.29 4.49 1.16 2.54 3.58 42.75 1.78 4.49 10.59 0.81 1.76 7.02 6.67 3.58 6.67
SK 11.92 44.75 11.57 25.36 35.7 44.75 13.42 44.75 25.36 3.19 17.6 19.61 35.7 35.7 35.7
SA 1.15 17.75 4.59 10.06 14.16 17.75 5.32 17.75 10.06 1.27 6.98 16.67 14.16 14.16 14.16
PO 0.51 7.93 2.05 4.49 6.33 7.93 8.53 7.93 4.49 1.66 4.76 5.46 6.33 6.33 6.33
PN 0.47 7.25 1.87 409.5 5.78 7.25 2.17 7.25 4.11 0.52 2.85 3.18 5.78 130.88 5.78
NG 1.94 29.75 7.69 16.86 23.73 29.75 8.92 29.75 16.86 20.04 11.7 13.04 23.73 23.73 23.73
NAF 0.71 11 2.84 6.23 19.5 11 3.3 11 6.23 2.68 4.33 4.82 8.78 8.78 8.78
KE 0.19 2.96 0.76 1.68 2.36 2.96 0.89 6.38 1.68 3.09 1.16 1.3 2.36 2.36 2.36
JP 18.55 7.25 1.87 4.11 5.78 7.25 2.17 7.25 4.11 0.52 2.85 3.18 5.78 5.78 5.78
IT 0.19 2.96 0.76 1.68 2.36 2.96 7.09 2.96 1.68 0.5 31.52 1.8 2.36 2.36 2.36
IN 1.04 53.6 358.93 1.68 2.36 2.96 0.89 2.96 1.68 0.23 1.16 1.8 2.36 4.24 2.36
HK 88.57 7.25 1.87 4.11 5.78 7.25 2.17 7.25 4.11 0.52 2.85 3.18 5.78 5.78 5.78
GH 0.82 12.61 3.26 7.14 10.06 12.61 3.78 12.61 7.14 0.9 4.96 5.52 10.06 10.06 10.06
GE 2.92 44.75 11.57 25.36 35.7 44.75 13.42 44.75 25.36 3.19 17.6 19.61 35.7 35.7 35.7
FR 2.92 44.75 11.57 25.36 35.7 44.75 13.42 44.75 25.36 3.19 17.6 19.61 35.7 35.7 35.7
CO 0.43 6.67 1.72 3.78 5.32 6.67 2 15.77 3.78 0.47 2.62 2.92 5.32 5.32 5.32
CH 35.01 3.35 0.86 1.9 2.67 3.35 1 3.35 1.9 0.58 1.32 1.47 2.67 2.67 4.84
BR 1.19 12.61 3.26 7.14 10.06 12.61 3.78 12.61 7.14 0.9 4.96 5.52 10.06 10.06 10.06
BO 0.47 7.25 1.87 4.11 5.78 7.25 2.17 7.25 4.11 0.52 2.85 3.18 5.78 5.78 5.78
AU 0.26 4.04 1.04 2.29 3.22 4.04 1.58 4.04 2.29 2.31 1.59 1.77 3.22 3.22 3.22
AR 0.57 8.75 2.26 4.96 6.98 8.75 2.62 8.75 4.96 1.9 3.44 3.83 14.54 6.98 6.98
AF 0.71 11 2.84 6.23 8.78 11 3.3 11 6.23 5.15 4.33 4.82 8.78 8.78 8.78

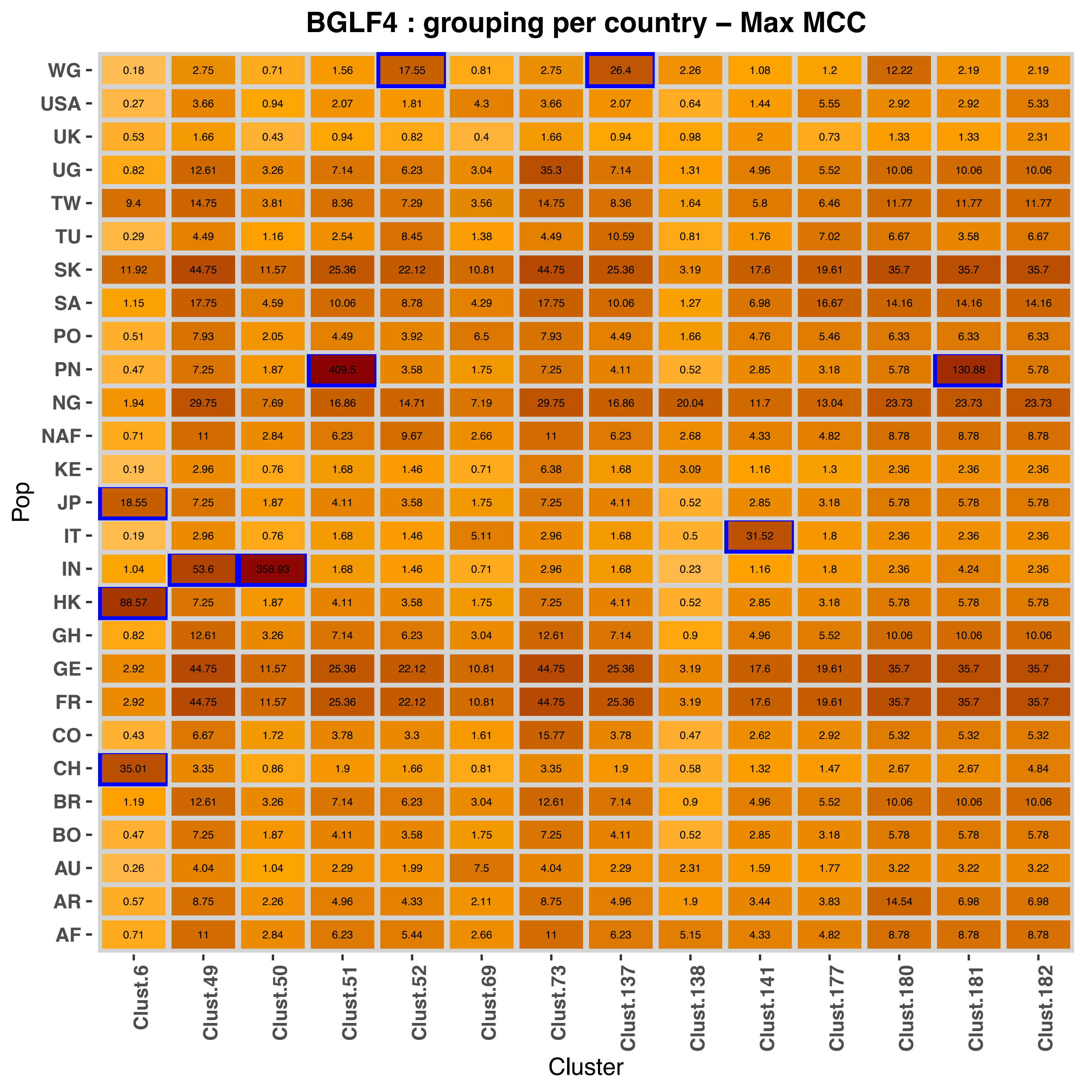
BGLF4 : grouping per country – Max MCC
Pop
Cluster
Clust.6 Clust.49 Clust.50 Clust.51 Clust.52 Clust.69 Clust.73 Clust.137 Clust.138 Clust.141 Clust.177 Clust.180 Clust.181 Clust.182
WG 0.18 2.75 0.71 1.56 17.55 0.81 2.75 26.4 2.26 1.08 1.2 12.22 2.19 2.19
USA 0.27 3.66 0.94 2.07 1.81 4.3 3.66 2.07 0.64 1.44 5.55 2.92 2.92 5.33
UK 0.53 1.66 0.43 0.94 0.82 0.4 1.66 0.94 0.98 2 0.73 1.33 1.33 2.31
UG 0.82 12.61 3.26 7.14 6.23 3.04 35.3 7.14 1.31 4.96 5.52 10.06 10.06 10.06
TW 9.4 14.75 3.81 8.36 7.29 3.56 14.75 8.36 1.64 5.8 6.46 11.77 11.77 11.77
TU 0.29 4.49 1.16 2.54 8.45 1.38 4.49 10.59 0.81 1.76 7.02 6.67 3.58 6.67
SK 11.92 44.75 11.57 25.36 22.12 10.81 44.75 25.36 3.19 17.6 19.61 35.7 35.7 35.7
SA 1.15 17.75 4.59 10.06 8.78 4.29 17.75 10.06 1.27 6.98 16.67 14.16 14.16 14.16
PO 0.51 7.93 2.05 4.49 3.92 6.5 7.93 4.49 1.66 4.76 5.46 6.33 6.33 6.33
PN 0.47 7.25 1.87 409.5 3.58 1.75 7.25 4.11 0.52 2.85 3.18 5.78 130.88 5.78
NG 1.94 29.75 7.69 16.86 14.71 7.19 29.75 16.86 20.04 11.7 13.04 23.73 23.73 23.73
NAF 0.71 11 2.84 6.23 9.67 2.66 11 6.23 2.68 4.33 4.82 8.78 8.78 8.78
KE 0.19 2.96 0.76 1.68 1.46 0.71 6.38 1.68 3.09 1.16 1.3 2.36 2.36 2.36
JP 18.55 7.25 1.87 4.11 3.58 1.75 7.25 4.11 0.52 2.85 3.18 5.78 5.78 5.78
IT 0.19 2.96 0.76 1.68 1.46 5.11 2.96 1.68 0.5 31.52 1.8 2.36 2.36 2.36
IN 1.04 53.6 358.93 1.68 1.46 0.71 2.96 1.68 0.23 1.16 1.8 2.36 4.24 2.36
HK 88.57 7.25 1.87 4.11 3.58 1.75 7.25 4.11 0.52 2.85 3.18 5.78 5.78 5.78
GH 0.82 12.61 3.26 7.14 6.23 3.04 12.61 7.14 0.9 4.96 5.52 10.06 10.06 10.06
GE 2.92 44.75 11.57 25.36 22.12 10.81 44.75 25.36 3.19 17.6 19.61 35.7 35.7 35.7
FR 2.92 44.75 11.57 25.36 22.12 10.81 44.75 25.36 3.19 17.6 19.61 35.7 35.7 35.7
CO 0.43 6.67 1.72 3.78 3.3 1.61 15.77 3.78 0.47 2.62 2.92 5.32 5.32 5.32
CH 35.01 3.35 0.86 1.9 1.66 0.81 3.35 1.9 0.58 1.32 1.47 2.67 2.67 4.84
BR 1.19 12.61 3.26 7.14 6.23 3.04 12.61 7.14 0.9 4.96 5.52 10.06 10.06 10.06
BO 0.47 7.25 1.87 4.11 3.58 1.75 7.25 4.11 0.52 2.85 3.18 5.78 5.78 5.78
AU 0.26 4.04 1.04 2.29 1.99 7.5 4.04 2.29 2.31 1.59 1.77 3.22 3.22 3.22
AR 0.57 8.75 2.26 4.96 4.33 2.11 8.75 4.96 1.9 3.44 3.83 14.54 6.98 6.98
AF 0.71 11 2.84 6.23 5.44 2.66 11 6.23 5.15 4.33 4.82 8.78 8.78 8.78

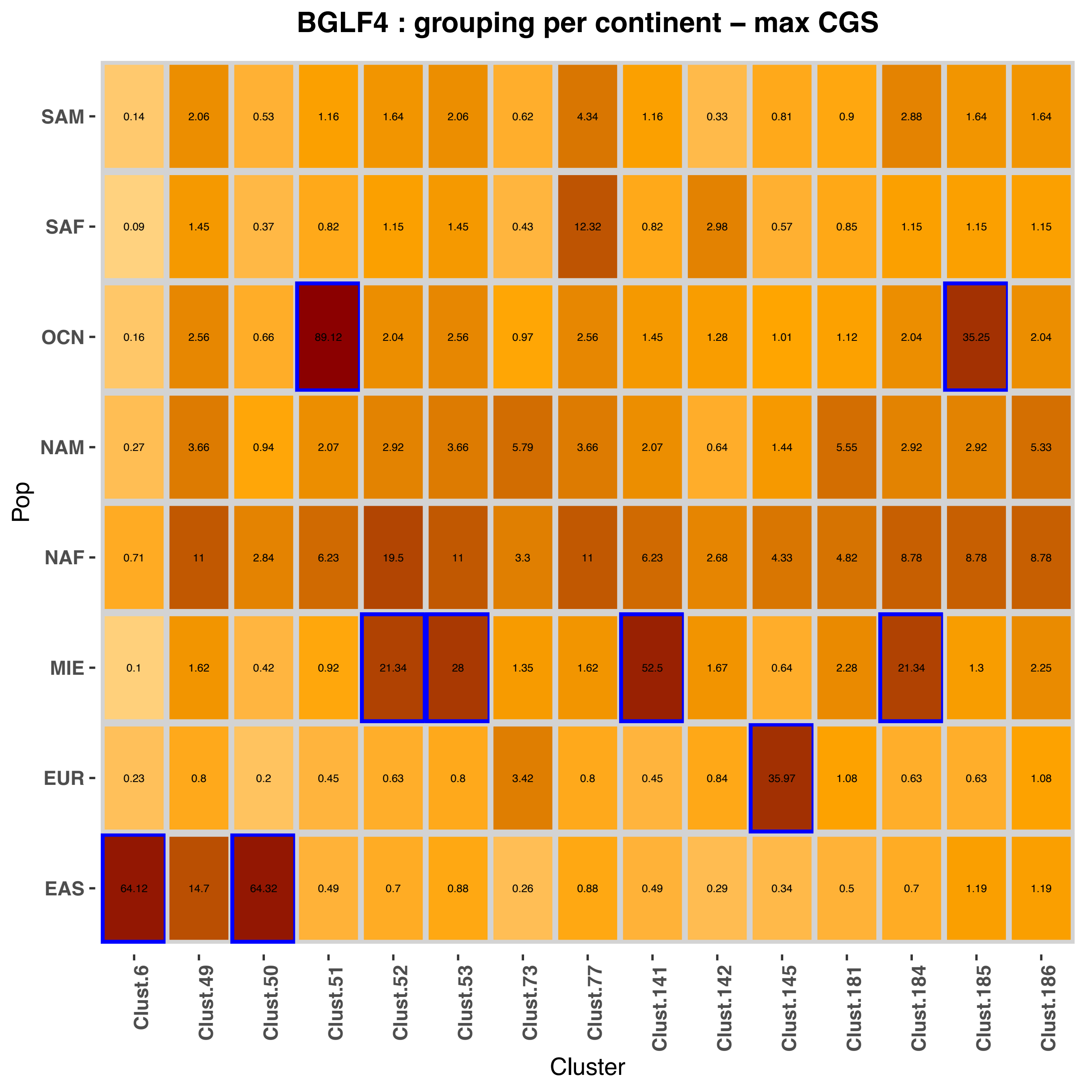

BGLF4 : grouping per continent – max CGS
Pop
SAM
SAF
OCN
NAM
NAF
MIE
EUR
EAS
0.14 2.06 0.53 1.16 1.64 2.06 0.62 4.34 1.16 0.33 0.81 0.9 2.88 1.64 1.64
0.09 1.45 0.37 0.82 1.15 1.45 0.43 12.32 0.82 2.98 0.57 0.85 1.15 1.15 1.15
0.16 2.56 0.66 89.12 2.04 2.56 0.97 2.56 1.45 1.28 1.01 1.12 2.04 35.25 2.04
0.27 3.66 0.94 2.07 2.92 3.66 5.79 3.66 2.07 0.64 1.44 5.55 2.92 2.92 5.33
0.71 11 2.84 6.23 19.5 11 3.3 11 6.23 2.68 4.33 4.82 8.78 8.78 8.78
0.1 1.62 0.42 0.92 21.34 28 1.35 1.62 52.5 1.67 0.64 2.28 21.34 1.3 2.25
0.23 0.8 0.2 0.45 0.63 0.8 3.42 0.8 0.45 0.84 35.97 1.08 0.63 0.63 1.08
64.12 14.7 64.32 0.49 0.7 0.88 0.26 0.88 0.49 0.29 0.34 0.5 0.7 1.19 1.19
Clust.6
Clust.49
Clust.50
Clust.51
Clust.52
Clust.53
Clust.73
Clust.77
Clust.141
Clust.142
Clust.145
Clust.181
Clust.184
Clust.185
Clust.186
Cluster

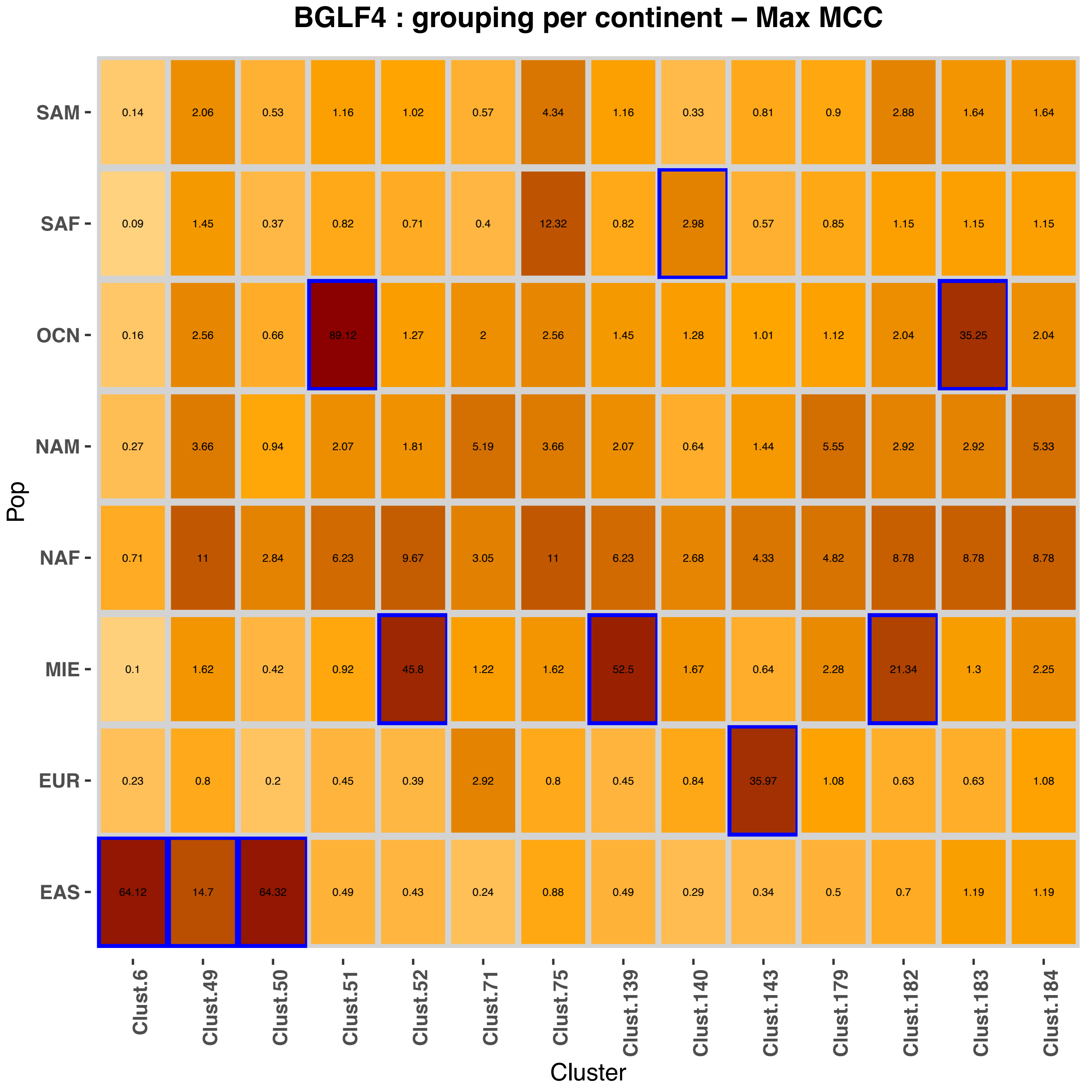
BGLF4 : grouping per continent – Max MCC
Pop
SAM
SAF
OCN
NAM
NAF
MIE
EUR
EAS
0.14 2.06 0.53 1.16 1.02 0.57 4.34 1.16 0.33 0.81 0.9 2.88 1.64 1.64
0.09 1.45 0.37 0.82 0.71 0.4 12.32 0.82 2.98 0.57 0.85 1.15 1.15 1.15
0.16 2.56 0.66 89.12 1.27 2 2.56 1.45 1.28 1.01 1.12 2.04 35.25 2.04
0.27 3.66 0.94 2.07 1.81 5.19 3.66 2.07 0.64 1.44 5.55 2.92 2.92 5.33
0.71 11 2.84 6.23 9.67 3.05 11 6.23 2.68 4.33 4.82 8.78 8.78 8.78
0.1 1.62 0.42 0.92 45.8 1.22 1.62 52.5 1.67 0.64 2.28 21.34 1.3 2.25
0.23 0.8 0.2 0.45 0.39 2.92 0.8 0.45 0.84 35.97 1.08 0.63 0.63 1.08
64.12 14.7 64.32 0.49 0.43 0.24 0.88 0.49 0.29 0.34 0.5 0.7 1.19 1.19
Clust.6
Clust.49
Clust.50
Clust.51
Clust.52
Clust.71
Clust.75
Clust.139
Clust.140
Clust.143
Clust.179
Clust.182
Clust.183
Clust.184
Cluster

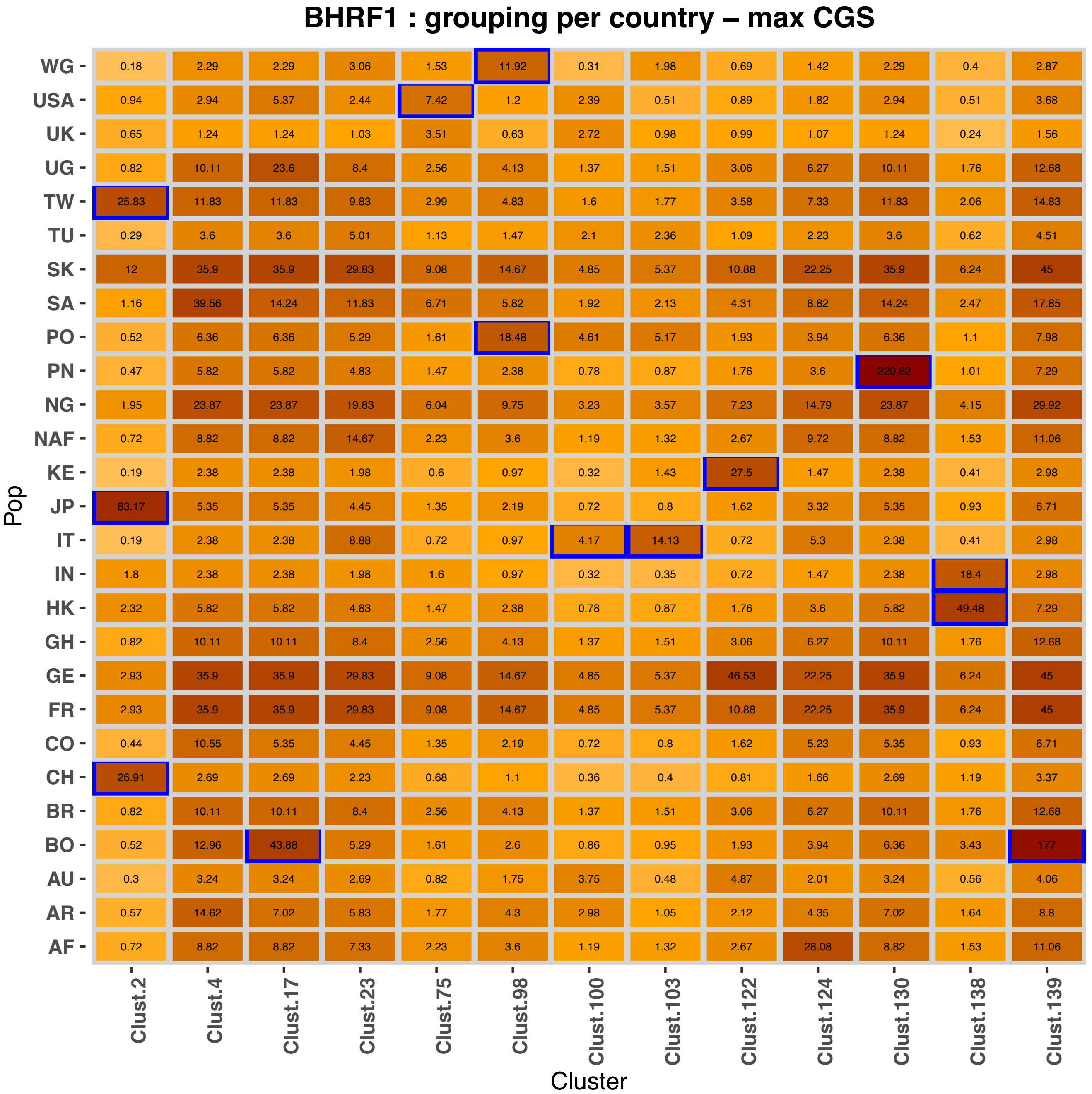
BHRF1 : grouping per country – max CGS
Pop
Cluster
Clust.2 Clust.4 Clust.17 Clust.23 Clust.75 Clust.98 Clust.100 Clust.103 Clust.122 Clust.124 Clust.130 Clust.138 Clust.139
WG 0.18 2.29 2.29 3.06 1.53 11.92 0.31 1.98 0.69 1.42 2.29 0.4 2.87
USA 0.94 2.94 5.37 2.44 7.42 1.2 2.39 0.51 0.89 1.82 2.94 0.51 3.68
UK 0.65 1.24 1.24 1.03 3.51 0.63 2.72 0.98 0.99 1.07 1.24 0.24 1.56
UG 0.82 10.11 23.6 8.4 2.56 4.13 1.37 1.51 3.06 6.27 10.11 1.76 12.68
TW 25.83 11.83 11.83 9.83 2.99 4.83 1.6 1.77 3.58 7.33 11.83 2.06 14.83
TU 0.29 3.6 3.6 5.01 1.13 1.47 2.1 2.36 1.09 2.23 3.6 0.62 4.51
SK 12 35.9 35.9 29.83 9.08 14.67 4.85 5.37 10.88 22.25 35.9 6.24 45
SA 1.16 39.56 14.24 11.83 6.71 5.82 1.92 2.13 4.31 8.82 14.24 2.47 17.85
PO 0.52 6.36 6.36 5.29 1.61 18.48 4.61 5.17 1.93 3.94 6.36 1.1 7.98
PN 0.47 5.82 5.82 4.83 1.47 2.38 0.78 0.87 1.76 3.6 220.62 1.01 7.29
NG 1.95 23.87 23.87 19.83 6.04 9.75 3.23 3.57 7.23 14.79 23.87 4.15 29.92
NAF 0.72 8.82 8.82 14.67 2.23 3.6 1.19 1.32 2.67 9.72 8.82 1.53 11.06
KE 0.19 2.38 2.38 1.98 0.6 0.97 0.32 1.43 27.5 1.47 2.38 0.41 2.98
JP 83.17 5.35 5.35 4.45 1.35 2.19 0.72 0.8 1.62 3.32 5.35 0.93 6.71
IT 0.19 2.38 2.38 8.88 0.72 0.97 4.17 14.13 0.72 5.3 2.38 0.41 2.98
IN 1.8 2.38 2.38 1.98 1.6 0.97 0.32 0.35 0.72 1.47 2.38 18.4 2.98
HK 2.32 5.82 5.82 4.83 1.47 2.38 0.78 0.87 1.76 3.6 5.82 49.48 7.29
GH 0.82 10.11 10.11 8.4 2.56 4.13 1.37 1.51 3.06 6.27 10.11 1.76 12.68
GE 2.93 35.9 35.9 29.83 9.08 14.67 4.85 5.37 46.53 22.25 35.9 6.24 45
FR 2.93 35.9 35.9 29.83 9.08 14.67 4.85 5.37 10.88 22.25 35.9 6.24 45
CO 0.44 10.55 5.35 4.45 1.35 2.19 0.72 0.8 1.62 5.23 5.35 0.93 6.71
CH 26.91 2.69 2.69 2.23 0.68 1.1 0.36 0.4 0.81 1.66 2.69 1.19 3.37
BR 0.82 10.11 10.11 8.4 2.56 4.13 1.37 1.51 3.06 6.27 10.11 1.76 12.68
BO 0.52 12.96 43.88 5.29 1.61 2.6 0.86 0.95 1.93 3.94 6.36 3.43 177
AU 0.3 3.24 3.24 2.69 0.82 1.75 3.75 0.48 4.87 2.01 3.24 0.56 4.06
AR 0.57 14.62 7.02 5.83 1.77 4.3 2.98 1.05 2.12 4.35 7.02 1.64 8.8
AF 0.72 8.82 8.82 7.33 2.23 3.6 1.19 1.32 2.67 28.08 8.82 1.53 11.06

BHRF1 : grouping per country – Max MCC

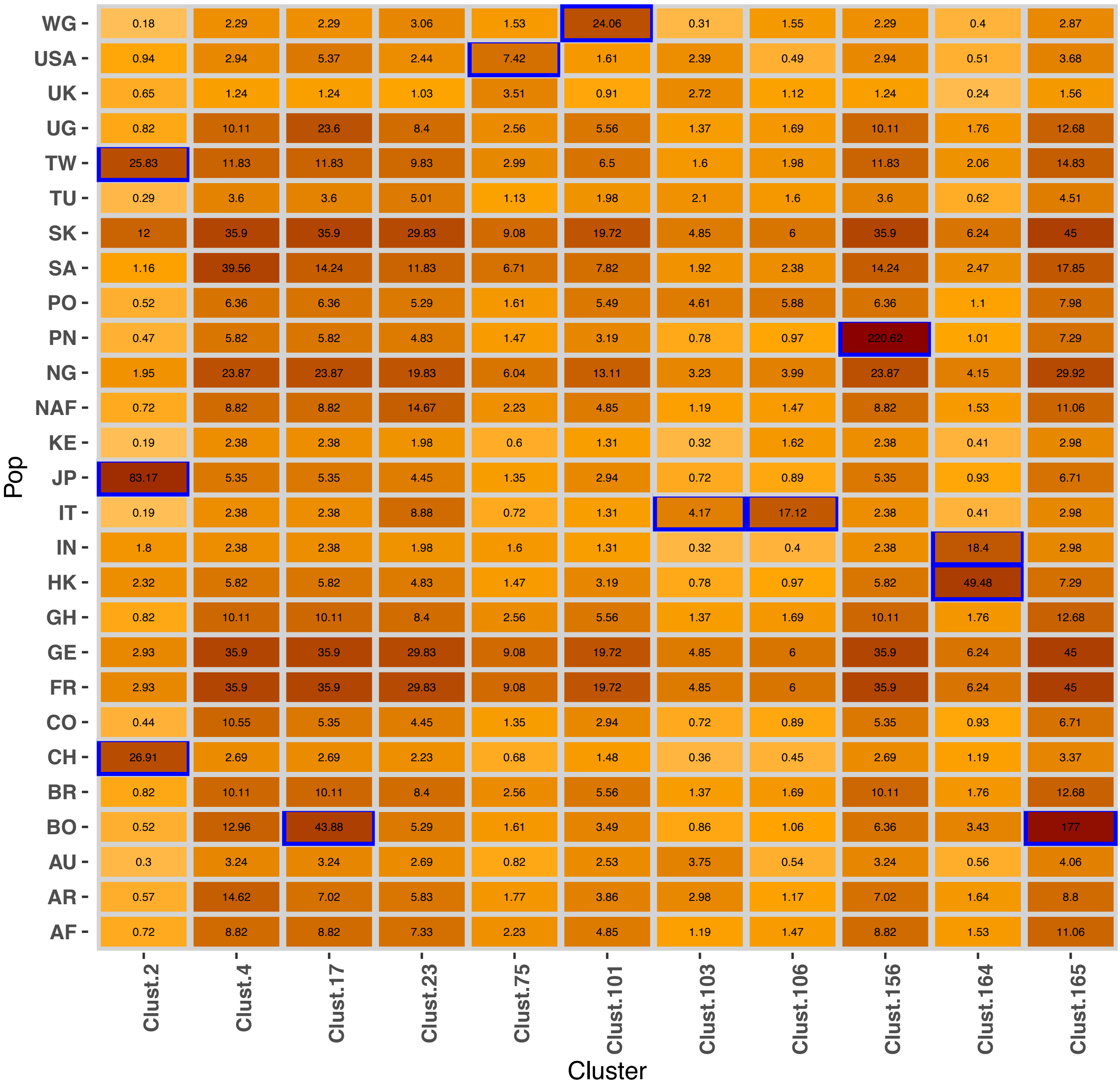

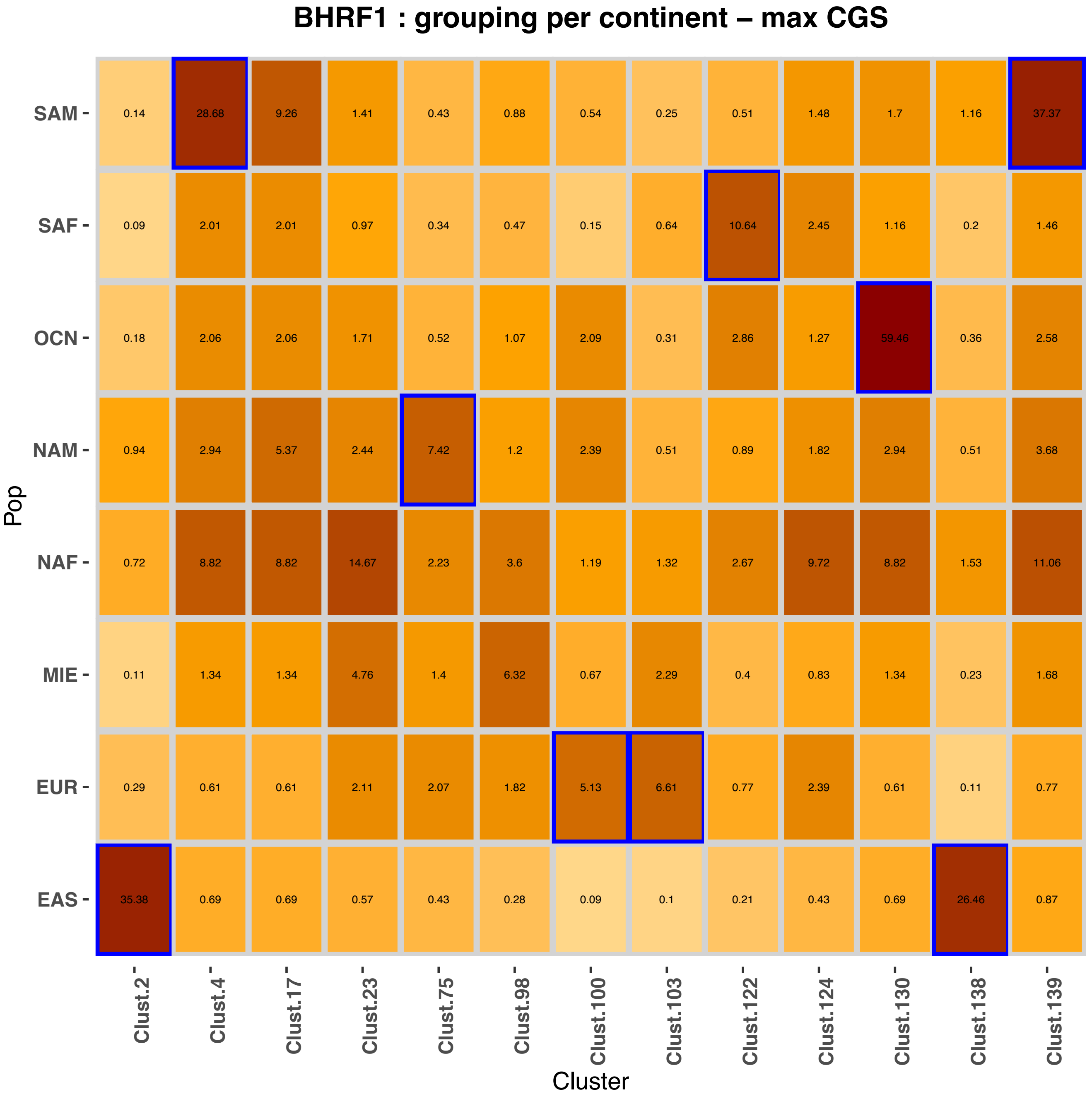
BHRF1 : grouping per continent – max CGS
Pop
SAM
SAF
OCN
NAM
NAF
MIE
EUR
EAS
Cluster
Clust.2
Clust.4
Clust.17
Clust.23
Clust.75
Clust.98
Clust.100
Clust.103
Clust.122
Clust.124
Clust.130
Clust.138
Clust.139
SAM: 0.14 28.68 9.26 1.41 0.43 0.88 0.54 0.25 0.51 1.48 1.7 1.16 37.37
SAF: 0.09 2.01 2.01 0.97 0.34 0.47 0.15 0.64 10.64 2.45 1.16 0.2 1.46
OCN: 0.18 2.06 2.06 1.71 0.52 1.07 2.09 0.31 2.86 1.27 59.46 0.36 2.58
NAM: 0.94 2.94 5.37 2.44 7.42 1.2 2.39 0.51 0.89 1.82 2.94 0.51 3.68
NAF: 0.72 8.82 8.82 14.67 2.23 3.6 1.19 1.32 2.67 9.72 8.82 1.53 11.06
MIE: 0.11 1.34 1.34 4.76 1.4 6.32 0.67 2.29 0.4 0.83 1.34 0.23 1.68
EUR: 0.29 0.61 0.61 2.11 2.07 1.82 5.13 6.61 0.77 2.39 0.61 0.11 0.77
EAS: 35.38 0.69 0.69 0.57 0.43 0.28 0.09 0.1 0.21 0.43 0.69 26.46 0.87

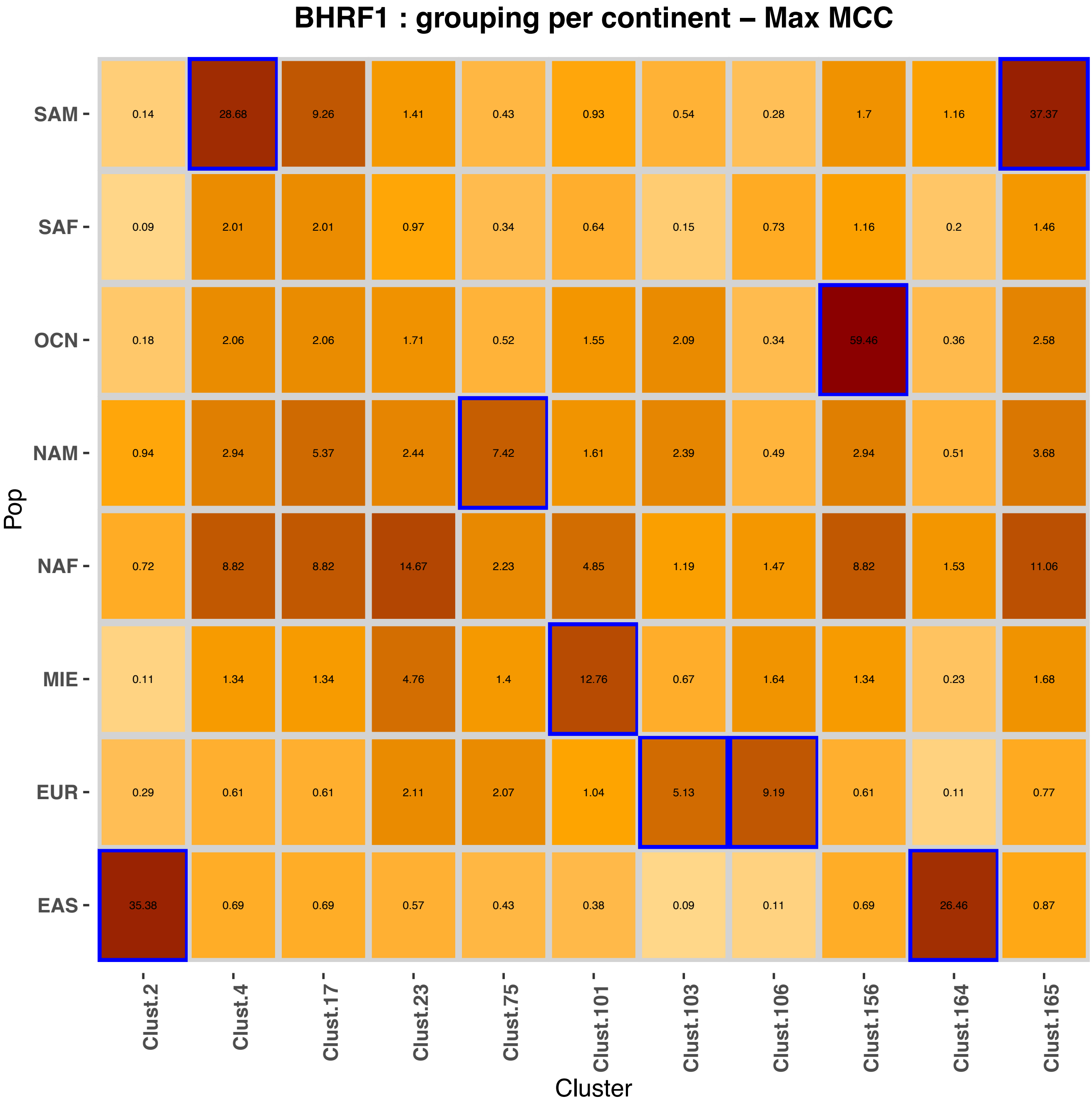
BHRF1 : grouping per continent – Max MCC
Pop
SAM
SAF
OCN
NAM
NAF
MIE
EUR
EAS
0.14 28.68 9.26 1.41 0.43 0.93 0.54 0.28 1.7 1.16 37.37
0.09 2.01 2.01 0.97 0.34 0.64 0.15 0.73 1.16 0.2 1.46
0.18 2.06 2.06 1.71 0.52 1.55 2.09 0.34 59.46 0.36 2.58
0.94 2.94 5.37 2.44 7.42 1.61 2.39 0.49 2.94 0.51 3.68
0.72 8.82 8.82 14.67 2.23 4.85 1.19 1.47 8.82 1.53 11.06
0.11 1.34 1.34 4.76 1.4 12.76 0.67 1.64 1.34 0.23 1.68
0.29 0.61 0.61 2.11 2.07 1.04 5.13 9.19 0.61 0.11 0.77
35.38 0.69 0.69 0.57 0.43 0.38 0.09 0.11 0.69 26.46 0.87
Clust.2
Clust.4
Clust.17
Clust.23
Clust.75
Clust.101
Clust.103
Clust.106
Clust.156
Clust.164
Clust.165
Cluster

BRLF1 : grouping per country – max CGS

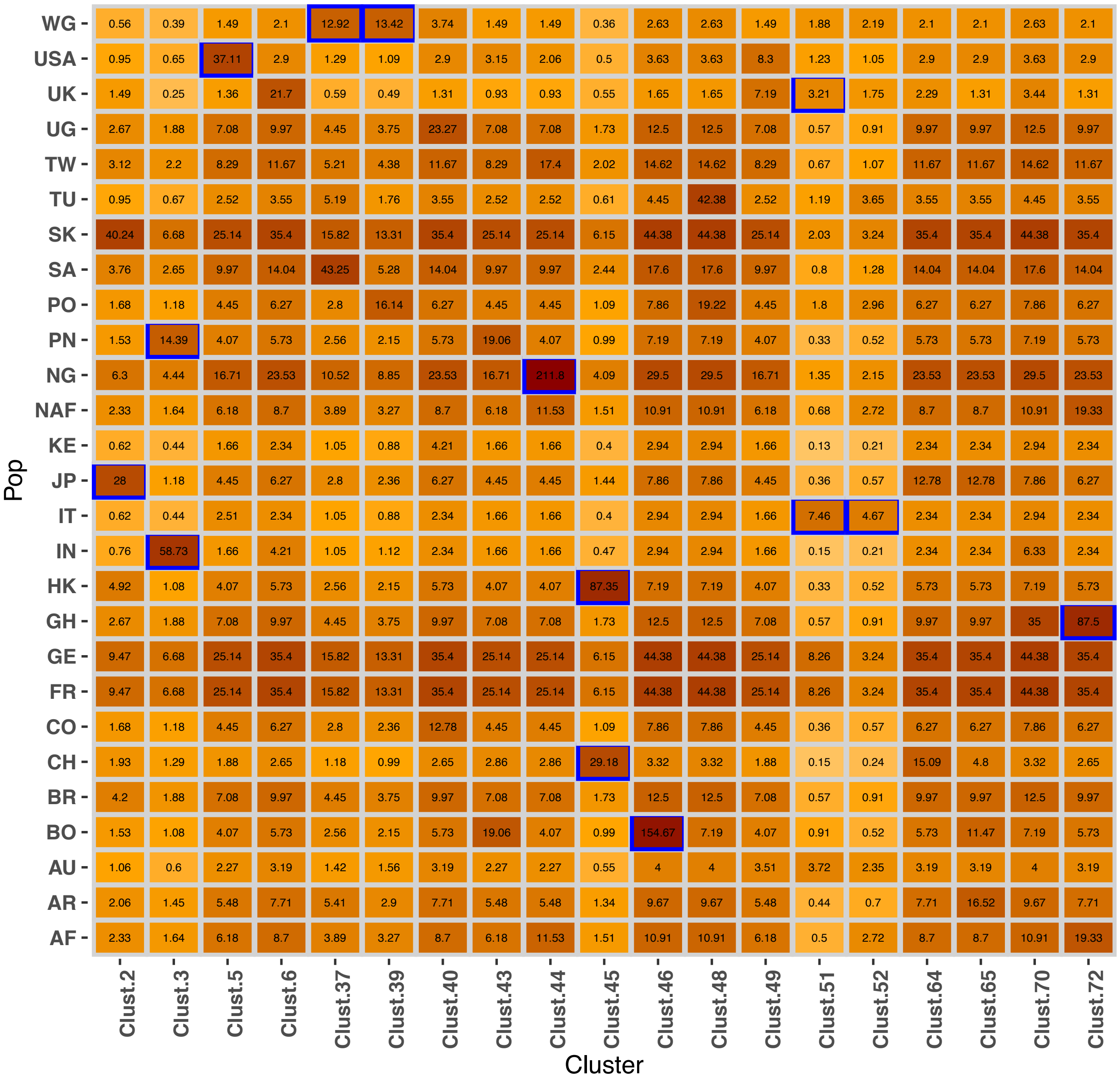

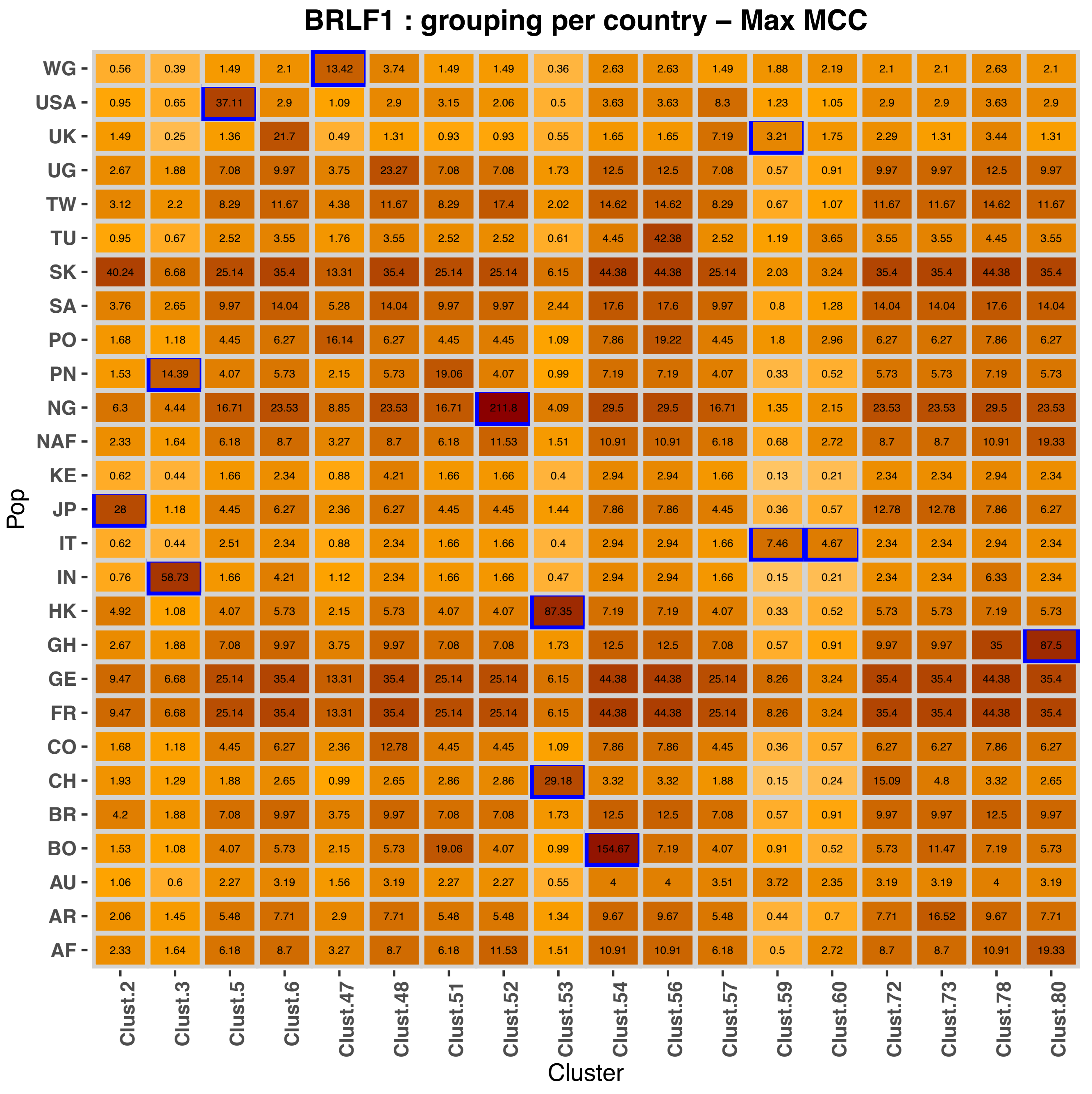
BRLF1 : grouping per country – Max MCC
Pop
Cluster
Clust.2 Clust.3 Clust.5 Clust.6 Clust.47 Clust.48 Clust.51 Clust.52 Clust.53 Clust.54 Clust.56 Clust.57 Clust.59 Clust.60 Clust.72 Clust.73 Clust.78 Clust.80
WG 0.56 0.39 1.49 2.1 13.42 3.74 1.49 1.49 0.36 2.63 2.63 1.49 1.88 2.19 2.1 2.1 2.63 2.1
USA 0.95 0.65 37.11 2.9 1.09 2.9 3.15 2.06 0.5 3.63 3.63 8.3 1.23 1.05 2.9 2.9 3.63 2.9
UK 1.49 0.25 1.36 21.7 0.49 1.31 0.93 0.93 0.55 1.65 1.65 7.19 3.21 1.75 2.29 1.31 3.44 1.31
UG 2.67 1.88 7.08 9.97 3.75 23.27 7.08 7.08 1.73 12.5 12.5 7.08 0.57 0.91 9.97 9.97 12.5 9.97
TW 3.12 2.2 8.29 11.67 4.38 11.67 8.29 17.4 2.02 14.62 14.62 8.29 0.67 1.07 11.67 11.67 14.62 11.67
TU 0.95 0.67 2.52 3.55 1.76 3.55 2.52 2.52 0.61 4.45 42.38 2.52 1.19 3.65 3.55 3.55 4.45 3.55
SK 40.24 6.68 25.14 35.4 13.31 35.4 25.14 25.14 6.15 44.38 44.38 25.14 2.03 3.24 35.4 35.4 44.38 35.4
SA 3.76 2.65 9.97 14.04 5.28 14.04 9.97 9.97 2.44 17.6 17.6 9.97 0.8 1.28 14.04 14.04 17.6 14.04
PO 1.68 1.18 4.45 6.27 16.14 6.27 4.45 4.45 1.09 7.86 19.22 4.45 1.8 2.96 6.27 6.27 7.86 6.27
PN 1.53 14.39 4.07 5.73 2.15 5.73 19.06 4.07 0.99 7.19 7.19 4.07 0.33 0.52 5.73 5.73 7.19 5.73
NG 6.3 4.44 16.71 23.53 8.85 23.53 16.71 211.8 4.09 29.5 29.5 16.71 1.35 2.15 23.53 23.53 29.5 23.53
NAF 2.33 1.64 6.18 8.7 3.27 8.7 6.18 11.53 1.51 10.91 10.91 6.18 0.68 2.72 8.7 8.7 10.91 19.33
KE 0.62 0.44 1.66 2.34 0.88 4.21 1.66 1.66 0.4 2.94 2.94 1.66 0.13 0.21 2.34 2.34 2.94 2.34
JP 28 1.18 4.45 6.27 2.36 6.27 4.45 4.45 1.44 7.86 7.86 4.45 0.36 0.57 12.78 12.78 7.86 6.27
IT 0.62 0.44 2.51 2.34 0.88 2.34 1.66 1.66 0.4 2.94 2.94 1.66 7.46 4.67 2.34 2.34 2.94 2.34
IN 0.76 58.73 1.66 4.21 1.12 2.34 1.66 1.66 0.47 2.94 2.94 1.66 0.15 0.21 2.34 2.34 6.33 2.34
HK 4.92 1.08 4.07 5.73 2.15 5.73 4.07 4.07 87.35 7.19 7.19 4.07 0.33 0.52 5.73 5.73 7.19 5.73
GH 2.67 1.88 7.08 9.97 3.75 9.97 7.08 7.08 1.73 12.5 12.5 7.08 0.57 0.91 9.97 9.97 35 87.5
GE 9.47 6.68 25.14 35.4 13.31 35.4 25.14 25.14 6.15 44.38 44.38 25.14 8.26 3.24 35.4 35.4 44.38 35.4
FR 9.47 6.68 25.14 35.4 13.31 35.4 25.14 25.14 6.15 44.38 44.38 25.14 8.26 3.24 35.4 35.4 44.38 35.4
CO 1.68 1.18 4.45 6.27 2.36 12.78 4.45 4.45 1.09 7.86 7.86 4.45 0.36 0.57 6.27 6.27 7.86 6.27
CH 1.93 1.29 1.88 2.65 0.99 2.65 2.86 2.86 29.18 3.32 3.32 1.88 0.15 0.24 15.09 4.8 3.32 2.65
BR 4.2 1.88 7.08 9.97 3.75 9.97 7.08 7.08 1.73 12.5 12.5 7.08 0.57 0.91 9.97 9.97 12.5 9.97
BO 1.53 1.08 4.07 5.73 2.15 5.73 19.06 4.07 0.99 154.67 7.19 4.07 0.91 0.52 5.73 11.47 7.19 5.73
AU 1.06 0.6 2.27 3.19 1.56 3.19 2.27 2.27 0.55 4 4 3.51 3.72 2.35 3.19 3.19 4 3.19
AR 2.06 1.45 5.48 7.71 2.9 7.71 5.48 5.48 1.34 9.67 9.67 5.48 0.44 0.7 7.71 16.52 9.67 7.71
AF 2.33 1.64 6.18 8.7 3.27 8.7 6.18 11.53 1.51 10.91 10.91 6.18 0.5 2.72 8.7 8.7 10.91 19.33

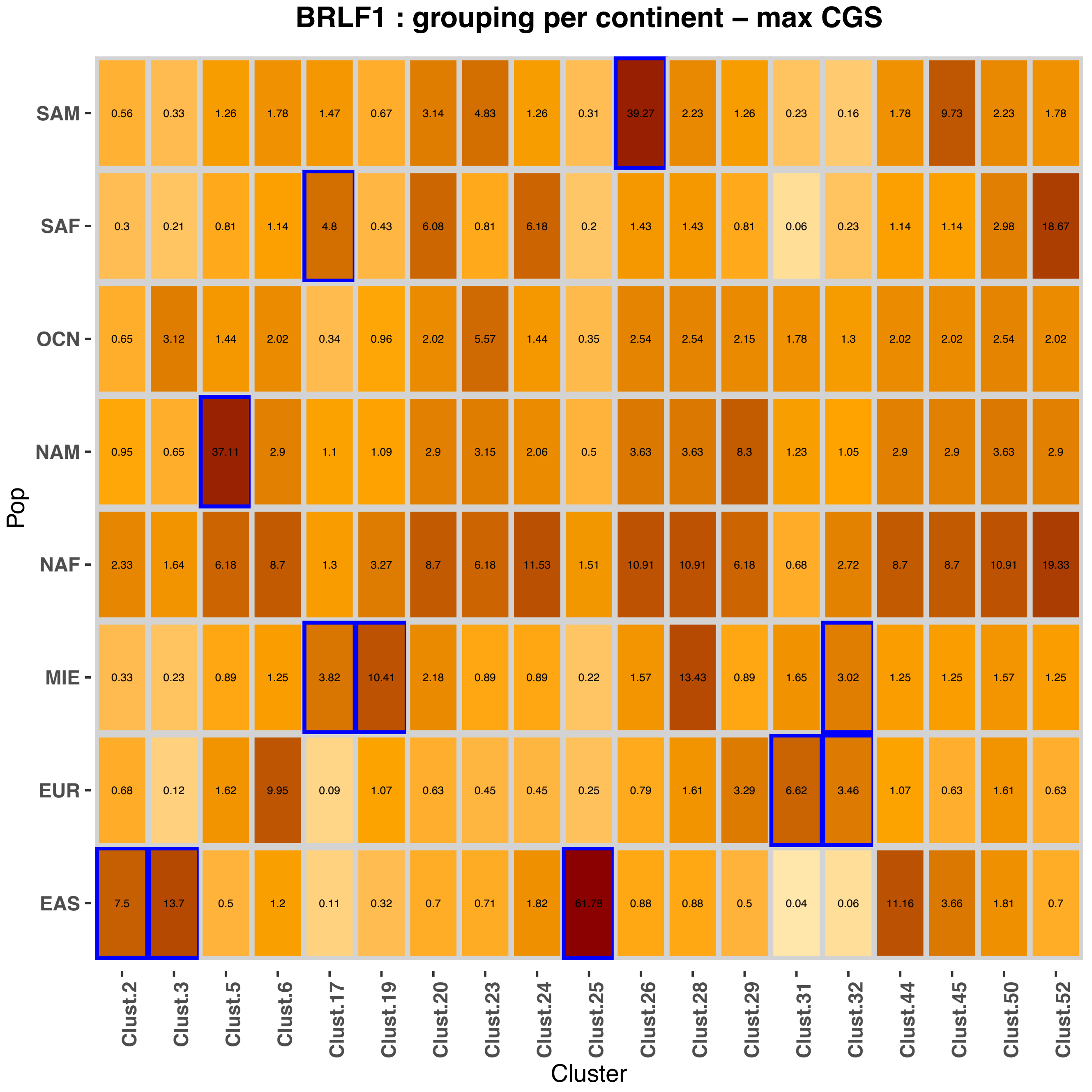

BRLF1 : grouping per continent – max CGS
Pop
Cluster
SAM
SAF
OCN
NAM
NAF
MIE
EUR
EAS
Clust.2
Clust.3
Clust.5
Clust.6
Clust.17
Clust.19
Clust.20
Clust.23
Clust.24
Clust.25
Clust.26
Clust.28
Clust.29
Clust.31
Clust.32
Clust.44
Clust.45
Clust.50
Clust.52
0.56 0.33 1.26 1.78 1.47 0.67 3.14 4.83 1.26 0.31 39.27 2.23 1.26 0.23 0.16 1.78 9.73 2.23 1.78
0.3 0.21 0.81 1.14 4.8 0.43 6.08 0.81 6.18 0.2 1.43 1.43 0.81 0.06 0.23 1.14 1.14 2.98 18.67
0.65 3.12 1.44 2.02 0.34 0.96 2.02 5.57 1.44 0.35 2.54 2.54 2.15 1.78 1.3 2.02 2.02 2.54 2.02
0.95 0.65 37.11 2.9 1.1 1.09 2.9 3.15 2.06 0.5 3.63 3.63 8.3 1.23 1.05 2.9 2.9 3.63 2.9
2.33 1.64 6.18 8.7 1.3 3.27 8.7 6.18 11.53 1.51 10.91 10.91 6.18 0.68 2.72 8.7 8.7 10.91 19.33
0.33 0.23 0.89 1.25 3.82 10.41 2.18 0.89 0.89 0.22 1.57 13.43 0.89 1.65 3.02 1.25 1.25 1.57 1.25
0.68 0.12 1.62 9.95 0.09 1.07 0.63 0.45 0.45 0.25 0.79 1.61 3.29 6.62 3.46 1.07 0.63 1.61 0.63
7.5 13.7 0.5 1.2 0.11 0.32 0.7 0.71 1.82 61.78 0.88 0.88 0.5 0.04 0.06 11.16 3.66 1.81 0.7

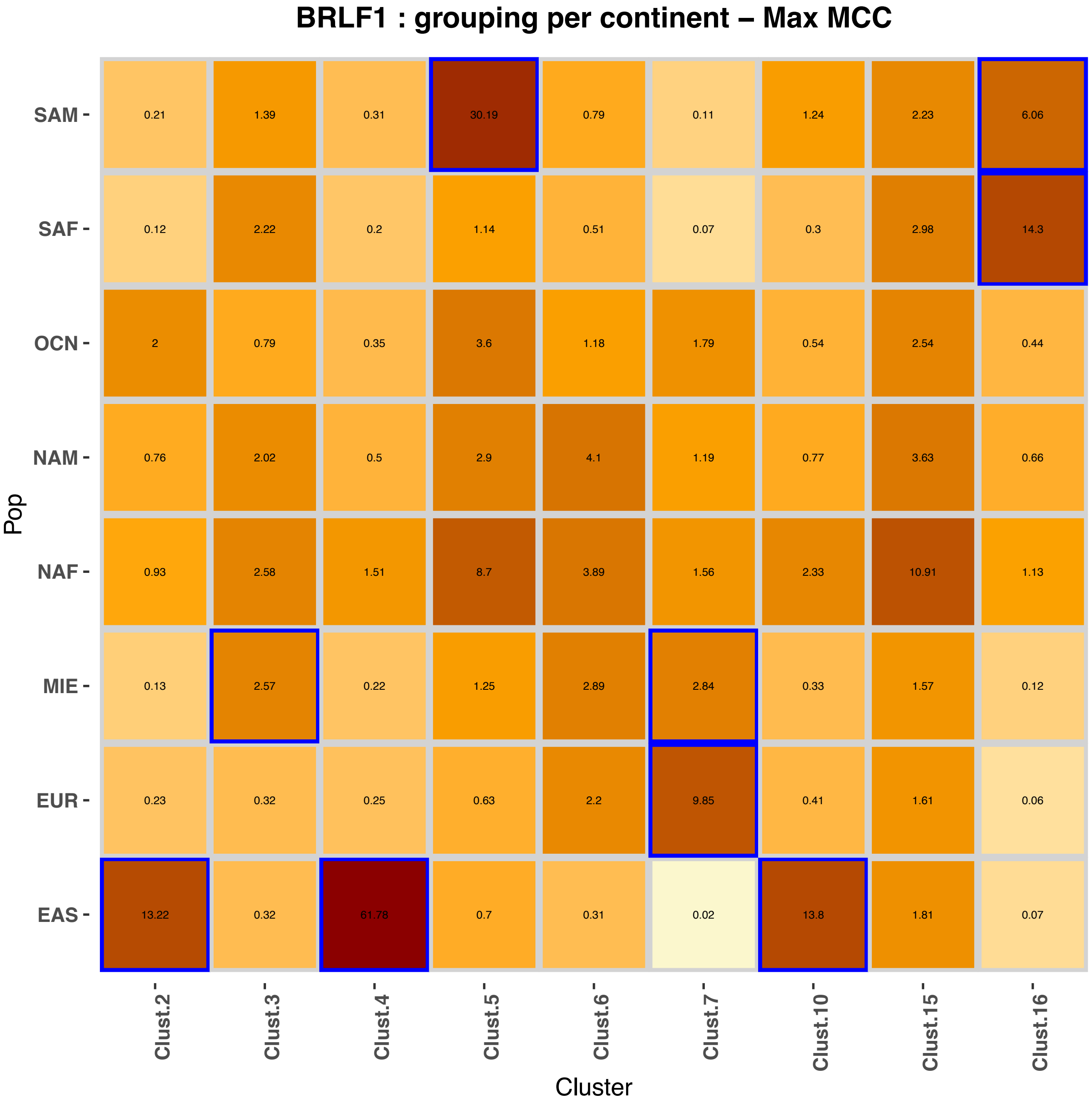
BRLF1 : grouping per continent – Max MCC
Pop
SAM
SAF
OCN
NAM
NAF
MIE
EUR
EAS
Cluster
Clust.2
Clust.3
Clust.4
Clust.5
Clust.6
Clust.7
Clust.10
Clust.15
Clust.16
0.21
1.39
0.31
30.19
0.79
0.11
1.24
2.23
6.06
0.12
2.22
0.2
1.14
0.51
0.07
0.3
2.98
14.3
2
0.79
0.35
3.6
1.18
1.79
0.54
2.54
0.44
0.76
2.02
0.5
2.9
4.1
1.19
0.77
3.63
0.66
0.93
2.58
1.51
8.7
3.89
1.56
2.33
10.91
1.13
0.13
2.57
0.22
1.25
2.89
2.84
0.33
1.57
0.12
0.23
0.32
0.25
0.63
2.2
9.85
0.41
1.61
0.06
13.22
0.32
61.78
0.7
0.31
0.02
13.8
1.81
0.07

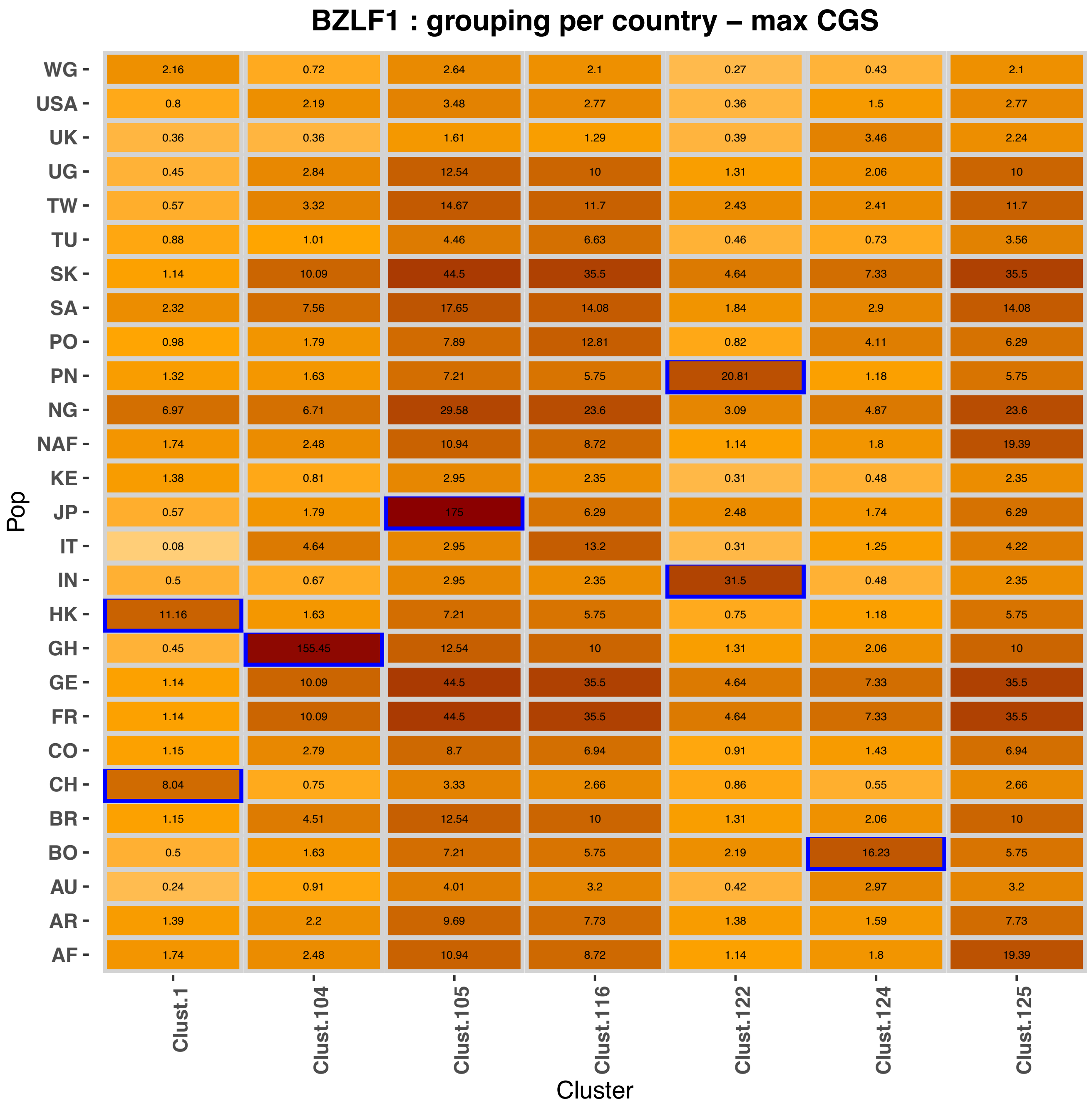
BZLF1 : grouping per country – max CGS
Pop
Cluster
Clust.1
Clust.104
Clust.105
Clust.116
Clust.122
Clust.124
Clust.125
WG 2.16 0.72 2.64 2.1 0.27 0.43 2.1
USA 0.8 2.19 3.48 2.77 0.36 1.5 2.77
UK 0.36 0.36 1.61 1.29 0.39 3.46 2.24
UG 0.45 2.84 12.54 10 1.31 2.06 10
TW 0.57 3.32 14.67 11.7 2.43 2.41 11.7
TU 0.88 1.01 4.46 6.63 0.46 0.73 3.56
SK 1.14 10.09 44.5 35.5 4.64 7.33 35.5
SA 2.32 7.56 17.65 14.08 1.84 2.9 14.08
PO 0.98 1.79 7.89 12.81 0.82 4.11 6.29
PN 1.32 1.63 7.21 5.75 20.81 1.18 5.75
NG 6.97 6.71 29.58 23.6 3.09 4.87 23.6
NAF 1.74 2.48 10.94 8.72 1.14 1.8 19.39
KE 1.38 0.81 2.95 2.35 0.31 0.48 2.35
JP 0.57 1.79 175 6.29 2.48 1.74 6.29
IT 0.08 4.64 2.95 13.2 0.31 1.25 4.22
IN 0.5 0.67 2.95 2.35 31.5 0.48 2.35
HK 11.16 1.63 7.21 5.75 0.75 1.18 5.75
GH 0.45 155.45 12.54 10 1.31 2.06 10
GE 1.14 10.09 44.5 35.5 4.64 7.33 35.5
FR 1.14 10.09 44.5 35.5 4.64 7.33 35.5
CO 1.15 2.79 8.7 6.94 0.91 1.43 6.94
CH 8.04 0.75 3.33 2.66 0.86 0.55 2.66
BR 1.15 4.51 12.54 10 1.31 2.06 10
BO 0.5 1.63 7.21 5.75 2.19 16.23 5.75
AU 0.24 0.91 4.01 3.2 0.42 2.97 3.2
AR 1.39 2.2 9.69 7.73 1.38 1.59 7.73
AF 1.74 2.48 10.94 8.72 1.14 1.8 19.39

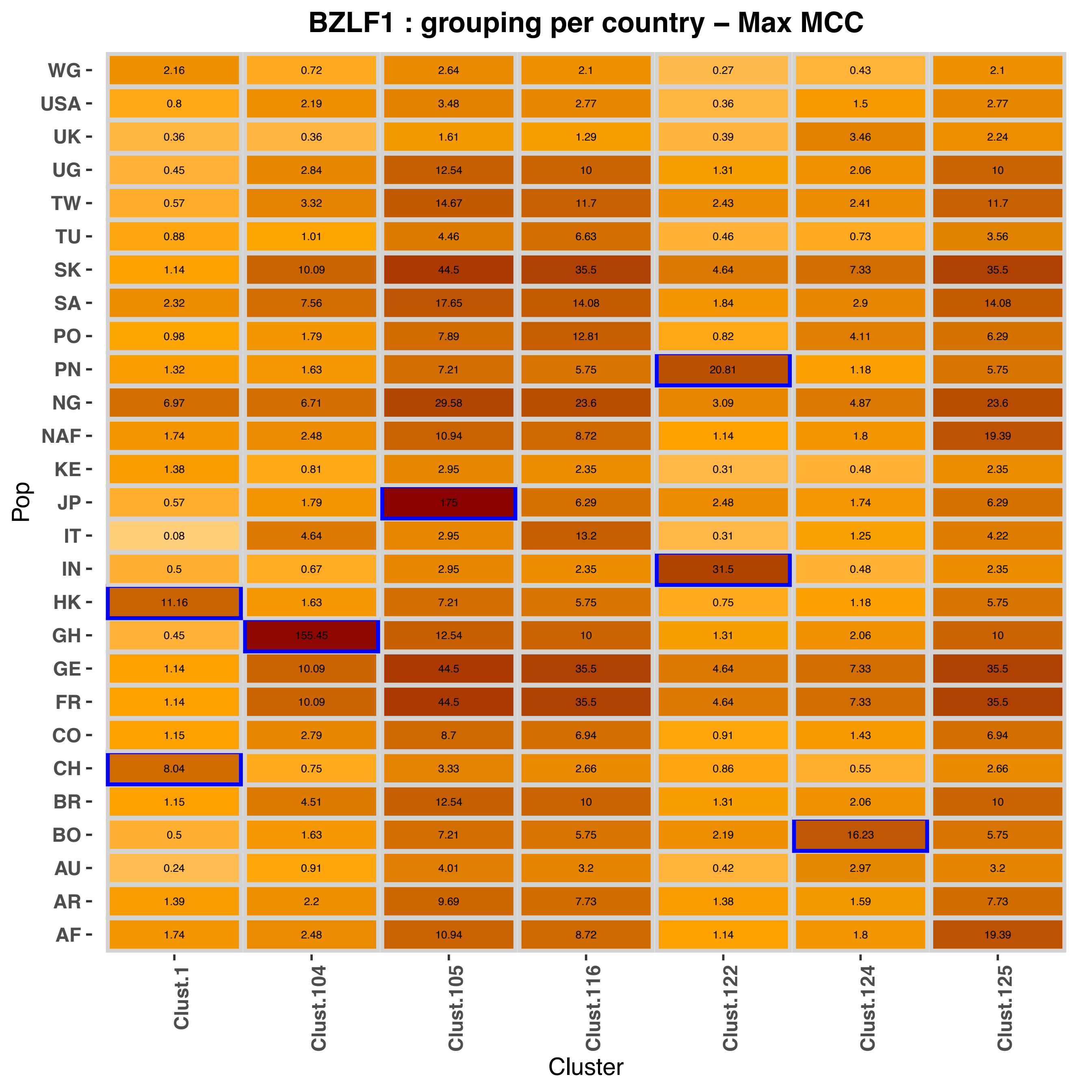
BZLF1 : grouping per country – Max MCC
Pop
Cluster
Clust.1
Clust.104
Clust.105
Clust.116
Clust.122
Clust.124
Clust.125
WG 2.16 0.72 2.64 2.1 0.27 0.43 2.1
USA 0.8 2.19 3.48 2.77 0.36 1.5 2.77
UK 0.36 0.36 1.61 1.29 0.39 3.46 2.24
UG 0.45 2.84 12.54 10 1.31 2.06 10
TW 0.57 3.32 14.67 11.7 2.43 2.41 11.7
TU 0.88 1.01 4.46 6.63 0.46 0.73 3.56
SK 1.14 10.09 44.5 35.5 4.64 7.33 35.5
SA 2.32 7.56 17.65 14.08 1.84 2.9 14.08
PO 0.98 1.79 7.89 12.81 0.82 4.11 6.29
PN 1.32 1.63 7.21 5.75 20.81 1.18 5.75
NG 6.97 6.71 29.58 23.6 3.09 4.87 23.6
NAF 1.74 2.48 10.94 8.72 1.14 1.8 19.39
KE 1.38 0.81 2.95 2.35 0.31 0.48 2.35
JP 0.57 1.79 175 6.29 2.48 1.74 6.29
IT 0.08 4.64 2.95 13.2 0.31 1.25 4.22
IN 0.5 0.67 2.95 2.35 31.5 0.48 2.35
HK 11.16 1.63 7.21 5.75 0.75 1.18 5.75
GH 0.45 155.45 12.54 10 1.31 2.06 10
GE 1.14 10.09 44.5 35.5 4.64 7.33 35.5
FR 1.14 10.09 44.5 35.5 4.64 7.33 35.5
CO 1.15 2.79 8.7 6.94 0.91 1.43 6.94
CH 8.04 0.75 3.33 2.66 0.86 0.55 2.66
BR 1.15 4.51 12.54 10 1.31 2.06 10
BO 0.5 1.63 7.21 5.75 2.19 16.23 5.75
AU 0.24 0.91 4.01 3.2 0.42 2.97 3.2
AR 1.39 2.2 9.69 7.73 1.38 1.59 7.73
AF 1.74 2.48 10.94 8.72 1.14 1.8 19.39

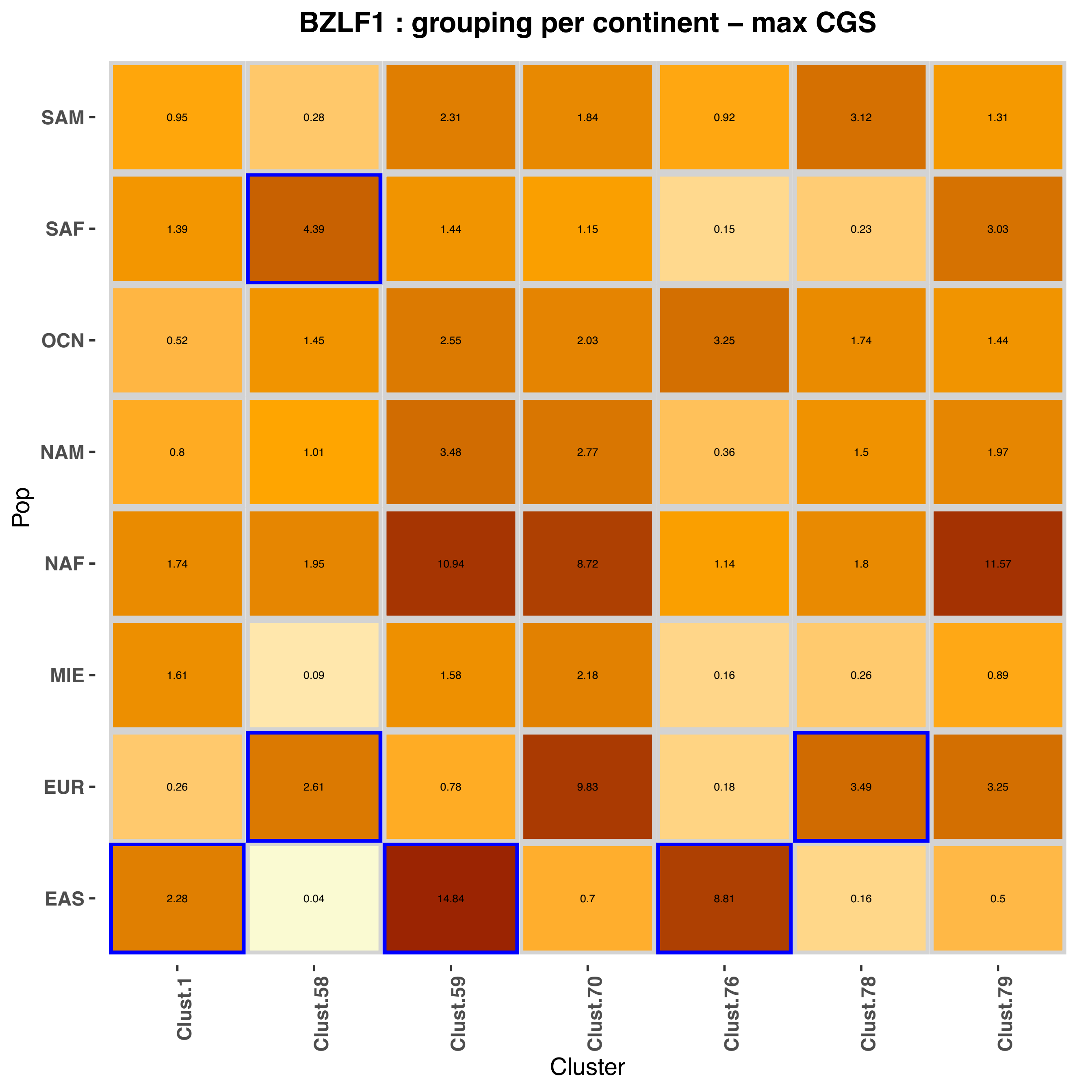
BZLF1 : grouping per continent – max CGS
SAM
SAF
OCN
NAM
NAF
MIE
EUR
EAS
Pop
Clust.1
Clust.58
Clust.59
Clust.70
Clust.76
Clust.78
Clust.79
Cluster
0.95
0.28
2.31
1.84
0.92
3.12
1.31
1.39
4.39
1.44
1.15
0.15
0.23
3.03
0.52
1.45
2.55
2.03
3.25
1.74
1.44
0.8
1.01
3.48
2.77
0.36
1.5
1.97
1.74
1.95
10.94
8.72
1.14
1.8
11.57
1.61
0.09
1.58
2.18
0.16
0.26
0.89
0.26
2.61
0.78
9.83
0.18
3.49
3.25
2.28
0.04
14.84
0.7
8.81
0.16
0.5

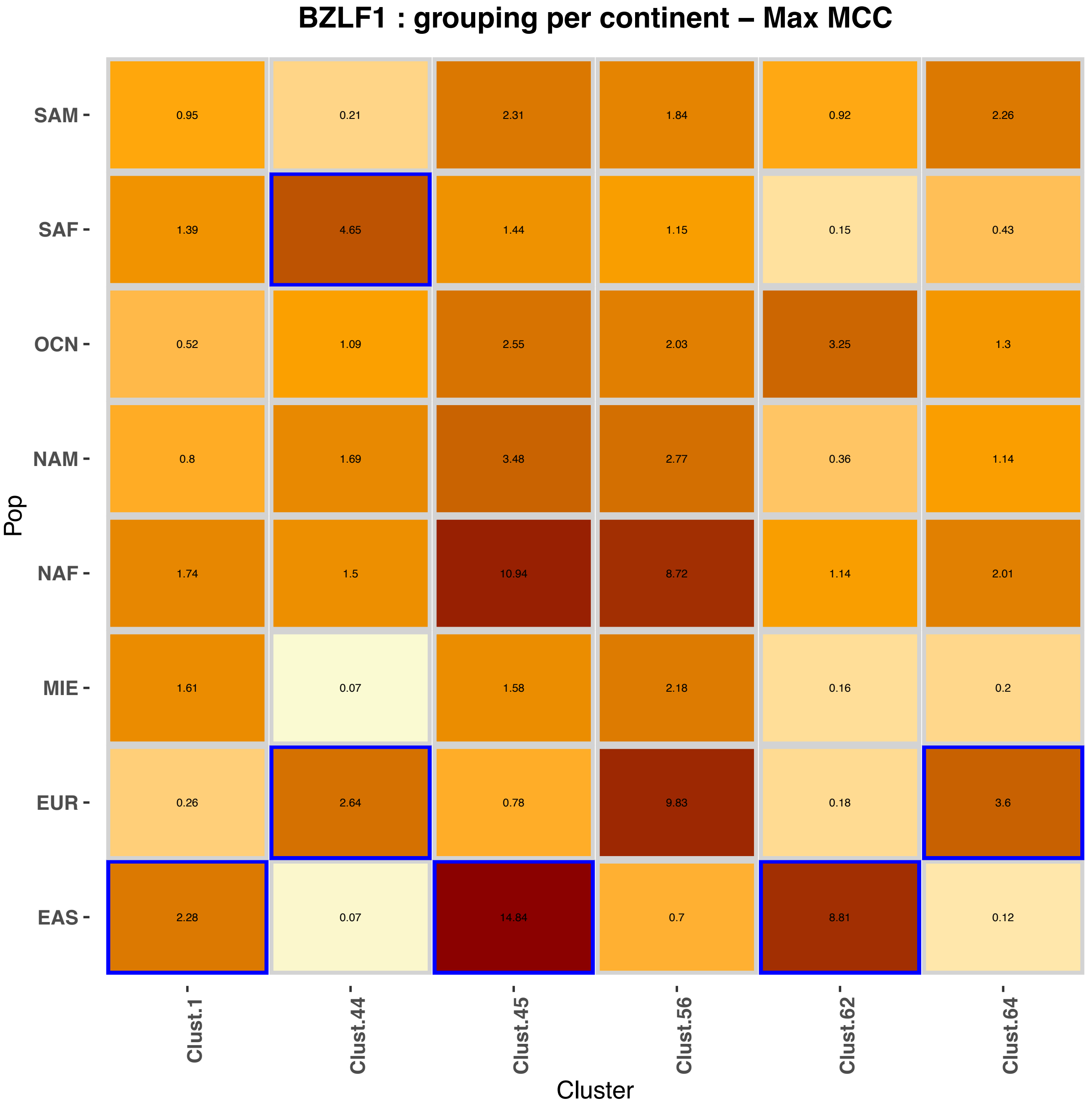

BZLF1 : grouping per continent – Max MCC
Pop
SAM
SAF
OCN
NAM
NAF
MIE
EUR
EAS
0.95
0.21
2.31
1.84
0.92
2.26
1.39
4.65
1.44
1.15
0.15
0.43
0.52
1.09
2.55
2.03
3.25
1.3
0.8
1.69
3.48
2.77
0.36
1.14
1.74
1.5
10.94
8.72
1.14
2.01
1.61
0.07
1.58
2.18
0.16
0.2
0.26
2.64
0.78
9.83
0.18
3.6
2.28
0.07
14.84
0.7
8.81
0.12
Clust.1
Clust.44
Clust.45
Clust.56
Clust.62
Clust.64
Cluster

EBER–1 : grouping per country – max CGS

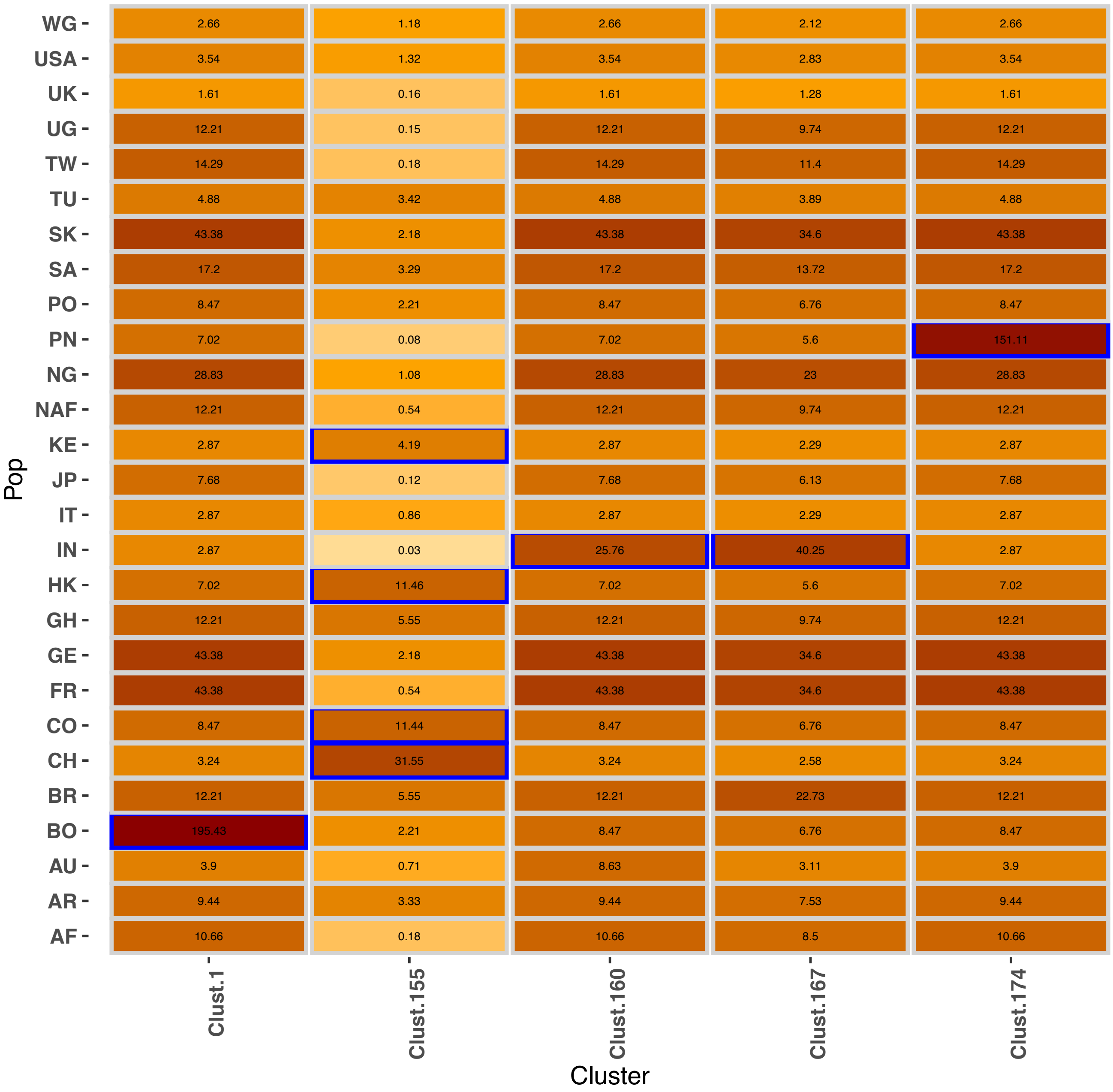

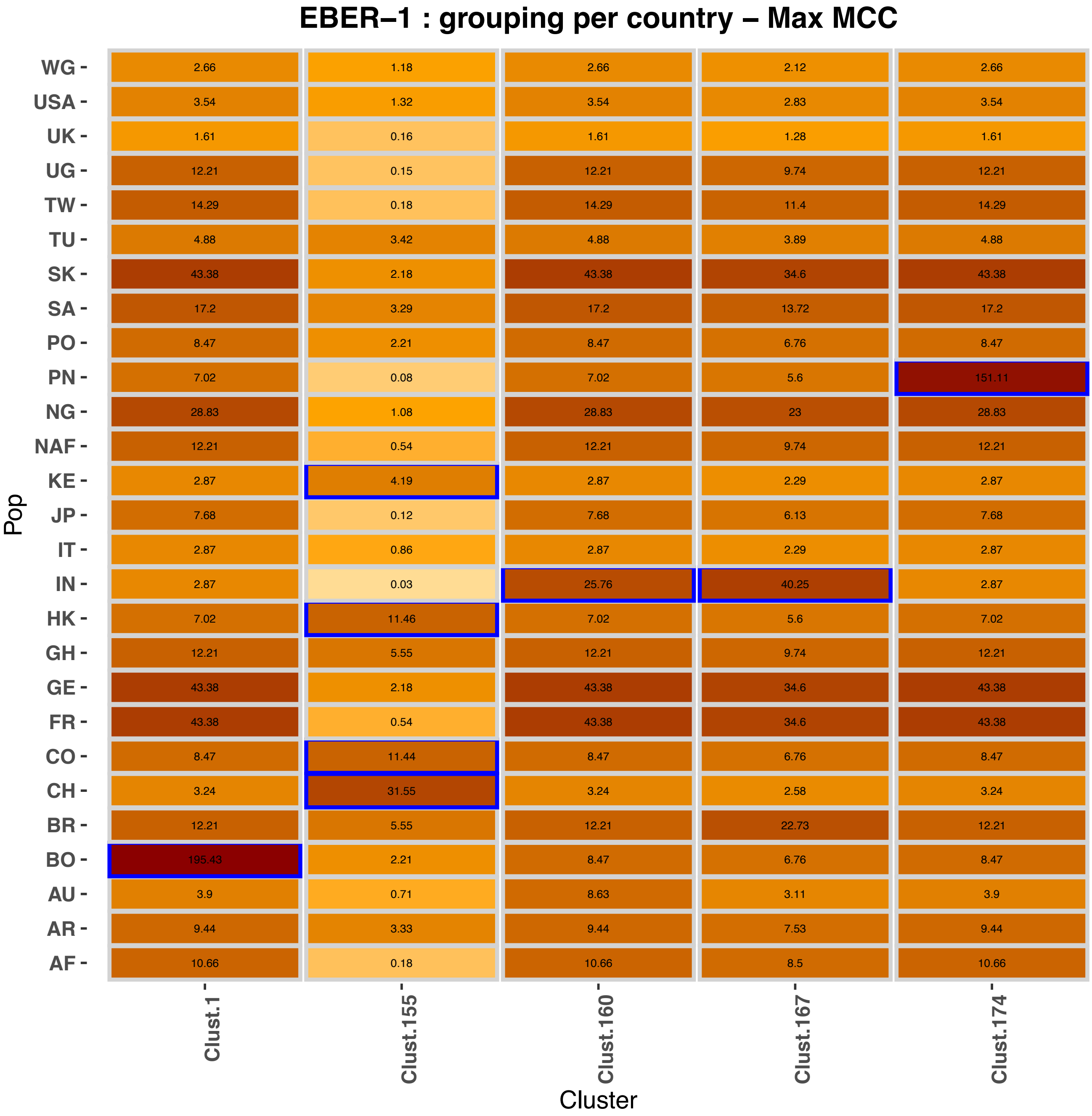
EBER–1 : grouping per country – Max MCC
Pop
Cluster
WG
USA
UK
UG
TW
TU
SK
SA
PO
PN
NG
NAF
KE
JP
IT
IN
HK
GH
GE
FR
CO
CH
BR
BO
AU
AR
AF
Clust.1
Clust.155
Clust.160
Clust.167
Clust.174

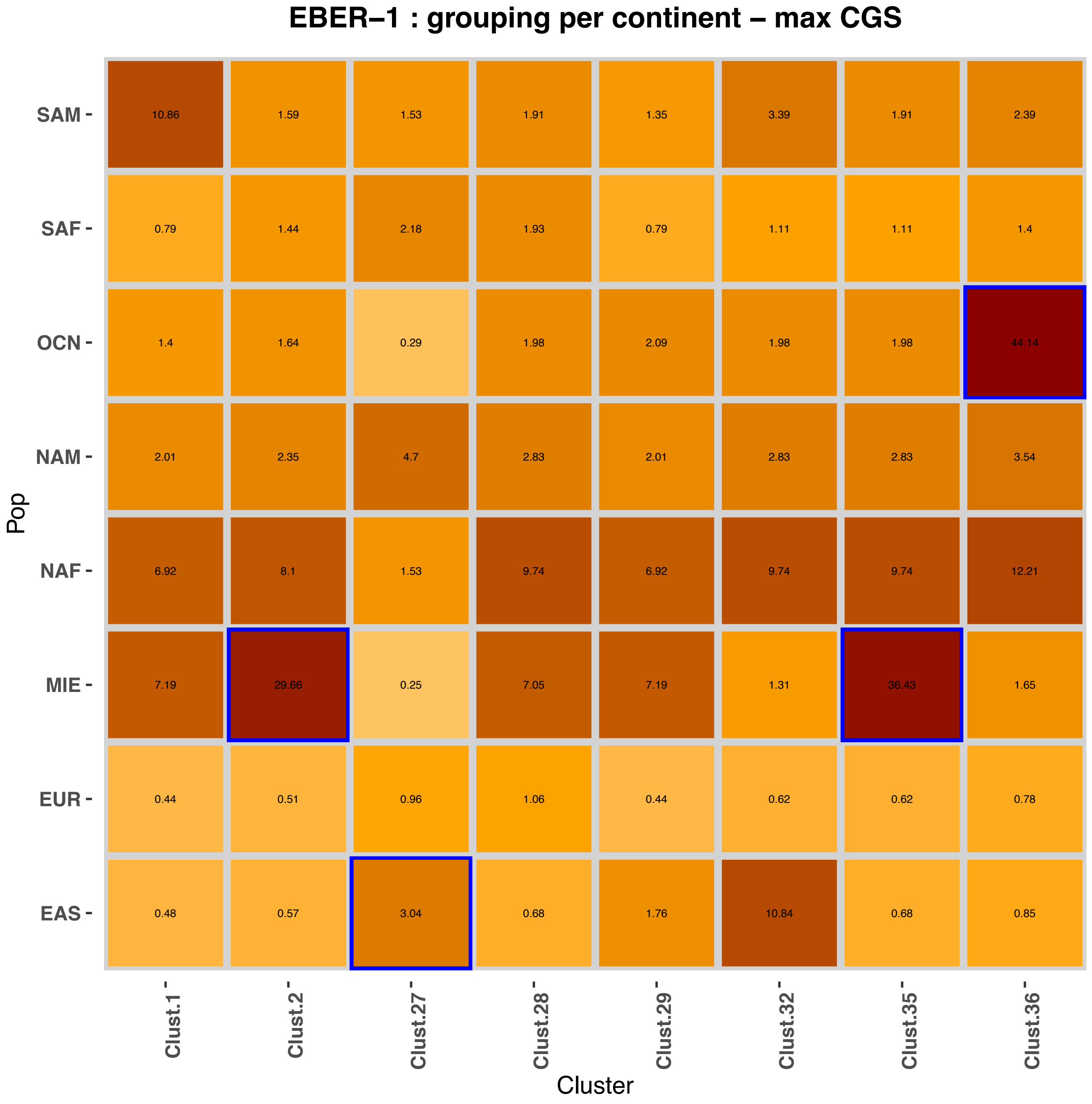
EBER–1 : grouping per continent – max CGS
Pop
SAM
SAF
OCN
NAM
NAF
MIE
EUR
EAS
Cluster
Clust.1
Clust.2
Clust.27
Clust.28
Clust.29
Clust.32
Clust.35
Clust.36
10.86 1.59 1.53 1.91 1.35 3.39 1.91 2.39
0.79 1.44 2.18 1.93 0.79 1.11 1.11 1.4
1.4 1.64 0.29 1.98 2.09 1.98 1.98 44.14
2.01 2.35 4.7 2.83 2.01 2.83 2.83 3.54
6.92 8.1 1.53 9.74 6.92 9.74 9.74 12.21
7.19 29.66 0.25 7.05 7.19 1.31 36.43 1.65
0.44 0.51 0.96 1.06 0.44 0.62 0.62 0.78
0.48 0.57 3.04 0.68 1.76 10.84 0.68 0.85

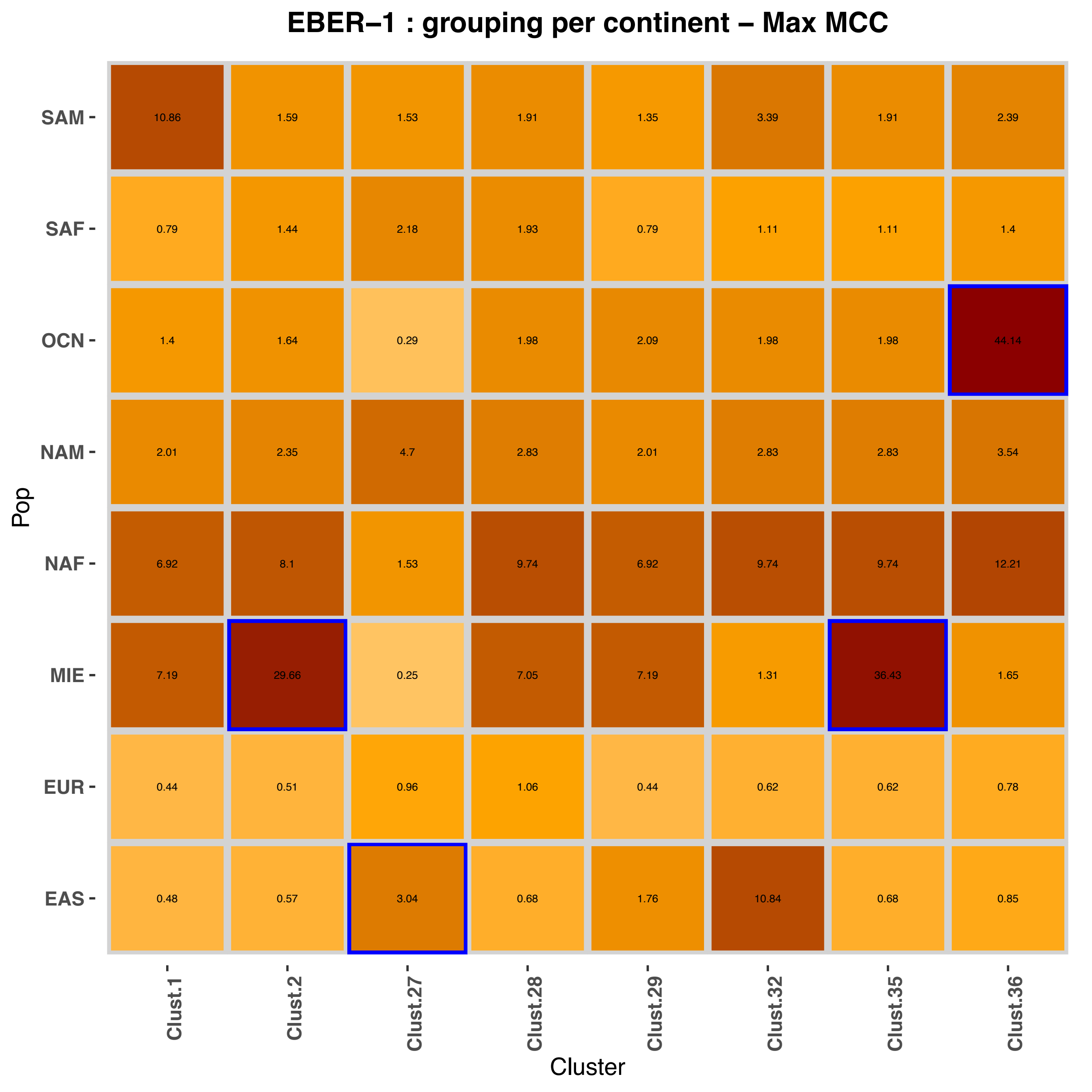
EBER–1 : grouping per continent – Max MCC
Pop
Cluster
SAM
SAF
OCN
NAM
NAF
MIE
EUR
EAS
Clust.1
Clust.2
Clust.27
Clust.28
Clust.29
Clust.32
Clust.35
Clust.36
SAM: 10.86 1.59 1.53 1.91 1.35 3.39 1.91 2.39
SAF: 0.79 1.44 2.18 1.93 0.79 1.11 1.11 1.4
OCN: 1.4 1.64 0.29 1.98 2.09 1.98 1.98 44.14
NAM: 2.01 2.35 4.7 2.83 2.01 2.83 2.83 3.54
NAF: 6.92 8.1 1.53 9.74 6.92 9.74 9.74 12.21
MIE: 7.19 29.66 0.25 7.05 7.19 1.31 36.43 1.65
EUR: 0.44 0.51 0.96 1.06 0.44 0.62 0.62 0.78
EAS: 0.48 0.57 3.04 0.68 1.76 10.84 0.68 0.85

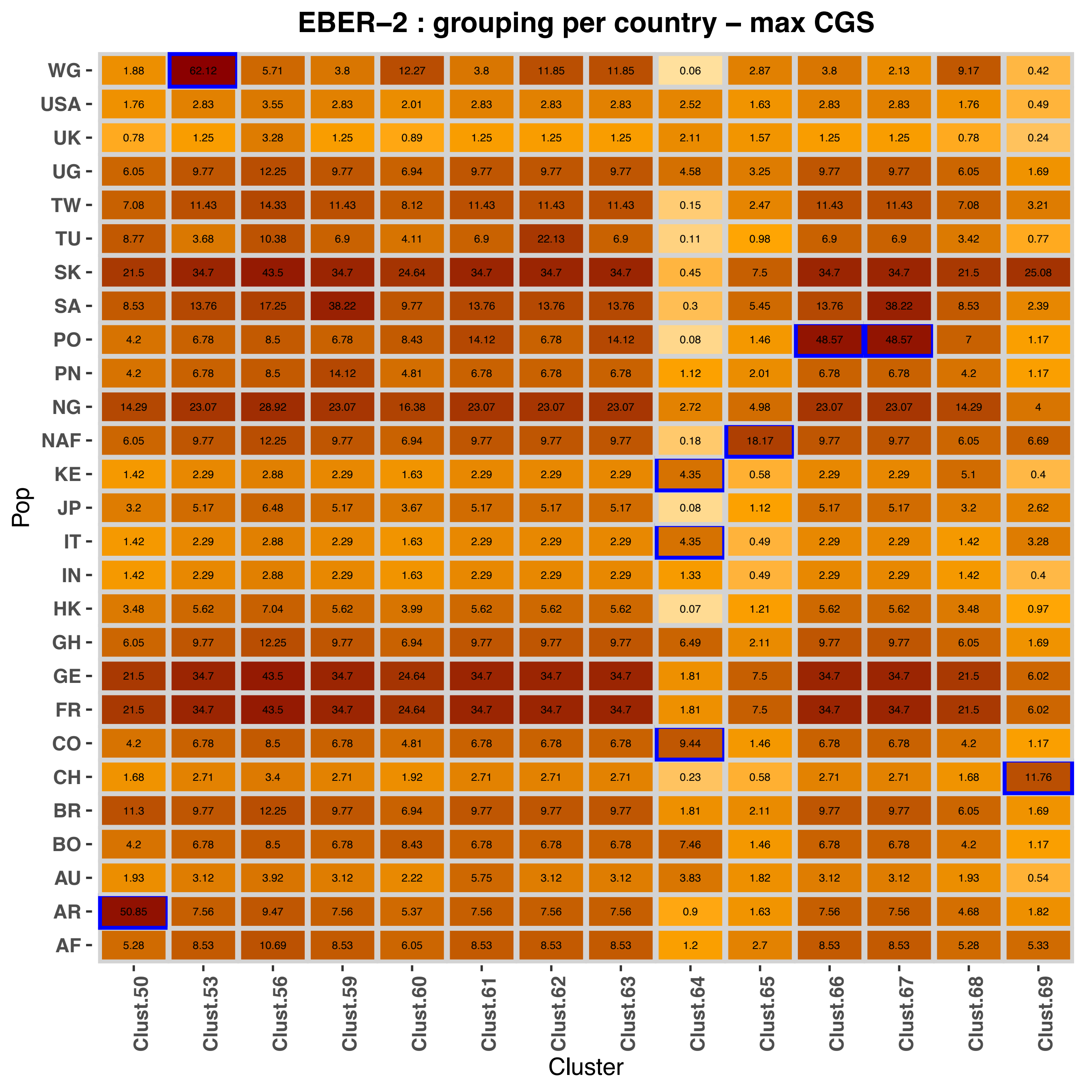
EBER–2 : grouping per country – max CGS
Pop
Cluster
Clust.50 Clust.53 Clust.56 Clust.59 Clust.60 Clust.61 Clust.62 Clust.63 Clust.64 Clust.65 Clust.66 Clust.67 Clust.68 Clust.69
WG 1.88 62.12 5.71 3.8 12.27 3.8 11.85 11.85 0.06 2.87 3.8 2.13 9.17 0.42
USA 1.76 2.83 3.55 2.83 2.01 2.83 2.83 2.83 2.52 1.63 2.83 2.83 1.76 0.49
UK 0.78 1.25 3.28 1.25 0.89 1.25 1.25 1.25 2.11 1.57 1.25 1.25 0.78 0.24
UG 6.05 9.77 12.25 9.77 6.94 9.77 9.77 9.77 4.58 3.25 9.77 9.77 6.05 1.69
TW 7.08 11.43 14.33 11.43 8.12 11.43 11.43 11.43 0.15 2.47 11.43 11.43 7.08 3.21
TU 8.77 3.68 10.38 6.9 4.11 6.9 22.13 6.9 0.11 0.98 6.9 6.9 3.42 0.77
SK 21.5 34.7 43.5 34.7 24.64 34.7 34.7 34.7 0.45 7.5 34.7 34.7 21.5 25.08
SA 8.53 13.76 17.25 38.22 9.77 13.76 13.76 13.76 0.3 5.45 13.76 38.22 8.53 2.39
PO 4.2 6.78 8.5 6.78 8.43 14.12 6.78 14.12 0.08 1.46 48.57 48.57 7 1.17
PN 4.2 6.78 8.5 14.12 4.81 6.78 6.78 6.78 1.12 2.01 6.78 6.78 4.2 1.17
NG 14.29 23.07 28.92 23.07 16.38 23.07 23.07 23.07 2.72 4.98 23.07 23.07 14.29 4
NAF 6.05 9.77 12.25 9.77 6.94 9.77 9.77 9.77 0.18 18.17 9.77 9.77 6.05 6.69
KE 1.42 2.29 2.88 2.29 1.63 2.29 2.29 2.29 4.35 0.58 2.29 2.29 5.1 0.4
JP 3.2 5.17 6.48 5.17 3.67 5.17 5.17 5.17 0.08 1.12 5.17 5.17 3.2 2.62
IT 1.42 2.29 2.88 2.29 1.63 2.29 2.29 2.29 4.35 0.49 2.29 2.29 1.42 3.28
IN 1.42 2.29 2.88 2.29 1.63 2.29 2.29 2.29 1.33 0.49 2.29 2.29 1.42 0.4
HK 3.48 5.62 7.04 5.62 3.99 5.62 5.62 5.62 0.07 1.21 5.62 5.62 3.48 0.97
GH 6.05 9.77 12.25 9.77 6.94 9.77 9.77 9.77 6.49 2.11 9.77 9.77 6.05 1.69
GE 21.5 34.7 43.5 34.7 24.64 34.7 34.7 34.7 1.81 7.5 34.7 34.7 21.5 6.02
FR 21.5 34.7 43.5 34.7 24.64 34.7 34.7 34.7 1.81 7.5 34.7 34.7 21.5 6.02
CO 4.2 6.78 8.5 6.78 4.81 6.78 6.78 6.78 9.44 1.46 6.78 6.78 4.2 1.17
CH 1.68 2.71 3.4 2.71 1.92 2.71 2.71 2.71 0.23 0.58 2.71 2.71 1.68 11.76
BR 11.3 9.77 12.25 9.77 6.94 9.77 9.77 9.77 1.81 2.11 9.77 9.77 6.05 1.69
BO 4.2 6.78 8.5 6.78 8.43 6.78 6.78 6.78 7.46 1.46 6.78 6.78 4.2 1.17
AU 1.93 3.12 3.92 3.12 2.22 5.75 3.12 3.12 3.83 1.82 3.12 3.12 1.93 0.54
AR 50.85 7.56 9.47 7.56 5.37 7.56 7.56 7.56 0.9 1.63 7.56 7.56 4.68 1.82
AF 5.28 8.53 10.69 8.53 6.05 8.53 8.53 8.53 1.2 2.7 8.53 8.53 5.28 5.33

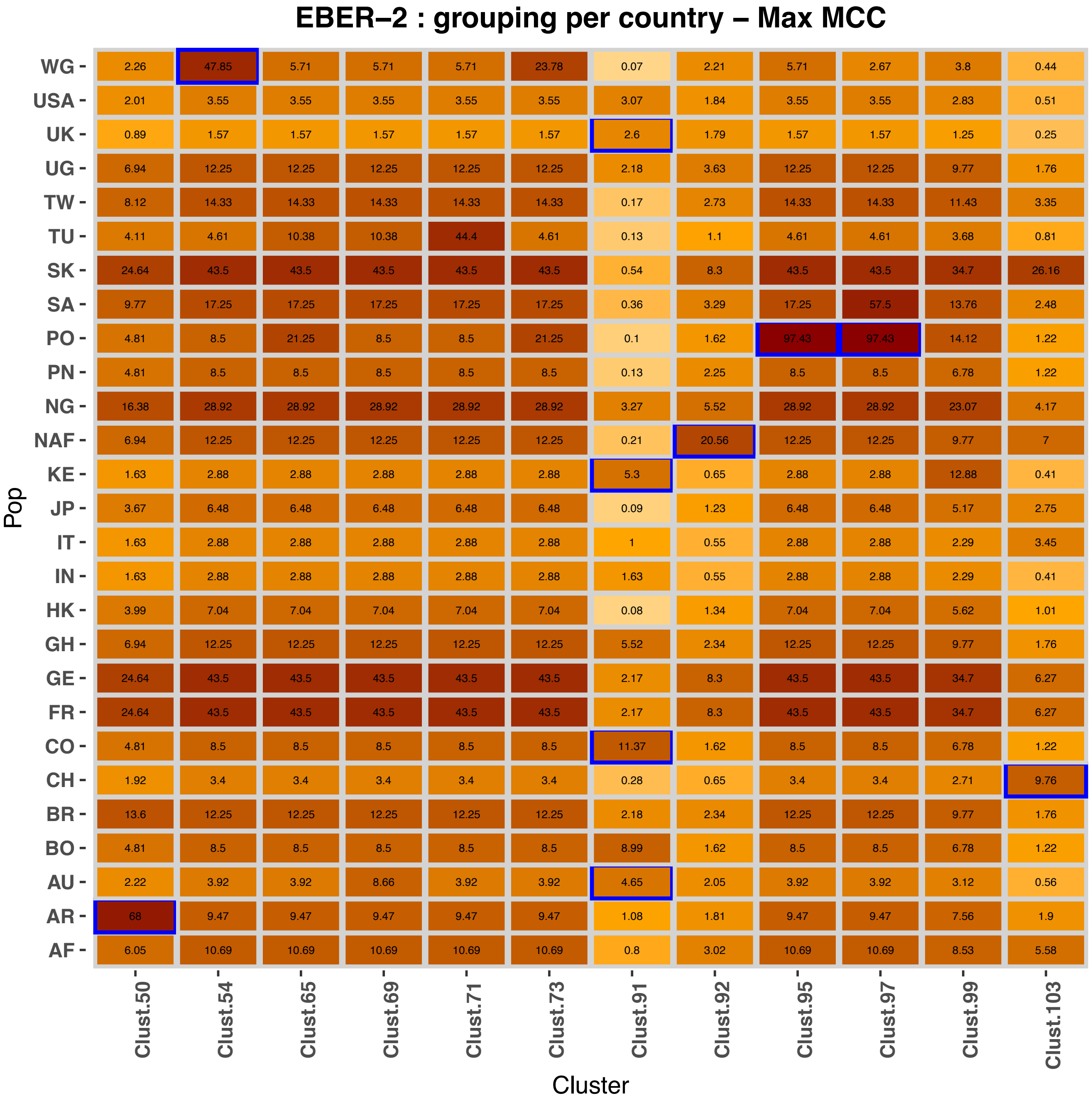
EBER–2 : grouping per country – Max MCC
Pop
Cluster
Clust.50 Clust.54 Clust.65 Clust.69 Clust.71 Clust.73 Clust.91 Clust.92 Clust.95 Clust.97 Clust.99 Clust.103
WG 2.26 47.85 5.71 5.71 5.71 23.78 0.07 2.21 5.71 2.67 3.8 0.44
USA 2.01 3.55 3.55 3.55 3.55 3.55 3.07 1.84 3.55 3.55 2.83 0.51
UK 0.89 1.57 1.57 1.57 1.57 1.57 2.6 1.79 1.57 1.57 1.25 0.25
UG 6.94 12.25 12.25 12.25 12.25 12.25 2.18 3.63 12.25 12.25 9.77 1.76
TW 8.12 14.33 14.33 14.33 14.33 14.33 0.17 2.73 14.33 14.33 11.43 3.35
TU 4.11 4.61 10.38 10.38 44.4 4.61 0.13 1.1 4.61 4.61 3.68 0.81
SK 24.64 43.5 43.5 43.5 43.5 43.5 0.54 8.3 43.5 43.5 34.7 26.16
SA 9.77 17.25 17.25 17.25 17.25 17.25 0.36 3.29 17.25 57.5 13.76 2.48
PO 4.81 8.5 21.25 8.5 8.5 21.25 0.1 1.62 97.43 97.43 14.12 1.22
PN 4.81 8.5 8.5 8.5 8.5 8.5 0.13 2.25 8.5 8.5 6.78 1.22
NG 16.38 28.92 28.92 28.92 28.92 28.92 3.27 5.52 28.92 28.92 23.07 4.17
NAF 6.94 12.25 12.25 12.25 12.25 12.25 0.21 20.56 12.25 12.25 9.77 7
KE 1.63 2.88 2.88 2.88 2.88 2.88 5.3 0.65 2.88 2.88 12.88 0.41
JP 3.67 6.48 6.48 6.48 6.48 6.48 0.09 1.23 6.48 6.48 5.17 2.75
IT 1.63 2.88 2.88 2.88 2.88 2.88 1 0.55 2.88 2.88 2.29 3.45
IN 1.63 2.88 2.88 2.88 2.88 2.88 1.63 0.55 2.88 2.88 2.29 0.41
HK 3.99 7.04 7.04 7.04 7.04 7.04 0.08 1.34 7.04 7.04 5.62 1.01
GH 6.94 12.25 12.25 12.25 12.25 12.25 5.52 2.34 12.25 12.25 9.77 1.76
GE 24.64 43.5 43.5 43.5 43.5 43.5 2.17 8.3 43.5 43.5 34.7 6.27
FR 24.64 43.5 43.5 43.5 43.5 43.5 2.17 8.3 43.5 43.5 34.7 6.27
CO 4.81 8.5 8.5 8.5 8.5 8.5 11.37 1.62 8.5 8.5 6.78 1.22
CH 1.92 3.4 3.4 3.4 3.4 3.4 0.28 0.65 3.4 3.4 2.71 9.76
BR 13.6 12.25 12.25 12.25 12.25 12.25 2.18 2.34 12.25 12.25 9.77 1.76
BO 4.81 8.5 8.5 8.5 8.5 8.5 8.99 1.62 8.5 8.5 6.78 1.22
AU 2.22 3.92 3.92 8.66 3.92 3.92 4.65 2.05 3.92 3.92 3.12 0.56
AR 68 9.47 9.47 9.47 9.47 9.47 1.08 1.81 9.47 9.47 7.56 1.9
AF 6.05 10.69 10.69 10.69 10.69 10.69 0.8 3.02 10.69 10.69 8.53 5.58

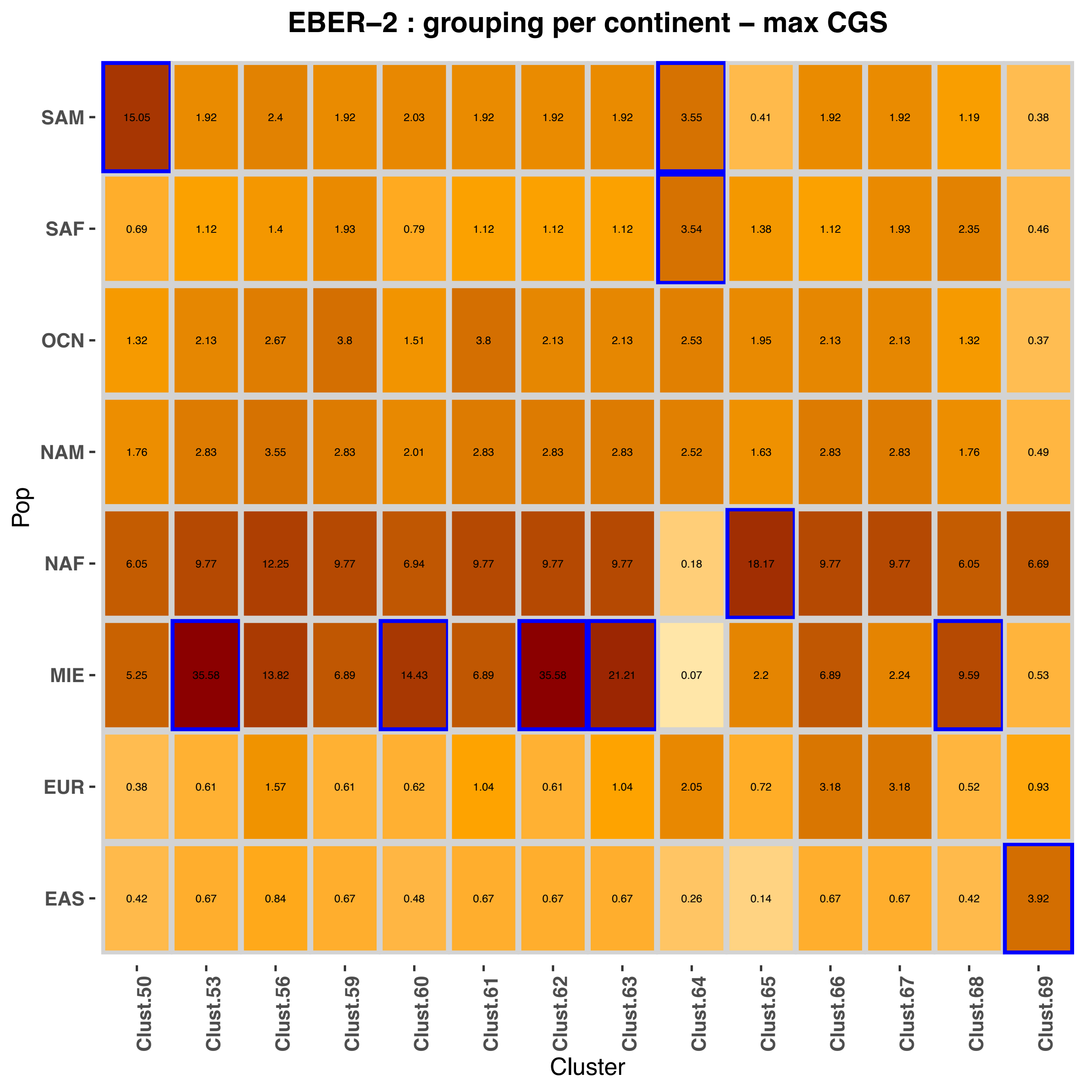
EBER–2 : grouping per continent – max CGS
Pop
SAM
SAF
OCN
NAM
NAF
MIE
EUR
EAS
15.05 1.92 2.4 1.92 2.03 1.92 1.92 1.92 3.55 0.41 1.92 1.92 1.19 0.38
0.69 1.12 1.4 1.93 0.79 1.12 1.12 1.12 3.54 1.38 1.12 1.93 2.35 0.46
1.32 2.13 2.67 3.8 1.51 3.8 2.13 2.13 2.53 1.95 2.13 2.13 1.32 0.37
1.76 2.83 3.55 2.83 2.01 2.83 2.83 2.83 2.52 1.63 2.83 2.83 1.76 0.49
6.05 9.77 12.25 9.77 6.94 9.77 9.77 9.77 0.18 18.17 9.77 9.77 6.05 6.69
5.25 35.58 13.82 6.89 14.43 6.89 35.58 21.21 0.07 2.2 6.89 2.24 9.59 0.53
0.38 0.61 1.57 0.61 0.62 1.04 0.61 1.04 2.05 0.72 3.18 3.18 0.52 0.93
0.42 0.67 0.84 0.67 0.48 0.67 0.67 0.67 0.26 0.14 0.67 0.67 0.42 3.92
Clust.50
Clust.53
Clust.56
Clust.59
Clust.60
Clust.61
Clust.62
Clust.63
Clust.64
Clust.65
Clust.66
Clust.67
Clust.68
Clust.69
Cluster

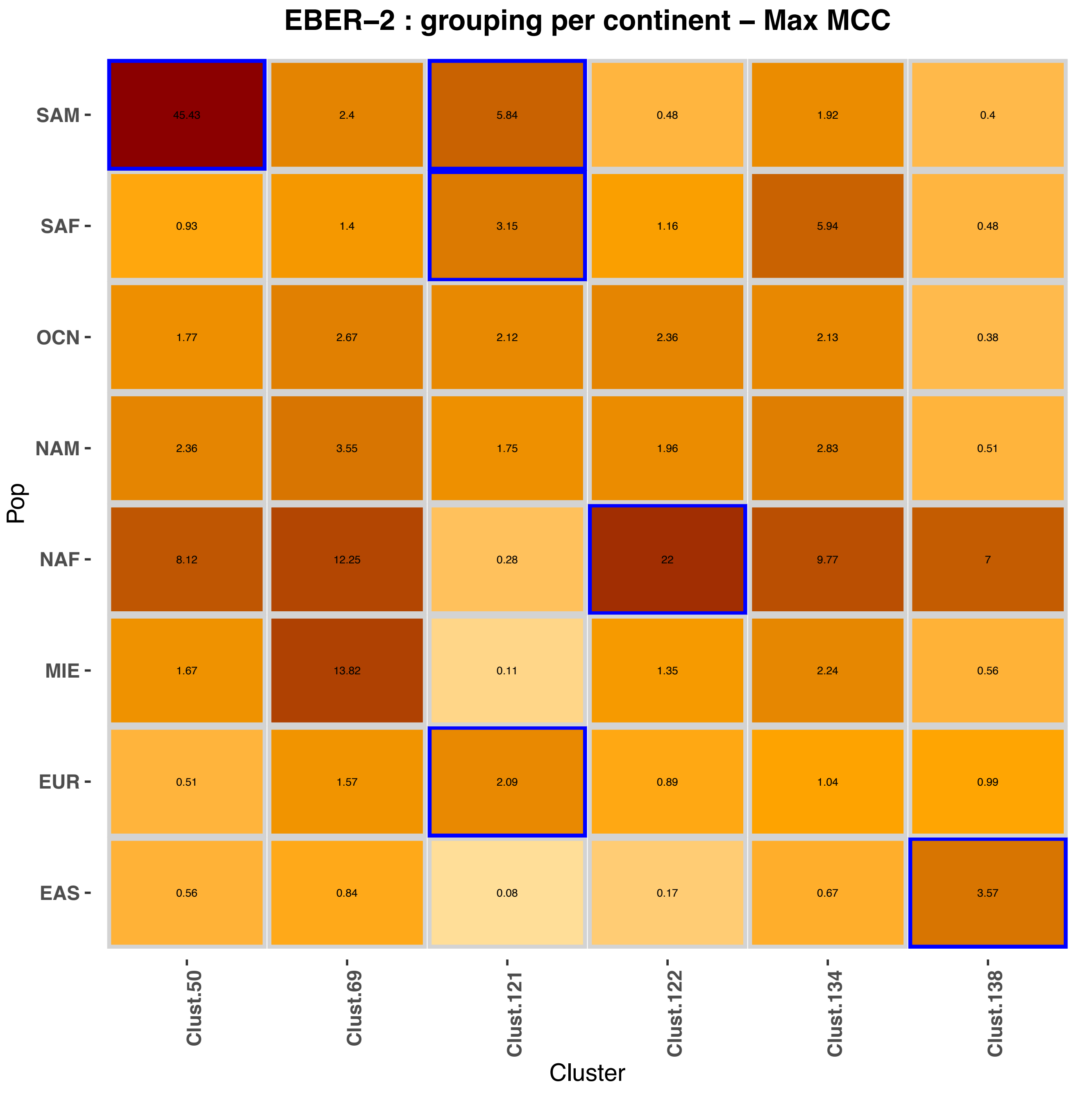
EBER–2 : grouping per continent – Max MCC
Pop
SAM
SAF
OCN
NAM
NAF
MIE
EUR
EAS
45.43 2.4 5.84 0.48 1.92 0.4
0.93 1.4 3.15 1.16 5.94 0.48
1.77 2.67 2.12 2.36 2.13 0.38
2.36 3.55 1.75 1.96 2.83 0.51
8.12 12.25 0.28 22 9.77 7
1.67 13.82 0.11 1.35 2.24 0.56
0.51 1.57 2.09 0.89 1.04 0.99
0.56 0.84 0.08 0.17 0.67 3.57
Clust.50
Clust.69
Clust.121
Clust.122
Clust.134
Clust.138
Cluster

EBNA–1 : grouping per country – max CGS

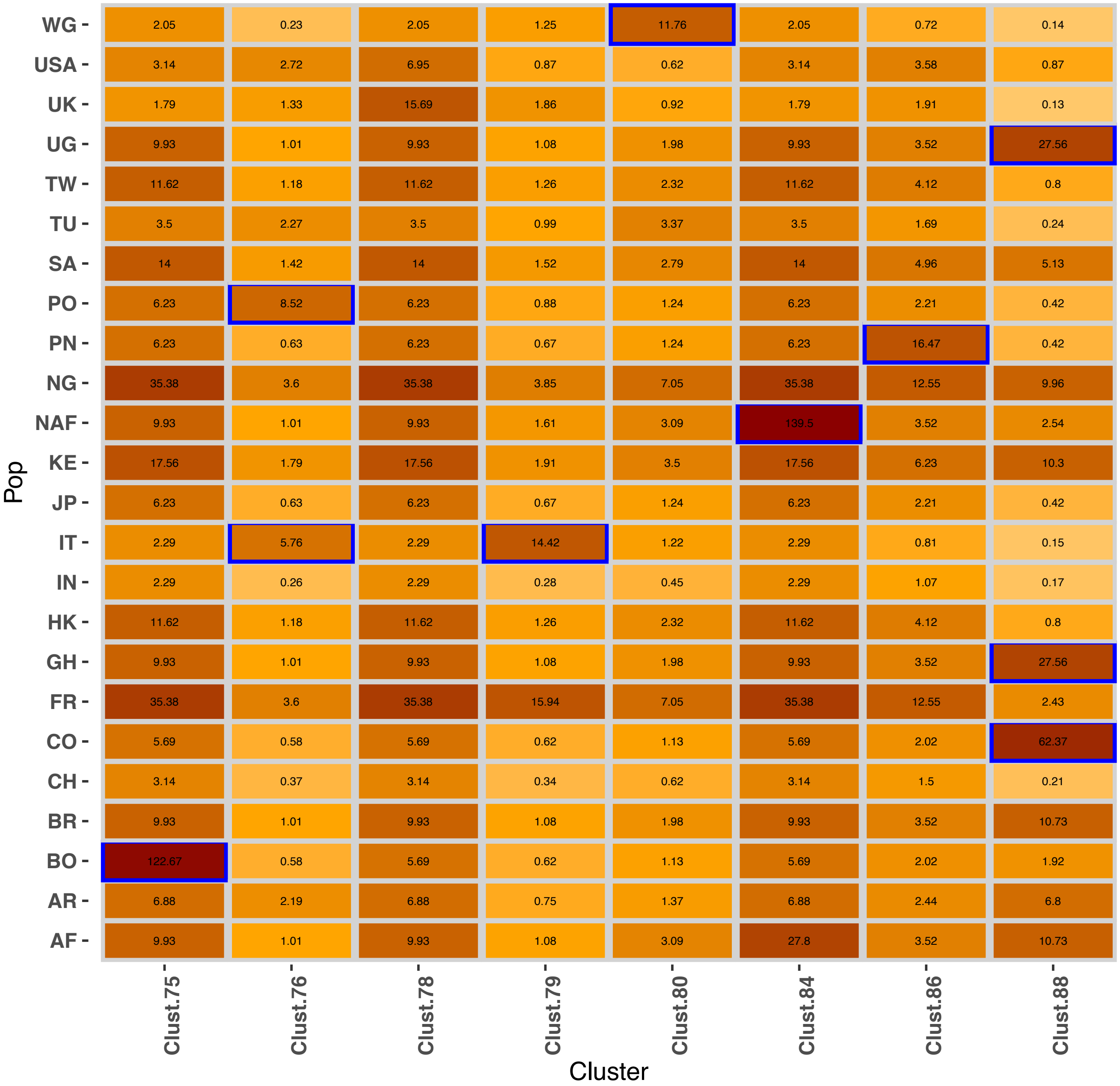

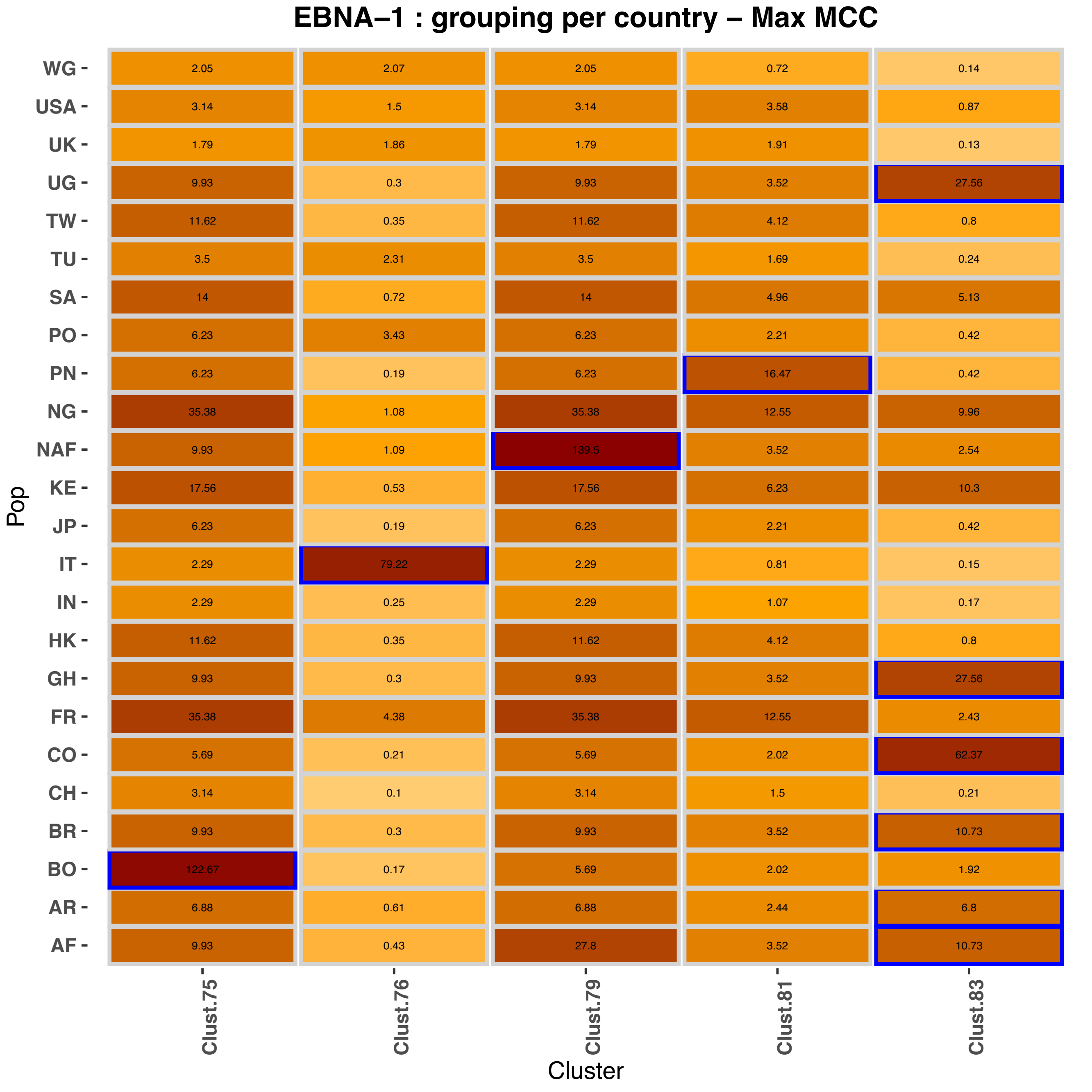
EBNA–1 : grouping per country – Max MCC
Pop
Cluster
Clust.75
Clust.76
Clust.79
Clust.81
Clust.83
WG 2.05 2.07 2.05 0.72 0.14
USA 3.14 1.5 3.14 3.58 0.87
UK 1.79 1.86 1.79 1.91 0.13
UG 9.93 0.3 9.93 3.52 27.56
TW 11.62 0.35 11.62 4.12 0.8
TU 3.5 2.31 3.5 1.69 0.24
SA 14 0.72 14 4.96 5.13
PO 6.23 3.43 6.23 2.21 0.42
PN 6.23 0.19 6.23 16.47 0.42
NG 35.38 1.08 35.38 12.55 9.96
NAF 9.93 1.09 139.5 3.52 2.54
KE 17.56 0.53 17.56 6.23 10.3
JP 6.23 0.19 6.23 2.21 0.42
IT 2.29 79.22 2.29 0.81 0.15
IN 2.29 0.25 2.29 1.07 0.17
HK 11.62 0.35 11.62 4.12 0.8
GH 9.93 0.3 9.93 3.52 27.56
FR 35.38 4.38 35.38 12.55 2.43
CO 5.69 0.21 5.69 2.02 62.37
CH 3.14 0.1 3.14 1.5 0.21
BR 9.93 0.3 9.93 3.52 10.73
BO 122.67 0.17 5.69 2.02 1.92
AR 6.88 0.61 6.88 2.44 6.8
AF 9.93 0.43 27.8 3.52 10.73

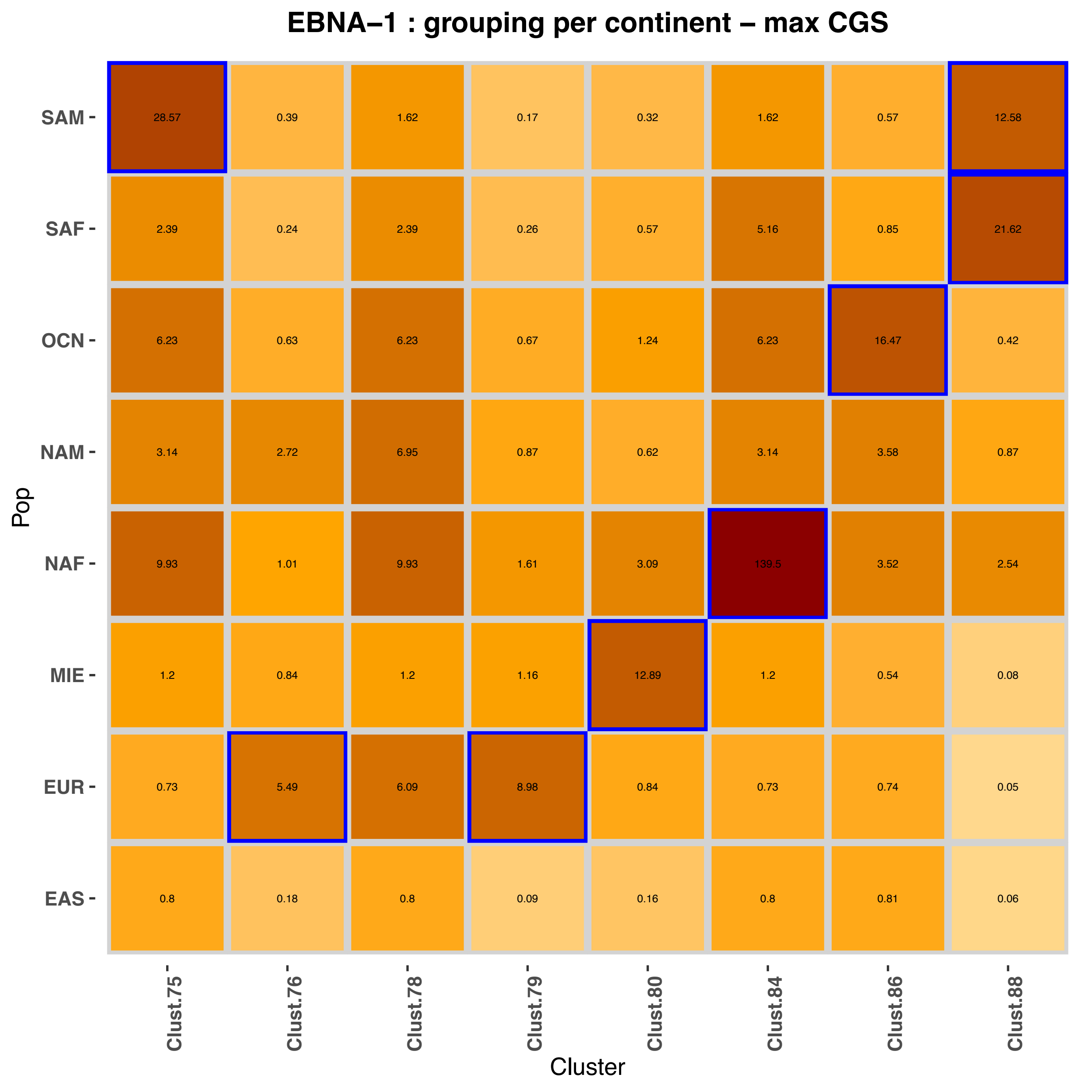

EBNA–1 : grouping per continent – max CGS
Pop
Cluster
SAM
SAF
OCN
NAM
NAF
MIE
EUR
EAS
Clust.75
Clust.76
Clust.78
Clust.79
Clust.80
Clust.84
Clust.86
Clust.88
28.57 0.39 1.62 0.17 0.32 1.62 0.57 12.58
2.39 0.24 2.39 0.26 0.57 5.16 0.85 21.62
6.23 0.63 6.23 0.67 1.24 6.23 16.47 0.42
3.14 2.72 6.95 0.87 0.62 3.14 3.58 0.87
9.93 1.01 9.93 1.61 3.09 139.5 3.52 2.54
1.2 0.84 1.2 1.16 12.89 1.2 0.54 0.08
0.73 5.49 6.09 8.98 0.84 0.73 0.74 0.05
0.8 0.18 0.8 0.09 0.16 0.8 0.81 0.06

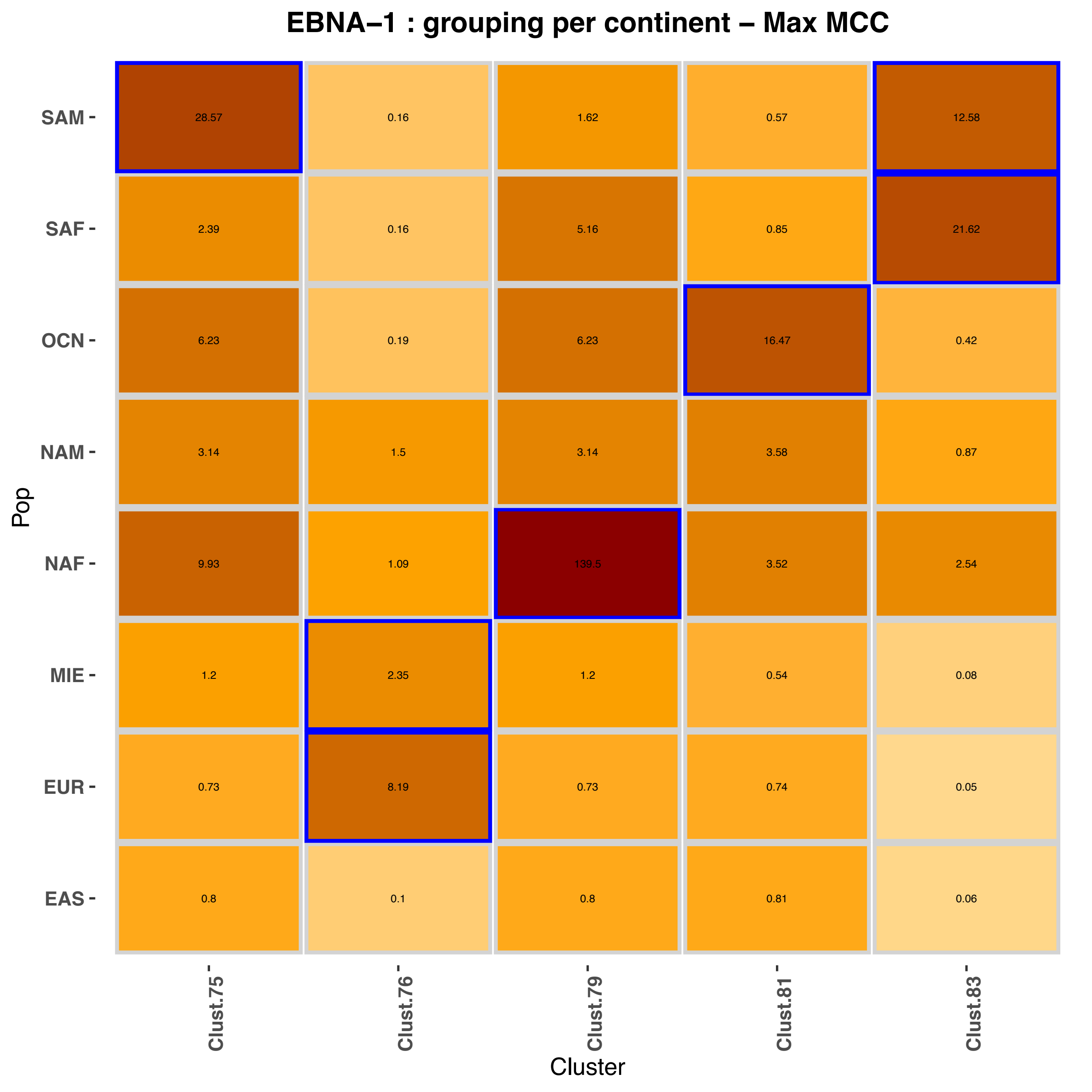

EBNA–1 : grouping per continent – Max MCC
Pop
SAM
SAF
OCN
NAM
NAF
MIE
EUR
EAS
Cluster
Clust.75
Clust.76
Clust.79
Clust.81
Clust.83
28.57
0.16
1.62
0.57
12.58
2.39
0.16
5.16
0.85
21.62
6.23
0.19
6.23
16.47
0.42
3.14
1.5
3.14
3.58
0.87
9.93
1.09
139.5
3.52
2.54
1.2
2.35
1.2
0.54
0.08
0.73
8.19
0.73
0.74
0.05
0.8
0.1
0.8
0.81
0.06

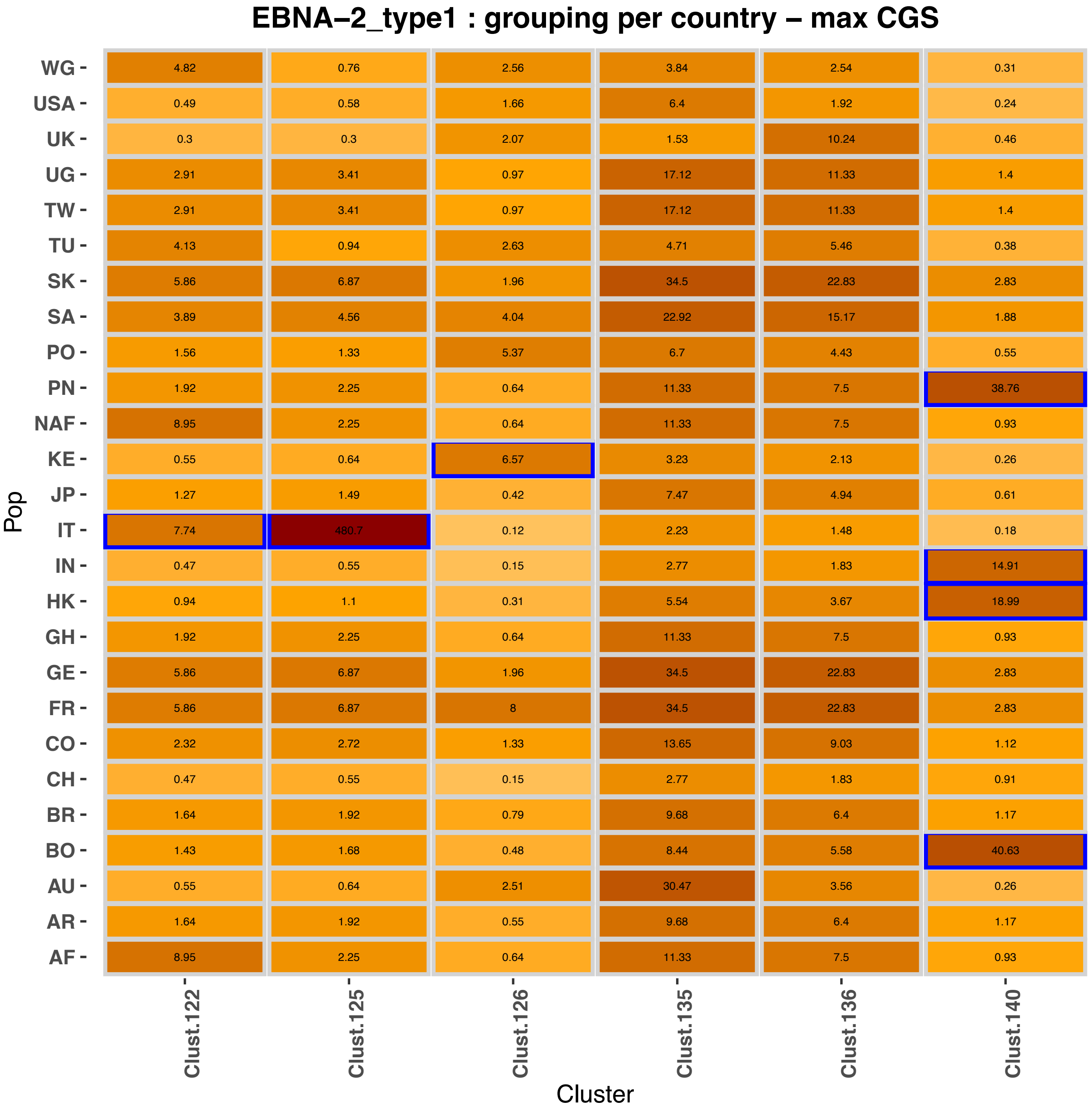
EBNA−2_type1 : grouping per country − max CGS
Pop
Cluster
Clust.122
Clust.125
Clust.126
Clust.135
Clust.136
Clust.140
WG 4.82 0.76 2.56 3.84 2.54 0.31
USA 0.49 0.58 1.66 6.4 1.92 0.24
UK 0.3 0.3 2.07 1.53 10.24 0.46
UG 2.91 3.41 0.97 17.12 11.33 1.4
TW 2.91 3.41 0.97 17.12 11.33 1.4
TU 4.13 0.94 2.63 4.71 5.46 0.38
SK 5.86 6.87 1.96 34.5 22.83 2.83
SA 3.89 4.56 4.04 22.92 15.17 1.88
PO 1.56 1.33 5.37 6.7 4.43 0.55
PN 1.92 2.25 0.64 11.33 7.5 38.76
NAF 8.95 2.25 0.64 11.33 7.5 0.93
KE 0.55 0.64 6.57 3.23 2.13 0.26
JP 1.27 1.49 0.42 7.47 4.94 0.61
IT 7.74 480.7 0.12 2.23 1.48 0.18
IN 0.47 0.55 0.15 2.77 1.83 14.91
HK 0.94 1.1 0.31 5.54 3.67 18.99
GH 1.92 2.25 0.64 11.33 7.5 0.93
GE 5.86 6.87 1.96 34.5 22.83 2.83
FR 5.86 6.87 8 34.5 22.83 2.83
CO 2.32 2.72 1.33 13.65 9.03 1.12
CH 0.47 0.55 0.15 2.77 1.83 0.91
BR 1.64 1.92 0.79 9.68 6.4 1.17
BO 1.43 1.68 0.48 8.44 5.58 40.63
AU 0.55 0.64 2.51 30.47 3.56 0.26
AR 1.64 1.92 0.55 9.68 6.4 1.17
AF 8.95 2.25 0.64 11.33 7.5 0.93

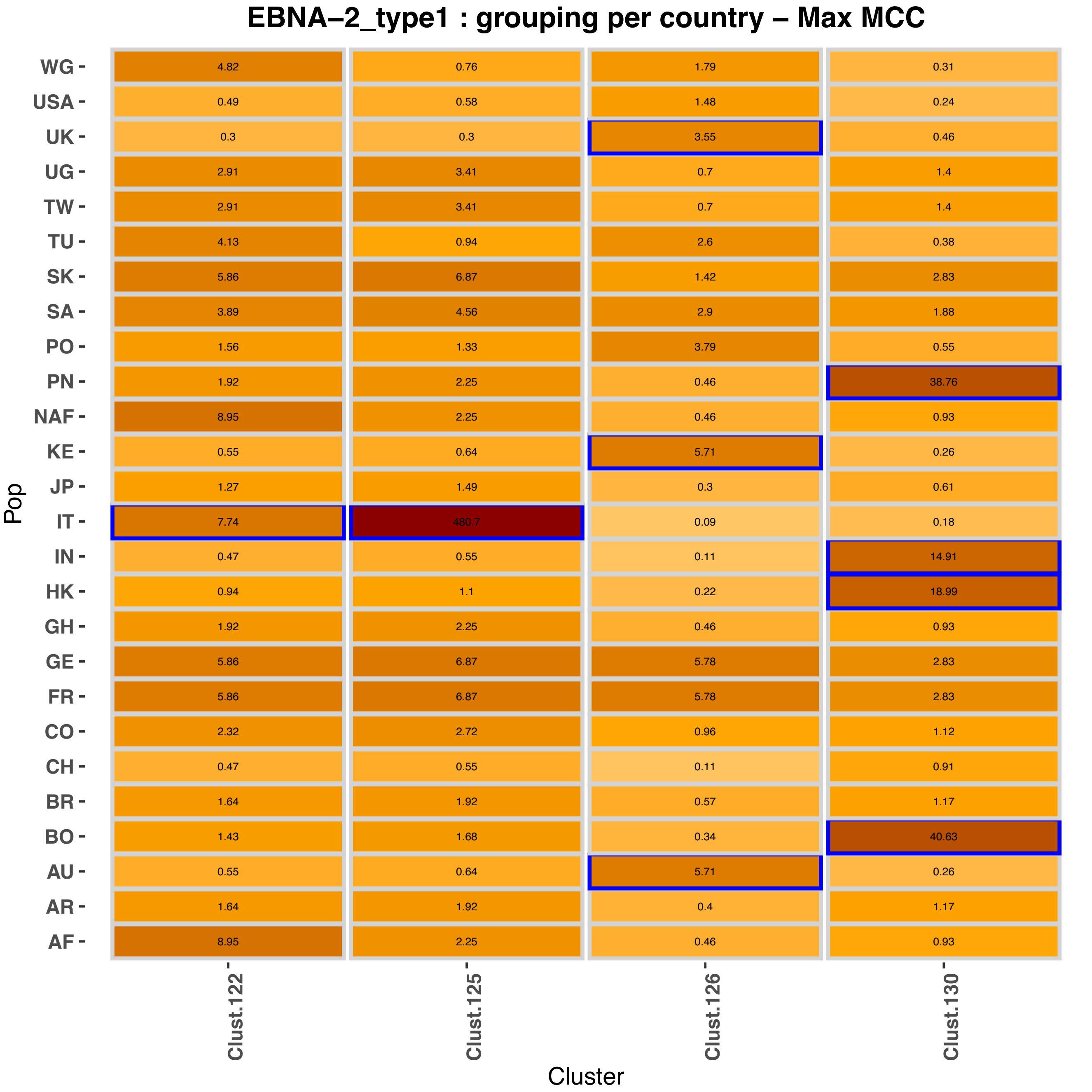
EBNA−2_type1 : grouping per country − Max MCC
Pop
Cluster
Clust.122
Clust.125
Clust.126
Clust.130
WG 4.82 0.76 1.79 0.31
USA 0.49 0.58 1.48 0.24
UK 0.3 0.3 3.55 0.46
UG 2.91 3.41 0.7 1.4
TW 2.91 3.41 0.7 1.4
TU 4.13 0.94 2.6 0.38
SK 5.86 6.87 1.42 2.83
SA 3.89 4.56 2.9 1.88
PO 1.56 1.33 3.79 0.55
PN 1.92 2.25 0.46 38.76
NAF 8.95 2.25 0.46 0.93
KE 0.55 0.64 5.71 0.26
JP 1.27 1.49 0.3 0.61
IT 7.74 480.7 0.09 0.18
IN 0.47 0.55 0.11 14.91
HK 0.94 1.1 0.22 18.99
GH 1.92 2.25 0.46 0.93
GE 5.86 6.87 5.78 2.83
FR 5.86 6.87 5.78 2.83
CO 2.32 2.72 0.96 1.12
CH 0.47 0.55 0.11 0.91
BR 1.64 1.92 0.57 1.17
BO 1.43 1.68 0.34 40.63
AU 0.55 0.64 5.71 0.26
AR 1.64 1.92 0.4 1.17
AF 8.95 2.25 0.46 0.93

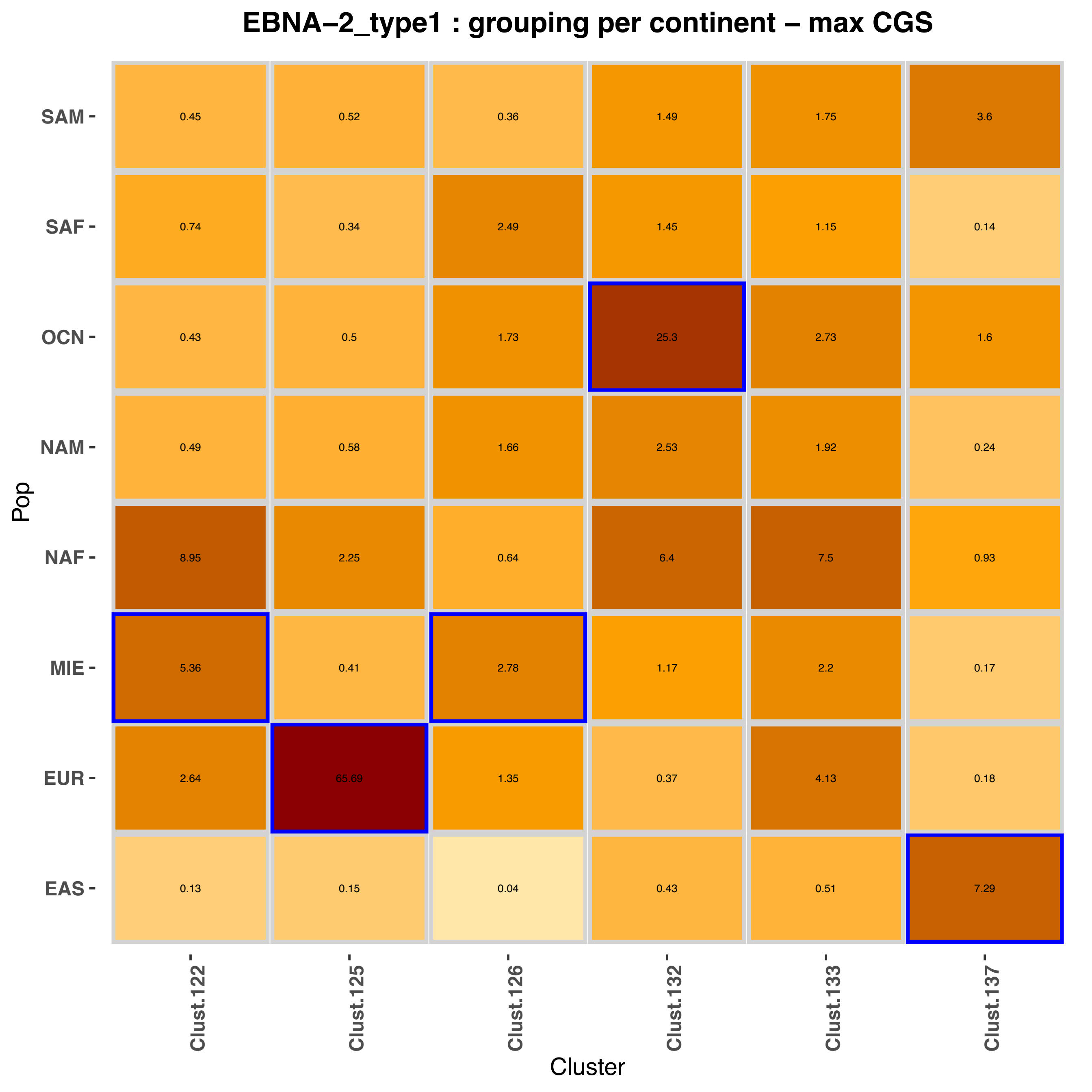
EBNA−2_type1 : grouping per continent − max CGS
Pop
SAM
0.45 0.52 0.36 1.49 1.75 3.6
SAF
0.74 0.34 2.49 1.45 1.15 0.14
OCN
0.43 0.5 1.73 25.3 2.73 1.6
NAM
0.49 0.58 1.66 2.53 1.92 0.24
NAF
8.95 2.25 0.64 6.4 7.5 0.93
MIE
5.36 0.41 2.78 1.17 2.2 0.17
EUR
2.64 65.69 1.35 0.37 4.13 0.18
EAS
0.13 0.15 0.04 0.43 0.51 7.29
Clust.122
Clust.125
Clust.126
Clust.132
Clust.133
Clust.137
Cluster

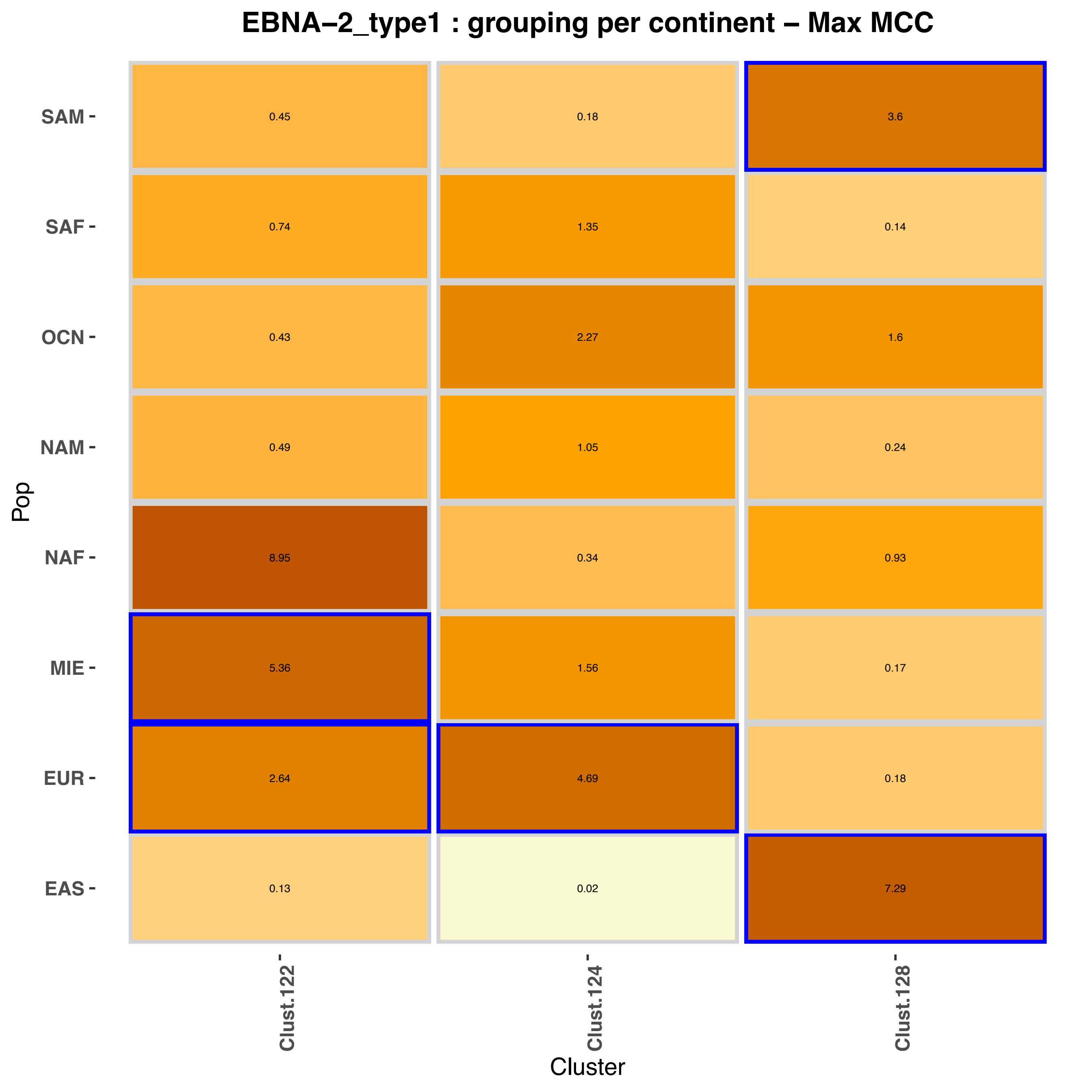
EBNA−2_type1 : grouping per continent − Max MCC
Pop
SAM
SAF
OCN
NAM
NAF
MIE
EUR
EAS
0.45
0.18
3.6
0.74
1.35
0.14
0.43
2.27
1.6
0.49
1.05
0.24
8.95
0.34
0.93
5.36
1.56
0.17
2.64
4.69
0.18
0.13
0.02
7.29
Clust.122
Clust.124
Clust.128
Cluster

EBNA–3A_type1 : grouping per country – max CGS

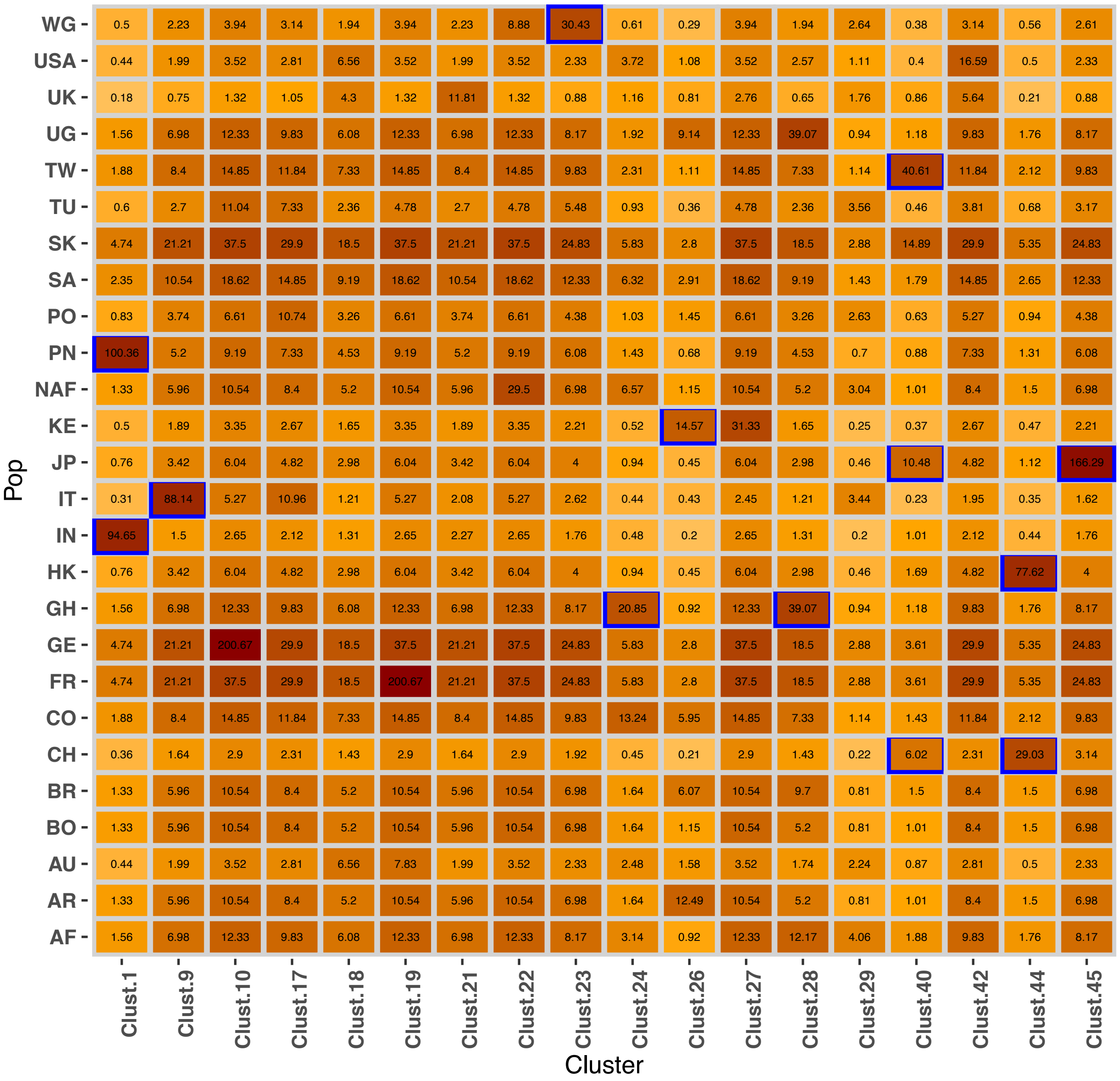

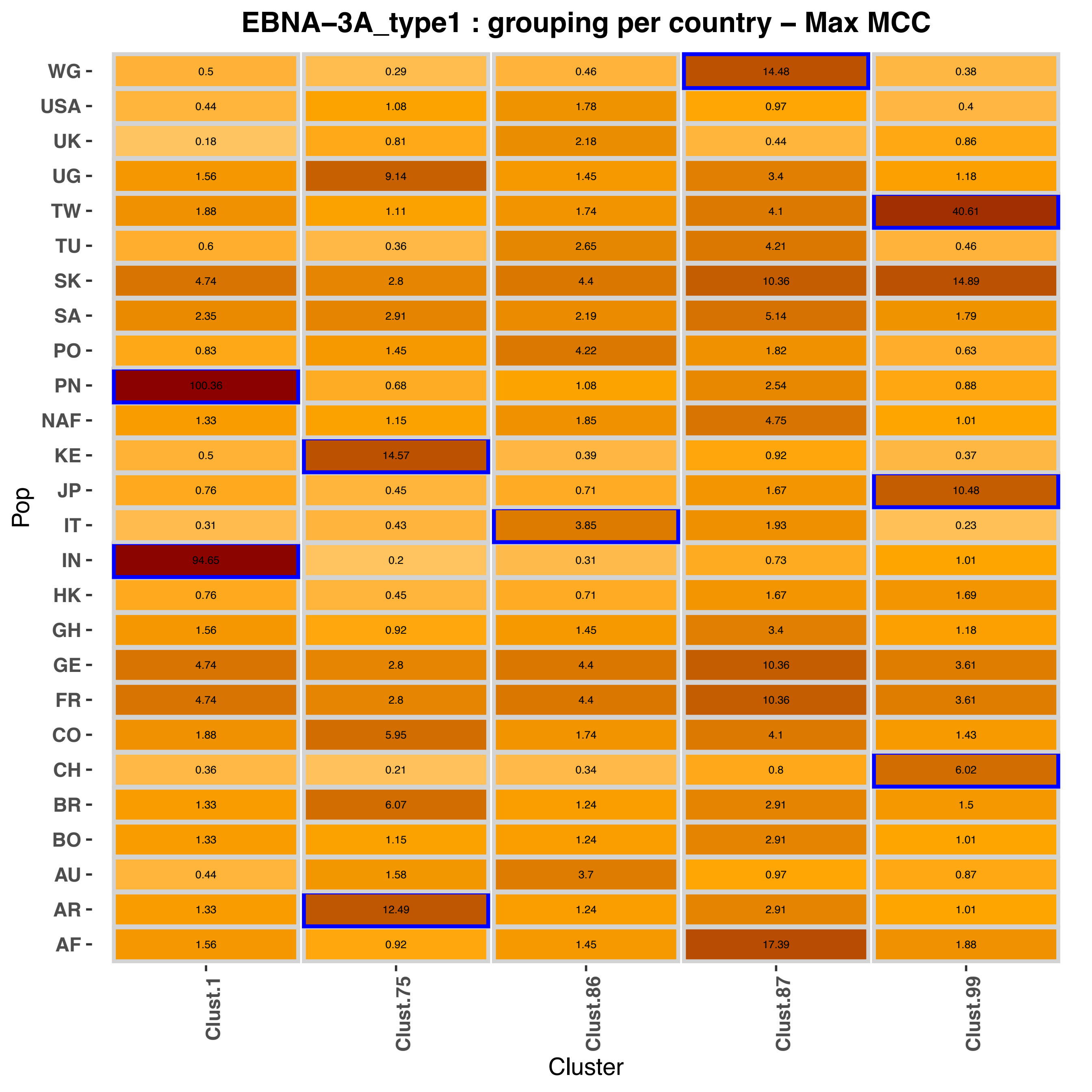

EBNA–3A_type1 : grouping per country – Max MCC
Pop
Cluster
Clust.1
Clust.75
Clust.86
Clust.87
Clust.99
WG
USA
UK
UG
TW
TU
SK
SA
PO
PN
NAF
KE
JP
IT
IN
HK
GH
GE
FR
CO
CH
BR
BO
AU
AR
AF

EBNA–3A_type1 : grouping per continent – max CGS

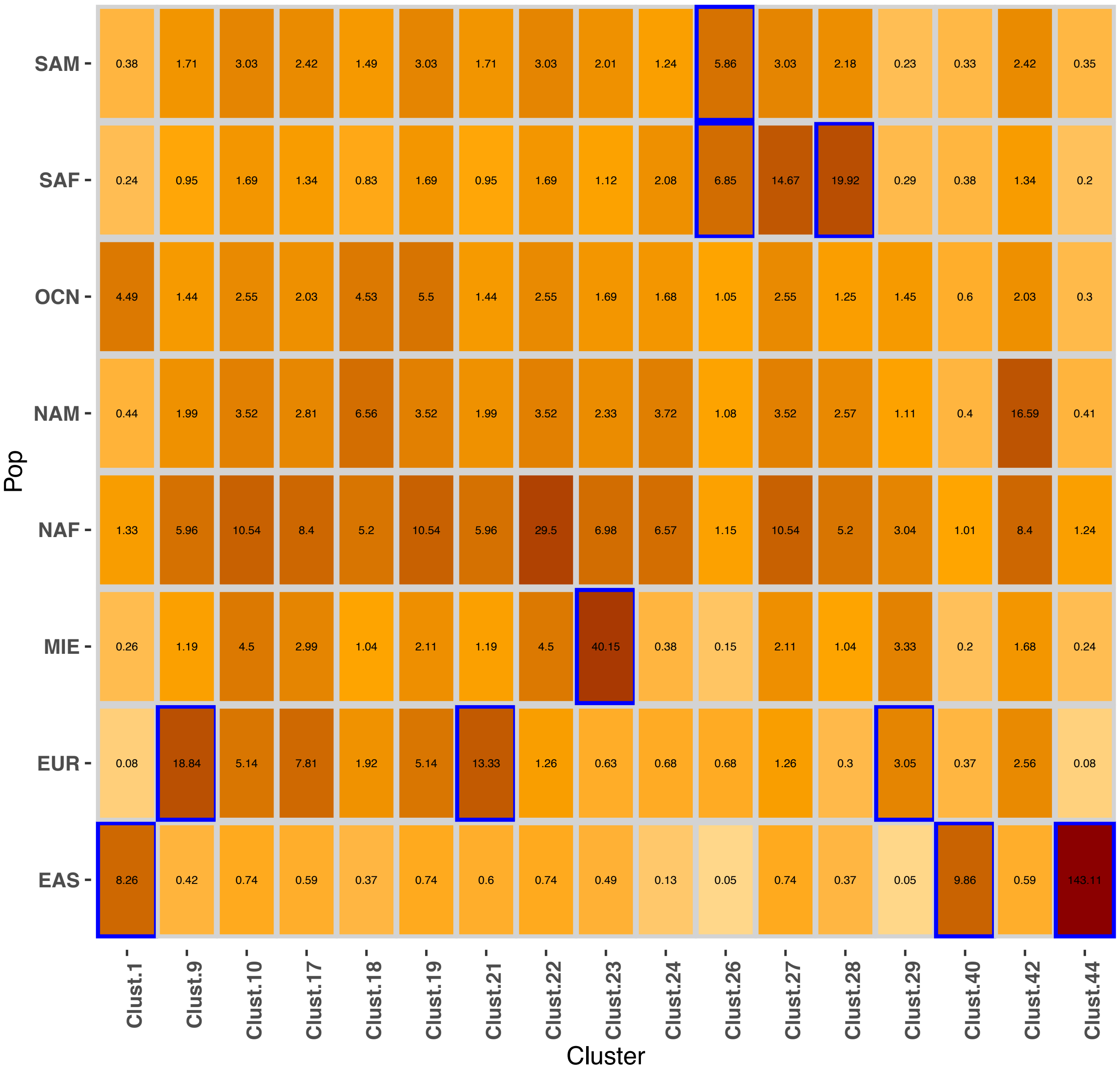

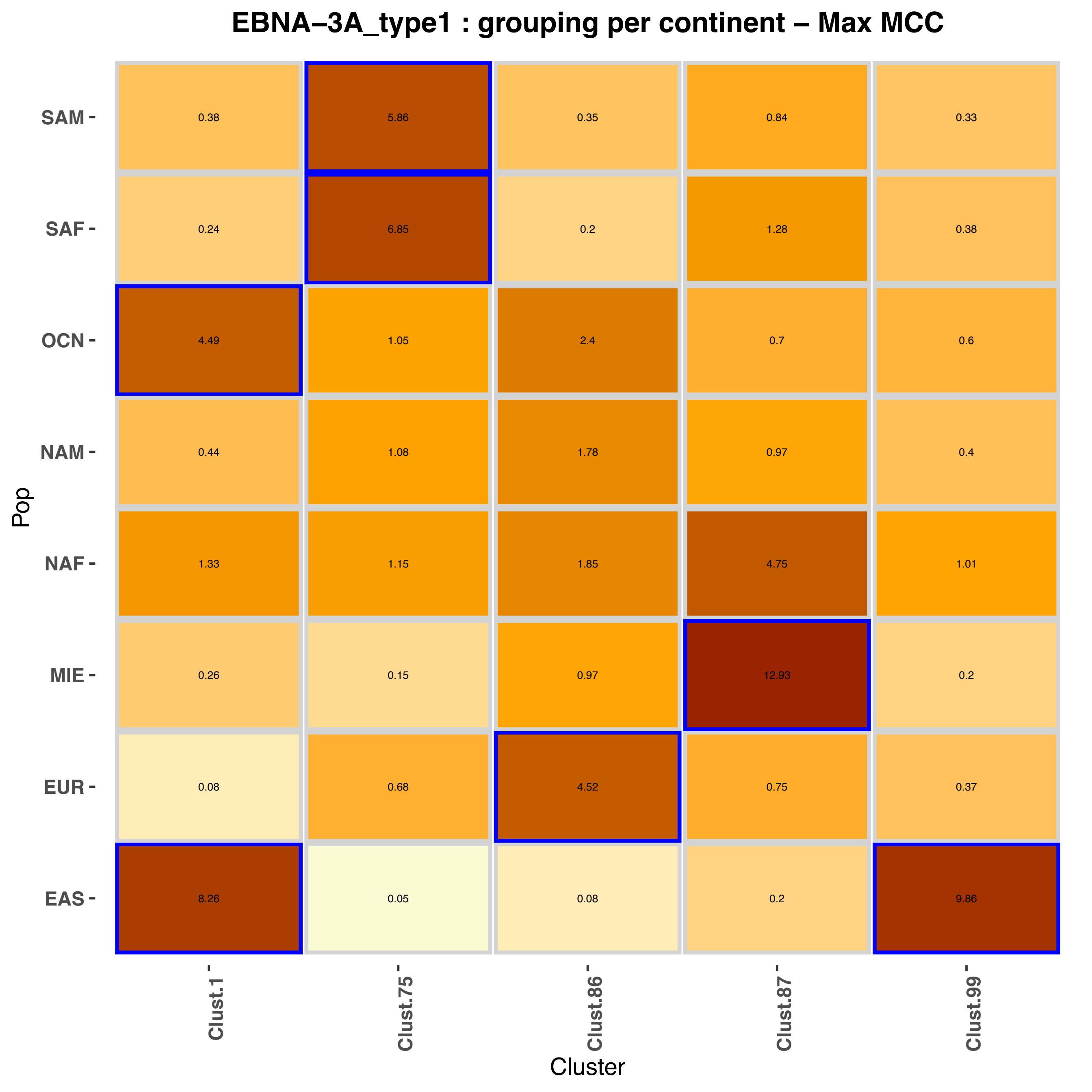
EBNA–3A_type1 : grouping per continent – Max MCC
Pop
SAM
SAF
OCN
NAM
NAF
MIE
EUR
EAS
Cluster
Clust.1
Clust.75
Clust.86
Clust.87
Clust.99
0.38 5.86 0.35 0.84 0.33
0.24 6.85 0.2 1.28 0.38
4.49 1.05 2.4 0.7 0.6
0.44 1.08 1.78 0.97 0.4
1.33 1.15 1.85 4.75 1.01
0.26 0.15 0.97 12.93 0.2
0.08 0.68 4.52 0.75 0.37
8.26 0.05 0.08 0.2 9.86

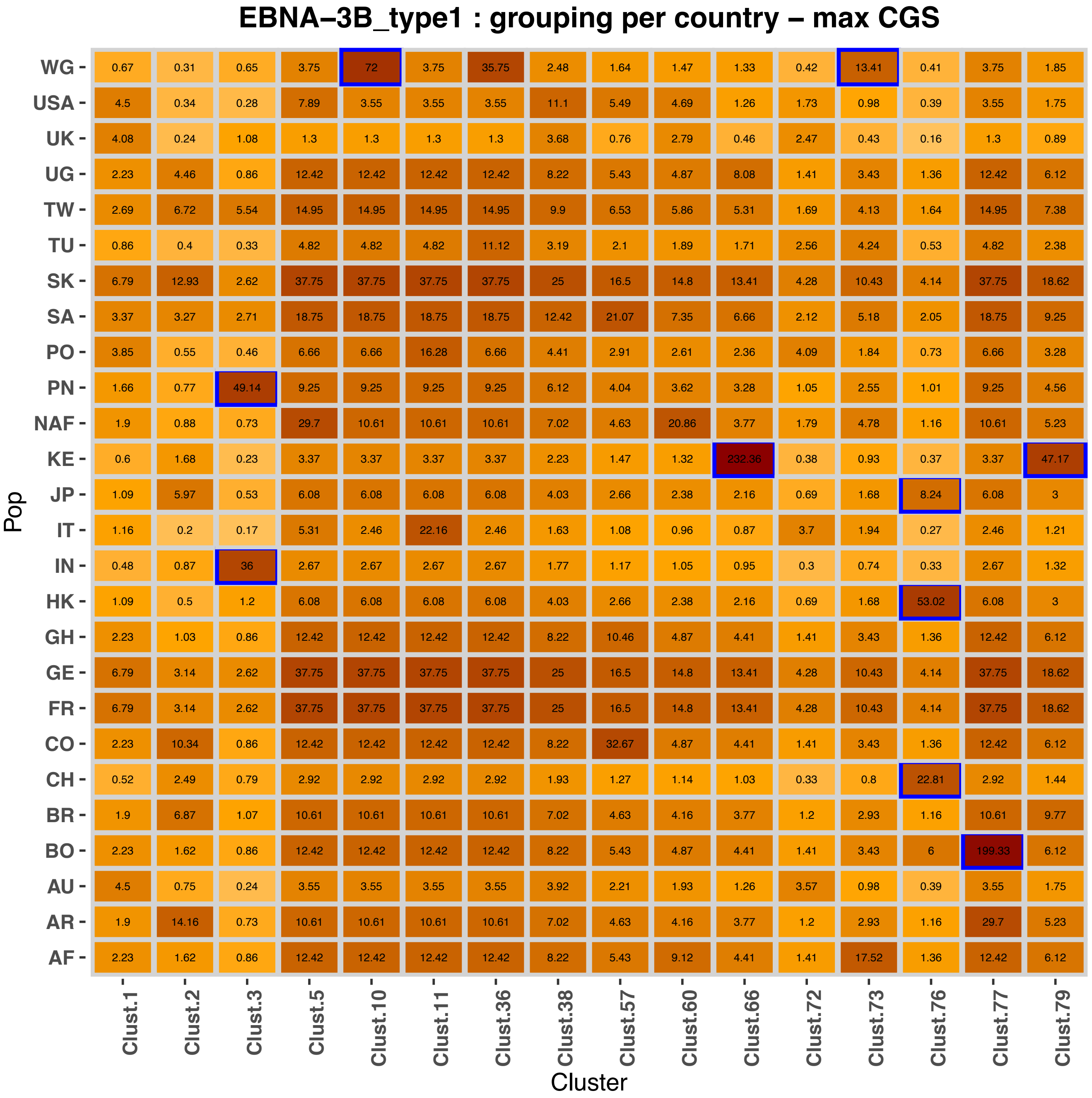
EBNA−3B_type1 : grouping per country − max CGS
Pop
Cluster
Clust.1 Clust.2 Clust.3 Clust.5 Clust.10 Clust.11 Clust.36 Clust.38 Clust.57 Clust.60 Clust.66 Clust.72 Clust.73 Clust.76 Clust.77 Clust.79
WG 0.67 0.31 0.65 3.75 72 3.75 35.75 2.48 1.64 1.47 1.33 0.42 13.41 0.41 3.75 1.85
USA 4.5 0.34 0.28 7.89 3.55 3.55 3.55 11.1 5.49 4.69 1.26 1.73 0.98 0.39 3.55 1.75
UK 4.08 0.24 1.08 1.3 1.3 1.3 1.3 3.68 0.76 2.79 0.46 2.47 0.43 0.16 1.3 0.89
UG 2.23 4.46 0.86 12.42 12.42 12.42 12.42 8.22 5.43 4.87 8.08 1.41 3.43 1.36 12.42 6.12
TW 2.69 6.72 5.54 14.95 14.95 14.95 14.95 9.9 6.53 5.86 5.31 1.69 4.13 1.64 14.95 7.38
TU 0.86 0.4 0.33 4.82 4.82 4.82 11.12 3.19 2.1 1.89 1.71 2.56 4.24 0.53 4.82 2.38
SK 6.79 12.93 2.62 37.75 37.75 37.75 37.75 25 16.5 14.8 13.41 4.28 10.43 4.14 37.75 18.62
SA 3.37 3.27 2.71 18.75 18.75 18.75 18.75 12.42 21.07 7.35 6.66 2.12 5.18 2.05 18.75 9.25
PO 3.85 0.55 0.46 6.66 6.66 16.28 6.66 4.41 2.91 2.61 2.36 4.09 1.84 0.73 6.66 3.28
PN 1.66 0.77 49.14 9.25 9.25 9.25 9.25 6.12 4.04 3.62 3.28 1.05 2.55 1.01 9.25 4.56
NAF 1.9 0.88 0.73 29.7 10.61 10.61 10.61 7.02 4.63 20.86 3.77 1.79 4.78 1.16 10.61 5.23
KE 0.6 1.68 0.23 3.37 3.37 3.37 3.37 2.23 1.47 1.32 232.36 0.38 0.93 0.37 3.37 47.17
JP 1.09 5.97 0.53 6.08 6.08 6.08 6.08 4.03 2.66 2.38 2.16 0.69 1.68 8.24 6.08 3
IT 1.16 0.2 0.17 5.31 2.46 22.16 2.46 1.63 1.08 0.96 0.87 3.7 1.94 0.27 2.46 1.21
IN 0.48 0.87 36 2.67 2.67 2.67 2.67 1.77 1.17 1.05 0.95 0.3 0.74 0.33 2.67 1.32
HK 1.09 0.5 1.2 6.08 6.08 6.08 6.08 4.03 2.66 2.38 2.16 0.69 1.68 53.02 6.08 3
GH 2.23 1.03 0.86 12.42 12.42 12.42 12.42 8.22 10.46 4.87 4.41 1.41 3.43 1.36 12.42 6.12
GE 6.79 3.14 2.62 37.75 37.75 37.75 37.75 25 16.5 14.8 13.41 4.28 10.43 4.14 37.75 18.62
FR 6.79 3.14 2.62 37.75 37.75 37.75 37.75 25 16.5 14.8 13.41 4.28 10.43 4.14 37.75 18.62
CO 2.23 10.34 0.86 12.42 12.42 12.42 12.42 8.22 32.67 4.87 4.41 1.41 3.43 1.36 12.42 6.12
CH 0.52 2.49 0.79 2.92 2.92 2.92 2.92 1.93 1.27 1.14 1.03 0.33 0.8 22.81 2.92 1.44
BR 1.9 6.87 1.07 10.61 10.61 10.61 10.61 7.02 4.63 4.16 3.77 1.2 2.93 1.16 10.61 9.77
BO 2.23 1.62 0.86 12.42 12.42 12.42 12.42 8.22 5.43 4.87 4.41 1.41 3.43 6 199.33 6.12
AU 4.5 0.75 0.24 3.55 3.55 3.55 3.55 3.92 2.21 1.93 1.26 3.57 0.98 0.39 3.55 1.75
AR 1.9 14.16 0.73 10.61 10.61 10.61 10.61 7.02 4.63 4.16 3.77 1.2 2.93 1.16 29.7 5.23
AF 2.23 1.62 0.86 12.42 12.42 12.42 12.42 8.22 5.43 9.12 4.41 1.41 17.52 1.36 12.42 6.12

EBNA–3B_type1 : grouping per country – Max MCC

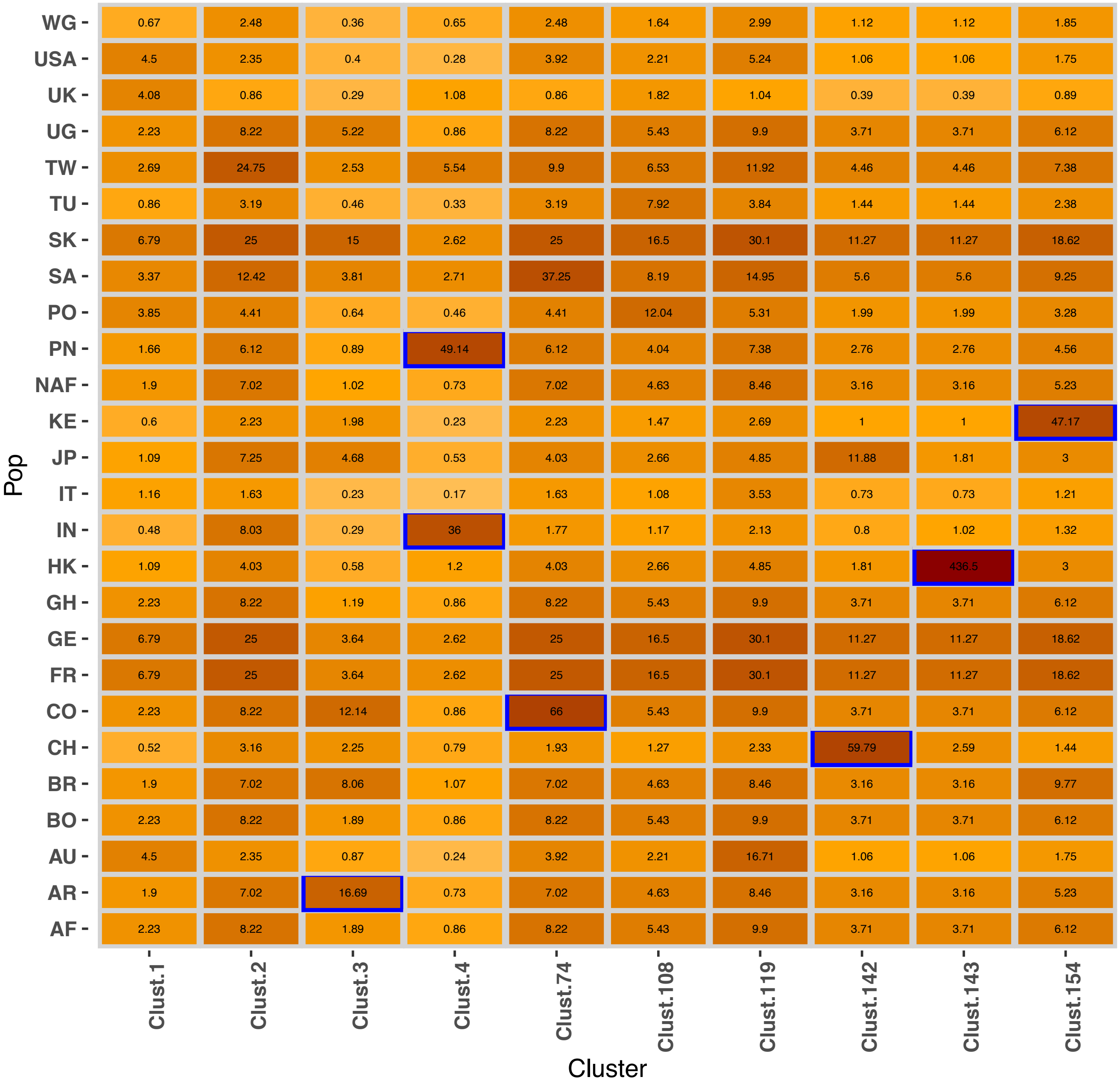

EBNA–3B_type1 : grouping per continent – max CGS

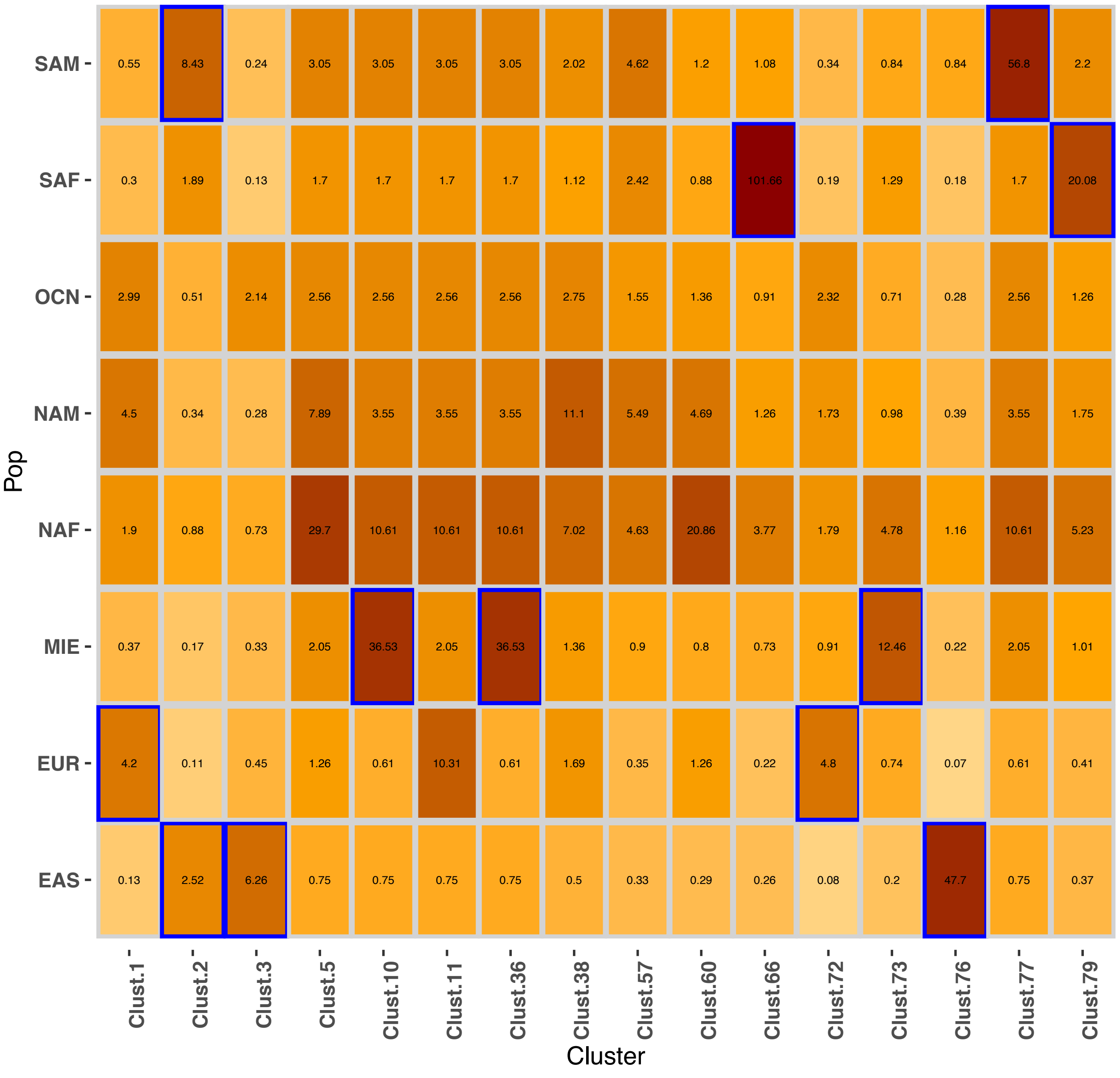

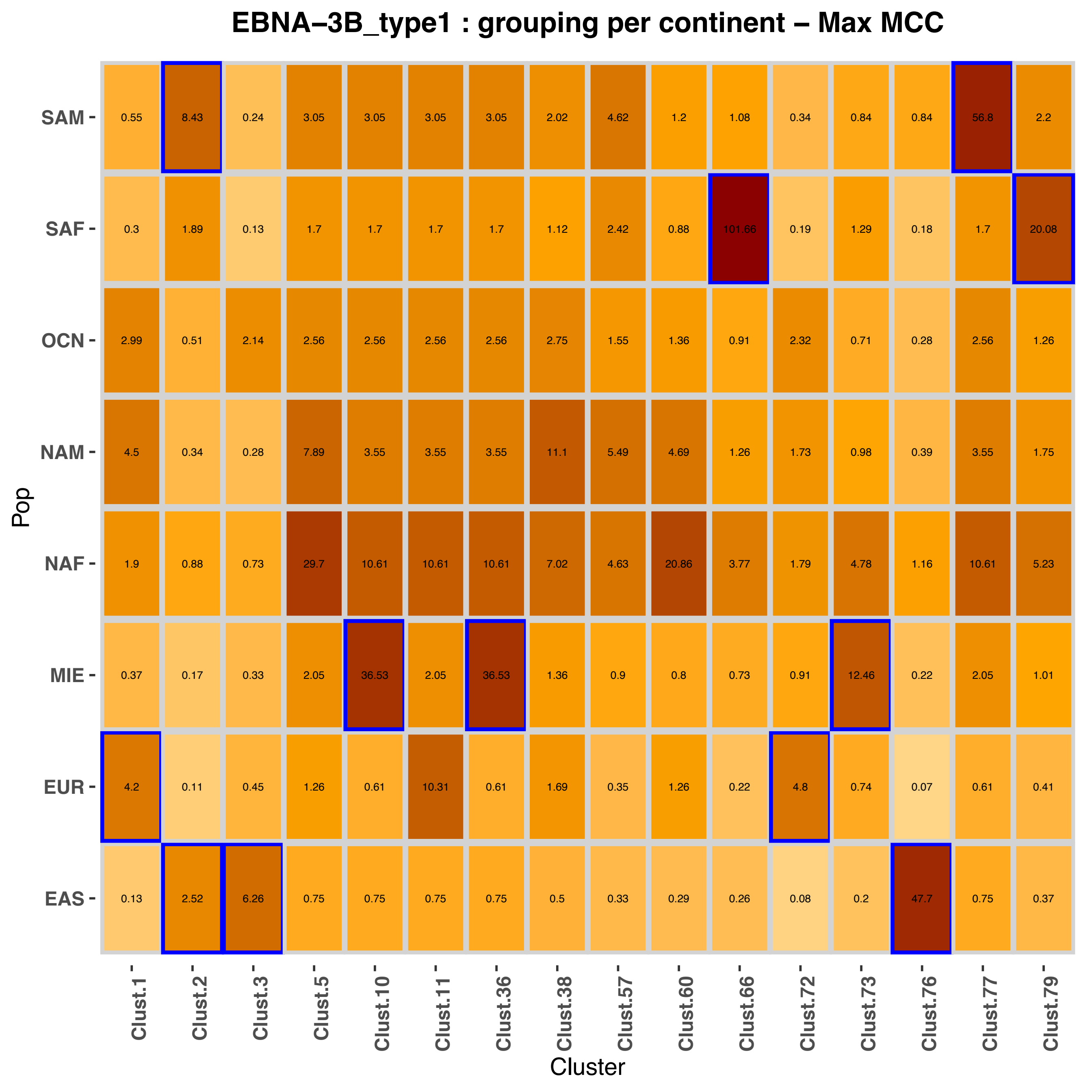
EBNA–3B_type1 : grouping per continent – Max MCC
Pop
Cluster
SAM
SAF
OCN
NAM
NAF
MIE
EUR
EAS
Clust.1
Clust.2
Clust.3
Clust.5
Clust.10
Clust.11
Clust.36
Clust.38
Clust.57
Clust.60
Clust.66
Clust.72
Clust.73
Clust.76
Clust.77
Clust.79
0.55 8.43 0.24 3.05 3.05 3.05 3.05 2.02 4.62 1.2 1.08 0.34 0.84 0.84 56.8 2.2
0.3 1.89 0.13 1.7 1.7 1.7 1.7 1.12 2.42 0.88 101.66 0.19 1.29 0.18 1.7 20.08
2.99 0.51 2.14 2.56 2.56 2.56 2.56 2.75 1.55 1.36 0.91 2.32 0.71 0.28 2.56 1.26
4.5 0.34 0.28 7.89 3.55 3.55 3.55 11.1 5.49 4.69 1.26 1.73 0.98 0.39 3.55 1.75
1.9 0.88 0.73 29.7 10.61 10.61 10.61 7.02 4.63 20.86 3.77 1.79 4.78 1.16 10.61 5.23
0.37 0.17 0.33 2.05 36.53 2.05 36.53 1.36 0.9 0.8 0.73 0.91 12.46 0.22 2.05 1.01
4.2 0.11 0.45 1.26 0.61 10.31 0.61 1.69 0.35 1.26 0.22 4.8 0.74 0.07 0.61 0.41
0.13 2.52 6.26 0.75 0.75 0.75 0.75 0.5 0.33 0.29 0.26 0.08 0.2 47.7 0.75 0.37

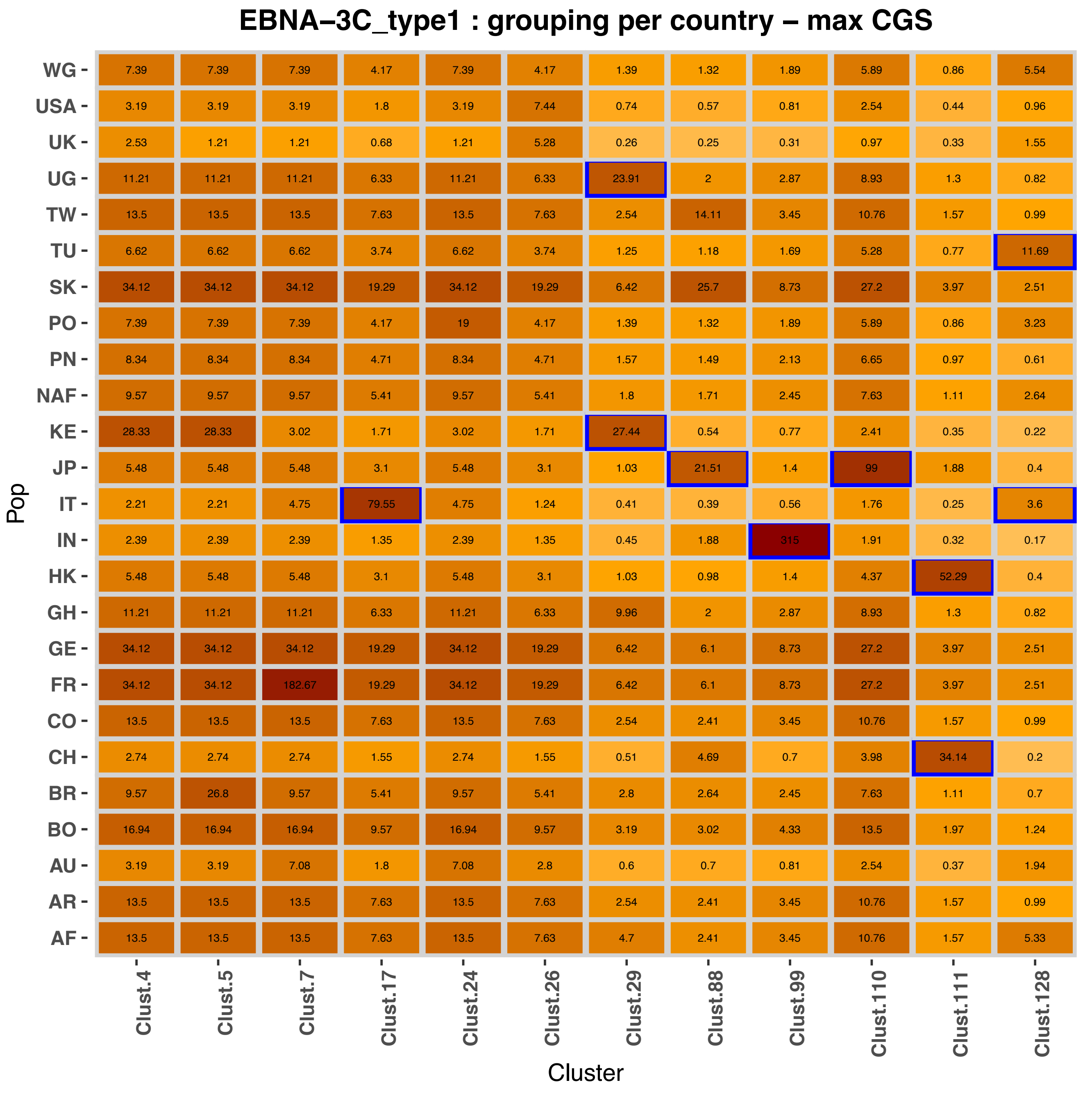
EBNA−3C_type1 : grouping per country − max CGS
Pop
Cluster
Clust.4 Clust.5 Clust.7 Clust.17 Clust.24 Clust.26 Clust.29 Clust.88 Clust.99 Clust.110 Clust.111 Clust.128
WG 7.39 7.39 7.39 4.17 7.39 4.17 1.39 1.32 1.89 5.89 0.86 5.54
USA 3.19 3.19 3.19 1.8 3.19 7.44 0.74 0.57 0.81 2.54 0.44 0.96
UK 2.53 1.21 1.21 0.68 1.21 5.28 0.26 0.25 0.31 0.97 0.33 1.55
UG 11.21 11.21 11.21 6.33 11.21 6.33 23.91 2 2.87 8.93 1.3 0.82
TW 13.5 13.5 13.5 7.63 13.5 7.63 2.54 14.11 3.45 10.76 1.57 0.99
TU 6.62 6.62 6.62 3.74 6.62 3.74 1.25 1.18 1.69 5.28 0.77 11.69
SK 34.12 34.12 34.12 19.29 34.12 19.29 6.42 25.7 8.73 27.2 3.97 2.51
PO 7.39 7.39 7.39 4.17 19 4.17 1.39 1.32 1.89 5.89 0.86 3.23
PN 8.34 8.34 8.34 4.71 8.34 4.71 1.57 1.49 2.13 6.65 0.97 0.61
NAF 9.57 9.57 9.57 5.41 9.57 5.41 1.8 1.71 2.45 7.63 1.11 2.64
KE 28.33 28.33 3.02 1.71 3.02 1.71 27.44 0.54 0.77 2.41 0.35 0.22
JP 5.48 5.48 5.48 3.1 5.48 3.1 1.03 21.51 1.4 99 1.88 0.4
IT 2.21 2.21 4.75 79.55 4.75 1.24 0.41 0.39 0.56 1.76 0.25 3.6
IN 2.39 2.39 2.39 1.35 2.39 1.35 0.45 1.88 315 1.91 0.32 0.17
HK 5.48 5.48 5.48 3.1 5.48 3.1 1.03 0.98 1.4 4.37 52.29 0.4
GH 11.21 11.21 11.21 6.33 11.21 6.33 9.96 2 2.87 8.93 1.3 0.82
GE 34.12 34.12 34.12 19.29 34.12 19.29 6.42 6.1 8.73 27.2 3.97 2.51
FR 34.12 34.12 182.67 19.29 34.12 19.29 6.42 6.1 8.73 27.2 3.97 2.51
CO 13.5 13.5 13.5 7.63 13.5 7.63 2.54 2.41 3.45 10.76 1.57 0.99
CH 2.74 2.74 2.74 1.55 2.74 1.55 0.51 4.69 0.7 3.98 34.14 0.2
BR 9.57 26.8 9.57 5.41 9.57 5.41 2.8 2.64 2.45 7.63 1.11 0.7
BO 16.94 16.94 16.94 9.57 16.94 9.57 3.19 3.02 4.33 13.5 1.97 1.24
AU 3.19 3.19 7.08 1.8 7.08 2.8 0.6 0.7 0.81 2.54 0.37 1.94
AR 13.5 13.5 13.5 7.63 13.5 7.63 2.54 2.41 3.45 10.76 1.57 0.99
AF 13.5 13.5 13.5 7.63 13.5 7.63 4.7 2.41 3.45 10.76 1.57 5.33

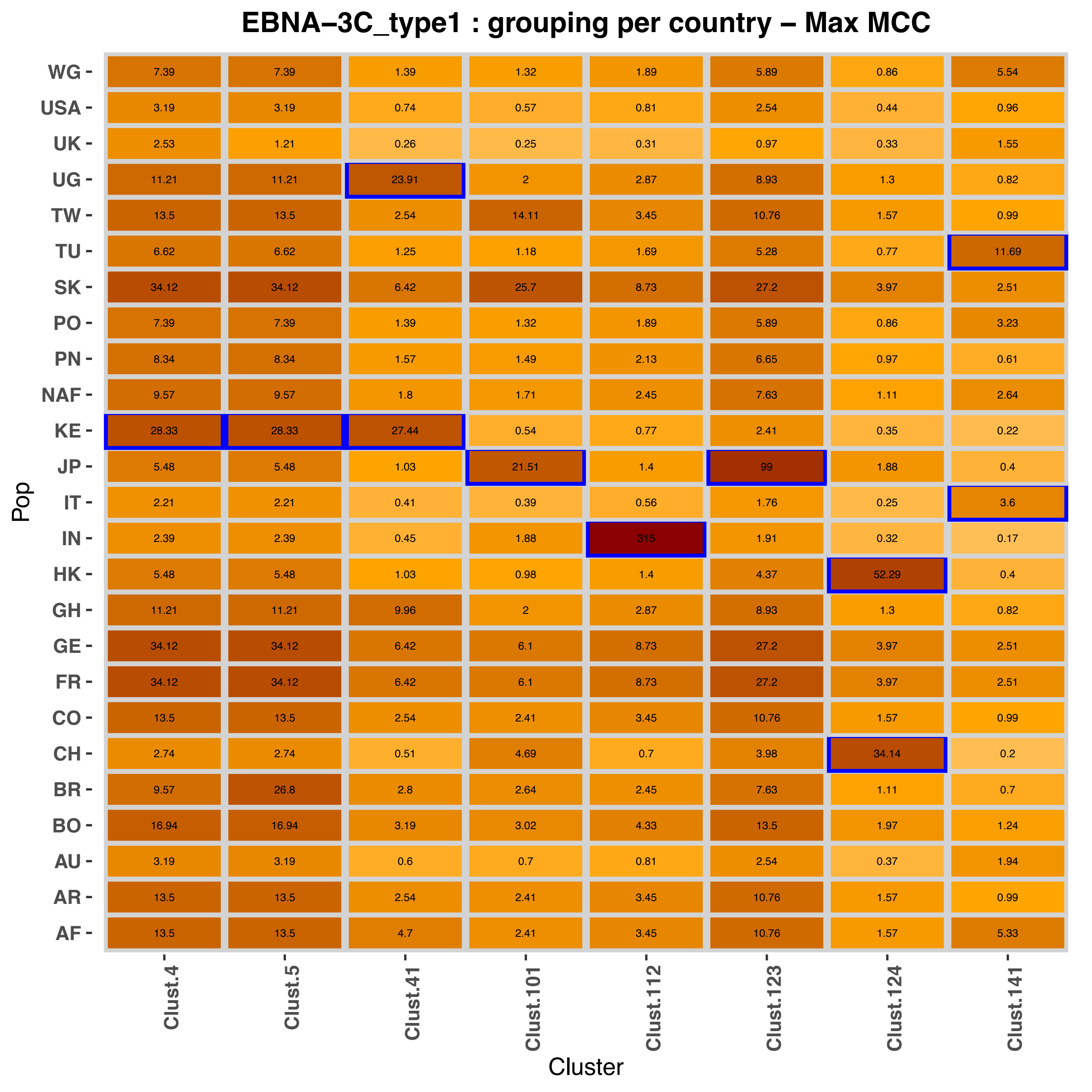
EBNA–3C_type1 : grouping per country – Max MCC
WG 7.39 7.39 1.39 1.32 1.89 5.89 0.86 5.54
USA 3.19 3.19 0.74 0.57 0.81 2.54 0.44 0.96
UK 2.53 1.21 0.26 0.25 0.31 0.97 0.33 1.55
UG 11.21 11.21 23.91 2 2.87 8.93 1.3 0.82
TW 13.5 13.5 2.54 14.11 3.45 10.76 1.57 0.99
TU 6.62 6.62 1.25 1.18 1.69 5.28 0.77 11.69
SK 34.12 34.12 6.42 25.7 8.73 27.2 3.97 2.51
PO 7.39 7.39 1.39 1.32 1.89 5.89 0.86 3.23
PN 8.34 8.34 1.57 1.49 2.13 6.65 0.97 0.61
NAF 9.57 9.57 1.8 1.71 2.45 7.63 1.11 2.64
KE 28.33 28.33 27.44 0.54 0.77 2.41 0.35 0.22
JP 5.48 5.48 1.03 21.51 1.4 99 1.88 0.4
IT 2.21 2.21 0.41 0.39 0.56 1.76 0.25 3.6
IN 2.39 2.39 0.45 1.88 315 1.91 0.32 0.17
HK 5.48 5.48 1.03 0.98 1.4 4.37 52.29 0.4
GH 11.21 11.21 9.96 2 2.87 8.93 1.3 0.82
GE 34.12 34.12 6.42 6.1 8.73 27.2 3.97 2.51
FR 34.12 34.12 6.42 6.1 8.73 27.2 3.97 2.51
CO 13.5 13.5 2.54 2.41 3.45 10.76 1.57 0.99
CH 2.74 2.74 0.51 4.69 0.7 3.98 34.14 0.2
BR 9.57 26.8 2.8 2.64 2.45 7.63 1.11 0.7
BO 16.94 16.94 3.19 3.02 4.33 13.5 1.97 1.24
AU 3.19 3.19 0.6 0.7 0.81 2.54 0.37 1.94
AR 13.5 13.5 2.54 2.41 3.45 10.76 1.57 0.99
AF 13.5 13.5 4.7 2.41 3.45 10.76 1.57 5.33
Pop
Clust.4 Clust.5 Clust.41 Clust.101 Clust.112 Clust.123 Clust.124 Clust.141
Cluster

EBNA–3C_type1 : grouping per continent – max CGS

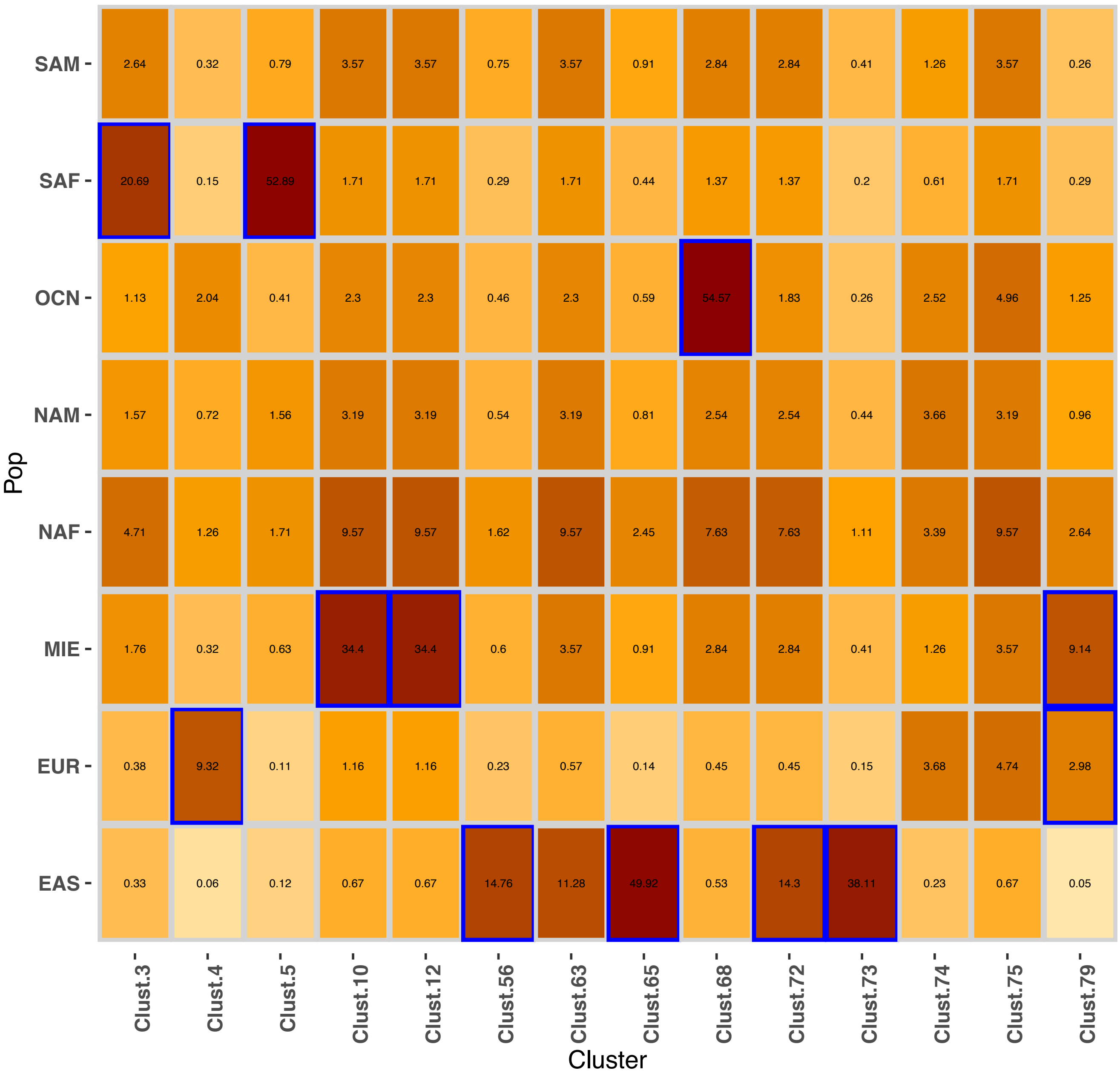

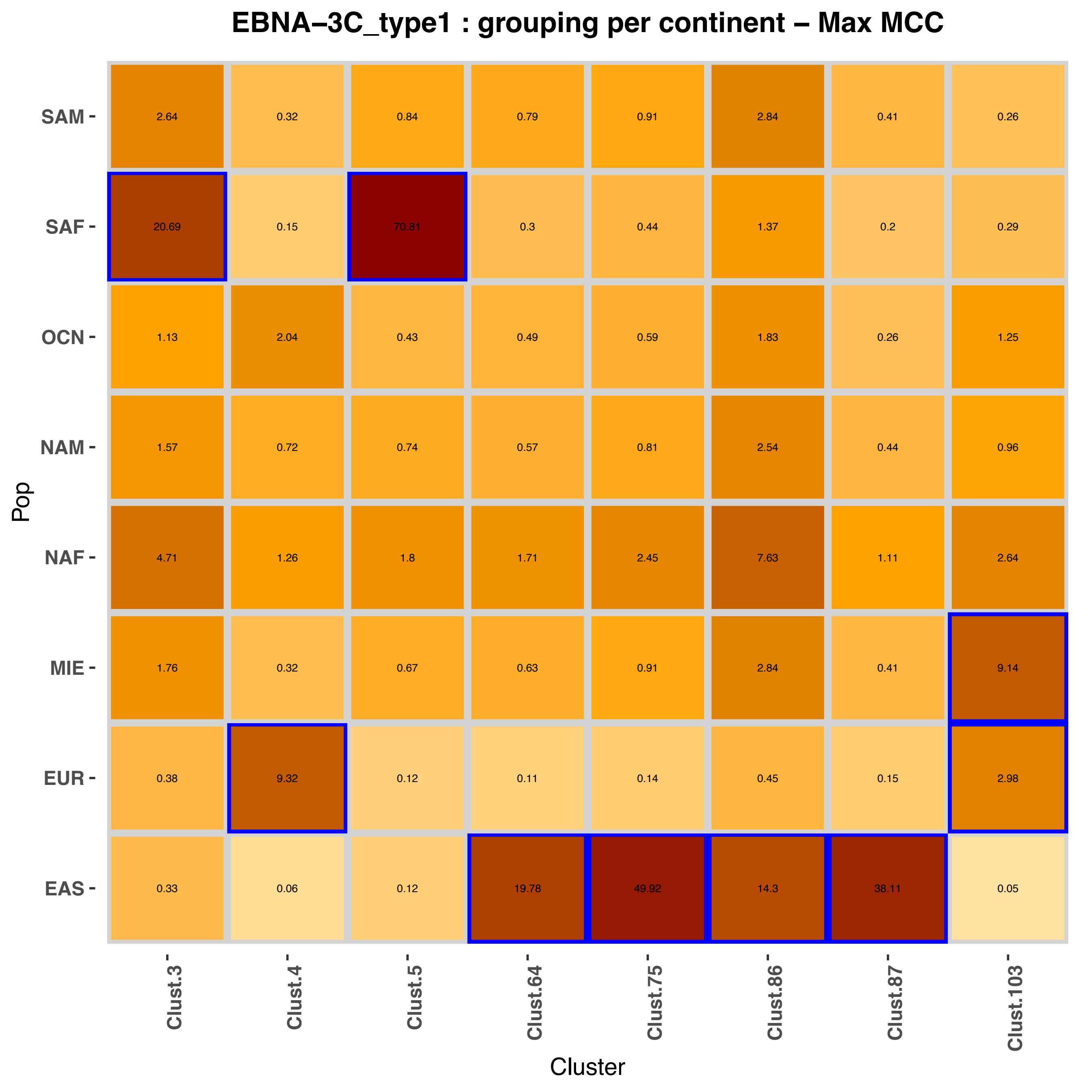
EBNA–3C_type1 : grouping per continent – Max MCC
Pop
SAM
SAF
OCN
NAM
NAF
MIE
EUR
EAS
Cluster
Clust.3
Clust.4
Clust.5
Clust.64
Clust.75
Clust.86
Clust.87
Clust.103
SAM: 2.64 0.32 0.84 0.79 0.91 2.84 0.41 0.26
SAF: 20.69 0.15 70.81 0.3 0.44 1.37 0.2 0.29
OCN: 1.13 2.04 0.43 0.49 0.59 1.83 0.26 1.25
NAM: 1.57 0.72 0.74 0.57 0.81 2.54 0.44 0.96
NAF: 4.71 1.26 1.8 1.71 2.45 7.63 1.11 2.64
MIE: 1.76 0.32 0.67 0.63 0.91 2.84 0.41 9.14
EUR: 0.38 9.32 0.12 0.11 0.14 0.45 0.15 2.98
EAS: 0.33 0.06 0.12 19.78 49.92 14.3 38.11 0.05

LMP–2A : grouping per country – max CGS

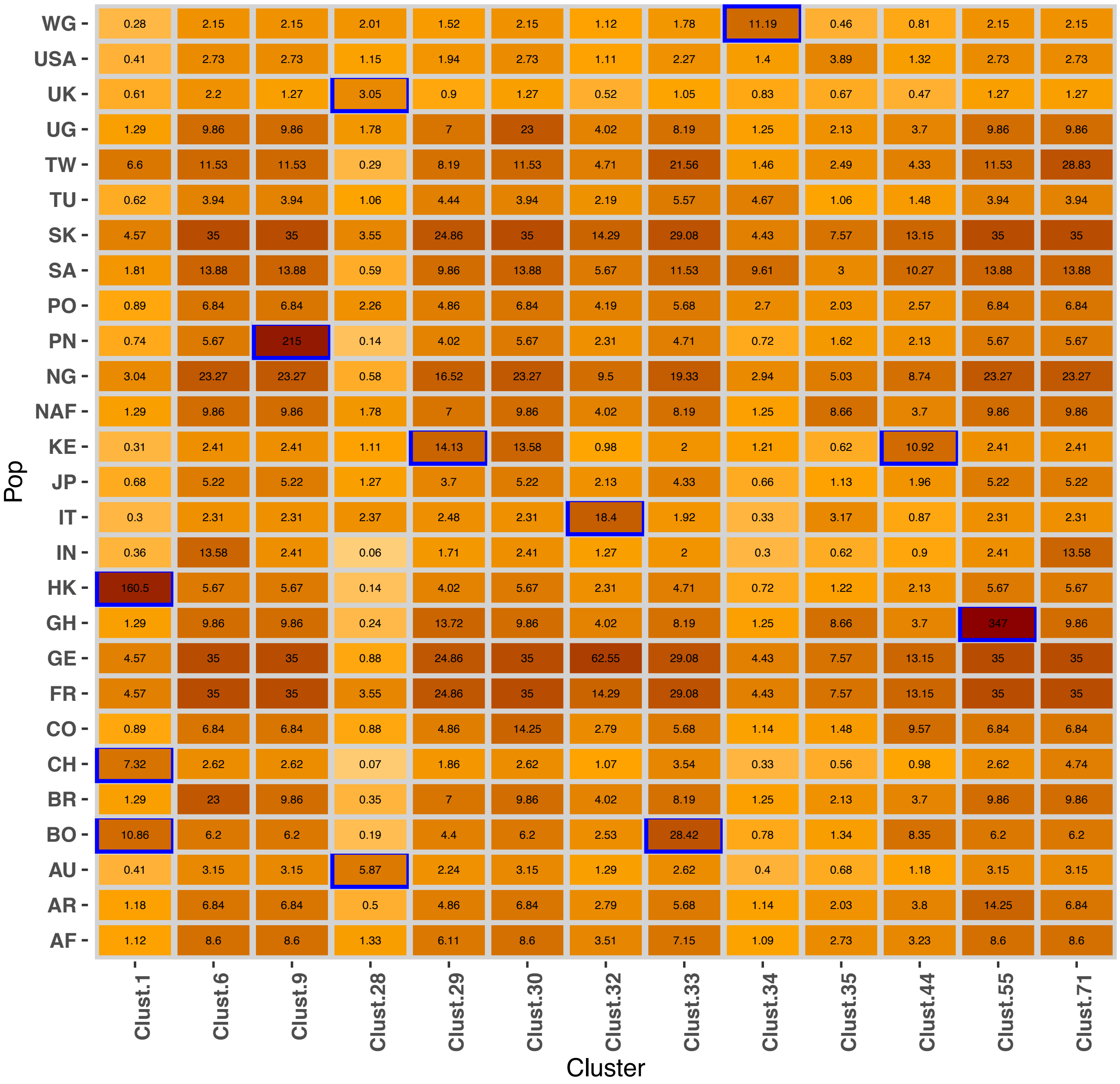

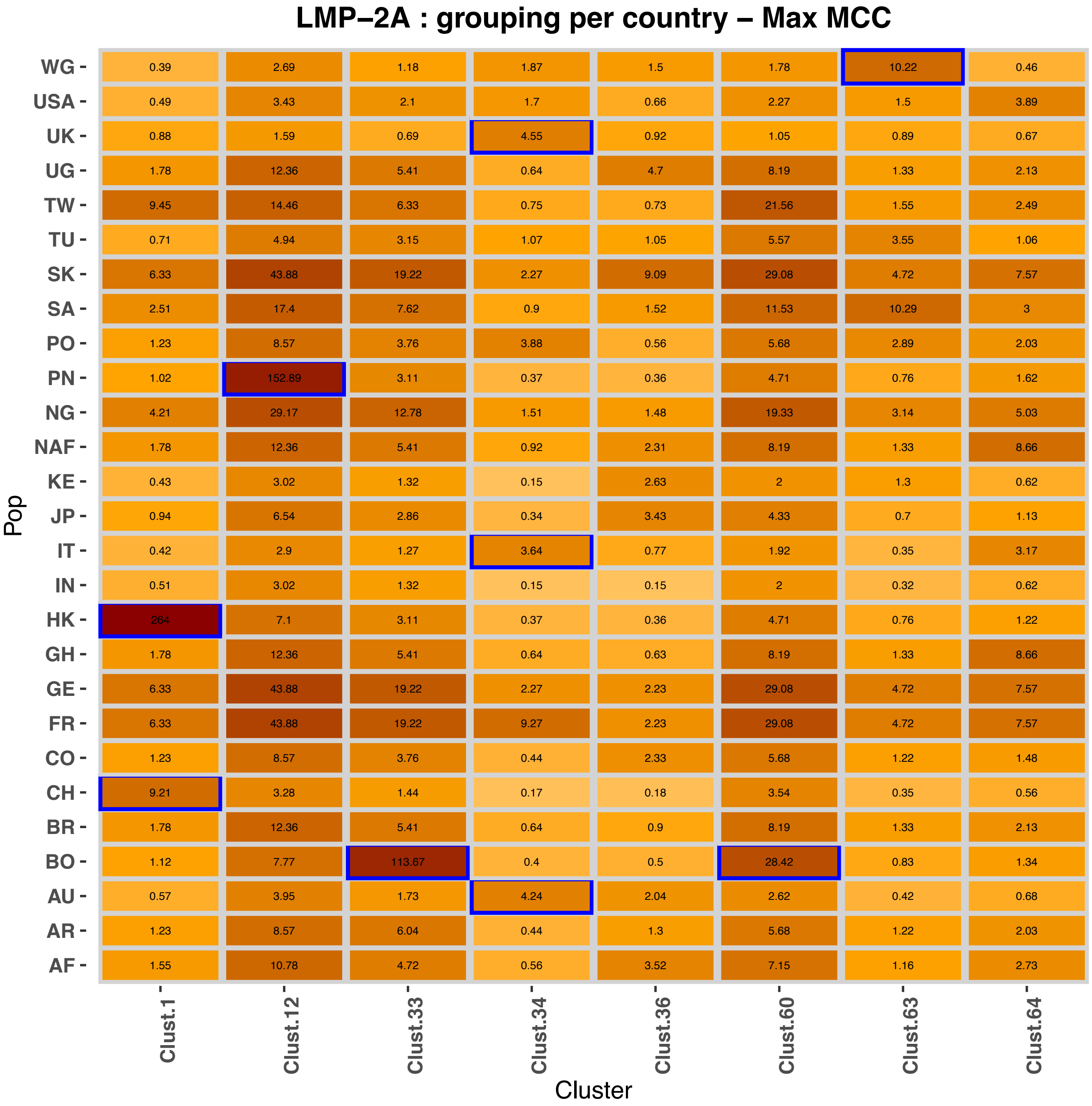
LMP−2A : grouping per country − Max MCC
Pop
Cluster
Clust.1 Clust.12 Clust.33 Clust.34 Clust.36 Clust.60 Clust.63 Clust.64
WG 0.39 2.69 1.18 1.87 1.5 1.78 10.22 0.46
USA 0.49 3.43 2.1 1.7 0.66 2.27 1.5 3.89
UK 0.88 1.59 0.69 4.55 0.92 1.05 0.89 0.67
UG 1.78 12.36 5.41 0.64 4.7 8.19 1.33 2.13
TW 9.45 14.46 6.33 0.75 0.73 21.56 1.55 2.49
TU 0.71 4.94 3.15 1.07 1.05 5.57 3.55 1.06
SK 6.33 43.88 19.22 2.27 9.09 29.08 4.72 7.57
SA 2.51 17.4 7.62 0.9 1.52 11.53 10.29 3
PO 1.23 8.57 3.76 3.88 0.56 5.68 2.89 2.03
PN 1.02 152.89 3.11 0.37 0.36 4.71 0.76 1.62
NG 4.21 29.17 12.78 1.51 1.48 19.33 3.14 5.03
NAF 1.78 12.36 5.41 0.92 2.31 8.19 1.33 8.66
KE 0.43 3.02 1.32 0.15 2.63 2 1.3 0.62
JP 0.94 6.54 2.86 0.34 3.43 4.33 0.7 1.13
IT 0.42 2.9 1.27 3.64 0.77 1.92 0.35 3.17
IN 0.51 3.02 1.32 0.15 0.15 2 0.32 0.62
HK 264 7.1 3.11 0.37 0.36 4.71 0.76 1.22
GH 1.78 12.36 5.41 0.64 0.63 8.19 1.33 8.66
GE 6.33 43.88 19.22 2.27 2.23 29.08 4.72 7.57
FR 6.33 43.88 19.22 9.27 2.23 29.08 4.72 7.57
CO 1.23 8.57 3.76 0.44 2.33 5.68 1.22 1.48
CH 9.21 3.28 1.44 0.17 0.18 3.54 0.35 0.56
BR 1.78 12.36 5.41 0.64 0.9 8.19 1.33 2.13
BO 1.12 7.77 113.67 0.4 0.5 28.42 0.83 1.34
AU 0.57 3.95 1.73 4.24 2.04 2.62 0.42 0.68
AR 1.23 8.57 6.04 0.44 1.3 5.68 1.22 2.03
AF 1.55 10.78 4.72 0.56 3.52 7.15 1.16 2.73

LMP−2A : grouping per continent – max CGS

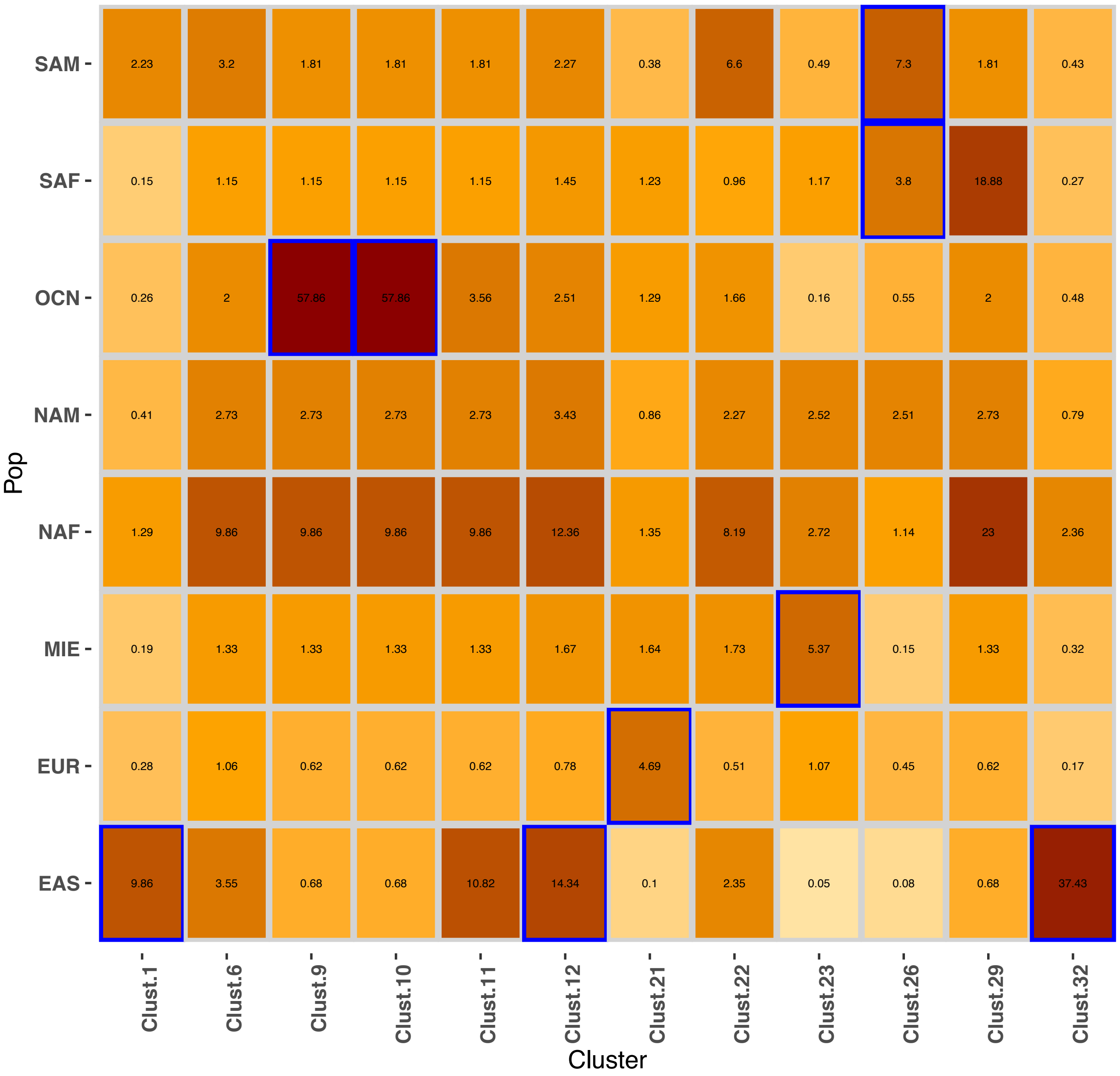

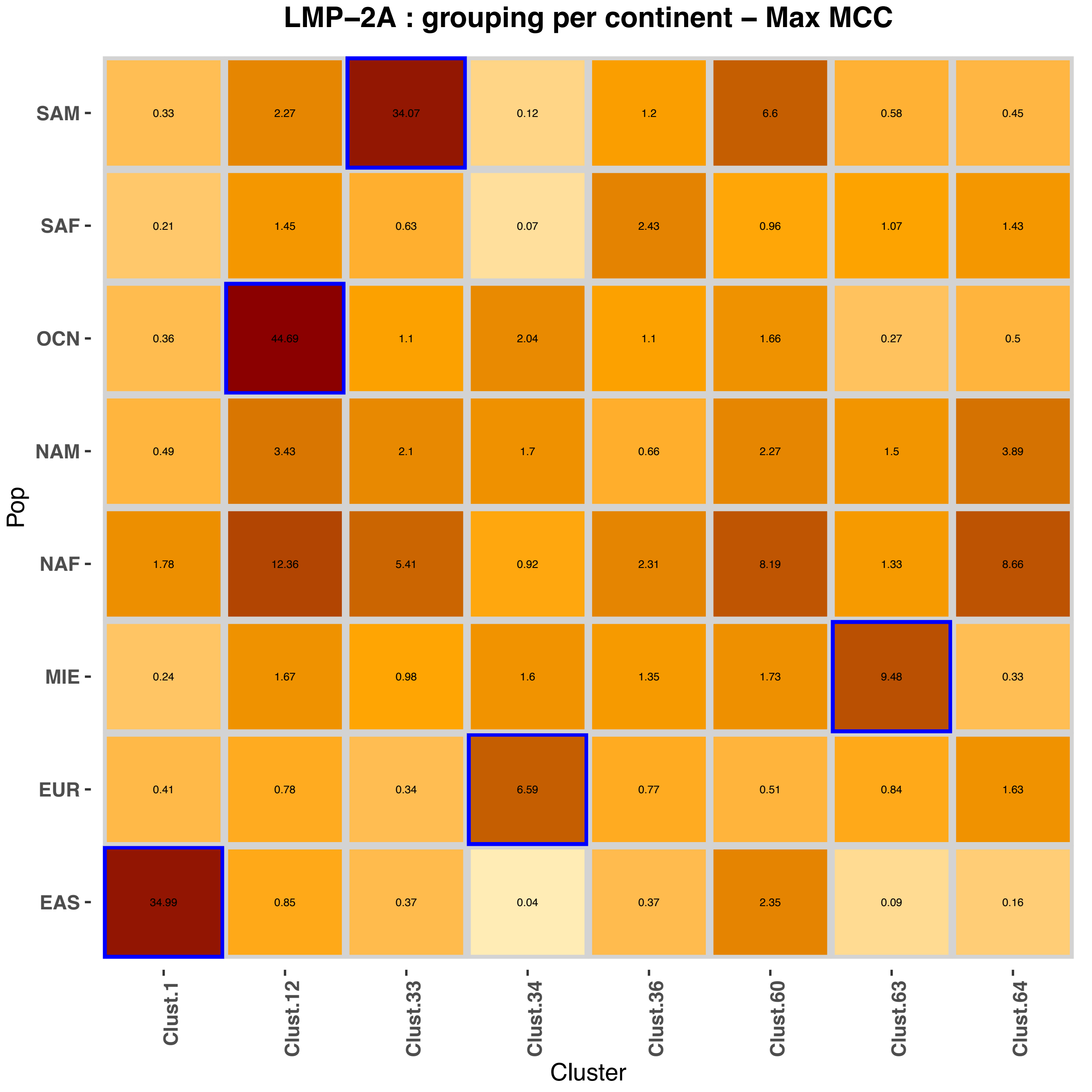

LMP−2A : grouping per continent − Max MCC
Pop
Cluster
SAM
SAF
OCN
NAM
NAF
MIE
EUR
EAS
Clust.1
Clust.12
Clust.33
Clust.34
Clust.36
Clust.60
Clust.63
Clust.64
0.33 2.27 34.07 0.12 1.2 6.6 0.58 0.45
0.21 1.45 0.63 0.07 2.43 0.96 1.07 1.43
0.36 44.69 1.1 2.04 1.1 1.66 0.27 0.5
0.49 3.43 2.1 1.7 0.66 2.27 1.5 3.89
1.78 12.36 5.41 0.92 2.31 8.19 1.33 8.66
0.24 1.67 0.98 1.6 1.35 1.73 9.48 0.33
0.41 0.78 0.34 6.59 0.77 0.51 0.84 1.63
34.99 0.85 0.37 0.04 0.37 2.35 0.09 0.16
