# Supplementary material for: Expanding the Geographic Characterisation of Epstein–Barr Virus Variation through Gene-Based Approaches
Source: Microorganisms. 2020 Oct 29;8(11):1686. doi: 10.3390/microorganisms8111686 (PMC7692309; doi:10.3390/microorganisms8111686)
Supplement: Supplementary file 1 [file microorganisms-08-01686-s001.zip › Supplementary/Supplementary_Figure_8.pdf]

Supplementary Figure 8

Gene's phylogenies selected clusterings. The gene phylogenies are reported coloured by country of origin, together with the same phylogenies coloured per max CGS clustering against country of origin, max CGS clustering against continent of origin, max MCC clustering against country of origin, and max MCC clustering against continent of origin. The black colour identifies isolates falling in clusters with less then 3 individuals, and that were in consequence excluded from the analysis.

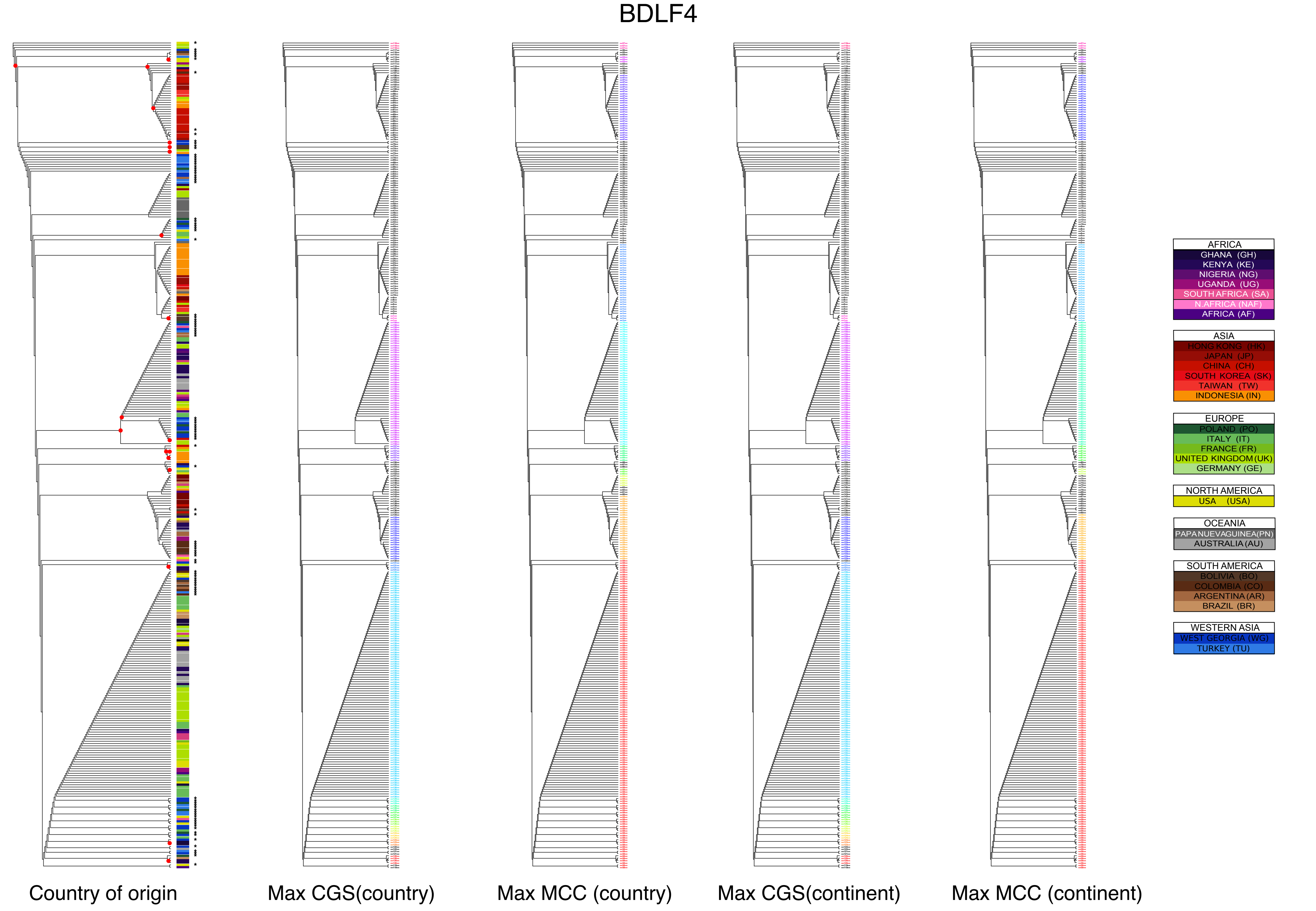

BDLF4
Country of origin
Max CGS(country)
Max MCC (country)
Max CGS(continent)
Max MCC (continent)
AFRICA
GHANA (GH)
KENYA (KE)
NIGERIA (NG)
UGANDA (UG)
SOUTH AFRICA (SA)
N.AFRICA (NAF)
AFRICA (AF)
ASIA
HONG KONG (HK)
JAPAN (JP)
CHINA (CH)
SOUTH KOREA (SK)
TAIWAN (TW)
INDONESIA (IN)
EUROPE
POLAND (PO)
ITALY (IT)
FRANCE (FR)
UNITED KINGDOM (UK)
GERMANY (GE)
NORTH AMERICA
USA (USA)
OCEANIA
PAPANUEVAGUINEA(PN)
AUSTRALIA (AU)
SOUTH AMERICA
BOLIVIA (BO)
COLOMBIA (CO)
ARGENTINA (AR)
BRAZIL (BR)
WESTERN ASIA
WEST GEORGIA (WG)
TURKEY (TU)

BGLF4

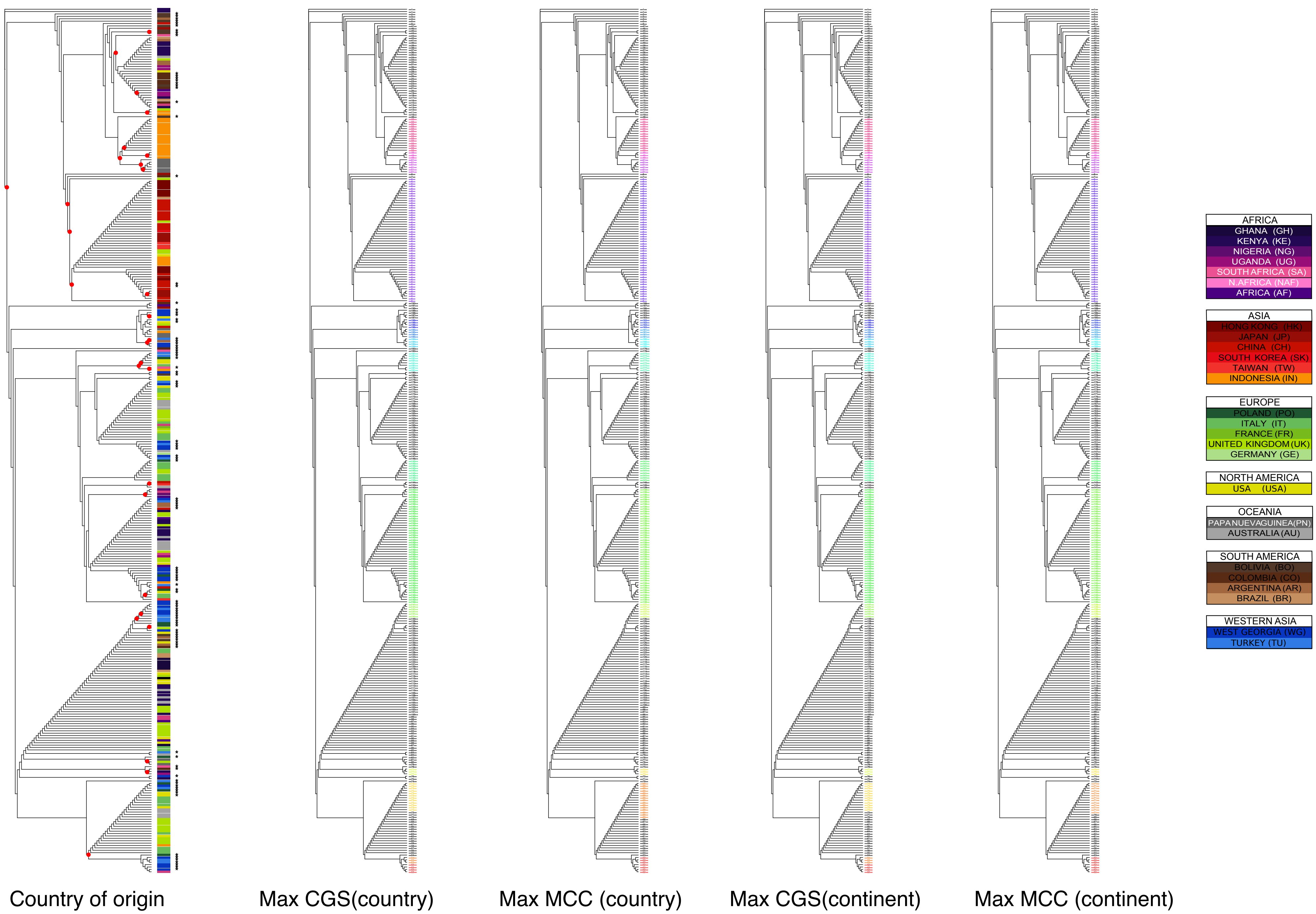

AFRICA
GHANA (GH)
KENYA (KE)
NIGERIA (NG)
UGANDA (UG)
SOUTH AFRICA (SA)
N.AFRICA (NAF)
AFRICA (AF)
ASIA
HONG KONG (HK)
JAPAN (JP)
CHINA (CH)
SOUTH KOREA (SK)
TAIWAN (TW)
INDONESIA (IN)
EUROPE
POLAND (PO)
ITALY (IT)
FRANCE (FR)
UNITED KINGDOM (UK)
GERMANY (GE)
NORTH AMERICA
USA (USA)
OCEANIA
PAPA NUEVA GUINEA (PN)
AUSTRALIA (AU)
SOUTH AMERICA
BOLIVIA (BO)
COLOMBIA (CO)
ARGENTINA (AR)
BRAZIL (BR)
WESTERN ASIA
WEST GEORGIA (WG)
TURKEY (TU)
Country of origin
Max CGS(country)
Max MCC (country)
Max CGS(continent)
Max MCC (continent)

BHRF1

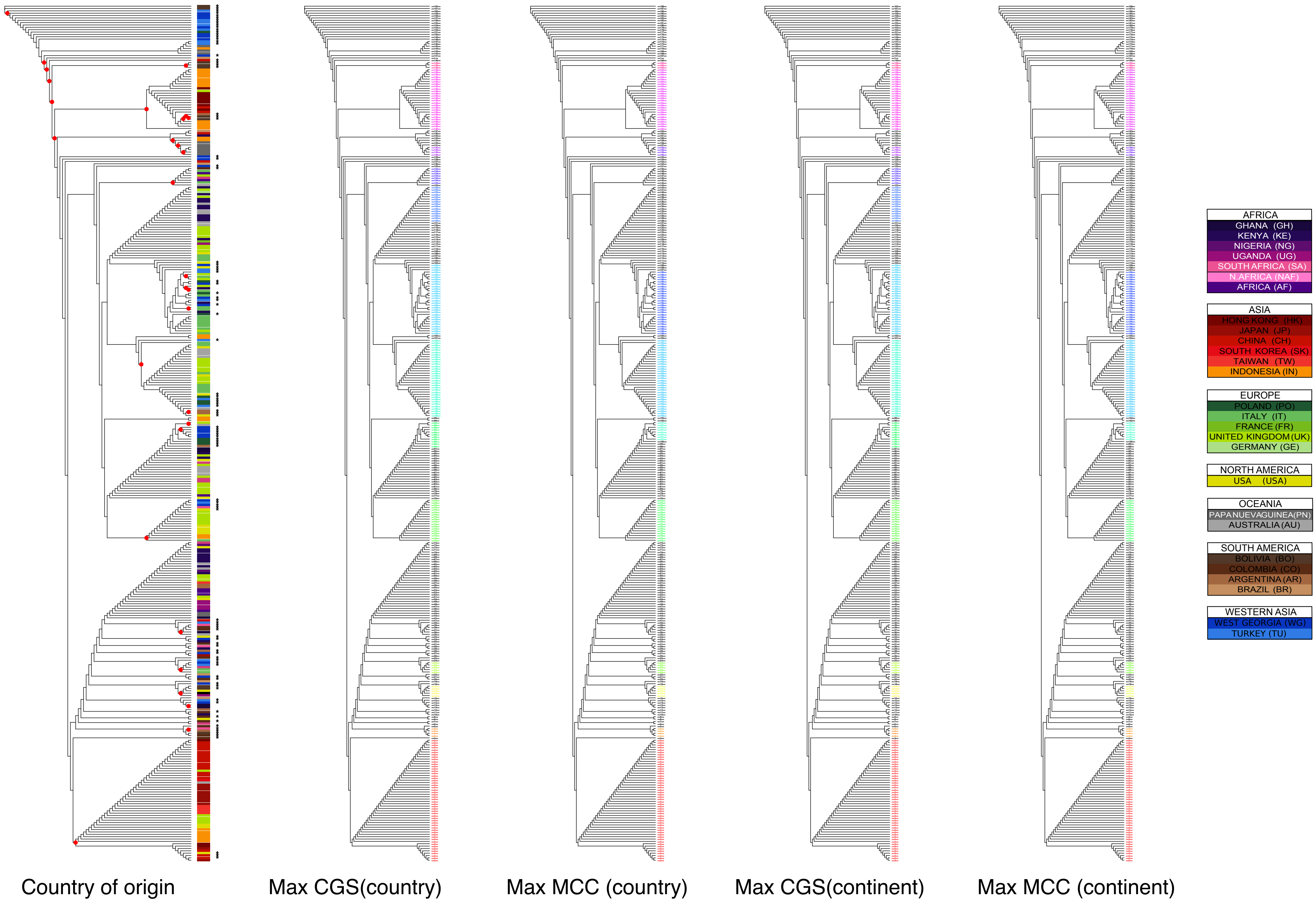

AFRICA
GHANA (GH)
KENYA (KE)
NIGERIA (NG)
UGANDA (UG)
SOUTH AFRICA (SA)
N.AFRICA (NAF)
AFRICA (AF)
ASIA
HONG KONG (HK)
JAPAN (JP)
CHINA (CH)
SOUTH KOREA (SK)
TAIWAN (TW)
INDONESIA (IN)
EUROPE
POLAND (PO)
ITALY (IT)
FRANCE (FR)
UNITED KINGDOM (UK)
GERMANY (GE)
NORTH AMERICA
USA (USA)
OCEANIA
PAPA NUEVAGUINEA(PN)
AUSTRALIA (AU)
SOUTH AMERICA
BOLIVIA (BO)
COLOMBIA (CO)
ARGENTINA (AR)
BRAZIL (BR)
WESTERN ASIA
WEST GEORGIA (WG)
TURKEY (TU)
Country of origin
Max CGS(country)
Max MCC (country)
Max CGS(continent)
Max MCC (continent)

BRLF1

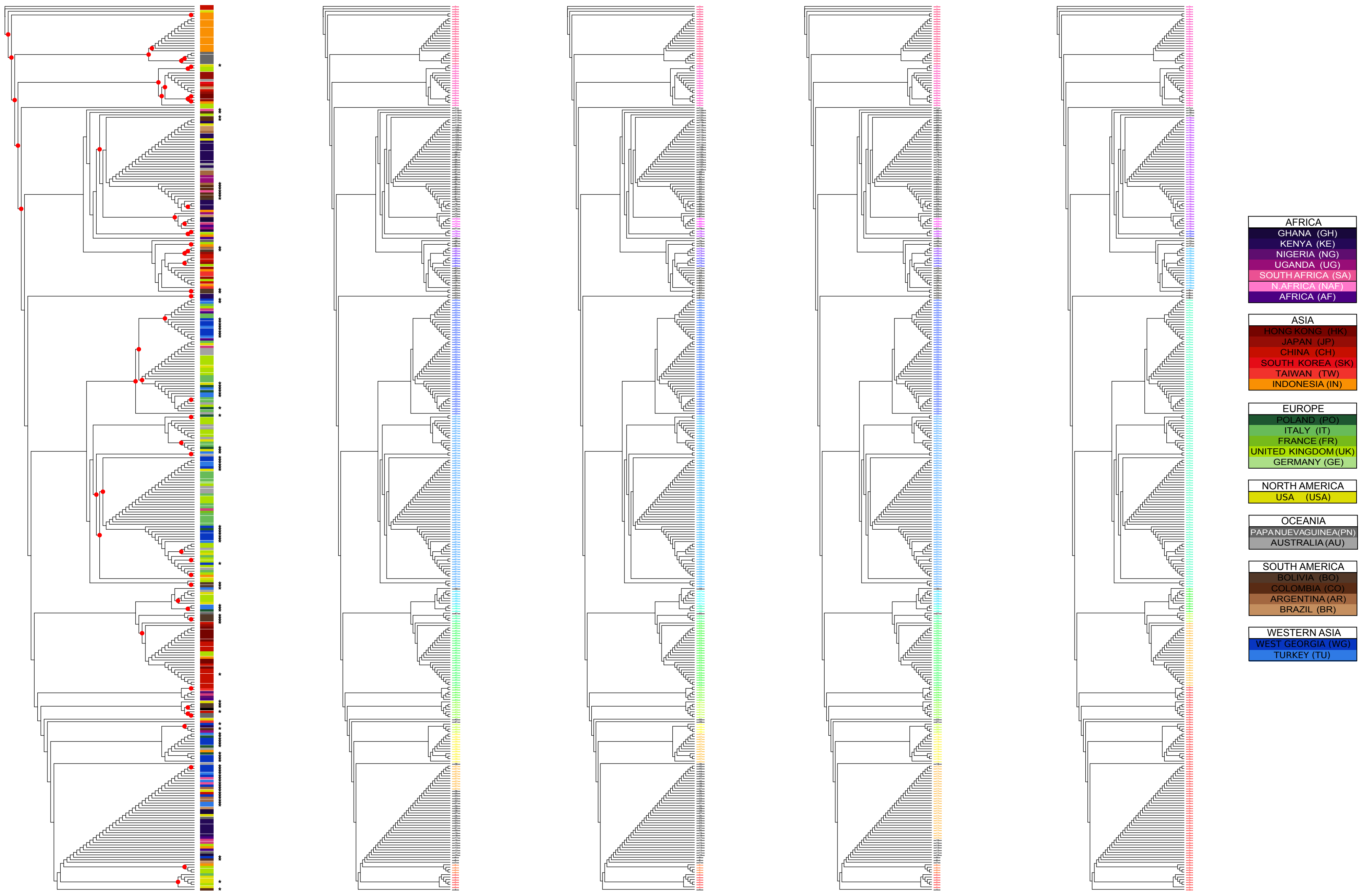

Country of origin | Max CGS(country) | Max MCC (country) | Max CGS(continent) | Max MCC (continent)

BZLF1

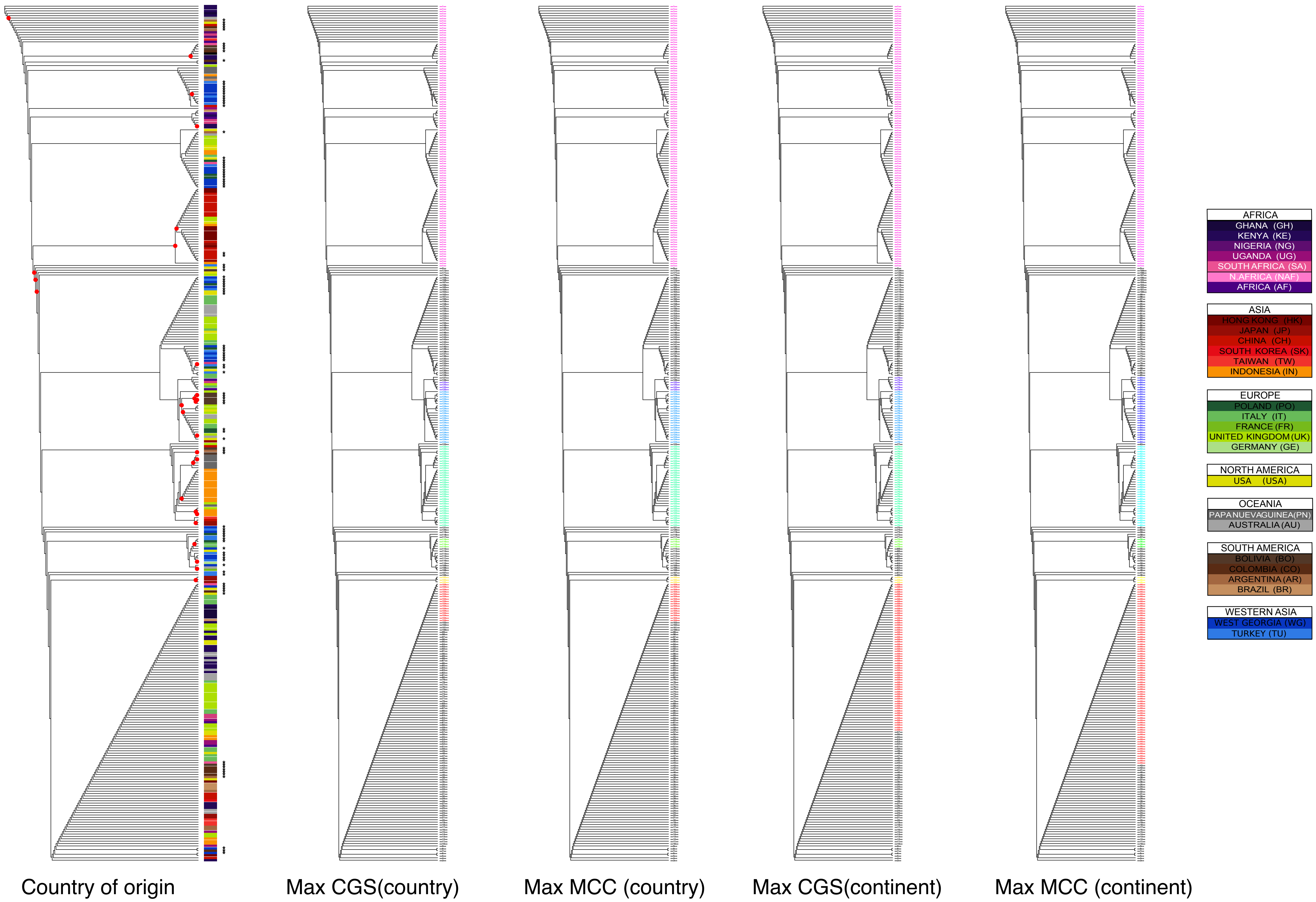
AFRICA
GHANA (GH)
KENYA (KE)
NIGERIA (NG)
UGANDA (UG)
SOUTH AFRICA (SA)
N.AFRICA (NAF)
AFRICA (AF)
ASIA
HONG KONG (HK)
JAPAN (JP)
CHINA (CH)
SOUTH KOREA (SK)
TAIWAN (TW)
INDONESIA (IN)
EUROPE
POLAND (PO)
ITALY (IT)
FRANCE (FR)
UNITED KINGDOM (UK)
GERMANY (GE)
NORTH AMERICA
USA (USA)
OCEANIA
PAPA NUEVAGUINEA(PN)
AUSTRALIA (AU)
SOUTH AMERICA
BOLIVIA (BO)
COLOMBIA (CO)
ARGENTINA (AR)
BRAZIL (BR)
WESTERN ASIA
WEST GEORGIA (WG)
TURKEY (TU)
Country of origin
Max CGS(country)
Max MCC (country)
Max CGS(continent)
Max MCC (continent)

EBER-1

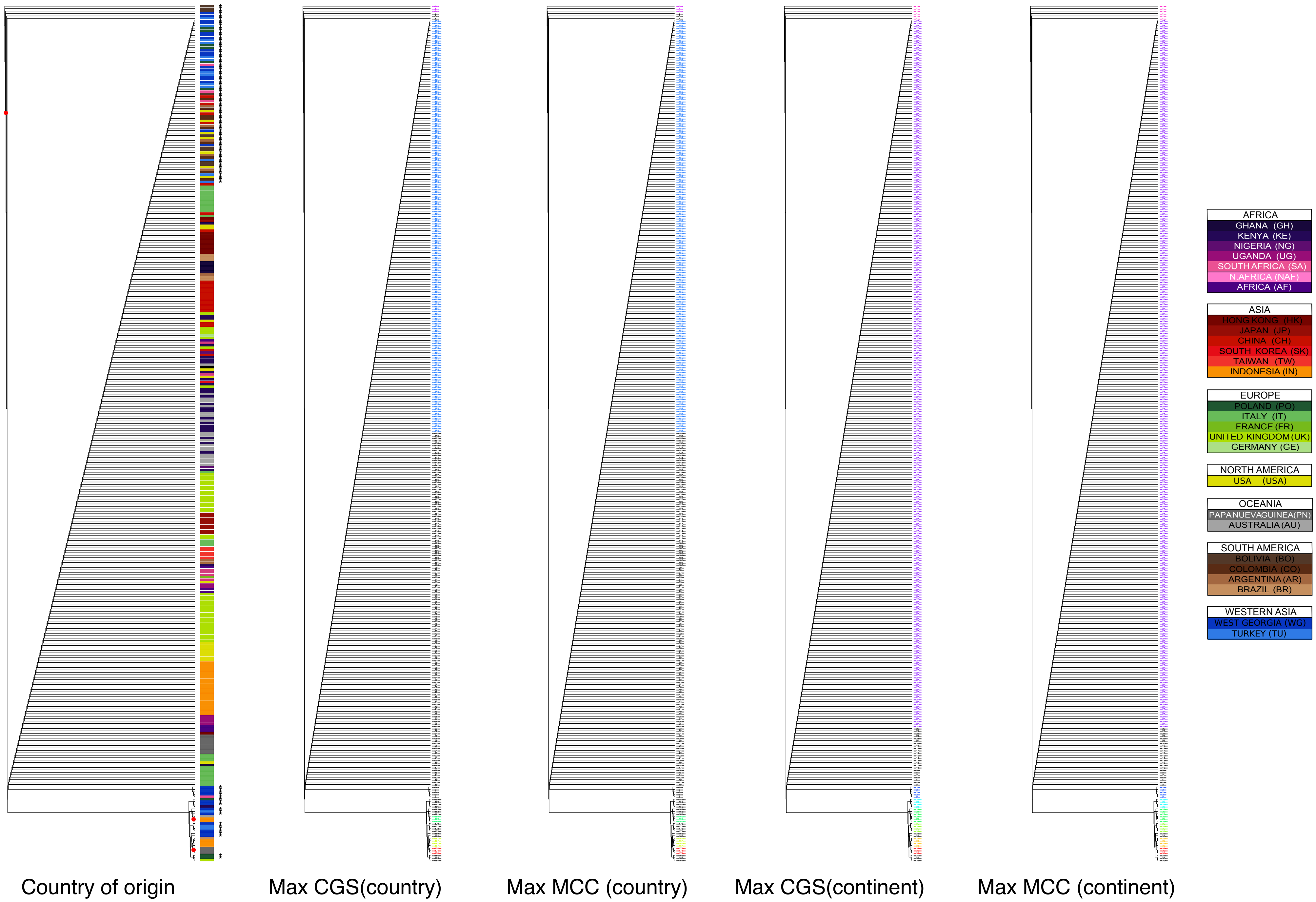

AFRICA
GHANA (GH)
KENYA (KE)
NIGERIA (NG)
UGANDA (UG)
SOUTH AFRICA (SA)
N.AFRICA (NAF)
AFRICA (AF)
ASIA
HONG KONG (HK)
JAPAN (JP)
CHINA (CH)
SOUTH KOREA (SK)
TAIWAN (TW)
INDONESIA (IN)
EUROPE
POLAND (PO)
ITALY (IT)
FRANCE (FR)
UNITED KINGDOM (UK)
GERMANY (GE)
NORTH AMERICA
USA (USA)
OCEANIA
PAPA NUEVA GUINEA(PN)
AUSTRALIA (AU)
SOUTH AMERICA
BOLIVIA (BO)
COLOMBIA (CO)
ARGENTINA (AR)
BRAZIL (BR)
WESTERN ASIA
WEST GEORGIA (WG)
TURKEY (TU)
Country of origin
Max CGS(country)
Max MCC (country)
Max CGS(continent)
Max MCC (continent)

EBER-2

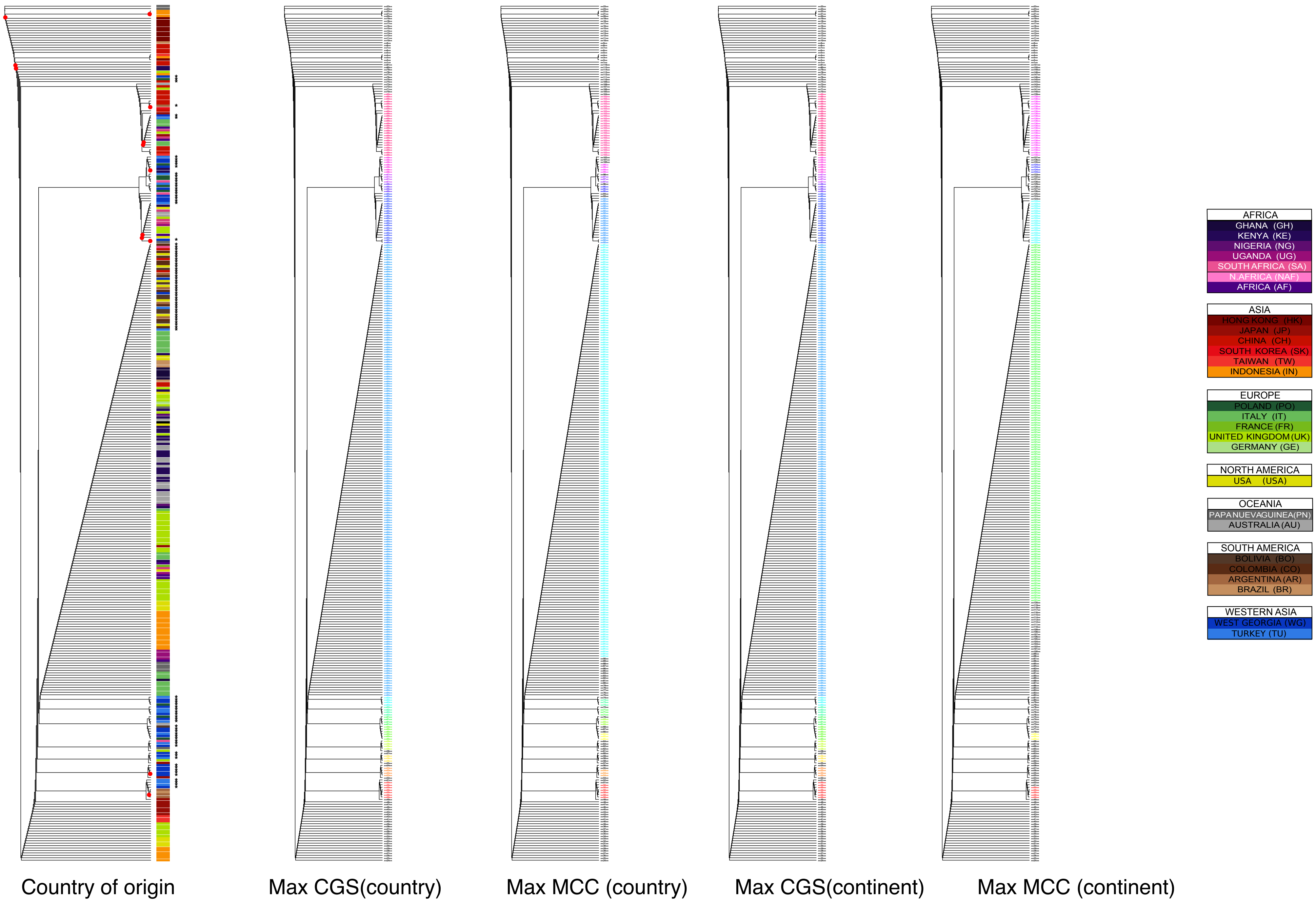
AFRICA
GHANA (GH)
KENYA (KE)
NIGERIA (NG)
UGANDA (UG)
SOUTH AFRICA (SA)
N.AFRICA (NAF)
AFRICA (AF)
ASIA
HONG KONG (HK)
JAPAN (JP)
CHINA (CH)
SOUTH KOREA (SK)
TAIWAN (TW)
INDONESIA (IN)
EUROPE
POLAND (PO)
ITALY (IT)
FRANCE (FR)
UNITED KINGDOM (UK)
GERMANY (GE)
NORTH AMERICA
USA (USA)
OCEANIA
PAPA NUEVAGUINEA(PN)
AUSTRALIA (AU)
SOUTH AMERICA
BOLIVIA (BO)
COLOMBIA (CO)
ARGENTINA (AR)
BRAZIL (BR)
WESTERN ASIA
WEST GEORGIA (WG)
TURKEY (TU)
Country of origin
Max CGS(country)
Max MCC (country)
Max CGS(continent)
Max MCC (continent)

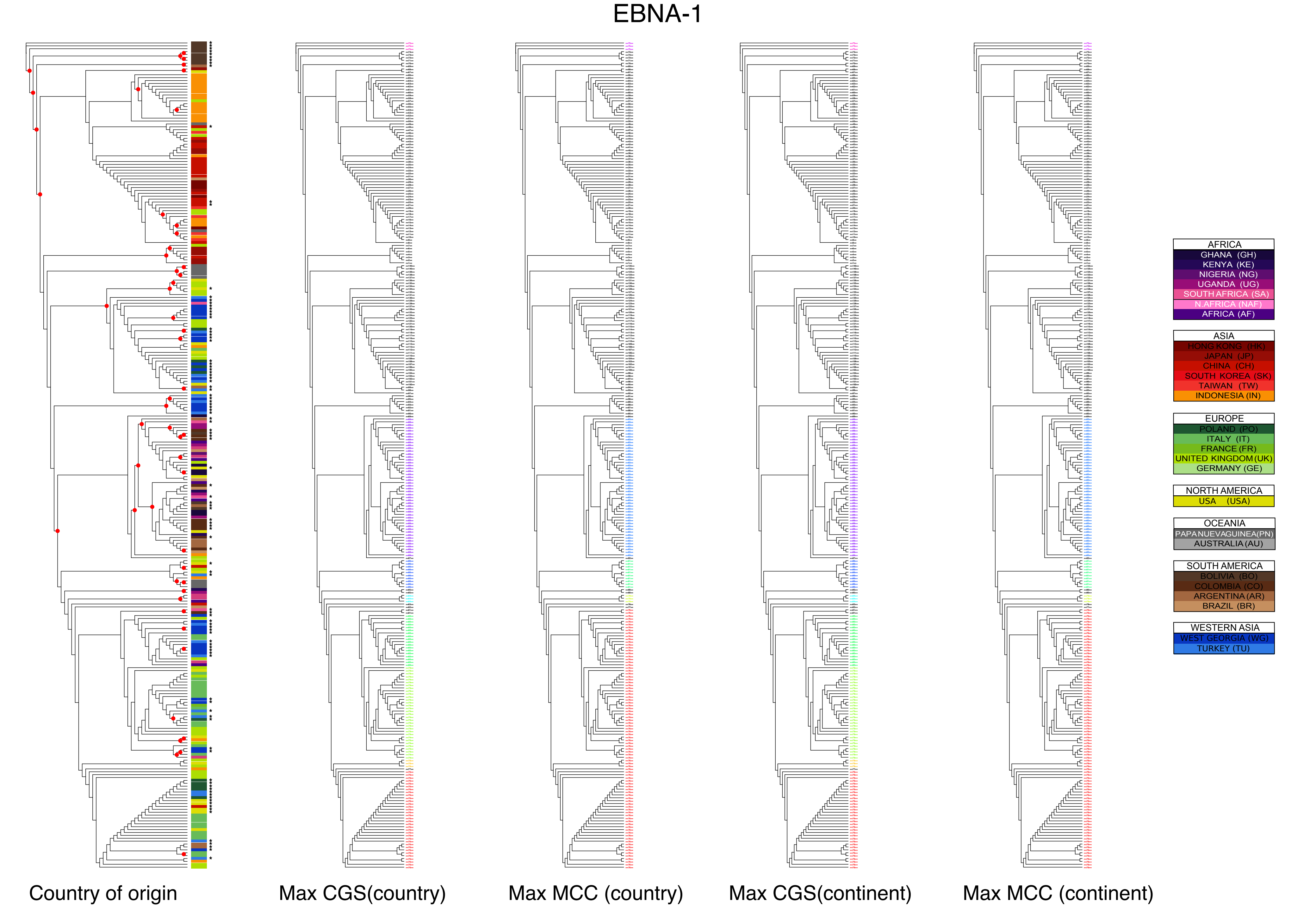

EBNA-1
AFRICA
GHANA (GH)
KENYA (KE)
NIGERIA (NG)
UGANDA (UG)
SOUTH AFRICA (SA)
N.AFRICA (NAF)
AFRICA (AF)
ASIA
HONG KONG (HK)
JAPAN (JP)
CHINA (CH)
SOUTH KOREA (SK)
TAIWAN (TW)
INDONESIA (IN)
EUROPE
POLAND (PO)
ITALY (IT)
FRANCE (FR)
UNITED KINGDOM (UK)
GERMANY (GE)
NORTH AMERICA
USA (USA)
OCEANIA
PAPANUEVAGUINEA(PN)
AUSTRALIA (AU)
SOUTH AMERICA
BOLIVIA (BO)
COLOMBIA (CO)
ARGENTINA (AR)
BRAZIL (BR)
WESTERN ASIA
WEST GEORGIA (WG)
TURKEY (TU)
Country of origin
Max CGS(country)
Max MCC (country)
Max CGS(continent)
Max MCC (continent)

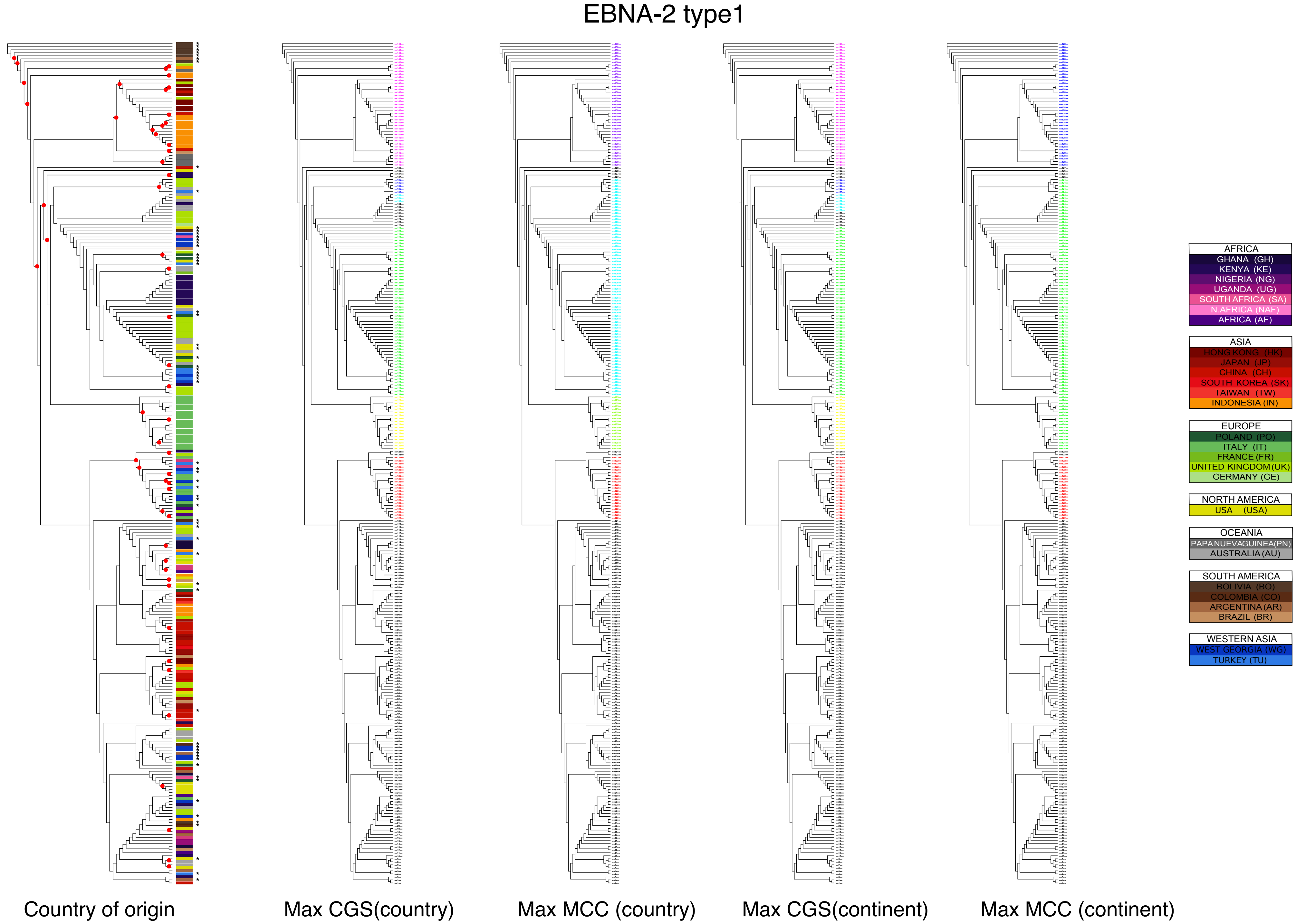
EBNA-2 type1
Country of origin
Max CGS(country)
Max MCC (country)
Max CGS(continent)
Max MCC (continent)
AFRICA
GHANA (GH)
KENYA (KE)
NIGERIA (NG)
UGANDA (UG)
SOUTH AFRICA (SA)
N.AFRICA (NAF)
AFRICA (AF)
ASIA
HONG KONG (HK)
JAPAN (JP)
CHINA (CH)
SOUTH KOREA (SK)
TAIWAN (TW)
INDONESIA (IN)
EUROPE
POLAND (PO)
ITALY (IT)
FRANCE (FR)
UNITED KINGDOM (UK)
GERMANY (GE)
NORTH AMERICA
USA (USA)
OCEANIA
PAPA NUEVAGUINEA (PN)
AUSTRALIA (AU)
SOUTH AMERICA
BOLIVIA (BO)
COLOMBIA (CO)
ARGENTINA (AR)
BRAZIL (BR)
WESTERN ASIA
WEST GEORGIA (WG)
TURKEY (TU)

EBNA-3A type1

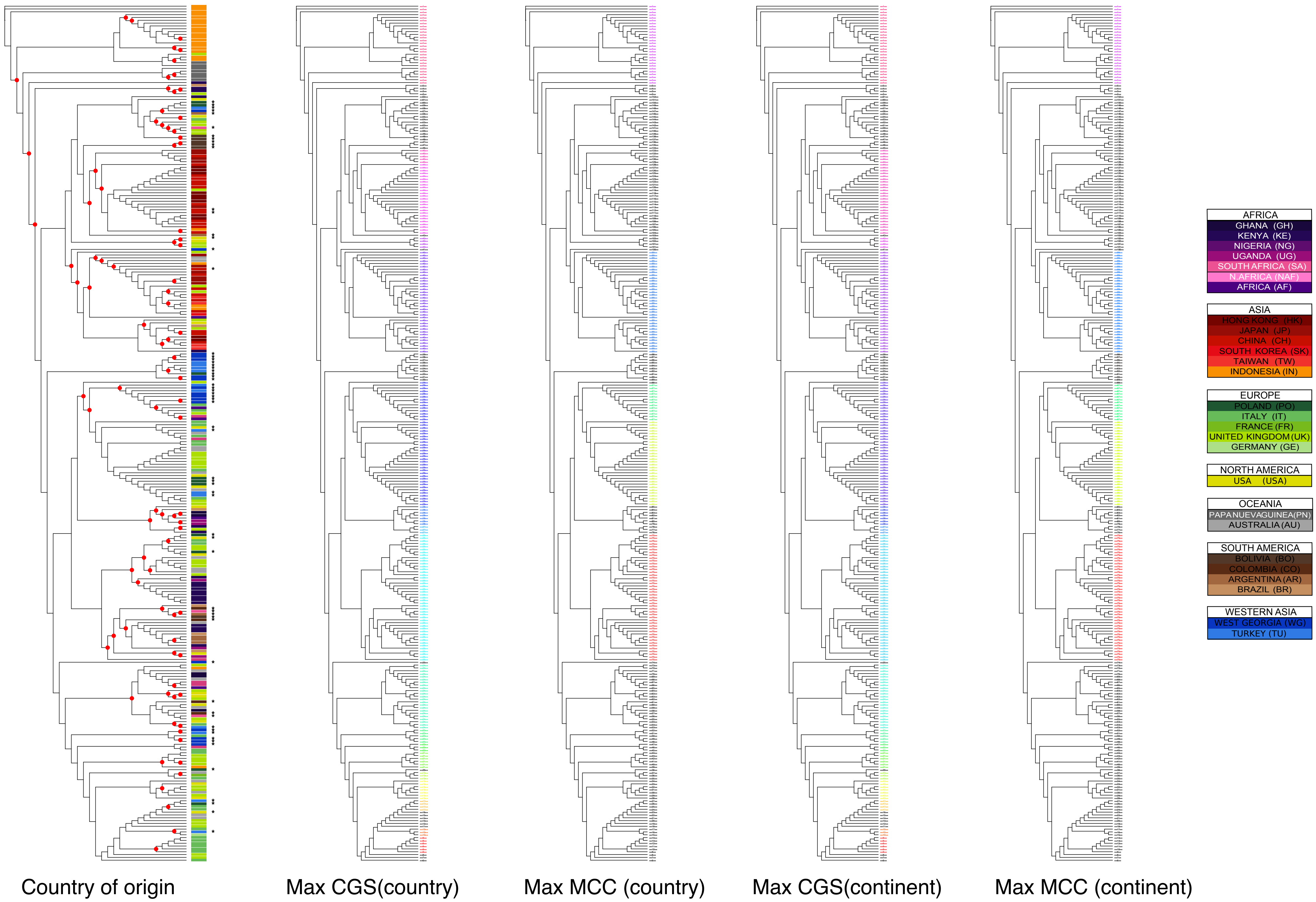

EBNA-3B type1

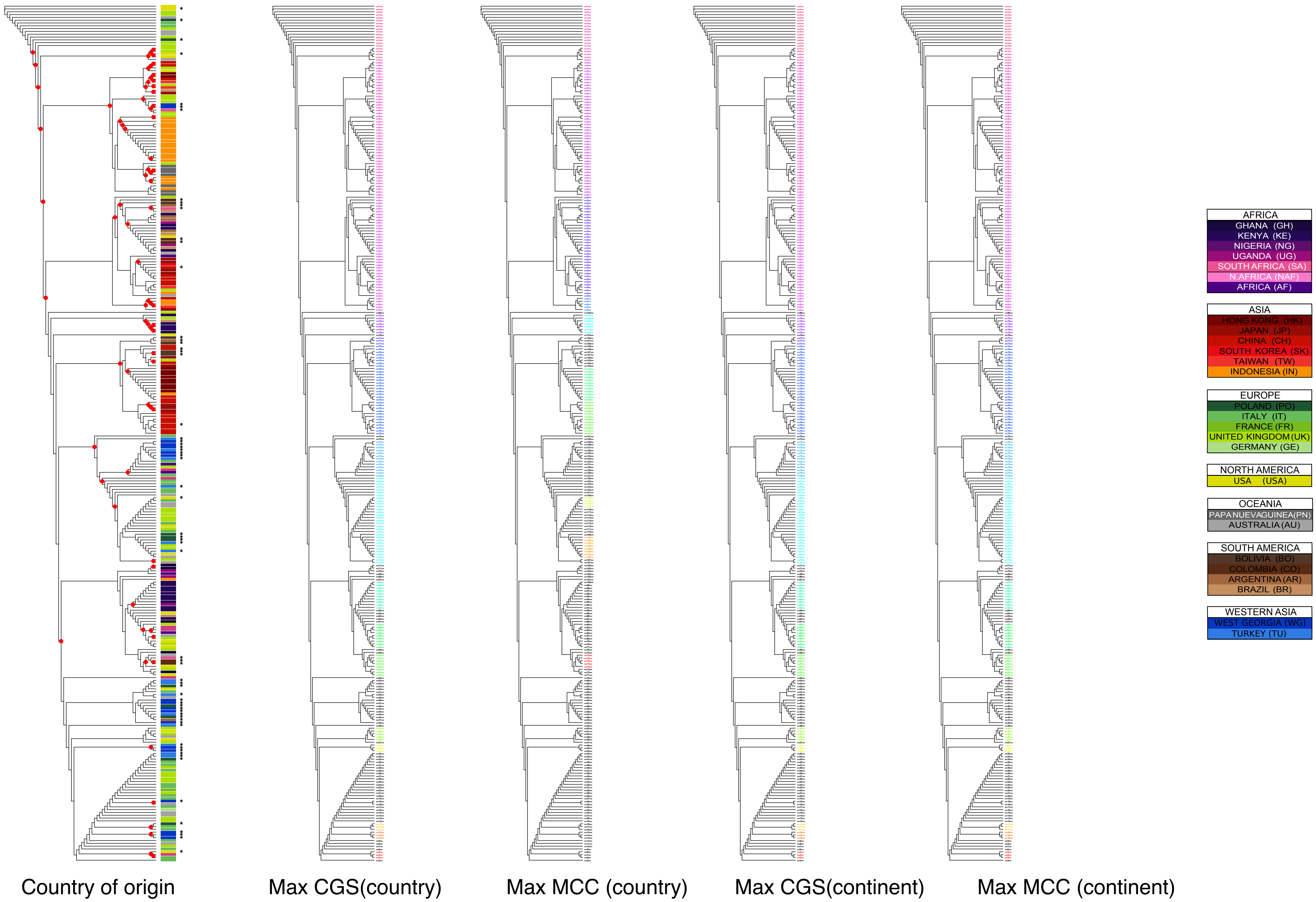

EBNA-3C type1

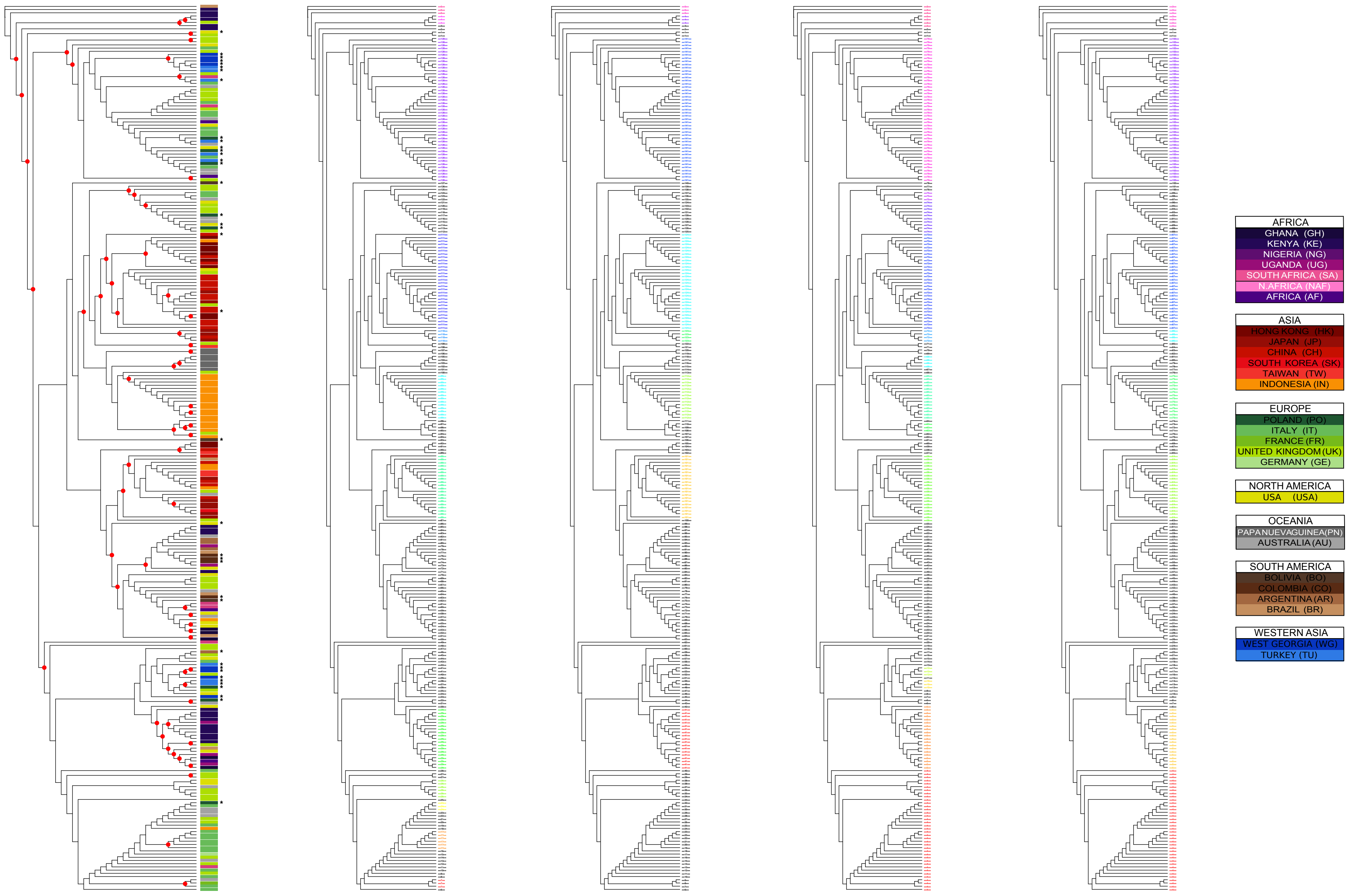

Country of origin | Max CGS(country) | Max MCC (country) | Max CGS(continent) | Max MCC (continent)

LMP-2A

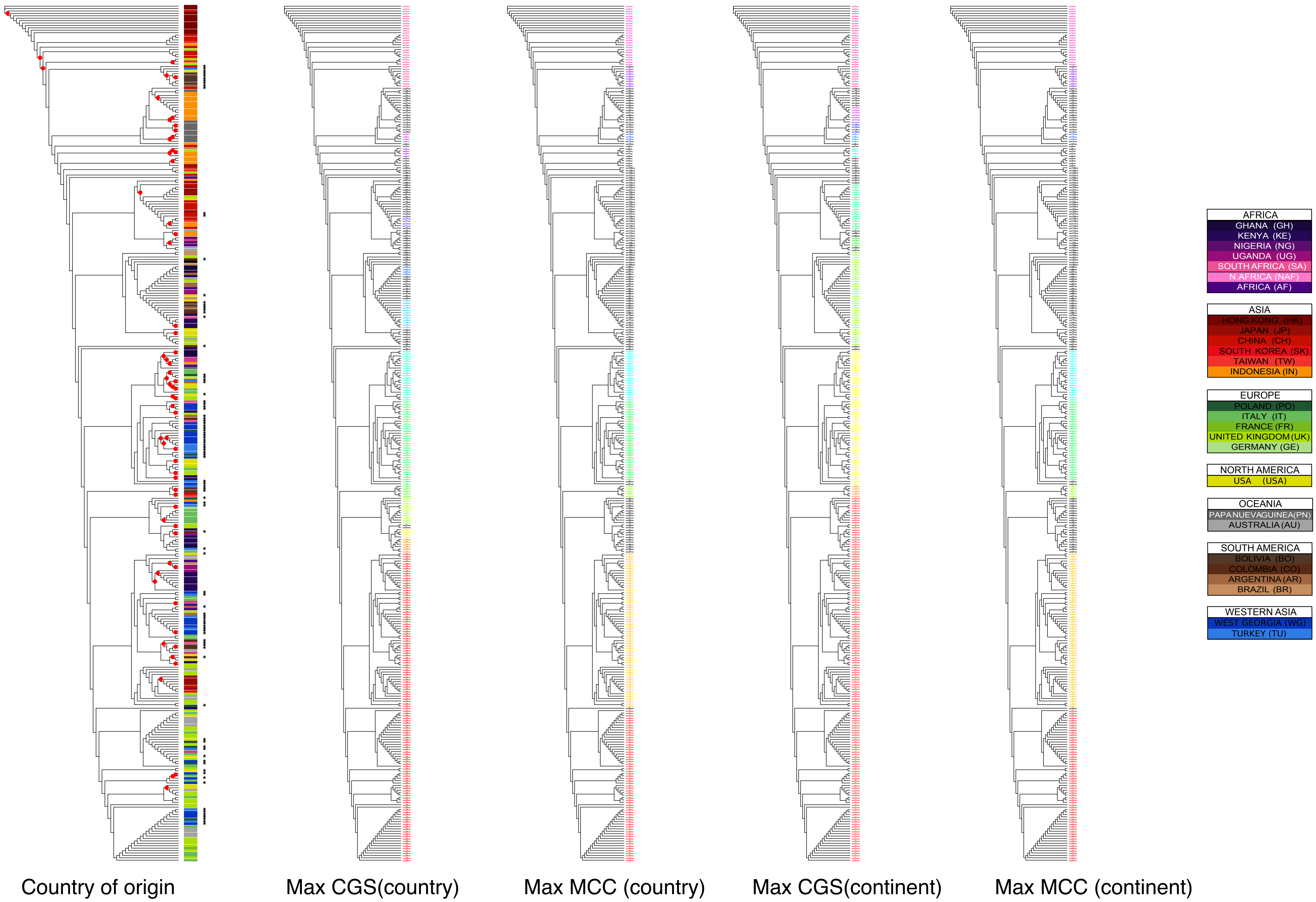

Country of origin
Max CGS(country)
Max MCC (country)
Max CGS(continent)
Max MCC (continent)
AFRICA
GHANA (GH)
KENYA (KE)
NIGERIA (NG)
UGANDA (UG)
SOUTH AFRICA (SA)
N.AFRICA (NAF)
AFRICA (AF)
ASIA
HONG KONG (HK)
JAPAN (JP)
CHINA (CH)
SOUTH KOREA (SK)
TAIWAN (TW)
INDONESIA (IN)
EUROPE
POLAND (PO)
ITALY (IT)
FRANCE (FR)
UNITED KINGDOM (UK)
GERMANY (GE)
NORTH AMERICA
USA (USA)
OCEANIA
PAPA NUEVAGUINEA(PN)
AUSTRALIA (AU)
SOUTH AMERICA
BOLIVIA (BO)
COLOMBIA (CO)
ARGENTINA (AR)
BRAZIL (BR)
WESTERN ASIA
WEST GEORGIA (WG)
TURKEY (TU)
