# Supplementary material for: Expanding the Geographic Characterisation of Epstein–Barr Virus Variation through Gene-Based Approaches
Source: Microorganisms. 2020 Oct 29;8(11):1686. doi: 10.3390/microorganisms8111686 (PMC7692309; doi:10.3390/microorganisms8111686)
Supplement: Supplementary file 1 [file microorganisms-08-01686-s001.zip › Supplementary/Supplementary_Figure_9.pdf]

Supplementary Figure 9

***EBNA-2* "CTC" insertion compared to geography.** *EBNA-2* phylogenetic tree coloured **a)** by country of origin, **b)** and by subtypes defined by the presence/absence and form of the "CTC" insertion.

***BHRF1* typing.** *BHRF1* maximum likelihood phylogenetic tree coloured by country of origin and *BHRF1* type.

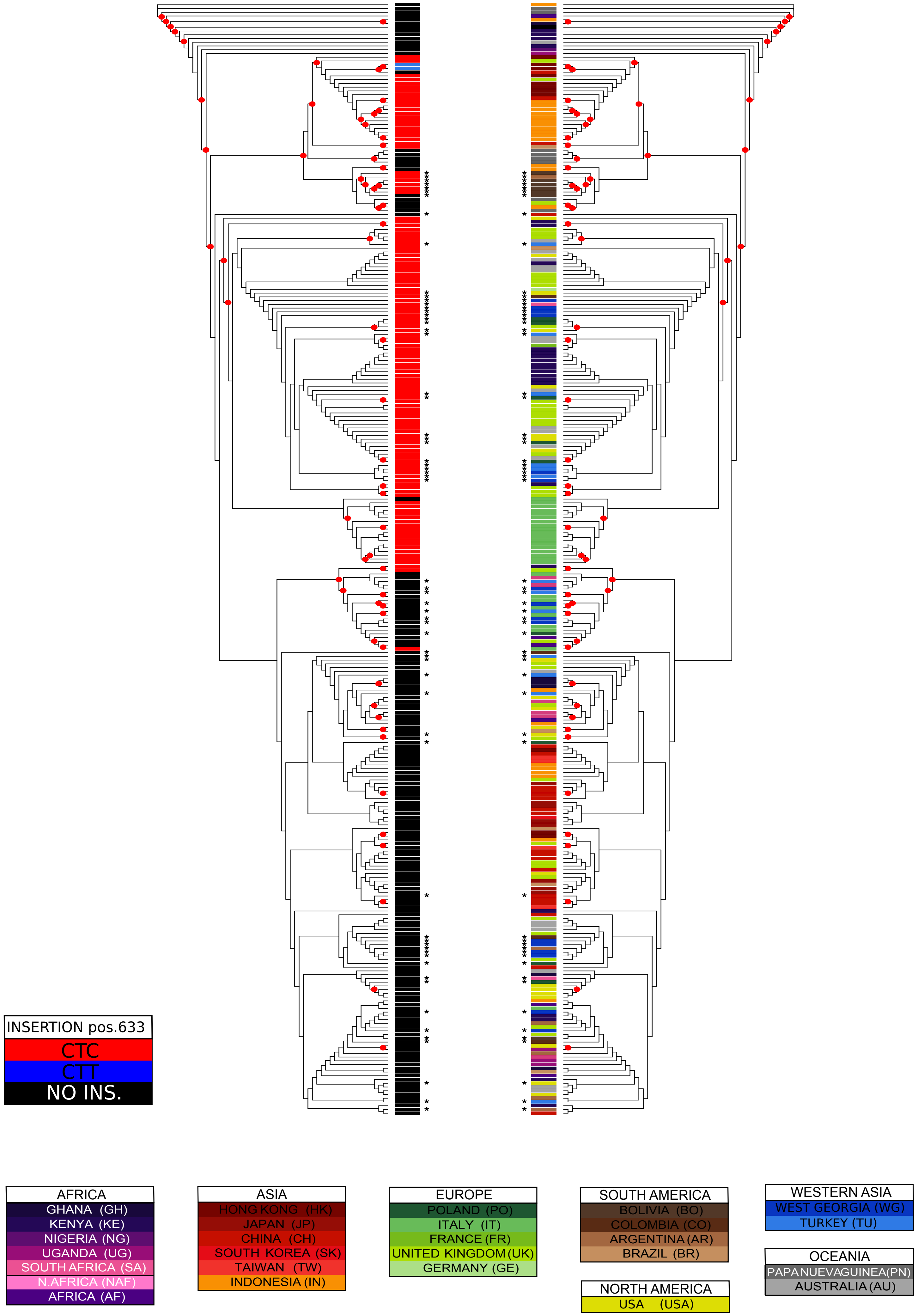
INSERTION pos.633
CTC
CTT
NO INS.
AFRICA
GHANA (GH)
KENYA (KE)
NIGERIA (NG)
UGANDA (UG)
SOUTH AFRICA (SA)
N.AFRICA (NAF)
AFRICA (AF)
ASIA
HONG KONG (HK)
JAPAN (JP)
CHINA (CH)
SOUTH KOREA (SK)
TAIWAN (TW)
INDONESIA (IN)
EUROPE
POLAND (PO)
ITALY (IT)
FRANCE (FR)
UNITED KINGDOM (UK)
GERMANY (GE)
SOUTH AMERICA
BOLIVIA (BO)
COLOMBIA (CO)
ARGENTINA (AR)
BRAZIL (BR)
NORTH AMERICA
USA (USA)
WESTERN ASIA
WEST GEORGIA (WG)
TURKEY (TU)
OCEANIA
PAPA NUEVAGUINEA(PN)
AUSTRALIA (AU)

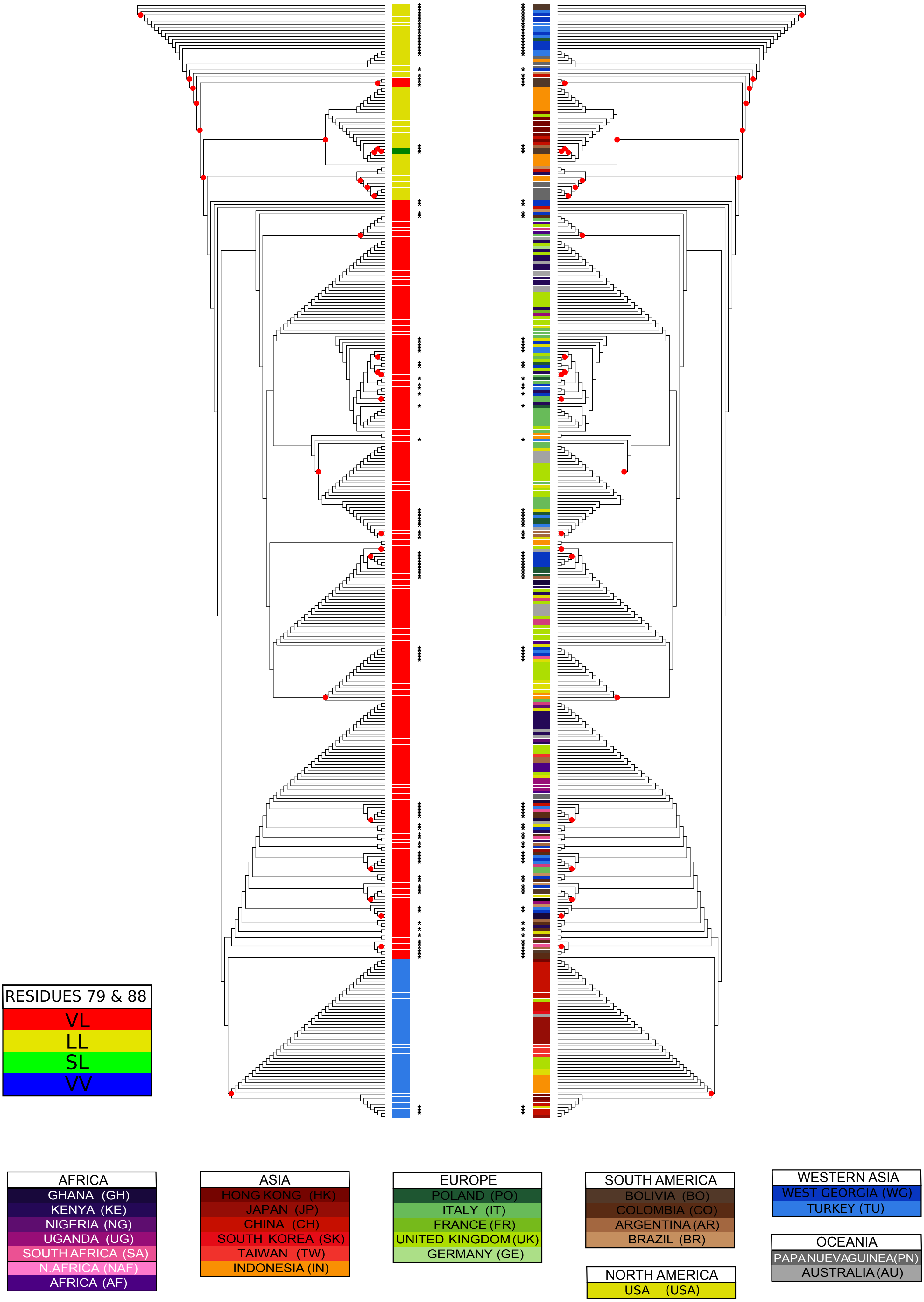

RESIDUES 79 & 88
VL
LL
SL
VV
AFRICA
GHANA (GH)
KENYA (KE)
NIGERIA (NG)
UGANDA (UG)
SOUTH AFRICA (SA)
N.AFRICA (NAF)
AFRICA (AF)
ASIA
HONG KONG (HK)
JAPAN (JP)
CHINA (CH)
SOUTH KOREA (SK)
TAIWAN (TW)
INDONESIA (IN)
EUROPE
POLAND (PO)
ITALY (IT)
FRANCE (FR)
UNITED KINGDOM (UK)
GERMANY (GE)
SOUTH AMERICA
BOLIVIA (BO)
COLOMBIA (CO)
ARGENTINA (AR)
BRAZIL (BR)
NORTH AMERICA
USA (USA)
WESTERN ASIA
WEST GEORGIA (WG)
TURKEY (TU)
OCEANIA
PAPA NUEVAGUINEA (PN)
AUSTRALIA (AU)
